# Divergent, Strain‐Release Reactions of Azabicyclo[1.1.0]butyl Carbinols: Semipinacol or Spiroepoxy Azetidine Formation

**DOI:** 10.1002/anie.202100583

**Published:** 2021-02-26

**Authors:** Charlotte H. U. Gregson, Adam Noble, Varinder K. Aggarwal

**Affiliations:** ^1^ School of Chemistry University of Bristol Cantock's Close Bristol BS8 1TS UK

**Keywords:** azabicyclo[1.1.0]butane, azetidines, epoxides, ring expansion, strained molecules

## Abstract

The azetidine moiety is a privileged motif in medicinal chemistry and new methods that access them efficiently are highly sought after. Towards this goal, we have found that azabicyclo[1.1.0]butyl carbinols, readily obtained from the highly strained azabicyclo[1.1.0]butane (ABB), can undergo divergent strain‐release reactions upon N‐activation. Treatment with trifluoroacetic anhydride or triflic anhydride triggered a semipinacol rearrangement to give keto 1,3,3‐substituted azetidines. More than 20 examples were explored, enabling us to evaluate selectivity and the migratory aptitude of different groups. Alternatively, treatment of the same alcohols with benzyl chloroformate in the presence of NaI led to iodohydrin intermediates which gave spiroepoxy azetidines upon treatment with base. The electronic nature of the activating agent dictates which pathway operates.

## Graphical abstract

Azabicyclo[1.1.0]butyl carbinols can be converted into a diverse range of 1,3,3‐substituted azetidines or spirocyclic epoxides through either a semipinacol rearrangement or via iodohydrin formation followed by spirocyclization. The reaction pathway that operates is determined by the electronic nature of the activating agent.

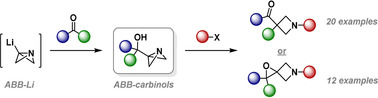

## Introduction

**Scheme 1:**
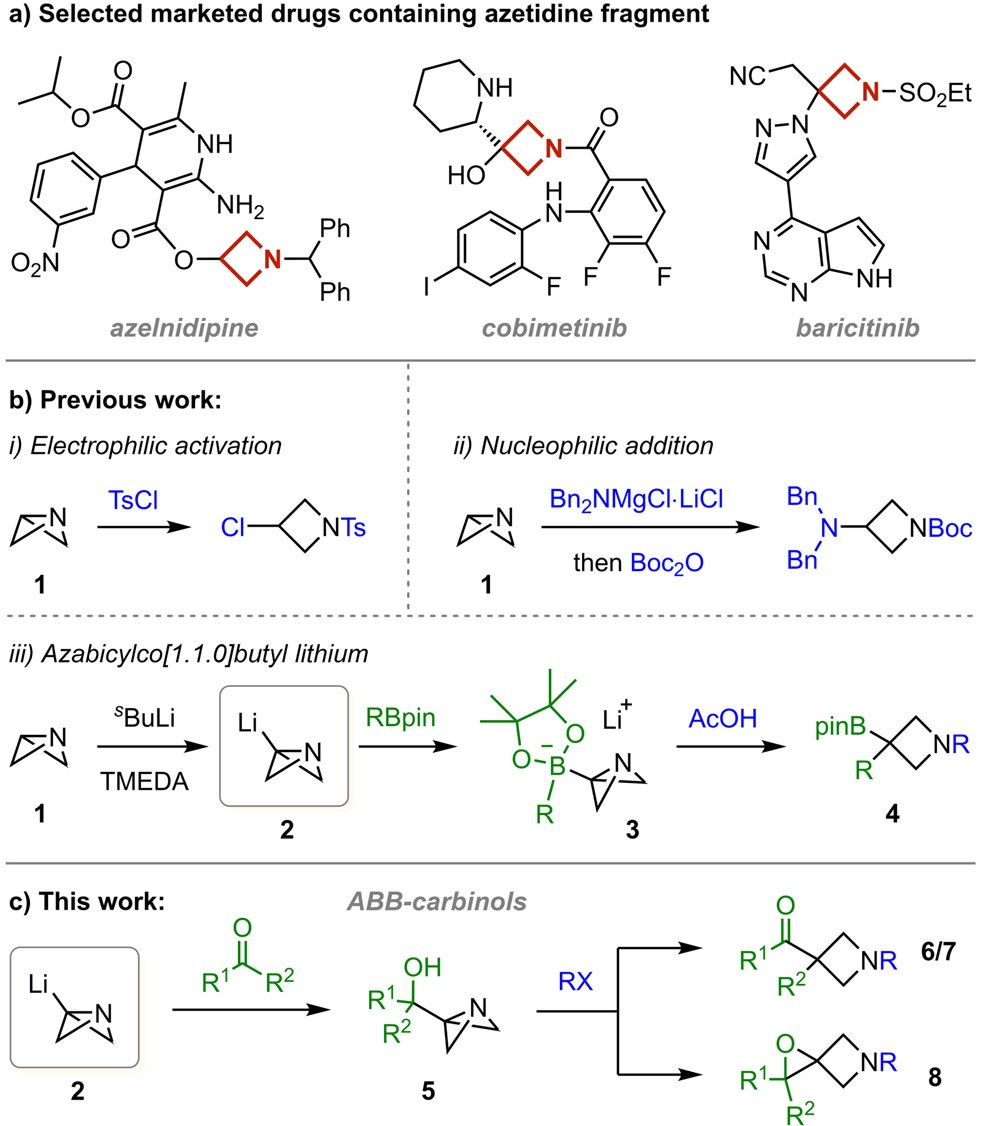
a) Marketed drugs containing 1,3‐substituted azetidine scaffolds. b) Electrophilic activation of ABB, nucleophilic addition to ABB and lithiation of ABB followed by reaction with boronic esters. c) Reaction of ABB‐Li with carbonyls and subsequent divergent reactivity.

The past decade has seen a surge in the application of azetidines in pharmaceutical drug candidates.^[1]^ This is due in part to their improved pharmacokinetics in comparison to their larger ring analogues: molecules with the azetidine motif were shown to exhibit greater metabolic stability and increased bioavailability.^[2]^ 1,3‐Substituted azetidines also benefit from having 3D character, which has been shown to improve clinical success,^[3]^ but without introducing chirality and so are more amenable to synthesis. This specific substitution pattern is featured on the azetidine scaffold of the marketed drugs azelnidipine,^[4]^ cobimetinib^[1a]^ and baricitinib^[1b]^ (Scheme 1 a).

An emerging method for the construction of 1,3‐substituted azetidines is the use of azabicyclo[1.1.0]butane (ABB, **1**).[^5^, ^6^] First prepared in 1969,^[7]^ the highly strained building block was shown to react with electrophiles such as tosyl chloride,^[5a]^ through activation of the ABB nitrogen, followed by nucleophilic addition at the bridgehead carbon (Scheme 1 b). More recently, it was demonstrated that ABB could react with strong nucleophiles such as turbo amides (Scheme 1 b)^[5b]^ or organocuprates^[5d]^ to enable rapid access to diverse 1,3‐substituted azetidines. We recently reported the preparation of azabicyclo[1.1.0]butyllithium (ABB‐Li, **2**) by the facile lithiation of **1** at the bridgehead position with TMEDA‐ligated *sec*‐butyllithium (Scheme 1 b).^[8a]^ ABB‐Li resembles a carbenoid as it is a nucleophile with inherent electrophilicity due to the strained structure. We subsequently employed **2** in reactions with boronic esters to give boronate complexes **3** that underwent strain‐release 1,2‐rearrangement^[8]^ upon activation of the ABB nitrogen with acetic acid. This alternative umpolung‐type reactivity of ABB enabled the synthesis of 1,3,3‐substituted azetidines (**4**) bearing a versatile tertiary boronic ester.

We wanted to further develop the use of **2** as a synthon for 1,3,3‐substituted azetidines by exploring alternative electrophiles which could also trigger strain‐release reactivity and considered the use of ketones and aldehydes (Scheme 1 c). We envisaged that after formation of azabicyclo[1.1.0]butyl carbinols (ABB‐carbinols, **5**), subsequent N‐activation by a suitable electrophile could trigger a pinacol‐type rearrangement, cleaving the bridging C−N bond and releasing ring strain.[^9^, ^10^] Such a pinacol‐type rearrangement is analogous to the semipinacol rearrangement of α‐hydroxy epoxides.^[11]^ This would lead to azetidines bearing a quaternary centre (**6**/**7**). Alternatively, N‐activation of ABB‐carbinols could result in a nucleophilic addition of the alcohol to form spiroepoxy azetidines (**8**)[^1a^, ^12^, ^13^] which would be interesting synthetic targets as they could display further strain‐release reactivity.^[12]^ In this paper, we report our success in discovering two divergent pathways from a common ABB‐carbinol intermediate that lead to either 1,3,3‐substituted azetidines, via a semipinacol rearrangement, or spirocyclic epoxides.

## Results and Discussion

**Scheme 2:**
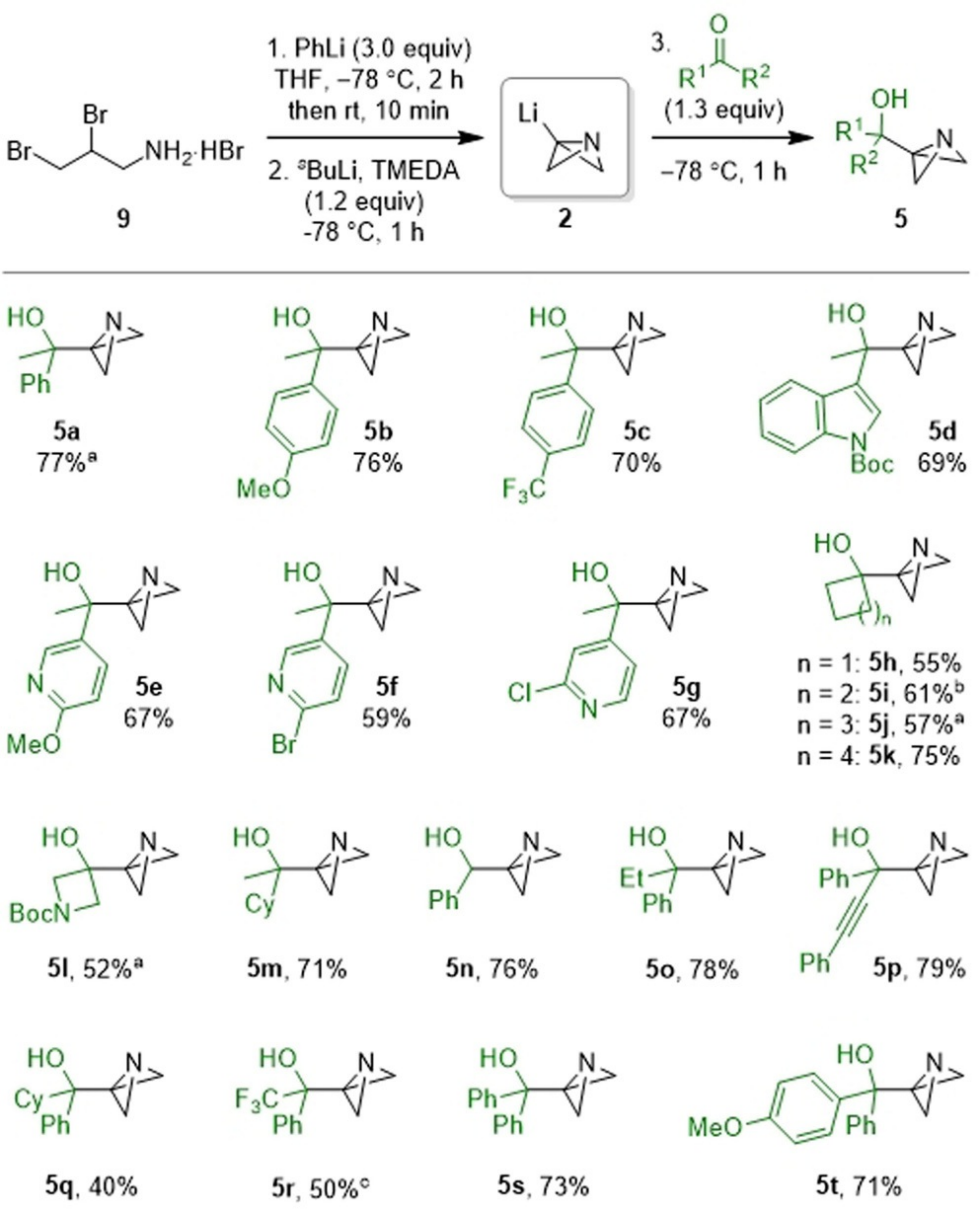
Synthesis of ABB‐carbinols by the reaction of ABB‐Li with carbonyl compounds. Reactions performed on 2.0 mmol scale at 0.31 M initial concentration. Yields determined by ^1^H NMR. [a] 4.0 mmol scale. [b] 1.0 mmol scale. [c] Purified by flash column chromatography.

We began our investigation by studying the reaction of ABB‐Li with acetophenone (Scheme 2).^[14]^ ABB‐Li was formed in situ by the sequential reaction of amine salt **9** with phenyllithium and *sec*‐butyllithium,^[8a]^ and subsequently reacted with acetophenone at −78 °C to form ABB‐carbinol **5 a** in good yield. This procedure was successfully applied to a wide range of ketones and aldehydes. The alcohol products were stable to aqueous work‐up and could be stored at −18 °C with no evidence of degradation. However, apart from the trifluoromethylated example **5 r**, the ABB‐carbinols were found to partially decompose on silica gel and so yields of the products were determined by ^1^H NMR. Presumably, the electron‐withdrawing nature of the CF_3_ group present in **5 r** reduces the basicity of the nitrogen and minimizes acid‐mediated decomposition.

**Scheme 3:**
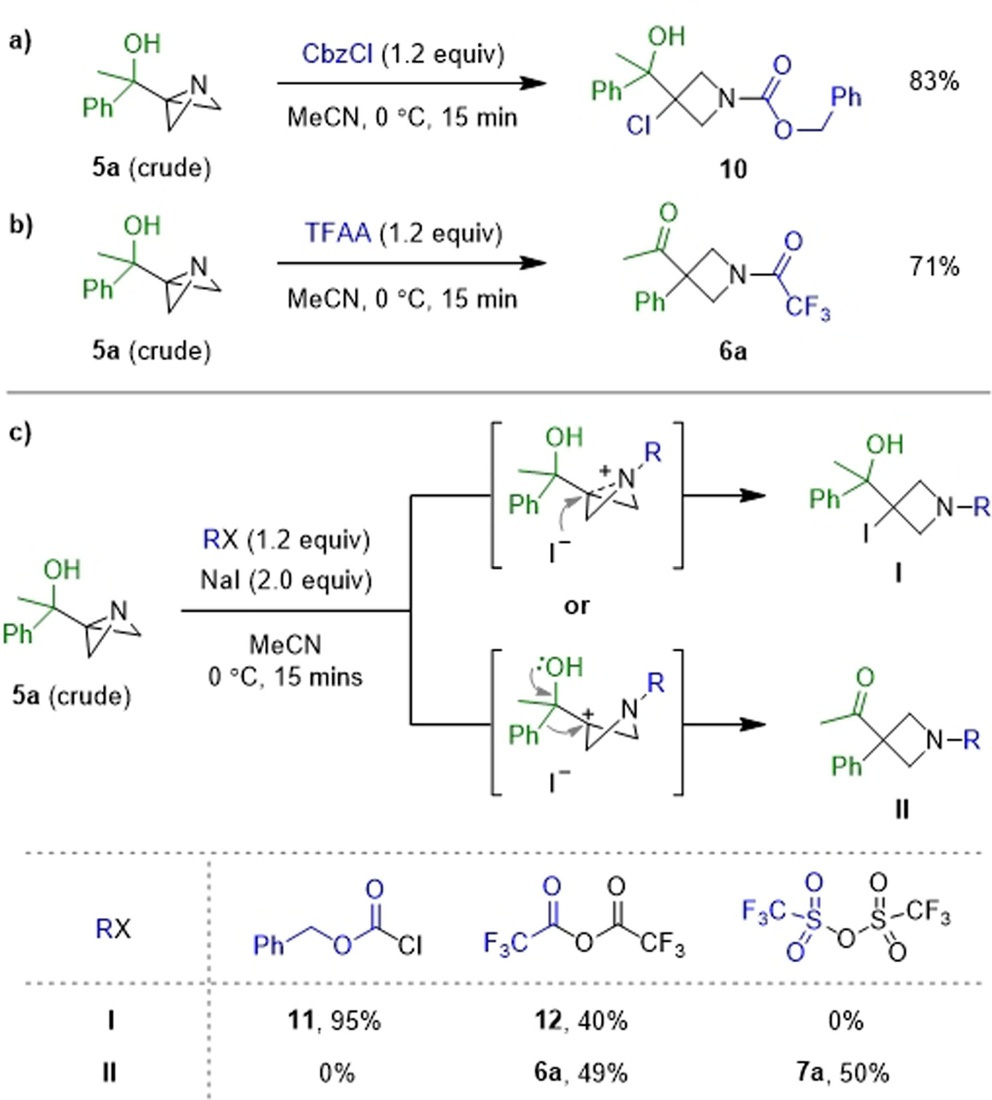
Divergent reactivity of **5 a** with a) CbzCl vs. b) TFAA. c) Investigations into the cause of divergence.

Having gained access to a range of ABB‐carbinols, their reactivity with different activating agents was investigated. Firstly, the addition of benzyl chloroformate (CbzCl) to **5 a** resulted in the formation of chlorohydrin **10** in 83 % yield (Scheme 3 a). This result is consistent with previous reports of the 1,3‐functionalisation of ABB with chloroformates.^[15]^ However, when trifluoroacetic anhydride (TFAA) was employed as the activating agent, the desired semipinacol rearrangement occurred to form keto azetidine **6 a** in 71 % yield (Scheme 3 b).

The divergent reactivity of **5 a** can be rationalised by the extent of positive charge build‐up at the electrophilic bridgehead carbon. When TFAA is used as an activator, the trifluoroacetyl group, being more electron‐withdrawing than the Cbz group, results in a greater build‐up of positive charge, thereby favouring the semipinacol reaction pathway. We postulated that the different counterions could also play a role in determining the outcome of the reaction, therefore, we investigated whether product formation was determined by the nucleophilicity of the counterion or by the nature of the activating group. This was achieved by performing the reactions in the presence of NaI in order to keep the counterion (iodide) constant (Scheme 3 c).^[16]^ Once again, with CbzCl, nucleophilic addition dominated to give exclusively iodohydrin **11** via the nucleophilic addition pathway. With TFAA, both nucleophilic addition and semipinacol rearrangement occurred to give **12** and **6 a** in 40 % and 49 % yield, respectively. Finally, when moving to an even more electrophilic activating agent, triflic anhydride (Tf_2_O), the semipinacol rearrangement dominated to give sulfonamide **7 a** in 50 % yield with no nucleophilic addition observed. Thus, the outcome of the reaction is predominantly determined by the electronic nature of the activating group on nitrogen: the more electron‐withdrawing it is the more the semipinacol pathway is favored.

**Scheme 4:**
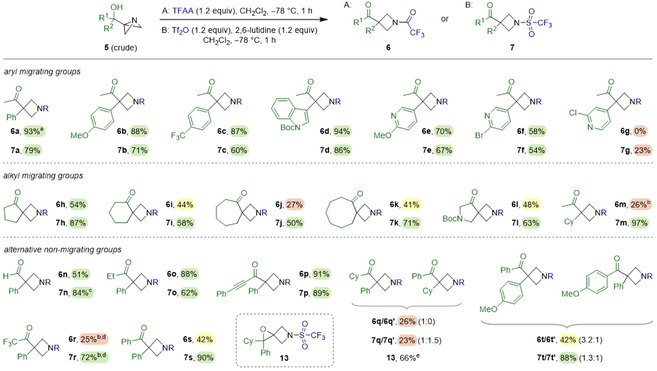
Scope of ABB‐carbinols employed in the semipinacol rearrangement reaction. Reactions performed on 0.25 mmol scale at 0.1 M. [a] Reaction also performed on 1.0 mmol scale to give **6 a** in 90 % yield. [b] Reactions stirred for 15 min at −78 °C then 30 min at 0 °C. [c] Product isolated after reduction by NaBH_4_ to the primary alcohol. [d] From purified **5 r**. [e] Formed under conditions B and yield determined by ^1^H NMR.

We sought to optimise the semipinacol pathway and found that, in the absence of NaI, the reaction yields with TFAA and Tf_2_O were improved by switching the solvent to CH_2_Cl_2_ and performing the reaction at −78 °C (conditions A and B; see Supporting Information for optimisation study). In the case of Tf_2_O, the yield was further increased by the addition of 2,6‐lutidine. We then explored the scope of the reaction using both sets of conditions (A and B) with a broad range of ABB‐carbinols (Scheme 4).

For non‐symmetrical alcohols bearing an aryl and methyl group, in all cases, and under both sets of conditions, we found exclusive migration of the aryl group to give azetidines **6**/**7 a**–**c** in good yields, even in the cases of electron‐poor aryl groups. Furthermore, electron‐rich and electron‐deficient heteroaromatic groups (indolyl and 3‐pyridyl) also worked well, giving compounds **6**/**7 d**–**f** in good yields—again exclusive migration of the aryl group was observed. However, 4‐pyridyl carbinol **5 g** was found to be a poor substrate, as the reaction was unsuccessful with TFAA and low yielding with Tf_2_O.

Cyclic ketones were also explored as the semipinacol rearrangement would result in a ring expansion to give valuable spirocyclic azetidine scaffolds. Azetidines **6 h**–**k** and **7 h**–**k** were obtained after ring expansions from ABB‐carbinols bearing 4‐, 5‐, 6‐, and 7‐membered rings in yields of 27–54 % using TFAA (method A) and 50–87 % using Tf_2_O (method B). The lower yields when using TFAA were attributed to competing nucleophilic addition of trifluoroacetate due to the lower migratory aptitude of alkyl groups.^[17]^ The semipinacol rearrangement of ABB‐carbinol **5 l** gave azetidines **6**/**7 l** with an intriguing 2,6‐diazaspiro[3.4]octane core in 48 % and 63 % yields, respectively.^[18]^ This example is particularly noteworthy as a Reaxys search identified this motif in >250 patents with >1300 unique examples where pharmacological data is presented. Furthermore, it is easily prepared in just two steps from N‐Boc azetidinone. In order to further probe the selectivity over which group migrates, a non‐symmetrical alcohol bearing two different alkyl groups (a cyclohexyl and a methyl group) was explored. Using both TFAA (method A) and Tf_2_O (method B), exclusive migration of the more substituted alkyl group^[11b]^ was observed, giving **6**/**7 m**, the latter in almost quantitative yield.

We subsequently compared the migration of a Ph group over other substituents, including more substituted alkyl groups and other functional groups. Comparing Ph with H, we found that the Ph group migrated exclusively to give aldehydes **6**/**7 n** in 51 % and 84 % yields, respectively.^[9]^ Comparing Ph/ethyl and Ph/alkynyl, again resulted in exclusive migration of the Ph group, giving azetidines **6**/**7 o**,**p** in excellent yields. However, comparing Ph/Cy resulted in high selectivity but only in the case of TFAA to give **6 q**; Tf_2_O gave a 1:1.5 ratio of products **7 q**/**7 q′**, now in favour of Cy migration. In both cases, the yield of the ketone was low. In fact, the main component of the latter reaction was spirocyclic epoxide **13**, formed in 66 %. Trifluoromethyl carbinol **5 r** was tested and as expected,^[19]^ exclusive migration of the Ph group was observed. This substrate was substantially less reactive than the others, requiring 0 °C to trigger the semipinacol rearrangement. Azetidine **6 r** was formed in only 25 % yield with competing nucleophilic addition of trifluoroacetate observed. Selectivity for the semipinacol rearrangement was much higher with Tf_2_O (conditions B), giving azetidine **7 r** in 72 % yield.

Azetidines **6 s** and **7 s** were obtained from ABB‐carbinol **5 s** derived from benzophenone in 42 % and 90 % yields under conditions A and B, respectively. In the case of **6 s**, the lower yield observed was due to competing nucleophilic addition. Finally, comparing Ph/p‐MeOC_6_H_4_, we found that the more electron‐rich aryl group migrated preferentially,^[20]^ but not by much: a 3.2:1 ratio of **6 t**/**6 t′** and 1.3:1 ratio of **7 t**/**7 t′** were obtained under conditions A and B, respectively.

The commonly observed relative migratory aptitude in semipinacol rearrangements[^9^, ^17^, ^20^] of aryl > alkenyl > hydride > substituted alkyl > less substituted alkyl is mirrored here in reactions using TFAA. A similar pattern is seen with the more electron‐withdrawing Tf_2_O, but the selectivity is lower since it induces a faster reaction. In the case of the especially hindered substrate **7 q**, containing both phenyl and cyclohexyl migrating groups, no selectivity is observed. Here, C−C bond rotation of the hindered substrate is likely to have a higher barrier than 1,2‐migration,^[21]^ which results in migration of whichever group is antiperiplanar to the central C−N upon reaction with Tf_2_O.

**Scheme 5:**
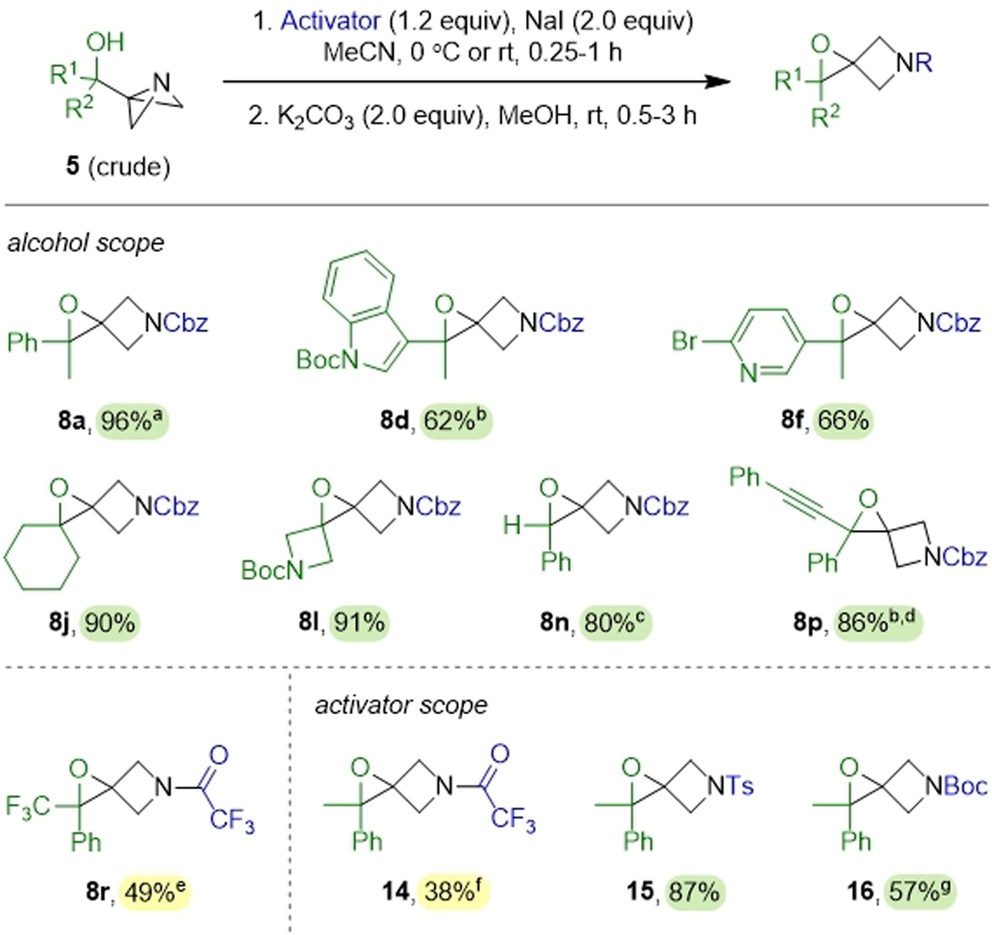
Scope of ABB‐carbinols employed in the epoxide formation reaction. Reactions performed on 0.25 mmol scale and 0.1 M initial concentration. [a] Reaction also performed on 1.0 mmol scale to give **8 a** in 93 % yield. [b] Intermediate iodohydrin purified before second step. [c] Stirred for 42 h after addition of K_2_CO_3_. [d] 0.05 M initial concentration. [e] From purified **5 r**. [f] Semipinacol reaction also occurred in 36 % yield. [g] After addition of Boc_2_O, heated at 80 °C for 18 h without K_2_CO_3_.

The formation of spirocyclic epoxide **13** from **5 q** under conditions B was intriguing, and we were keen to establish whether this pathway could be promoted more generally. We reasoned that iodohydrin **11**, formed from the reaction of **5 a** with CbzCl and NaI (Scheme 3 c), could potentially serve as an intermediate in the selective synthesis of spiroepoxy azetidines. Indeed, addition of potassium carbonate to a solution of **11** in methanol resulted in the quantitative formation of epoxy azetidine **8 a** after 15 minutes.^[22]^ This method was telescoped to a one‐pot procedure, and ABB‐carbinol **5 a** was converted to **8 a** in 96 % yield (Scheme 5).

We then extended this protocol to produce a range of spirocyclic epoxides from selected ABB‐carbinols (Scheme 5). Employing ABB‐carbinols with heteroaryl groups (indolyl and pyridyl) gave **8 d** and **8 f** in good yields of 62 % and 66 %, respectively. Electron‐rich indolyl epoxide **8 d** was found to be unstable on silica gel, which complicated purification. However, we found that if the intermediate iodohydrin was purified prior to base‐induced cyclization, no further purification of **8 d** was necessary. Dispiro compounds **8 j** and **8 l** were obtained from ABB‐carbinols **5 j** and **5 l** in excellent yields of 90 % and 91 %, respectively. Compound **8 l** is particularly interesting, as the nitrogen protecting groups are orthogonal and serve to desymmetrize the molecule. Trisubstituted epoxide **8 n** was also accessible in 80 % yield from benzaldehyde derived **5 n**. We found that with less substitution, the rate of cyclization was much slower and required 42 hours to reach completion. We were also able to synthesize the sensitive propargylic epoxide **8 p** in excellent yield (86 %). Due to its instability on silica gel (as with **8 d**), purification of the intermediate iodohydrin was necessary in order to isolate analytically pure **8 p**.

In the case of trifluoromethyl ABB‐carbinol **5 r**, CbzCl was found to be unreactive, but treatment with TFAA in the presence of NaI led to selective formation of the iodohydrin intermediate. After the addition of potassium carbonate, epoxide **8 r** was isolated in 49 % yield. The high selectivity for iodohydrin formation from **5 r** contrasts with the reactivity of **5 a**, where treatment with TFAA in the presence of NaI resulted in the formation of both iodohydrin **12** and the semipinacol product **6 a**. This is a result of the slower rate of the semipinacol rearrangement with the trifluoromethyl ABB‐carbinol **5 r**. Despite the reaction of **5 a** with TFAA/NaI leading to a mixture of **12** and **6 a**, treating this mixture with potassium carbonate led to the formation of epoxide **14** in 38 % yield. Interestingly, other activators could also be employed with NaI. Tosyl chloride (TsCl) behaved similarly to CbzCl to give sulfonamide **15** in 87 % yield, whereas di‐*tert*‐butyl dicarbonate (Boc_2_O) was slower to react and required heating to activate ABB‐carbinol **5 a**. Under these conditions with Boc_2_O, cyclization of the iodohydrin occurred without additional base to give epoxide **16** in 57 % yield.

## Conclusion

We have discovered novel, divergent reactivity of azabicyclo[1.1.0]butyl carbinols, which are themselves easily obtained from the reaction of azabicyclo[1.1.0]butyllithium with carbonyl compounds. We found that strongly electrophilic activating reagents (TFAA and Tf_2_O) induce a semipinacol rearrangement in ABB‐carbinols to give either amide or sulfonamide azetidines. The semipinacol rearrangement proceeds with migration of the group best able to stabilize the positive charge, so follows the order aryl > substituted alkyl > less substituted alkyl. Even electron‐deficient aromatics and heteroaromatics migrate in preference to a methyl group. When two alkyl groups are present, the semipinacol rearrangement is much slower and in the case of TFAA as the activator, nucleophilic addition of the counterion begins to compete, leading to lower yields. However, switching to Tf_2_O as the activator allows the semipinacol rearrangement to dominate, leading to good reaction yields.

Conversely, when ABB‐carbinols are treated with less electrophilic activating agents, such as CbzCl, no semipinacol rearrangement occurs. Instead, nucleophilic addition of the counterion dominates to exclusively form chlorohydrin products. Performing the reactions in the presence of NaI leads to the formation of iodohydrins that can be easily converted into structurally interesting spiroepoxy azetidines through a base‐mediated cyclization. Thus, from a common ABB‐carbinol starting material, we can now access either keto azetidines or spiroepoxy azetidines through semipinacol rearrangements and spirocyclizations, respectively.

## Conflict of interest

The authors declare no conflict of interest.

## Supporting information

As a service to our authors and readers, this journal provides supporting information supplied by the authors. Such materials are peer reviewed and may be re‐organized for online delivery, but are not copy‐edited or typeset. Technical support issues arising from supporting information (other than missing files) should be addressed to the authors.

Supplementary
