# Supplementary material for: Divergent, Strain‐Release Reactions of Azabicyclo[1.1.0]butyl Carbinols: Semipinacol or Spiroepoxy Azetidine Formation
Source: Angew Chem Int Ed Engl. 2021 Feb 26;60(13):7360–5. doi: 10.1002/anie.202100583 (PMC8247891; doi:10.1002/anie.202100583)

Angewandte Chemie
Eine Zeitschrift der Gesellschaft Deutscher Chemiker

Supporting Information

Divergent, Strain-Release Reactions of Azabicyclo[1.1.0]butyl Carbinols: Semipinacol or Spiroepoxy Azetidine Formation

*Charlotte H. U. Gregson, Adam Noble, and Varinder K. Aggarwal**

anie_202100583_sm_miscellaneous_information.pdf

Contents

1. Experimental 1
   1.1. General experimental 1
      1.1.1. Solvents, reagents and glassware 1
      1.1.2. Chromatography and spectroscopy 2
   1.2. General procedures 2
      1.2.1. General procedure 1 2
      1.2.2. General procedure 2a 3
      1.2.3. General procedure 2b 4
      1.2.4. General procedure 3a 4
      1.2.5. General procedure 3b 5
   1.3. Synthesis of azabicyclo[1.1.0]butyl carbinols 6
   1.4. Initial reaction investigations 11
   1.5. Optimisation of the semipinacol rearrangement reaction 14
   1.6. Scope of the semipinacol rearrangement reaction 15
   1.7. Scope of the epoxide formation reaction 38
2. NMR Spectra 47

1. Experimental

1.1. General experimental

1.1.1. Solvents, reagents and glassware

All solvents were commercially supplied or dried on an Anhydrous Engineering alumina column drying system. All reactions were conducted under an inert atmosphere of nitrogen using standard Schlenk techniques unless stated otherwise. All glassware was oven dried prior to use. All reagents were purchased from commercial sources and used as sold. Exceptions: all

liquid ketone/aldehyde reagents were filtered through a plug of neutral alumina immediately prior to use; benzyl chloroformate was distilled under an inert atmosphere at standard pressure and then stored over sodium carbonate; trifluoroacetic anhydride (TFAA) was distilled over phosphorous(V) pentoxide under an inert atmosphere at standard pressure; tetramethylethylenediamine (TMEDA) was distilled over calcium hydride under an inert atmosphere at standard pressure; organolithium reagents were regularly titrated.

1.1.2. Chromatography and spectroscopy

All reactions were followed by thin-layer chromatography (TLC) when practical, which were visualised under UV light or by staining with aqueous potassium permanganate. ^{1}H, ^{13}C and ^{19}F NMR spectra were recorded using Jeol ECS 400 MHz, Jeol ECz 400 MHz, Bruker 400 MHz and Bruker Avance III HD 500 MHz Cryo spectrometers. Chemical shifts (δ) are given in parts per million (ppm) and coupling constants (J) are given in Hertz (Hz), rounded to the nearest 0.1 Hz. NMR assignments were made according to spin systems, using two-dimensional NMR spectroscopy (COSY, HSQC, HMBC) to assist the assignment. NMR yields were determined using dibromomethane as an internal standard. High resolution mass spectra (HRMS) were recorded by Bruker Daltonics Apex IV by Electrospray Ionisation (ESI) or Atmospheric pressure chemical ionization (APCI). IR spectra were recorded on a Perkin Elmer Spectrum One FT-IR as a thin film. Absorption maxima (ν_{max}) are reported in wavenumbers (cm^{-1}).

1.2. General procedures

1.2.1. General procedure 1

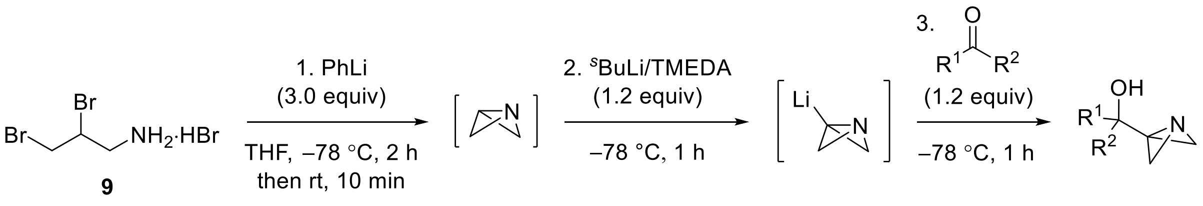

In a 100 mL Schlenk flask, phenyl lithium (in Bu_2O, 6.0 mmol, 3.0 equiv) was added dropwise (at a rate of 0.11 mL/min) to a suspension of salt **9**[A] (596 mg, 2.0 mmol, 1.0 equiv) in anhydrous tetrahydrofuran (6.4 mL) at –78 °C (dry ice/acetone). After complete addition, the resulting solution was stirred for a further 2 hours at –78 °C. After this time, the reaction mixture was removed from the cooling bath and warmed to room temperature over 10 minutes with stirring

(the reaction mixture should be homogeneous once at room temperature). After cooling back down to –78 °C, TMEDA (0.36 mL, 2.4 mmol, 1.2 equiv) was then added followed by *sec*-butyl lithium (in cyclohexane, 2.4 mmol, 1.2 equiv) dropwise (at a rate of 0.11 mL/min), and the resulting solution stirred for 1 hour at –78 °C. After this time, the ketone/aldehyde (0.26 mmol, 1.3 equiv) was added dropwise, either neat or as a solution in anhydrous tetrahydrofuran. The reaction was stirred for a further 1 hour at –78 °C. Diethyl ether or ethyl acetate (30 mL) and water (20 mL) were then added, and the reaction mixture transferred to a separating funnel. The phases were separated, and the aqueous phase was extracted with diethyl ether or ethyl acetate (3 × 30 mL) and the combined organic phases were washed with brine, dried ($MgSO_4$), filtered and concentrated under reduced pressure.

The crude NMR yield of the product was then determined using the following procedure. The crude mixture was dissolved in deuterated chloroform and dibromomethane (42 μL, 0.6 mmol, 0.3 equiv) was added as an internal standard. After the yield was determined by quantitative ^{1}H NMR, the crude product was transferred to a volumetric flask which was filled to the mark with dichloromethane and the solution concentration was then calculated. The volume of solution of azabicyclo[1.1.0]butyl carbinol corresponding to 0.25 mmol was removed by syringe and transferred to a round bottom flask then evaporated to dryness to be used in subsequent reactions. The crude azabicyclo[1.1.0]butyl carbinols can be stored for extended periods (months) in a freezer under inert atmosphere without degradation.

Notes: (**A**) Salt **9** was synthesised according to a reported procedure: R. Gianatassio, J. M. Lopchuk, J. Wang, C.-M. Pan, L. R. Malins, L. Prieto, T. A. Brandt, M. R. Collins, G. M. Gallego, N. W. Sach, J. E. Spangler, H. Zhu and P. S. Baran, *Science* **2016**, *351*, 241−246.

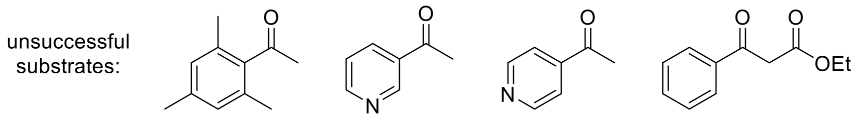

1.2.2. General procedure 2a

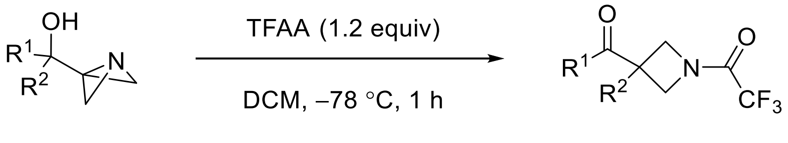

To a round bottom flask containing the crude azabicyclo[1.1.0]butyl carbinol (0.25 mmol, 1.0 equiv) under an inert atmosphere was added anhydrous dichloromethane (2.5 mL). The

flask was cooled to –78 °C and trifluoroacetic anhydride (0.04 mL, 0.3 mmol, 1.2 equiv) was added with stirring. After 1 hour at –78 °C, diethyl ether or ethyl acetate (10 mL) and saturated aqueous sodium bicarbonate solution (10 mL) were added and the reaction mixture transferred to a separating funnel. The phases were separated, and the aqueous phase was extracted with diethyl ether or ethyl acetate (3 × 20 mL) and the combined organic phases were washed with brine, dried ($MgSO_4$), filtered and concentrated under reduced pressure.

1.2.3. General procedure 2b

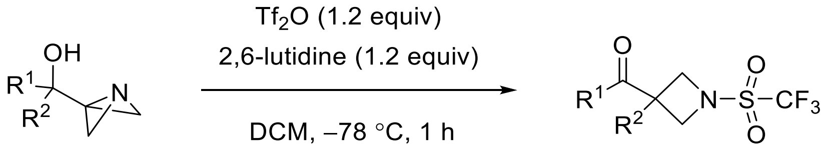

To a round bottom flask containing the crude azabicyclo[1.1.0]butyl carbinol (0.25 mmol, 1.0 equiv) under an inert atmosphere was added anhydrous dichloromethane (2.5 mL). The flask was cooled to –78 °C and 2,6-lutidine (34 μL, 0.3 mmol, 1.2 equiv) followed by trifluoromethanesulfonic anhydride (0.05 mL, 0.3 mmol, 1.2 equiv) was added with stirring. After 1 hour at –78 °C, diethyl ether or ethyl acetate (10 mL) and saturated aqueous sodium bicarbonate solution (10 mL) were added and the reaction mixture transferred to a separating funnel. The phases were separated, and the aqueous phase was extracted with diethyl ether or ethyl acetate (3 × 20 mL) and the combined organic phases were washed with brine, dried ($MgSO_4$), filtered and concentrated under reduced pressure.

1.2.4. General procedure 3a

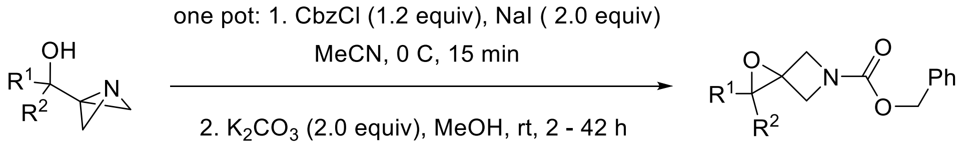

To a round bottom flask containing a mixture of the crude azabicyclo[1.1.0]butyl carbinol (0.25 mmol, 1.0 equiv) and sodium iodide (75 mg, 0.5 mmol, 2.0 equiv) under an inert atmosphere was added anhydrous acetonitrile (2.5 mL). The flask was cooled to 0 °C and benzyl chloroformate (0.04 mL, 0.3 mmol, 1.2 equiv) was added with stirring. After 15 minutes at 0 °C, potassium carbonate (69 mg, 0.5 mmol, 2.0 equiv) was added followed by anhydrous methanol (2.5 mL) and the reaction was warmed to room temperature. The progress of the reaction was monitored by TLC and usually found to be complete within 3 hours at room

temperature. After the reaction was complete, diethyl ether or ethyl acetate (10 mL) and saturated aqueous sodium bicarbonate solution (10 mL) were added, and the reaction mixture transferred to a separating funnel. The phases were separated, and the aqueous phase was extracted with diethyl ether or ethyl acetate (3 × 20 mL) and the combined organic phases were washed with brine, dried ($MgSO_4$), filtered and concentrated under reduced pressure.

1.2.5. General procedure 3b

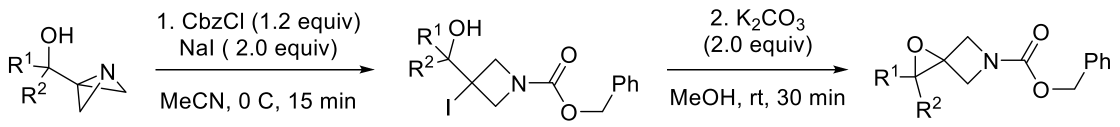

To a round bottom flask containing a mixture of the crude azabicyclo[1.1.0]butyl carbinol (0.25 mmol, 1.0 equiv) and sodium iodide (75 mg, 0.5 mmol, 2.0 equiv) under an inert atmosphere was added anhydrous acetonitrile (2.5 mL). The flask was cooled to 0 °C and benzyl chloroformate (0.04 mL, 0.3 mmol, 1.2 equiv) was added with stirring. After 15 minutes at 0 °C, saturated aqueous sodium bicarbonate solution (10 mL) and diethyl ether or ethyl acetate (10 mL) were added, and the reaction mixture transferred to a separating funnel. The phases were separated, and the aqueous phase was extracted with diethyl ether or ethyl acetate (3 × 20 mL) and the combined organic phases were washed with brine, dried ($MgSO_4$), filtered and concentrated under reduced pressure.

After isolation and characterisation, the iodohydrin was transferred to a round bottom flask and potassium carbonate (69 mg, 0.5 mmol, 2.0 equiv) was added. Under and inert atmosphere, anhydrous methanol (2.5 mL) was added, and the reaction was stirred at room temperature for 30 minutes. Saturated aqueous sodium bicarbonate solution (10 mL) and diethyl ether or ethyl acetate (10 mL) were added, and the reaction mixture transferred to a separating funnel. The phases were separated, and the aqueous phase was extracted with diethyl ether or ethyl acetate (3 × 20 mL) and the combined organic phases were washed with brine, dried ($MgSO_4$), filtered and concentrated under reduced pressure.

1.3. Synthesis of azabicyclo[1.1.0]butyl carbinols

1-(1-Azabicyclo[1.1.0]butan-3-yl)-1-phenylethan-1-ol, 5a

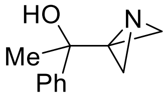

Prepared using general procedure 1 on a 4.0 mmol scale. Acetophenone (0.60 mL, 5.2 mmol, 1.3 equiv) was added neat to the reaction. ^{1}H NMR yield: 80%. See spectrum

HRMS (ESI): Calcd. for $C_{11}H_{13}NONa^+$ ($[M+Na]^+$) *m/z* 198.0889, found *m/z* 198.0889.

1-(1-Azabicyclo[1.1.0]butan-3-yl)-1-(4-methoxyphenyl)ethan-1-ol, 5b

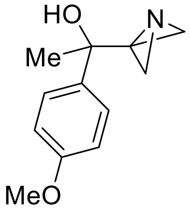

Prepared using general procedure 1. 4′-Methoxyacetophenone (0.390 g, 2.6 mmol, 1.3 equiv) was weighed into a vial which was evacuated and backfilled with nitrogen. Anhydrous tetrahydrofuran (1.0 mL) was added and this solution was added dropwise to the reaction and a further portion of anhydrous tetrahydrofuran (0.5 mL) was used to wash the residual reagent from the vial into the reaction mixture. ^{1}H NMR yield: 70%. See spectrum

HRMS (ESI): Calcd. for $C_{12}H_{15}NO_2Na^+$ ($[M+Na]^+$) *m/z* 228.0995, found *m/z* 228.1006.

1-(1-Azabicyclo[1.1.0]butan-3-yl)-1-(4-(trifluoromethyl)phenyl)ethan-1-ol, 5c

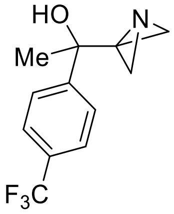

Prepared using general procedure 1. 4′-(Trifluoromethyl)acetophenone (0.490 g, 2.6 mmol, 1.3 equiv) was weighed into a vial which was evacuated and backfilled with nitrogen. Anhydrous tetrahydrofuran (1.0 mL) was added and this solution was added dropwise to the reaction and a further portion of anhydrous tetrahydrofuran (0.5 mL) was used to wash the residual reagent from the vial into the reaction mixture. ^{1}H NMR yield: 70%. See spectrum

HRMS (ESI): Calcd. for $C_{12}H_{13}F_3NO^+$ ($[M+Na]^+$) *m/z* 244.0944, found *m/z* 244.0944.

(1- *tert*-Butyl 3-(1-(1-azabicyclo[1.1.0]butan-3-yl)-1-hydroxyethyl)-1H-indole-1-carboxylate, 5d

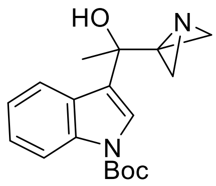

Prepared using general procedure 1. *tert*-Butyl 3-acetyl-1H-indole-1-carboxylate[A] (674 mg, 2.6 mmol, 1.3 equiv) was weighed into a vial which was evacuated and backfilled with nitrogen. Anhydrous tetrahydrofuran (5 mL) was added and this solution was added dropwise to the reaction mixture over 3 minutes. ^{1}H NMR yield: 69%. See spectrum

HRMS (ESI): Calcd. for $C_{18}H_{22}N_2O_3Na^+$ ($[M+Na]^+$) *m/z* 337.1523, found *m/z* 337.1525.

Notes: (**A**) Prepared according to a literature procedure: L. Chen, J.-J. Shen, Q. Gao, S. Xu, *Chem. Sci.* **2018**, *9*, 5855–5859.

1-(1-Azabicyclo[1.1.0]butan-3-yl)-1-(6-methoxypyridin-3-yl)ethan-1-ol, 5e

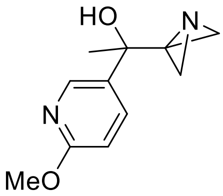

Prepared using general procedure 1. 1-(6-methoxypyridin-3-yl)ethan-1-one (393 mg, 2.6 mmol, 1.3 equiv) was weighed into a vial which was evacuated and backfilled with nitrogen. Anhydrous tetrahydrofuran (1.5 mL) was added and this solution was added dropwise to the reaction mixture. ^{1}H NMR yield: 66%. See spectrum

HRMS (ESI): Calcd. for $C_{11}H_{15}N_2O_2^+$ ($[M+H]^+$) *m/z* 207.1128, found *m/z* 207.1132.

1-(1-Azabicyclo[1.1.0]butan-3-yl)-1-(6-bromopyridin-3-yl)ethan-1-ol, 5f

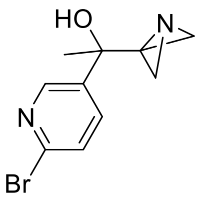

Prepared using general procedure 1. 1-(6-Bromopyridin-3-yl)ethan-1-one (520 mg, 2.6 mmol, 1.3 equiv) was weighed into a vial which was evacuated and backfilled with nitrogen. Anhydrous tetrahydrofuran (5 mL) was added and this solution was added dropwise to the reaction mixture over 3 minutes. ^{1}H NMR yield: 59%. See spectrum

HRMS (ESI): Calcd. for $C_{10}H_{11}BrN_2ONa^+$ ($[M+Na]^+$) *m/z* 276.9947, found *m/z* 276.9954.

1-(1-Azabicyclo[1.1.0]butan-3-yl)-1-(2-chloropyridin-4-yl)ethan-1-ol, 5g

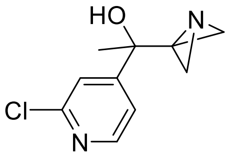

Prepared using general procedure 1. 1-(2-Chloropyridin-4-yl)ethan-1-one (405 mg, 2.6 mmol, 1.3 equiv) was weighed into a vial which was evacuated and backfilled with nitrogen. Anhydrous tetrahydrofuran (1.5 mL) was added and this solution was added dropwise to the reaction mixture over 3 minutes. ^{1}H NMR yield: 62%. See spectrum

HRMS (ESI): Calcd. for $C_{10}H_{12}ClN_2O^+$ ($[M+H]^+$) *m/z* 211.0633, found *m/z* 211.0636.

1-(1-Azabicyclo[1.1.0]butan-3-yl)cyclobutan-1-ol, 5h

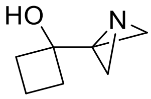

Prepared using general procedure 1. Cyclobutanone (0.19 mL, 2.6 mmol, 1.3 equiv) was added neat to the reaction dropwise over 10 minutes. ^{1}H NMR yield: 55%. See spectrum

HRMS (ESI): Calcd. for $C_7H_{12}NO^+$ ($[M+H]^+$) *m/z* 126.0913, found *m/z* 126.0918.

1-(1-Azabicyclo[1.1.0]butan-3-yl)cyclopentan-1-ol, 5i

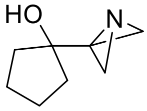

Prepared using general procedure 1 on a 1.0 mmol scale. Cyclopentanone (0.11 mL, 1.3 mmol, 1.3 equiv) was added neat to the reaction. ^{1}H NMR yield: 61%. See spectrum

HRMS (APCI): Calcd. for $C_8H_{13}NO^+$ ($[M+Na]^+$) *m/z* 140.1070, found *m/z* 140.1071.

1-(1-Azabicyclo[1.1.0]butan-3-yl)cyclohexan-1-ol, 5j

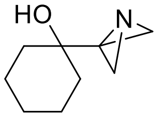

Prepared using general procedure 1 on a 4.0 mmol scale. Cyclohexanone (0.62 mL, 5.2 mmol, 1.3 equiv) was added neat to the reaction. ^{1}H NMR yield: 88%. See spectrum

HRMS (ESI): Calcd. for $C_9H_{15}NONa^+$ ($[M+Na]^+$) *m/z* 176.1046, found *m/z* 176.1043.

1-(1-Azabicyclo[1.1.0]butan-3-yl)cycloheptan-1-ol, 5k

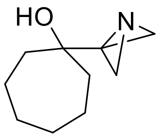

Prepared using general procedure 1. Cycloheptanone (0.31 mL, 2.6 mmol, 1.3 equiv) was added neat to the reaction. ^{1}H NMR yield: 75%. See spectrum

HRMS (APCI): Calcd. for $C_{10}H_{17}NO^{+}$ ($[M+H]^{+}$) *m*/*z* 168.1383, found *m*/*z* 168.1385.

***tert*-Butyl 3-(1-azabicyclo[1.1.0]butan-3-yl)-3-hydroxyazetidine-1-carboxylate, 5l**

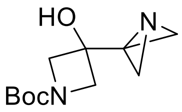

Prepared using general procedure 1 on a 4 mmol scale. *tert*-Butyl 3-oxoazetidine-1-carboxylate (0.890 g, 5.2 mmol, 1.3 equiv) was weighed into a vial which was evacuated and backfilled with nitrogen. Anhydrous tetrahydrofuran (3.0 mL) was added and this solution was added dropwise to the reaction mixture slowly over 10 minutes. ^{1}H NMR yield: 51%. See spectrum

HRMS (ESI): Calcd. for $C_{11}H_{18}N_2O_3Na^{+}$ ($[M+Na]^{+}$) *m*/*z* 249.1210, found *m*/*z* 249.1200.

1-(1-Azabicyclo[1.1.0]butan-3-yl)-1-cyclohexylethan-1-ol, 5m

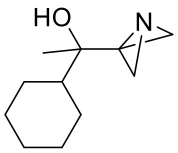

Prepared using general procedure 1. 1-Cyclohexylethan-1-one (0.36 mL, 2.6 mmol, 1.3 equiv) was added neat to the reaction. ^{1}H NMR yield: 71%. See spectrum

HRMS (ESI): Calcd. for $C_{11}H_{19}NONa^{+}$ ($[M+Na]^{+}$) *m*/*z* 204.1359, found *m*/*z* 204.1353.

(1-Azabicyclo[1.1.0]butan-3-yl)(phenyl)methanol, 5n

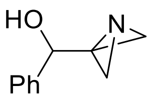

Prepared using general procedure 1. Benzaldehyde (0.26 mL, 2.6 mmol, 1.3 equiv) was added neat to the reaction. ^{1}H NMR yield: 71%. See spectrum

HRMS (ESI): Calcd. for $C_{10}H_{11}NONa^{+}$ ($[M+Na]^{+}$) *m*/*z* 184.0733, found *m*/*z* 184.0736.

1-(1-Azabicyclo[1.1.0]butan-3-yl)-1-phenylpropan-1-ol, 5o

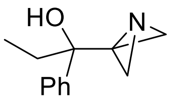

Prepared using general procedure 1. Propiophenone (0.30 mL, 2.6 mmol, 1.3 equiv) was added neat to the reaction. ^{1}H NMR yield: 78%. See spectrum

HRMS (APCI): Calcd. for $C_{12}H_{15}NO^{+}$ ($[M+H]^{+}$) *m*/*z* 190.1226, found *m*/*z* 190.1227.

1-(1-Azabicyclo[1.1.0]butan-3-yl)-1,3-diphenylprop-2-yn-1-ol, 5p

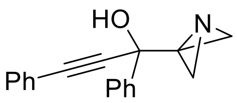

Prepared using general procedure 1. 1,3-Diphenylprop-2-yn-1-one (536 mg, 2.6 mmol, 1.3 equiv) was weighed into a vial which was evacuated and backfilled with nitrogen. Anhydrous tetrahydrofuran (1.5 mL) was added and this solution was added dropwise to the reaction mixture. ^{1}H NMR yield: 79%. See spectrum

HRMS (ESI): Calcd. for $C_{18}H_{15}NONa^{+}$ ([M+Na]$^{+}$) *m*/*z* 284.1046, found *m*/*z* 284.1043.

(1-Azabicyclo[1.1.0]butan-3-yl)(cyclohexyl)(phenyl)methanol, 5q

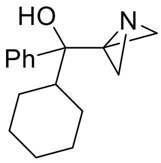

Prepared using general procedure 1. Cyclohexyl(phenyl)methanone (308 mg, 2.6 mmol, 1.3 equiv) was weighed into a vial which was evacuated and backfilled with nitrogen. Anhydrous tetrahydrofuran (1.5 mL) was added and this solution was added dropwise to the reaction mixture. ^{1}H NMR yield: 40%. See spectrum

HRMS (APCI): Calcd. for $C_{16}H_{21}NO^{+}$ ([M+Na]$^{+}$) *m*/*z* 244.1696, found *m*/*z* 244.1696.

1-(1-Azabicyclo[1.1.0]butan-3-yl)-2,2,2-trifluoro-1-phenylethan-1-ol, 5r

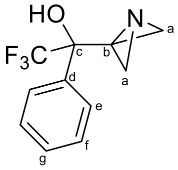

Prepared using general procedure 1. 2,2,2-Trifluoro-1-phenylethan-1-one (0.37 mL, 2.6 mmol, 1.3 equiv) was added neat to the reaction. The crude reaction mixture was purified by flash column chromatography (90:10 pentane/ethyl acetate) to give **5r** (231 mg, 50%) as a white solid.

R_F 0.46 (80:20 petroleum ether/ethyl acetate); **IR** (film) ν_{max}/cm^{-1}: 3161, 1453, 1390, 1282, 1247, 1151; **HRMS** (ESI): Calcd. for $C_{11}H_{10}F_3NONa^{+}$ ([M+Na]$^{+}$) *m*/*z* 252.0607, found *m*/*z* 252.0612;

^{1}H NMR ($CDCl_3$, 400 MHz) δ 7.80 – 7.63 (m, 2H, 2 × C_fH), 7.47 – 7.35 (m, 3H, C_gH, 2 × C_fH), 4.66 (s, 1H, O*H*), 2.91 (ddd, $^{2}J_{HH}$ = 6.7, $^{4}J_{HH}$ = 2.4, 1.2, 1H, C_aH), 2.32 (dd, $^{4}J_{HH}$ = 6.7, 2.5, 1H, C_aH), 1.70 (d, $^{4}J_{HH}$ = 2.4, 1H, C_aH), 1.37 (d, $^{4}J_{HH}$ = 2.5, 1H, C_aH); **^{13}C NMR** ($CDCl_3$, 101 MHz) δ 136.12 (C_d), 129.12 (C_g), 128.26 (C_e), 127.28 (C_f), 124.76 (d, $^{1}J_{CF}$ = 286.7, CF_3), 74.36 (q, $^{2}J_{CF}$ = 28.9, C_c), 56.38 (C_a), 52.81 (C_a), 34.37 (C_b); **^{19}F NMR** ($CDCl_3$, 377 MHz) δ –77.43. See spectra

Azabicyclo[1.1.0]butan-3-yl)diphenylmethanol, 5s

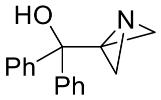

Prepared using general procedure 1. Benzophenone (0.474 g, 2.6 mmol, 1.3 equiv) was weighed into a vial which was evacuated and backfilled with nitrogen. Anhydrous tetrahydrofuran (1.0 mL) was added, and this solution was added dropwise to the reaction and a further portion of anhydrous tetrahydrofuran (0.5 mL) was used to wash the residual reagent from the vial into the reaction mixture. ^{1}H NMR yield: 73%. See spectrum

HRMS (ESI): Calcd. for $C_{16}H_{15}NONa^{+}$ ($[M+Na]^{+}$) *m/z* 260.1046, found *m/z* 260.1048.

(1-Azabicyclo[1.1.0]butan-3-yl)(4-methoxyphenyl)(phenyl)methanol, 5t

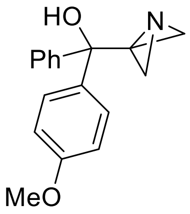

Prepared using general procedure 1. 4-Methoxybenzophenone (0.552 g, 2.6 mmol, 1.3 equiv) was weighed into a vial which was evacuated and backfilled with nitrogen. Anhydrous tetrahydrofuran (1.0 mL) was added and this solution was added dropwise to the reaction and a further portion of anhydrous tetrahydrofuran (0.5 mL) was used to wash the residual reagent from the vial into the reaction mixture. ^{1}H NMR yield: 70%. See spectrum

HRMS (ESI): Calcd. for $C_{17}H_{17}NO_2Na^{+}$ ($[M+Na]^{+}$) *m/z* 290.1151, found *m/z* 290.1153.

1.4. Initial reaction investigations

Benzyl 3-chloro-3-(1-hydroxy-1-phenylethyl)azetidine-1-carboxylate, 10

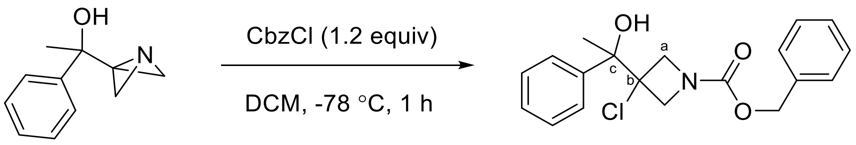

To a round bottom flask containing crude 1-(1-azabicyclo[1.1.0]butan-3-yl)-1-phenylethan-1-ol (**5a**, 0.25 mmol, 1.0 equiv) under an inert atmosphere was added anhydrous dichloromethane (2.5 mL). The flask was cooled to -78 °C and benzyl chloroformate (0.04 mL, 0.3 mmol, 1.2 equiv) was added with stirring. After 1 hour at -78 °C, diethyl ether (10 mL) and saturated aqueous sodium bicarbonate solution (10 mL) were added and the reaction mixture transferred to a separating funnel. The phases were separated, and the aqueous phase was extracted with diethyl ether (3 × 20 mL) and the combined organic phases were washed with brine, dried

($MgSO_4$), filtered and concentrated under reduced pressure. The crude reaction mixture was purified by flash column chromatography (95:5 → 80:20 pentane/acetone) to give **10** (64 mg, 74%) as a colourless oil.

Reaction repeated on a 0.1 mmol scale in anhydrous acetonitrile and 0 °C for 15 minutes. An NMR yield of 83% was achieved.

R_F 0.32 (80:20 pentane/acetone); **IR** (film) ν_{max}/cm^{-1}: 3400, 2948, 1694, 1446, 1424, 1356, 1213, 1130, 1058, 1033; **HRMS** (ESI): Calcd. for $C_{19}H_{20}ClNO_3Na^+$ ($[M+Na]^+$) *m/z* 368.1024, found *m/z* 368.1021;

^{1}H NMR ($CDCl_3$, 400 MHz) δ 7.57 – 7.49 (m, 2H, ArC*H*), 7.39 – 7.28 (m, 8H, ArC*H*), 5.05 (s, 2H, ROCH_2Ph), 4.69 (dd, $^2J_{HH}$ = 9.8, $^4J_{HH}$ = 1.2, 2H, 2 × C_aH), 4.19 (d, $^2J_{HH}$ = 9.6, 1H, C_aH), 3.94 (dd, $^2J_{HH}$ = 9.8, $^4J_{HH}$ = 1.2, 1H, C_aH), 2.79 (br. s, 1H, O*H*), 1.71 (s, 3H, CH_3); **^{13}C NMR** ($CDCl_3$, 101 MHz) δ 156.44 (R_2NC(O)OR), 142.20 (Ar*C*), 136.40 (Ar*C*), 128.61, 128.25 (Ar*C*H), 128.15 (Ar*C*H), 128.07 (Ar*C*H), 128.05 (Ar*C*H), 126.60 (Ar*C*H), 75.78 (C_c), 69.14 (C_b), 67.11 (RO*C*H_2Ph), 61.59 – 60.15 (br., 2 × C_a), 24.52 (*C*H_3). See spectra

Benzyl 3-(1-hydroxy-1-phenylethyl)-3-iodoazetidine-1-carboxylate, 11

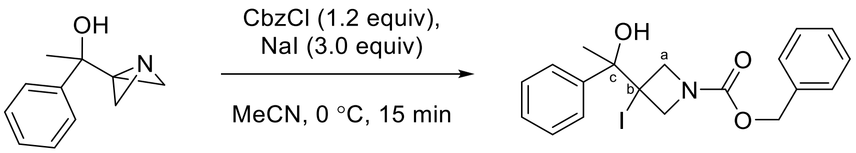

To a round bottom flask containing crude 1-(1-azabicyclo[1.1.0]butan-3-yl)-1-phenylethan-1-ol (**5a**, 0.25 mmol, 1.0 equiv) and sodium iodide (75 mg, 2.0 equiv) under an inert atmosphere was added anhydrous acetonitrile (2.5 mL). The flask was cooled to 0 °C and benzyl chloroformate (0.04 mL, 0.3 mmol, 1.2 equiv) was added with stirring. After 15 minutes at 0 °C, diethyl ether (10 mL) and saturated aqueous sodium bicarbonate solution (5 mL) were added and the reaction mixture transferred to a separating funnel. The phases were separated, and the aqueous phase was extracted with diethyl ether (3 × 20 mL) and the combined organic phases were washed with brine, dried ($MgSO_4$), filtered and concentrated under reduced pressure. The crude reaction mixture was purified by flash column chromatography (95:5 → 85:15 pentane/acetone) to give **11** (100 mg, 91%) as a colourless oil.

R_F 0.33 (80:20 pentane/acetone); **IR** (film) ν_{max}/cm^{-1}: 3433, 2969, 1693, 1446, 1422, 1356, 1194, 1129, 1059, 1033; **HRMS** (ESI): Calcd. for $C_{19}H_{20}INO_3Na^+$ ($[M+Na]^+$) *m*/*z* 460.0380, found *m*/*z* 460.0382;

^{1}H NMR ($CDCl_3$, 400 MHz) δ 7.49 – 7.43 (m, 2H, ArC*H*), 7.36 – 7.25 (m, 8H, ArC*H*), 5.02 (s, 2H, ROCH_2Ph), 4.97 (d, $^2J_{HH}$ = 10.3, 1H, C_aH), 4.89 (d, $^2J_{HH}$ = 9.9, 1H, C_aH), 4.48 (d, $^2J_{HH}$ = 9.9, 1H, C_aH), 4.31 (dd, $^2J_{HH}$ = 10.3, 1.3, 1H, C_aH), 2.65 (br. s, 1H, O*H*), 1.77 (s, 3H, CH_3); **^{13}C NMR** ($CDCl_3$, 101 MHz) δ (R_2N*C*(O)OR), 143.55 (br. Ar*C*), 136.43 (Ar*C*), 128.68 (Ar*C*H), 128.32 (Ar*C*H), 128.25 (Ar*C*H), 128.13 (Ar*C*H), 126.60 (Ar*C*H), 76.68 (C_c), 67.21 (RO*C*H_2Ph), 64.97 (br., 2 × C_a), 45.32 (C_b), 26.03 (*C*H_3). See spectra

2,2,2-Trifluoro-1-(3-(1-hydroxy-1-phenylethyl)-3-iodoazetidin-1-yl)ethan-1-one, 12

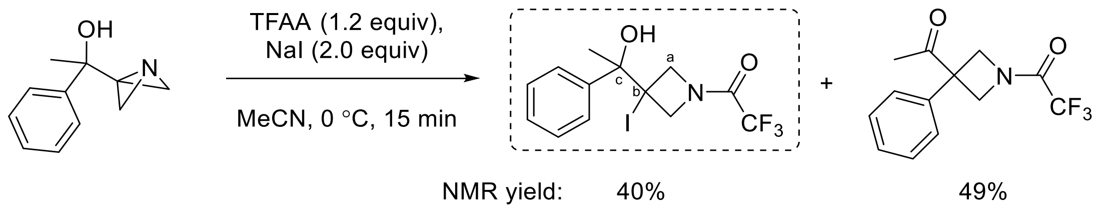

To a round bottom flask containing crude 1-(1-azabicyclo[1.1.0]butan-3-yl)-1-phenylethan-1-ol (**5a**, 0.25 mmol, 1.0 equiv) and sodium iodide (75 mg, 2.0 equiv) under an inert atmosphere was added anhydrous acetonitrile (2.5 mL). The flask was cooled to 0 °C and trifluoroacetic anhydride (0.04 mL, 0.3 mmol, 1.2 equiv) was added with stirring. After 15 minutes at 0 °C, diethyl ether (10 mL) and saturated aqueous sodium bicarbonate solution (5 mL) were added and the reaction mixture transferred to a separating funnel. The phases were separated, and the aqueous phase was extracted with diethyl ether (3 × 20 mL) and the combined organic phases were washed with brine, dried ($MgSO_4$), filtered and concentrated under reduced pressure. The crude reaction mixture was purified by flash column chromatography (50:50 DCM/pentane → DCM → 98:2 DCM/ethyl acetate). These column conditions were repeated on the mixed fractions. Mixed fractions were observed again and discarded. The combined clean fractions gave **12** (27 mg, 27%) as a colourless oil.

R_F 0.39 (95:5 DCM/ethyl acetate); **IR** (film) ν_{max}/cm^{-1}: 3432, 2970, 2922, 2852, 1684, 1455, 1253, 1206, 1148; **HRMS** (ESI): Calcd. for $C_{13}H_{13}F_3INO_2Na^+$ ($[M+Na]^+$) *m*/*z* 421.9835, found *m*/*z* 421.9832;

Rotamers observed.

^{1}H NMR ($CDCl_3$, 400 MHz) δ 7.52 (ddd, $^3J_{HH}$ = 7.8, 6.3, $^4J_{HH}$ = 1.5, 2H, 2 × C_fH), 7.43 – 7.30 (m, 3H, ArCH), 5.35 (d, $^2J_{HH}$ = 11.0, 1H, C_aH), 5.24 (d, $^2J_{HH}$ = 10.7, 1H, C_aH), 5.08 (d, $^2J_{HH}$ = 11.7, 1H, C_aH), 5.01 (d, $^2J_{HH}$ = 11.7, 1H, C_aH), 4.79 (dt, $^2J_{HH}$ = 10.7, $^4J_{HH}$ = 1.5, 1H, C_aH), 4.57 (d, $^2J_{HH}$ = 10.7, 2H, 2 × C_aH), 4.35 (dd, $^2J_{HH}$ = 11.9, $^4J_{HH}$ = 1.8, 1H, C_aH), 2.39 (br. s, 1H, OH), 2.37 (br. s, 1H, OH), 1.85 (s, 3H, CH_3), 1.84 (s, 3H, CH_3); **^{13}C NMR** ($CDCl_3$, 101 MHz) δ 156.35 (q, $^2J_{CF}$ = 37.9, $CF_3C(O)NR_2$), 156.27 (q, $^2J_{CF}$ = 37.9, $CF_3C(O)NR_2$), 142.55 (C_d), 141.82 (C_d), 128.58 (C_g), 128.52 (C_g), 128.42 (2 × C_e), 128.40 (2 × C_e), 126.74 (2 × C_f), 126.63 (2 × C_f), 115.99 (q, $^1J_{CF}$ = 288.1, CF_3), 115.96 (q, $^1J_{CF}$ = 288.1, CF_3), 76.40 (C_c), 76.37 (C_c), 67.29 (q, $^3J_{CF}$ = 2.38, C_a), 66.37 (q, $^3J_{CF}$ = 2.38, C_a), 64.61 (C_a), 63.71 (C_a), 42.42 (C_b), 26.89 (CH_3), 26.33 (CH_3); **^{19}F NMR** ($CDCl_3$, 377 MHz) δ –72.52, –72.56. See spectra

1.5. Optimisation of the semipinacol rearrangement reaction

To a round bottom flask containing crude 1-(1-azabicyclo[1.1.0]butan-3-yl)-1-phenylethan-1-ol (0.10 mmol, 1.0 equiv) under an inert atmosphere was added anhydrous solvent (1 mL). The flask was cooled to 0 °C or –78 °C and trifluoroacetic anhydride (16 µL, 0.12 mmol, 1.2 equiv) or trifluoromethanesulfonic anhydride (0.02 mL, 0.12 mmol, 1.2 equiv) was added with stirring. After the reaction time, diethyl ether or ethyl acetate (5 mL) and saturated aqueous sodium bicarbonate solution (5 mL) were added, and the reaction mixture transferred to a separating funnel. The phases were separated, and the aqueous phase was extracted with diethyl ether or ethyl acetate (3 × 5 mL) and the combined organic phases were washed with brine, dried ($MgSO_4$), filtered and concentrated under reduced pressure. An NMR yield of the product was measured.

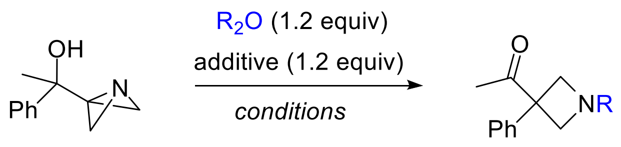

6a/7a

entry	R_2O	additive	solvent	temp, time	**6a/7a**
1	$(CF_3CO)_2O$		MeCN	0 °C, 15 mins	71%
2	$(CF_3CO)_2O$		DCM	-78 °C, 1 h	93%[a]
3	$(CF_3CO)_2O$	2,6-lutidene	DCM	-78 °C, 1 h	61%
4	Tf_2O		MeCN	0 °C, 15 mins	40%
5	Tf_2O		DCM	-78 °C, 1 h	67%
6	Tf_2O	2,6-lutidene	DCM	-78 °C, 1 h	79%[a]

[a] Performed on a 0.25 mmol scale and isolated yield given

1.6. Scope of the semipinacol rearrangement reaction

1-(3-Acetyl-3-phenylazetidin-1-yl)-2,2,2-trifluoroethan-1-one, 6a

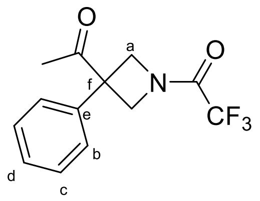

Prepared following general procedure 2a using the crude 1-(1-azabicyclo[1.1.0]butan-3-yl)-1-phenylethan-1-ol, **5a** (0.25 mmol). The crude reaction mixture was purified by flash column chromatography (95:5 → 75:25 pentane/ethyl acetate) to give **6a** (63 mg, 93%) as a white solid.

The same procedure was repeated on a 1.0 mmol scale (4 × scale) to give **6a** (243 mg, 90%) as a white solid.

R_F 0.23 (80:20 petroleum ether/ethyl acetate); **IR** (film) ν_{max}/cm^{-1}: 2956, 2922, 1695, 1468, 1221, 1205, 1150; **HRMS** (ESI): Calcd. for $C_{13}H_{13}F_3NO_2^+$ ($[M+H]^+$) *m/z* 272.0893, found *m/z* 272.0891;

^{1}H NMR ($CDCl_3$, 400 MHz) δ 7.56 – 7.41 (m, 2H, C_c*H*), 7.41 – 7.34 (m, 1H, C_d*H*), 7.24 – 7.18 (m, 2H, C_b*H*), 5.10 (dt, $^2J_{HH}$ = 9.5, $^4J_{HH}$ = 1.3, 1H, C_a*H*), 4.66 (d, $^2J_{HH}$ = 10.5, 1H, C_a*H*), 4.61 – 4.51 (m, 2H, C_a*H*), 2.05 (s, 3H, CH_3); **^{13}C NMR** ($CDCl_3$, 101 MHz) δ 203.66 (CH_3*C*(O)C_f), 156.44 (q, $^2J_{CF}$ = 38.5, CF_3*C*(O)NR_2), 137.79 (*C_e*), 129.63 (2 × *C_c*), 128.55 (*C_d*),

126.23 (2 × C_b), 116.06 (q, $^{1}J_{CF}$ = 288.2, CF3), 58.82 (q, $^{4}J_{CF}$ = 2.5, C_a), 55.70 (C_a), 54.36 (C_f), 24.66 (CH_3); **^{19}F NMR** ($CDCl_3$, 377 MHz) δ –72.54. See spectra.

1-(3-Phenyl-1-((trifluoromethyl)sulfonyl)azetidin-3-yl)ethan-1-one, 7a

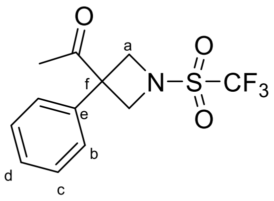

Prepared following general procedure 2b using the crude 1-(1-azabicyclo[1.1.0]butan-3-yl)-1-phenylethan-1-ol, **5a** (0.25 mmol). The crude reaction mixture was purified by flash column chromatography (98:2 → 80:20 pentane/ethyl acetate) to give **7a** (60 mg, 79%) as a white solid.

R_F 0.39 (80:20 petroleum ether/ethyl acetate); **IR** (film) ν_{max}/cm^{-1}: 2965, 2918, 1714, 1383, 1225, 1193; **HRMS** (ESI): Calcd. for $C_{12}H_{12}F_3NO_3SNa^+$ ($[M+Na]^+$) m/z 330.0382, found m/z 330.0376;

^{1}H NMR ($CDCl_3$, 400 MHz) δ 7.49 – 7.41 (m, 2H, C_cH), 7.41 – 7.35 (m, 1H, C_dH), 7.20 – 7.12 (m, 2H, C_bH), 4.80 (d, $^{2}J_{HH}$ = 7.9, 2H, 2 × C_aH), 4.53 (d, $^{2}J_{HH}$ = 7.9, 2H, 2 × C_aH), 2.05 (CH_3). **^{13}C NMR** ($CDCl_3$, 101 MHz) δ 203.02 ($CH_3C(O)C_f$), 137.32 (C_e), 129.70 (2 × C_c), 128.71 (C_d), 126.03 (2 × C_b), 119.87 (q, $^{1}J_{CF}$ = 322.3, CF3), 58.76 (2 × C_a), 53.25 (C_f), 24.61 (CH_3); **^{19}F NMR** ($CDCl_3$, 377 MHz) δ –75.28. See spectra

1-(3-Acetyl-3-(4-methoxyphenyl)azetidin-1-yl)-2,2,2-trifluoroethan-1-one, 6b

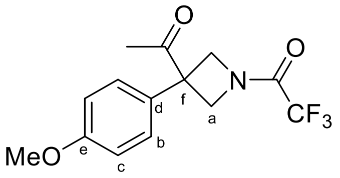

Prepared following general procedure 2a using the crude 1-(1-azabicyclo[1.1.0]butan-3-yl)-1-(4-methoxyphenyl)ethan-1-ol, **5b** (0.25 mmol). The crude reaction mixture was purified by flash column chromatography (95:5 → 65:35 pentane/ethyl acetate) to give **6b** (66 mg, 88%) as an off-white solid.

R_F 0.38 (70:30 petroleum ether/ethyl acetate); **IR** (film) ν_{max}/cm^{-1}: 2981, 2870, 1698, 1514, 1251, 1145; **HRMS** (ESI): Calcd. for $C_{14}H_{15}F_3NO_3^+$ ($[M+H]^+$) m/z 302.0999, found m/z 302.1000;

^{1}H NMR ($CDCl_3$, 400 MHz) δ 7.12 (m, 2H, 2 × C_bH), 6.95 (m, 2H, 2 × C_cH), 5.07 (d, $^{2}J_{HH}$ = 9.5, 1H, C_aH), 4.63 (d, $^{2}J_{HH}$ = 10.6, 1H, C_aH), 4.53 (d, $^{2}J_{HH}$ = 9.5, 1H, C_aH), 4.49 (d, $^{2}J_{HH}$ = 10.6, 1H, C_aH), 3.82 (s, 3H, OCH_3), 2.04 (s, 3H, $C(O)CH_3$); **^{13}C NMR** ($CDCl_3$, 101 MHz) δ 204.00

(CH_3*C*(O)C_f), 159.66 (C_e), 156.41 (q, $^2J_{CF}$ = 37.7), 129.55 (C_d), 127.44 (2 × C_b), 116.04 (q, $^1J_{CF}$ = 285.86, CF_3), 114.96 (2 × C_c), 58.81 (q, $^4J_{CF}$ = 2.4, C_a), 55.68 (C_a), 55.46 (OCH_3), 53.66 (C_f), 24.48 (C(O)CH_3); **^{19}F NMR** ($CDCl_3$, 377 MHz) δ –72.56. See spectra

1-(3-(4-Methoxyphenyl)-1-((trifluoromethyl)sulfonyl)azetidin-3-yl)ethan-1-one, 7b

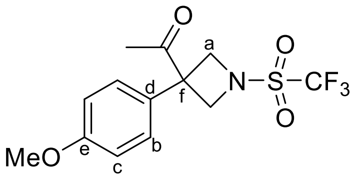

Prepared following general procedure 2b using the crude 1-(1-azabicyclo[1.1.0]butan-3-yl)-1-(4-methoxyphenyl)ethan-1-ol, **5b** (0.25 mmol). The crude reaction mixture was purified by flash column chromatography (95:5 → 65:35 pentane/ethyl acetate) to give **7b** (60 mg, 71%) as a white solid.

R_F 0.51 (70:30 petroleum ether/ethyl acetate); **IR** (film) ν_{max}/cm^{-1}: 2982, 1869, 1707, 1514, 1384, 1375, 1224, 1190; **HRMS** (ESI): Calcd. for $C_{13}H_{14}F_3NO_4SNa^+$ ($[M+Na]^+$) *m/z* 360.0488, found *m/z* 360.0489;

^{1}H NMR ($CDCl_3$, 400 MHz) δ 7.08 (m, 2H, 2 × C_b*H*), 6.94 (m, 2H, 2 × C_c*H*), 4.76 (d, $^2J_{HH}$ = 8.1, 2H, 2 × C_a*H*), 4.46 (d, $^2J_{HH}$ = 8.1, 2H, 2 × C_a*H*), 3.80 (s, 3H, OCH_3), 2.02 (s, 3H, C(O)CH_3); **^{13}C NMR** ($CDCl_3$, 101 MHz) δ 203.32 (CH_3*C*(O)C_f), 159.77 (C_e), 129.05 (C_d), 127.25 (2 × C_b), 119.85 (q, $^1J_{CF}$ = 322.4), 115.04 (2 × C_c), 58.77 (C_a), 55.47 (OCH_3), 52.58 (C_f), 24.44 (C(O)CH_3); **^{19}F NMR** ($CDCl_3$, 377 MHz) δ –75.13. See spectra

1-(3-Acetyl-3-(4-(trifluoromethyl)phenyl)azetidin-1-yl)-2,2,2-trifluoroethan-1-one, 6c

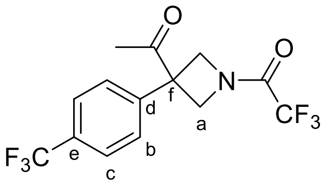

Prepared following general procedure 2a using the crude 1-(1-azabicyclo[1.1.0]butan-3-yl)-1-(4-(trifluoromethyl)phenyl)ethan-1-ol, **5c** (0.25 mmol). The crude reaction mixture was purified by flash column chromatography (95:5 → 70:30 pentane/ethyl acetate) to give **6c** (74 mg, 87%) as a white solid.

R_F 0.25 (70:30 petroleum ether/ethyl acetate); **IR** (film) ν_{max}/cm^{-1}: 2981, 2870, 1705, 1332, 1146, 1130; **HRMS** (ESI): Calcd. for $C_{14}H_{11}F_6NO_2Na^+$ ($[M+Na]^+$) *m/z* 362.0586, found *m/z* 362.0582;

^{1}H NMR ($CDCl_3$, 400 MHz) δ 7.71 (d, $^3J_{HH}$ = 8.2, 2H, 2 × C_c*H*), 7.36 (d, $^3J_{HH}$ = 8.2, 2H, 2 × C_b*H*), 5.12 (d, $^2J_{HH}$ = 9.6, 1H, C_a*H*), 4.69 (d, $^2J_{HH}$ = 10.7, 1H, C_a*H*), 4.58 (d, $^2J_{HH}$ = 9.6, 1H,

C$_a$*H*), 4.54 (d, $^2J_{HH}$ = 10.7, 1H, C$_a$*H*), 2.07 (s, 1H, C*H*$_3$); **^{13}C NMR** (CDCl$_3$, 101 MHz) δ 202.68 (CH$_3$*C*(O)C$_f$), 156.44 (q, $^2J_{CF}$ = 37.9, CF$_3$*C*(O)NR$_2$), 141.74 (*C$_d$*), 130.97 (q, $^2J_{CF}$ = 33.1, *C$_e$*), 126.84 (2 × *C$_b$*), 126.65 (q, $^3J_{CF}$ = 3.7, 2 × *C$_c$*), 123.75 (q, $^1J_{CF}$ = 272.4, Ar*C*F$_3$), 115.98 (q, $^1J_{CF}$ = 288.1, C(O)*C*F$_3$), 58.76 (q, $^4J_{CF}$ = 2.4, *C$_a$*), 55.68 (*C$_a$*), 54.41 (*C$_f$*), 24.76 (*C*H$_3$); **^{19}F NMR** (CDCl$_3$, 377 MHz) δ -62.89, –72.72. See spectra

1-(3-(4-(Trifluoromethyl)phenyl)-1-((trifluoromethyl)sulfonyl)azetidin-3-yl)ethan-1-one, 7c

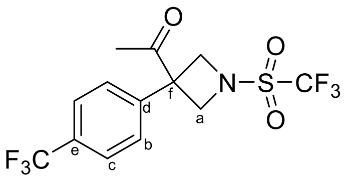

Prepared following general procedure 2a using the crude 1-(1-azabicyclo[1.1.0]butan-3-yl)-1-(4-(trifluoromethyl)phenyl)ethan-1-ol, **5c** (0.25 mmol). The crude reaction mixture was purified by flash column chromatography (95:5 → 70:30 pentane/ethyl acetate) to give **7c** (56 mg, 60%) as a white solid.

R$_F$ 0.20 (70:30 petroleum ether/ethyl acetate); **IR** (film) ν_{max}/cm^{-1}: 2981, 2870, 1711, 1377, 1332, 1200, 1161, 1120; **HRMS** (ESI): Calcd. for $C_{13}H_{11}F_6NO_3SNa^+$ ([M+Na]$^+$) *m/z* 398.0256, found *m/z* 398.0256;

^{1}H NMR (CDCl$_3$, 400 MHz) δ 7.73 (d, $^3J_{HH}$ = 8.2, 2H, 2 × C$_c$*H*), 7.33 (d, $^3J_{HH}$ = 8.2, 2H, 2 × C$_b$*H*), 4.83 (d, $^2J_{HH}$ = 8.1, 1H, 2 × C$_a$*H*), 4.53 (d, $^2J_{HH}$ = 8.1, 2H, 2 × C$_a$*H*), 2.08 (s, 3H, C*H*$_3$); **^{13}C NMR** (CDCl$_3$, 101 MHz) δ 202.03 (CH$_3$*C*(O)C$_f$), 141.23 (*C$_d$*), 131.22 (q, $^2J_{CF}$ = 33.0, *C$_e$*), 126.80 (q, $^3J_{CF}$ = 3.7, 2 × *C$_c$*), 126.64 (2 × *C$_b$*), 123.72 (q, $^1J_{CF}$ = 272.4, Ar*C*F$_3$), 119.81 (q, $^1J_{CF}$ = 322.1, SO$_2$*C*F$_3$), 58.65 (*C$_a$*), 53.27 (*C$_f$*), 24.78 (*C*H$_3$); **^{19}F NMR** (CDCl$_3$, 377 MHz) δ -62.89, –75.33. See spectra

***tert*-Butyl 3-(3-acetyl-1-(2,2,2-trifluoroacetyl)azetidin-3-yl)-1H-indole-1-carboxylate, 6d**

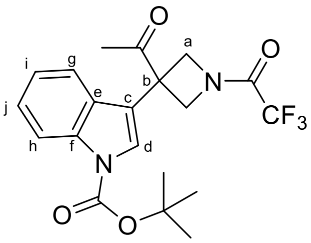

Prepared following general procedure 2a using the crude tert-butyl 3-(1-(1-azabicyclo[1.1.0]butan-3-yl)-1-hydroxyethyl)-1H-indole-1-carboxylate, **5d** (0.25 mmol). The crude reaction mixture was purified by flash column chromatography (98:2 → 80:20 pentane/ethyl acetate) to give **6d** (96 mg, 94%) as a white solid.

R$_F$ 0.10 (90:10 pentane/ethyl acetate); **IR** (film) ν_{max}/cm^{-1}: 2981, 2871, 1697, 1452, 1372, 1245, 1227, 1147; **HRMS** (ESI): Calcd. for $C_{20}H_{21}F_3N_2O_4Na^+$ ([M+Na]$^+$) *m/z* 433.1346, found *m/z* 433.1360;

^{1}H NMR ($CDCl_3$, 400 MHz) δ 8.17 (br. d, $^3J_{HH}$ = 8.3, 1H, C_hH), 7.70 (s, 1H, C_dH), 7.37 (ddd, $^3J_{HH}$ = 8.3, 7.1, $^4J_{HH}$ = 1.3, 1H, C_jH), 7.23 (ddd, $^3J_{HH}$ = 8.0, 7.1, $^4J_{HH}$ = 1.0, 1H, C_iH), 7.15 (dt, $^3J_{HH}$ = 8.0, $^4J_{HH}$ = 1.0, 1H, C_gH), 5.19 (d, $^2J_{HH}$ = 9.2, 1H), 4.67 (d, $^2J_{HH}$ = 10.3, 1H), 4.53 (d, $^2J_{HH}$ = 10.3, 2H), 4.50 (d, $^2J_{HH}$ = 9.2, 1H), 2.08 (s, 3H, C(O)OCH_3), 1.71 (s, 9H, C(CH_3)$_3$); **^{13}C NMR** ($CDCl_3$, 101 MHz) δ 203.92 (CH_3C(O)C_b), 156.77 (q, $^2J_{HH}$ = 37.9, CF_3C(O)NR_2), 149.50 (R_2NC(O)OR), 136.17 (C_f), 127.55 (C_e), 125.80 (C_j), 123.77 (C_d), 123.63 (C_i), 119.23 (C_g), 118.01 (C_b), 116.13 (q, $^1J_{HH}$ = 287.7, CF_3), 116.02 (C_h), 85.04 (R_2NC(O)OC(CH_3)$_3$), 58.18 (q, $^3J_{HH}$ = 2.3, C_a), 55.33 (C_a), 48.73 (C_b), 28.38 ((CH_3)$_3$), 24.79 (C(O)OCH_3); **^{19}F NMR** ($CDCl_3$, 377 MHz) δ –72.62. See spectra

***tert*-Butyl 3-(3-acetyl-1-((trifluoromethyl)sulfonyl)azetidin-3-yl)-1H-indole-1-carboxylate, 7d**

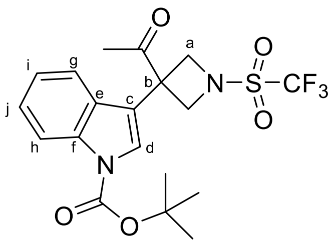

Prepared following general procedure 2b using the crude tert-butyl 3-(1-(1-azabicyclo[1.1.0]butan-3-yl)-1-hydroxyethyl)-1H-indole-1-carboxylate, **5d** (0.25 mmol). The crude reaction mixture was purified by flash column chromatography (DCM) to give **7d** (96 mg, 86%) as a white solid.

R$_F$ 0.46 (DCM); **IR** (film) ν_{max}/cm^{-1}: 2981, 2870, 1718, 1453, 1373, 1152; **HRMS** (ESI): Calcd. for $C_{19}H_{21}F_3N_2O_5SNa^+$ ([M+Na]$^+$) *m/z* 469.1015, found *m/z* 469.1013;

^{1}H NMR ($CDCl_3$, 400 MHz) δ 8.17 (br. d, $^3J_{HH}$ = 8.4, 1H, C_hH), 7.69 (s, 1H, C_dH), 7.38 (ddd, $^3J_{HH}$ = 8.4, 7.2, $^4J_{HH}$ = 1.1, 1H, C_jH), 7.23 (ddd, $^3J_{HH}$ = 7.9, 7.2, $^4J_{HH}$ = 1.0, 1H, C_iH), 7.13 (dt, $^3J_{HH}$ = 7.9, $^4J_{HH}$ = 1.1, 1H, C_gH), 4.87 (d, $^2J_{HH}$ = 7.9, 2H, 2 × C_aH), 4.49 (d, $^2J_{HH}$ = 7.9, 2H, 2 × C_aH), 2.08 (s, 3H, C(O)CH_3), 1.72 (s, 9H, C(CH_3)$_3$); **^{13}C NMR** ($CDCl_3$, 101 MHz) δ 203.07 (CH_3C(O)C_b), 149.40 (R_2NC(O)C(CH_3)$_3$), 136.02 (C_f), 127.29 (C_e), 125.74 (C_j), 123.59 (C_j), 123.56 (C_i), 119.86 (q, $^1J_{CF}$ = 322.7, CF_3), 119.09 (C_g), 117.42 (C_b), 115.95 (C_h), 85.03 (R_2NC(O)OC(CH_3)$_3$), 58.16 (2 × C_a), 47.63 (C_b), 28.29 ((CH_3)$_3$), 24.63 (C(O)OC(CH_3)$_3$); **^{19}F NMR** ($CDCl_3$, 377 MHz) δ –75.21. See spectra

1-(3-Acetyl-3-(6-methoxypyridin-3-yl)azetidin-1-yl)-2,2,2-trifluoroethan-1-one, 6e

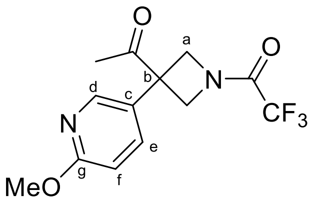

Prepared following general procedure 2a using the crude 1-(1-azabicyclo[1.1.0]butan-3-yl)-1-(6-methoxypyridin-3-yl)ethan-1-ol, **5e** (0.25 mmol). The crude reaction mixture was purified by flash column chromatography (98:2 → 80:20 pentane/acetone) to give **6e** (53 mg, 70%) as a colourless oil.

R$_F$ 0.09 (90:10 pentane/acetone); **IR** (film) ν_{max}/cm^{-1}: 2952, 2925, 1698, 1606, 1497, 1380, 1294, 1257,1206, 1151; **HRMS** (ESI): Calcd. for $C_{13}H_{13}F_3N_2O_3Na^+$ ([M+Na]$^+$) *m/z* 325.0770, found *m/z* 325.0786;

^{1}H NMR ($CDCl_3$, 400 MHz) δ 8.07 (dd, $^4J_{HH}$ = 2.7, $^5J_{HH}$ = 0.8, 1H, C_dH), 7.37 (dd, $^3J_{HH}$ = 8.7, $^4J_{HH}$ = 2.7, 1H, C_eH), 6.80 (dd, $^3J_{HH}$ = 8.7, $^5J_{HH}$ = 0.8, 1H, C_fH), 5.07 (dt, $^2J_{HH}$ = 9.6, $^4J_{HH}$ = 1.3, 1H, C_aH), 4.65 (d, $^2J_{HH}$ = 10.6, 1H, C_aH), 4.52 (dt, $^2J_{HH}$ = 9.9, $^4J_{HH}$ = 1.3, 1H, C_aH), 4.48 (d, $^2J_{HH}$ = 10.6, 1H, C_aH), 3.93 (s, 3H, OCH_3), 2.08 (s, 3H, RC(O)CH_3); **^{13}C NMR** ($CDCl_3$, 101 MHz) δ 203.35 (CH_3C(O)C_b), 164.22 (C_g), 156.46 (q, $^2J_{CF}$ = 37.8, R_2NC(O)CF_3), 144.93 (C_d), 136.54 (C_e), 126.06 (C_c), 115.98 (q, $^1J_{CF}$ = 288.1, CF_3), 112.10 (C_f), 58.70 (q, $^1J_{CF}$ = 2.4, C_a), 55.56 (C_a), 53.85 (OCH_3), 52.08 (C_b), 24.62 (RC(O)CH_3); **^{19}F NMR** ($CDCl_3$, 377 MHz) δ –72.68. See spectra

1-(3-(6-Methoxypyridin-3-yl)-1-((trifluoromethyl)sulfonyl)azetidin-3-yl)ethan-1-one, 7e

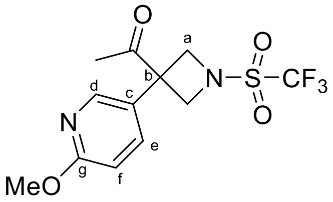

Prepared following general procedure 2b using the crude 1-(1-azabicyclo[1.1.0]butan-3-yl)-1-(6-methoxypyridin-3-yl)ethan-1-ol, **5e** (0.25 mmol). The crude reaction mixture was purified by flash column chromatography (98:2 → 85:15 pentane/acetone) then further purified by flash column chromatography (100:0 → 95:5 DCM/ethyl acetate) to give **7e** (57 mg, 67%) as a white solid.

R$_F$ 0.46 (95:5 DCM/ethyl acetate); **IR** (film) ν_{max}/cm^{-1}: 2988, 2852, 1709, 1612, 1497, 1380, 1297, 1227, 1195; **HRMS** (ESI): Calcd. for $C_{12}H_{13}F_3N_2O_4SNa^+$ ([M+Na]$^+$) *m/z* 361.0440, found *m/z* 361.0458

^{1}H NMR ($CDCl_3$, 400 MHz) δ 8.05 (dd, $^4J_{HH}$ = 2.7, $^5J_{HH}$ = 0.8, 1H, C_dH), 7.35 (dd, $^3J_{HH}$ = 8.7, $^4J_{HH}$ = 2.7, 1H, C_eH), 6.81 (dd, $^3J_{HH}$ = 8.7, $^5J_{HH}$ = 0.8, 1H, C_fH), 4.79 (d, $^2J_{HH}$ = 8.1, 2H,

2 × C$_a$*H*), 4.46 (d, $^2J_{HH}$ = 8.1, 2H, 2 × C$_a$*H*), 3.95 (s, 3H, OC*H*$_3$), 2.08 (s, 3H, RC(O)C*H*$_3$); **^{13}C NMR** ($CDCl_3$, 101 MHz) δ 202.74 (CH$_3$*C*(O)C$_b$), 164.29 (*C$_g$*), 144.74 (*C$_d$*), 136.33 (*C$_e$*), 125.58 (*C$_c$*), 119.78 (q, $^1J_{CF}$ = 322.2, *C*F$_3$), 112.21 (*C$_f$*), 58.58 (2 × *C$_a$*), 53.87 (O*C*H$_3$), 50.94 (*C$_b$*), 24.59 (RC(O)*C*H$_3$); **^{19}F NMR** ($CDCl_3$, 377 MHz) δ –75.15. See spectra

1-(3-Acetyl-3-(6-bromopyridin-3-yl)azetidin-1-yl)-2,2,2-trifluoroethan-1-one, 6f

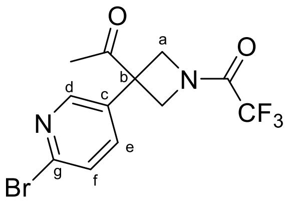

Prepared following general procedure 2a using the crude 1-(1-azabicyclo[1.1.0]butan-3-yl)-1-(6-bromopyridin-3-yl)ethan-1-ol, **5f** (0.25 mmol). The crude reaction mixture was purified by flash column chromatography (95:5 DCM/ethyl acetate) then further purified by flash column chromatography (95:5 → 80:20 pentane/acetone) to give **6f** (51 mg, 58%) as a colourless oil.

R$_F$ 0.25 (80:20 pentane/acetone); **IR** (film) ν_{max}/cm^{-1}: 2956, 1693, 1456, 1366, 1205, 1148; **HRMS** (ESI): Calcd. for $C_{12}H_{10}BrF_3N_2O_2Na^+$ ([M+Na]$^+$) *m/z* 372.9770, found *m/z* 372.9792.

^{1}H NMR ($CDCl_3$, 400 MHz) δ 8.30 (dd, $^4J_{HH}$ = 2.8, $^5J_{HH}$ = 0.8, 1H, C$_d$*H*), 7.57 (dd, $^3J_{HH}$ = 8.3, $^5J_{HH}$ = 0.8, 1H, C$_f$*H*), 7.40 (dd, $^3J_{HH}$ = 8.3, $^4J_{HH}$ = 2.7, 1H, C$_e$*H*), 5.10 (dt, $^2J_{HH}$ = 9.9, $^4J_{HH}$ = 1.0, 1H, C$_a$*H*), 4.69 (dd, $^2J_{HH}$ = 10.6, $^4J_{HH}$ =1.0, 1H, C$_a$*H*), 4.56 (d, $^2J_{HH}$ = 9.9, 1H, C$_a$*H*), 4.50 (d, $^2J_{HH}$ = 10.6, 1H, C$_a$*H*), 2.11 (s, 3H, C*H*$_3$); **^{13}C NMR** ($CDCl_3$, 101 MHz) δ 202.18 (CH$_3$*C*(O)C$_b$), 156.39 (q, $^2J_{CF}$ = 38.0, R$_2$N*C*(O)CF$_3$), 148.12 (*C$_d$*), 142.58 (*C$_c$*), 136.47 (*C$_e$*), 132.94 (*C$_g$*), 128.94 (*C$_f$*), 115.87 (q, $^1J_{CF}$ = 288.1, *C*F$_3$), 58.62 (q, $^1J_{CF}$ = 2.7, *C$_a$*), 55.52 (*C$_a$*), 52.30 (*C$_b$*), 24.86 (*C*H$_3$); **^{19}F NMR** ($CDCl_3$, 377 MHz) δ –72.53. See spectra

1-(3-(6-Bromopyridin-3-yl)-1-((trifluoromethyl)sulfonyl)azetidin-3-yl)ethan-1-one, 7f

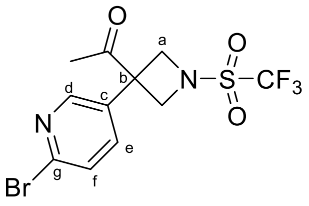

Prepared following general procedure 2b using the crude 1-(1-azabicyclo[1.1.0]butan-3-yl)-1-(6-bromopyridin-3-yl)ethan-1-ol, **5f** (0.25 mmol). The crude reaction mixture was purified by flash column chromatography (98:2 → 85:15 pentane/acetone) then further purified by flash column chromatography (95:5 DCM/ethyl acetate) to give **7f** (52 mg, 54%) as a white solid.

R$_F$ 0.39 (95:5 DCM/ethyl acetate); **IR** (film) ν_{max}/cm^{-1}: 2981, 2872, 1715, 1580, 1557, 1459, 1379, 1230, 1196; **HRMS** (ESI): Calcd. for $C_{11}H_{10}BrF_3N_2O_3SNa^+$ ($[M+Na]^+$) *m/z* 408.9440, found *m/z* 408.9452.

^{1}H NMR ($CDCl_3$, 400 MHz) δ 8.28 (d, $^4J_{HH}$ = 2.7, 1H, C_dH), 7.59 (dd, $^3J_{HH}$ = 8.4, $^5J_{HH}$ = 0.8, 1H, C_fH), 7.37 (dd, $^3J_{HH}$ = 8.4, $^4J_{HH}$ = 2.7, 1H, C_eH), 4.82 (d, $^2J_{HH}$ = 8.3, 2H, 2 × C_aH), 4.49 (d, $^2J_{HH}$ = 8.2, 2H, 2 × C_aH), 2.12 (s, 3H, CH_3); **^{13}C NMR** ($CDCl_3$, 101 MHz) δ 201.62 ($CH_3C(O)C_b$), 147.91 (C_d), 142.82 (C_c), 136.26 (C_e), 132.52 (C_g), 129.06 (C_f), 119.70 (q, $^1J_{CF}$ = 322.0, CF_3), 58.49 (2 × C_a), 51.16 (C_b), 24.86 (CH_3); **^{19}F NMR** ($CDCl_3$, 377 MHz) δ –75.23. See spectra

1-(3-(2-Chloropyridin-4-yl)-1-((trifluoromethyl)sulfonyl)azetidin-3-yl)ethan-1-one, 7g

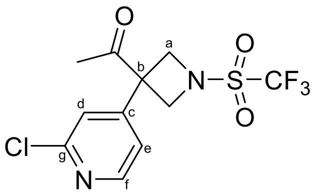

Prepared following general procedure 2b using the crude 1-(1-azabicyclo[1.1.0]butan-3-yl)-1-(2-chloropyridin-4-yl)ethan-1-ol, **5g** (0.25 mmol). The crude reaction mixture was purified by flash column chromatography (95:5 → 75:25 pentane/acetone) to give **7g** (20 mg, 23%) as a white solid.

R$_F$ 0.38 (80:20 pentane/acetone); **IR** (film) ν_{max}/cm^{-1}: 2967, 2944, 1720, 1591, 1543, 1466, 1380, 1225, 1194; **HRMS** (ESI): Calcd. for $C_{11}H_{10}F_3ClN_2O_3SNa^+$ ($[M+Na]^+$) *m/z* 343.0126, found *m/z* 343.0120;

^{1}H NMR ($CDCl_3$, 400 MHz) δ 8.49 (dd, $^3J_{HH}$ = 5.2, $^5J_{HH}$ = 0.7, 1H, C_fH), 7.19 (dd, $^4J_{HH}$ = 1.7, $^5J_{HH}$ = 0.7, 1H, C_dH), 7.06 (dd, $^3J_{HH}$ = 5.2, $^4J_{HH}$ = 1.7, 1H), 4.79 (d, $^2J_{HH}$ = 8.30, 2H, 2 × C_aH), 4.47 (d, $^2J_{HH}$ = 8.30, 2H, 2 × C_aH), 2.12 (s, 3H, CH_3); **^{13}C NMR** ($CDCl_3$, 101 MHz) δ 200.69 ($CH_3C(O)C_b$), 153.26 (C_g), 151.12 (C_f), 149.13 (C_c), 121.78 (C_d), 119.67 (q, $^1J_{CF}$ = 322.2, CF_3), 119.66 (C_g), 58.08 (2 × C_a), 52.54 (C_b), 24.88 (CH_3); **^{19}F NMR** ($CDCl_3$, 377 MHz) δ –75.28. See spectra

2-(2,2,2-Trifluoroacetyl)-2-azaspiro[3.4]octan-5-one, 6h

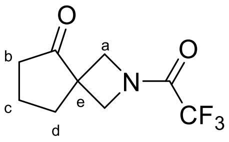

Prepared following general procedure 2a using the crude 1-(1-azabicyclo[1.1.0]butan-3-yl)cyclobutan-1-ol, **5h** (0.25 mmol). The crude reaction mixture was purified by flash column chromatography (95:5 → 30:70 pentane/ethyl acetate) to give **6h** (30 mg, 54%) as a colourless oil.

R$_F$ 0.18 (70:30 petroleum ether/ethyl acetate); **IR** (film) ν_{max}/cm^{-1}: 2978, 2872, 1741, 1694, 1464, 1253, 1199, 1143; **HRMS** (ESI): Calcd. for $C_9H_{11}F_3NO_2^+$ ($[M+H]^+$) *m/z* 222.0736, found *m/z* 222.0731;

^{1}H NMR ($CDCl_3$, 400 MHz) δ 4.47 (d, $^2J_{HH}$ = 9.3, 1H, C_aH), 4.18 (d, $^2J_{HH}$ = 10.2, 1H, C_aH), 4.14 (d, $^2J_{HH}$ = 9.3, 1H, C_aH), 3.89 (d, $^2J_{HH}$ = 10.2, 1H, C_aH), 2.36 – 2.18 (m, 4H, C_bH_2, C_dH_2), 1.96 – 1.88 (m, 2H, C_cH_2); **^{13}C NMR** ($CDCl_3$, 101 MHz) δ 216.66 ($C_bC(O)C_e$), 156.40 (q, $^2J_{CF}$ = 37.9, $CF_2C(O)NR_2$), 116.08 (q, $^1J_{CF}$ = 289.0, CF_3), 59.01 (q, $^4J_{CF}$ = 2.2, C_a), 56.71 (C_a), 45.60 (C_e), 36.88 (C_b), 34.88 (C_d), 19.28 (C_c); **^{19}F NMR** ($CDCl_3$, 377 MHz) δ –72.56. See spectra

2-((Trifluoromethyl)sulfonyl)-2-azaspiro[3.4]octan-5-one, 7h

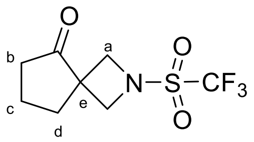

Prepared following general procedure 2b using the crude 1-(1-azabicyclo[1.1.0]butan-3-yl)cyclobutan-1-ol, **5h** (0.25 mmol). The crude reaction mixture was purified by flash column chromatography (95:5 → 50:50 pentane/ethyl acetate) to give **7h** (56 mg, 87%) as a white solid.

R$_F$ 0.20 (70:30 petroleum ether/ethyl acetate); **IR** (film) ν_{max}/cm^{-1}: 2978, 2873, 1734, 1380, 1218, 1193, 1155, 1178, 1088; **HRMS** (ESI): Calcd. for $C_8H_{10}F_3NO_3SNa^+$ ($[M+Na]^+$) *m/z* 280.0226, found *m/z* 280.0216;

^{1}H NMR ($CDCl_3$, 400 MHz) δ 4.30 (d, $^2J_{HH}$ = 7.5, 2H, 2 × C_aH), 3.95 (d, $^2J_{HH}$ = 7.5, 2H, 2 × C_aH), 2.29 (t, $^3J_{HH}$ = 7.8, 2H, C_bH_2), 2.27 (t, $^3J_{HH}$ = 6.8, 2H, C_dH_2), 1.91 (m, 2H, C_cH_2); **^{13}C NMR** ($CDCl_3$, 126 MHz) δ 215.69 ($C_bC(O)C_e$), 120.01 (q, $^1J_{CF}$ = 325.4, CF_3), 59.48 (2 × C_a), 44.51 (C_e), 36.79 (C_b), 34.75 (C_d), 19.18 (C_c).; **^{19}F NMR** ($CDCl_3$, 377 MHz) δ -74.98. See spectra

2-(2,2,2-Trifluoroacetyl)-2-azaspiro[3.5]nonan-5-one, 6i

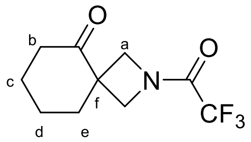

Prepared following general procedure 2a using the crude 1-(1-azabicyclo[1.1.0]butan-3-yl)cyclopentan-1-ol, **5i** (0.25 mmol). The crude reaction mixture was purified by flash column chromatography (95:5 → 75:25 pentane/ethyl acetate) to give **6i** (26 mg, 44%) as a colourless oil.

R_F 0.14 (80:20 petroleum ether/ethyl acetate); **IR** (film) ν_{max}/cm^{-1}: redo, 2944, 2863, 1695, 1465, 1448, 1250, 1200, 1145; **HRMS** (ESI): Calcd. for $C_{10}H_{13}F_3NO_2^+$ ($[M+H]^+$) *m*/*z* 236.0893, found *m*/*z* 236.0891;

^{1}H NMR ($CDCl_3$, 400 MHz) δ 4.72 (d, $^2J_{HH}$ = 9.5, 1H, C_aH), 4.30 (d, $^2J_{HH}$ = 10.4, 1H, C_aH), 3.97 (d, $^2J_{HH}$ = 9.5, 1H, C_aH), 3.85 (d, $^2J_{HH}$ = 10.4, 1H, C_aH), 2.39 (t, J = 6.6, 2H, C_bH_2), 2.22 – 2.10 (m, 1H, C_eH), 2.04 (ddd, $^2J_{HH}$ = 13.4, $^3J_{HH}$ = 8.2, 4.6, 1H, C_eH), 1.98 – 1.89 (m, 1H, C_cH), 1.89 – 1.71 (m, 3H, C_cH, C_dH_2); **^{13}C NMR** ($CDCl_3$, 101 MHz) δ 207.59 (C_bC(O)C_f), 156.81 (q, $^2J_{CF}$ = 37.5, CF_3C(O)NR_2), 116.09 (q, $^1J_{CF}$ = 288.1, CF_3), 57.52 (q, $^4J_{CF}$ = 2.3, C_a), 55.56 (C_a), 47.00 (C_f), 38.68 (C_b), 37.38 (C_e), 26.66 (C_c), 21.93 (C_d); **^{19}F NMR** ($CDCl_3$, 377 MHz) δ –72.68. See spectra

2-((Trifluoromethyl)sulfonyl)-2-azaspiro[3.5]nonan-5-one, 7i

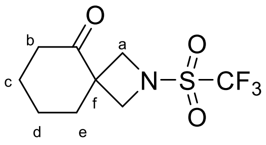

Prepared following general procedure 2b using the crude 1-(1-azabicyclo[1.1.0]butan-3-yl)cyclopentan-1-ol, **5i** (0.25 mmol). The crude reaction mixture was purified by flash column chromatography (98:2 → 80:20 pentane/ethyl acetate) to give **7i** (39 mg, 58%) as a white solid.

R_F 0.24 (80:20 petroleum ether/ethyl acetate); **IR** (film) ν_{max}/cm^{-1}: 2924, 2861, 1714, 1384, 1187, 1220; **HRMS** (ESI): Calcd. for $C_9H_{12}F_3NO_3SNa^+$ ($[M+Na]^+$) *m*/*z* 294.0382, found *m*/*z* 294.0390;

^{1}H NMR ($CDCl_3$, 400 MHz) δ 4.48 (d, $^2J_{HH}$ = 7.6, 2H, 2 × C_aH), 3.87 (d, $^2J_{HH}$ = 7.8, 2H, 2 × C_aH), 2.39 (t, $^3J_{HH}$ = 6.8, 2H, C_bH_2), 2.15 – 2.08 (m, 2H, C_eH_2), 1.87 (qd, $^3J_{HH}$ = 6.8, 3.1, 2H, C_cH_2), 1.79 – 1.65 (m, 2H, C_dH_2); **^{13}C NMR** ($CDCl_3$, 126 MHz) δ 206.94 (C_bC(O)C_f), 120.10 (q, $^1J_{CF}$ = 322.7, CF_3), 58.22 (2 × C_a), 46.04 (C_f), 38.87 (C_b), 37.29 (C_e), 26.75 (C_c), 21.99 (C_d); **^{19}F NMR** ($CDCl_3$, 377 MHz) δ -74.85. See spectra

2-(2,2,2-Trifluoroacetyl)-2-azaspiro[3.6]decan-5-one, 6j

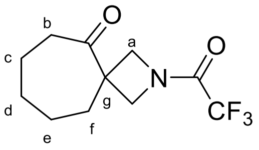

Prepared following general procedure 2a using the crude 1-(1-azabicyclo[1.1.0]butan-3-yl)cyclohexan-1-ol, **5j** (0.25 mmol). The crude reaction mixture was purified by flash column chromatography (95:5 → 60:40 pentane/ethyl acetate) to give **7j** (17 mg, 27%) as a colourless oil.

R_F 0.16 (80:20 petroleum ether/ethyl acetate); **IR** (film) ν_{max}/cm^{-1}: 2933, 2869, 1698, 1463, 1255, 1202, 1144; **HRMS** (ESI): Calcd. for $C_{11}H_{15}F_3NO_2^+$ ($[M+Na]^+$) *m/z* 250.1049, found *m/z* 250.1059;

^{1}H NMR ($CDCl_3$, 400 MHz) δ 4.64 (dt, $^2J_{HH}$ = 9.3, $^4J_{HH}$ = 1.3, 1H, C_aH), 4.27 (d, $^2J_{HH}$ = 10.3, 1H, C_aH), 4.02 (dt, $^2J_{HH}$ = 9.3, $^4J_{HH}$ = 1.3, 1H, C_aH), 3.87 (d, $^2J_{HH}$ = 10.3, 1H, C_aH), 2.70 – 2.51 (m, 2H, C_bH_2), 2.15 – 1.99 (m, 2H, C_fH_2), 1.74 – 1.54 (m, 6H, C_cH_2, C_dH_2, C_eH_2); **^{13}C NMR** ($CDCl_3$, 101 MHz) δ 211.58 ($C_bC(O)C_g$), 156.74 (q, $^2J_{CF}$ = 37.2, $CF_3C(O)NR_2$), 116. 18 (q, $^1J_{CF}$ = 287.4, CF_3), 59.75 (q, $^4J_{CF}$ = 2.2, C_a), 56.56 (C_a), 48.98 (C_g), 41.81 (C_b), 35.56 (C_f), 29.15 (C_c), 25.53 (C_e), 24.48 (C_d); **^{19}F NMR** ($CDCl_3$, 377 MHz) δ –72.53. See spectra

2-((Trifluoromethyl)sulfonyl)-2-azaspiro[3.6]decan-5-one, 7j

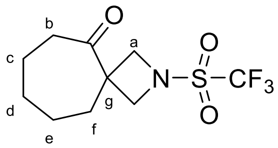

Prepared following general procedure 2b using the crude 1-(1-azabicyclo[1.1.0]butan-3-yl)cyclohexan-1-ol, **5j** (0.25 mmol). The crude reaction mixture was purified by flash column chromatography (95:5 → 60:40 pentane/ethyl acetate) to give **7j** (35 mg, 50%) as a white solid.

R_F 0.31 (80:20 petroleum ether/ethyl acetate); **IR** (film) ν_{max}/cm^{-1}: 2932, 2860, 1701, 1453, 1385, 1208; **HRMS** (ESI): Calcd. for $C_{10}H_{15}F_3NO_3S^+$ ($[M+H]^+$) *m/z* 286.0719, found *m/z* 286.0720;

^{1}H NMR ($CDCl_3$, 400 MHz) δ 4.43 (d, $^2J_{HH}$ = 7.7, 2H, 2 × C_aH), 3.89 (d, $^2J_{HH}$ = 7.7, 2H, 2 × C_aH), 2.60 (m, 2H, C_bH_2), 2.09 (m, 2H, C_fH_2), 1.66 – 1.58 (m, 6H, C_cH_2, C_dH_2, C_eH_2); **^{13}C NMR** ($CDCl_3$, 101 MHz) δ 210.84 ($C_bC(O)C_g$), 120.05 (q, $^1J_{CF}$ = 322.3, CF_3), 59.69 (2 × C_a), 47.97 (C_g), 41.69 (C_b), 35.27 (C_f), 29.04 (C_c), 25.42 (C_e), 24.43 (C_d); **^{19}F NMR** ($CDCl_3$, 377 MHz) δ -74.87. See spectra

2-(2,2,2-Trifluoroacetyl)-2-azaspiro[3.7]undecan-5-one, 6k

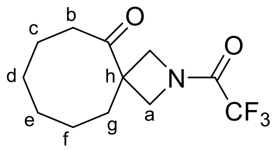

Prepared following general procedure 2a using the crude 1-(1-azabicyclo[1.1.0]butan-3-yl)cycloheptan-1-ol, **5k** (0.25 mmol). The crude reaction mixture was purified by flash column chromatography (95:5 → 70:30 pentane/ethyl acetate) to give **6k** (27 mg, 41%) as a white solid.

R_F 0.20 (80:20 petroleum ether/ethyl acetate); **IR** (film) ν_{max}/cm^{-1}: 2930, 2860, 1696, 1467, 1253, 1201, 1147; **HRMS** (ESI): Calcd. for $C_{12}H_{16}F_3NO_2Na^+$ ($[M+Na]^+$) *m/z* 286.1025, found *m/z* 286.1029;

^{1}H NMR ($CDCl_3$, 400 MHz) δ 4.64 (d, $^2J_{HH}$ = 9.4, 1H, C_aH), 4.24 (d, $^2J_{HH}$ = 10.4, 1H, C_aH), 4.04 (d, $^2J_{HH}$ = 9.4, 1H, C_aH), 3.85 (d, $^2J_{HH}$ = 10.4, 1H, C_aH), 2.54 (ddd, $^2J_{HH}$ = 12.4, $^3J_{HH}$ = 8.9, 4.4, 1H, C_bH), 2.45 (ddd, $^2J_{HH}$ = 12.4, $^3J_{HH}$ = 7.9, 4.4, 1H, C_bH), 2.33 (dt, $^2J_{HH}$ = 14.7, $^3J_{HH}$ = 6.1, 1H, C_gH), 2.23 (dt, $^2J_{HH}$ = 14.7, $^3J_{HH}$ = 5.6, 1H, C_gH), 1.86 – 1.77 (m, 2H, C_cH_2), 1.59 – 1.48 (m, 4H, C_fH_2, C_dH_2), 1.44 – 1.35 (m, 1H, C_eH), 1.26 – 1.15 (m, 1H C_eH); **^{13}C NMR** ($CDCl_3$, 126 MHz) δ 213.38 ($C_bC(O)C_h$), 156.84 (q, $^2J_{CF}$ = 37.5, $CF_3C(O)NR_2$), 116.10 (q, $^1J_{CF}$ = 288.1, CF_3), 57.38 (q, $^4J_{CF}$ = 2.3, C_a), 55.30 (C_a), 49.65 (C_h), 37.98 (C_b), 34.54 (C_g), 27.83 (C_c), 25.80 (C_d), 24.27 (C_e), 23.18 (C_f); **^{19}F NMR** ($CDCl_3$, 377 MHz) δ –72.63. See spectra

2-((Trifluoromethyl)sulfonyl)-2-azaspiro[3.7]undecan-5-one, 7k

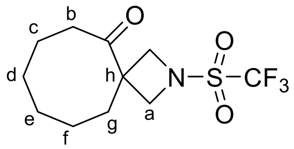

Prepared following general procedure 2b using the crude 1-(1-azabicyclo[1.1.0]butan-3-yl)cycloheptan-1-ol, **5k** (0.25 mmol). The crude reaction mixture was purified by flash column chromatography (100:0 → 90:10 pentane/acetone) to give **7k** (54 mg, 71%) as a white solid.

R_F 0.35 (80:20 petroleum ether/acetone); **IR** (film) ν_{max}/cm^{-1}: 2948, 2864, 1698, 1381, 1224, 1194; **HRMS** (ESI): Calcd. for $C_{11}H_{16}F_3NO_3SNa^+$ ($[M+Na]^+$) *m/z* 3220695, found *m/z* 322.0714;

^{1}H NMR (400 MHz, Chloroform-*d*) δ 4.41 (dd, $^2J_{HH}$ = 7.9, $^4J_{HH}$ = 1.0, 2H, 2 × C_aH), 3.90 (dd, $^2J_{HH}$ = 7.8, $^4J_{HH}$ = 1.0, 2H, 2 × C_aH), 2.48 (m, 2H, C_bH_2), 2.29 (m, 2H, C_gH_2), 1.80 (m, 2H, C_cH_2), 1.55 (m, 4H, C_fH_2, C_dH_2), 1.29 (m, 2H, C_eH_2); **^{13}C NMR** (101 MHz, Chloroform-*d*) δ 212.71 ($C_bC(O)C_h$), 120.00 (q, $^1J_{CF}$ = 322.7, CF_3), 58.02 (C_a), 47.60 (C_h), 38.07 (C_b), 34.53 (C_g), 27.51 (C_c), 25.85 (C_d), 24.25 (C_e), 22.93 (C_f); **^{19}F NMR** ($CDCl_3$, 377 MHz) δ -74.92. See spectra

tert-Butyl 8-oxo-2-(2,2,2-trifluoroacetyl)-2,6-diazaspiro[3.4]octane-6-carboxylate, 6l

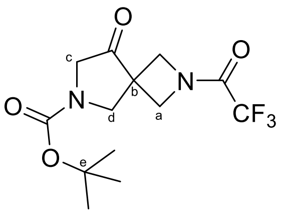

Prepared following general procedure 2a using the crude *tert*-butyl 3-(1-azabicyclo[1.1.0]butan-3-yl)-3-hydroxyazetidine-1-carboxylate, **5l** (0.25 mmol). The crude reaction mixture was purified by flash column chromatography (100:0 → 85:15 DCM/ethyl acetate) to give **6l** (38 mg, 48%) as a white solid.

R_F 0.23 (90:10 DCM/ethyl acetate); **IR** (film) ν_{max}/cm^{-1}: 2972, 1764, 1589, 1458, 1402, 1262, 1201, 1147; **HRMS** (APCI): Calcd. for $C_{13}H_{17}N_2O_4F_3Na^+$ ($[M+H]^+$) *m/z* 345.1033, found *m/z* 345.1042.

^{1}H NMR ($CDCl_3$, 400 MHz) δ 4.56 (d, $^2J_{HH}$ = 9.5, 1H, C_aH), 4.30 (d, $^2J_{HH}$ = 10.4, 2H, 2 × C_aH), 4.05 (d, $^2J_{HH}$ = 10.8, 1H, C_aH), 4.01 – 3.97 (m, C_dH_2), 3.90 (d, $^2J_{HH}$ = 20.2, C_cH), 3.85 (d, $^2J_{HH}$ = 20.2, C_cH), 1.47 (s, 9H, 3 × CH_3); **^{13}C NMR** ($CDCl_3$, 126 MHz) δ 209.08 (br., $C_cC(O)C_b$), 156.31 (q, $^1J_{CF}$ = 37.8, $R_2NC(O)R$), 115.91 (q, $^1J_{CF}$ = 288.5, CF_3), 81.41 (C_e), 58.64 (q, $^1J_{CF}$ = 2.5, C_a), 56. 20 (C_a), 52.86 (br., C_c), 52.10 (br., C_d), 44.99 (br., C_b), 28.39 ($C(CH_3)_3$); **^{19}F NMR** ($CDCl_3$, 377 MHz) δ –72.70. See spectra

tert-Butyl 8-oxo-2-((trifluoromethyl)sulfonyl)-2,6-diazaspiro[3.4]octane-6-carboxylate, 7l

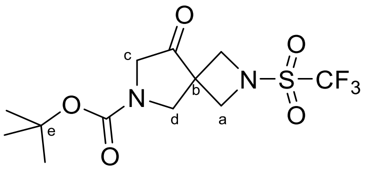

Prepared following general procedure 2b using the crude *tert*-butyl 3-(1-azabicyclo[1.1.0]butan-3-yl)-3-hydroxyazetidine-1-carboxylate, **5l** (0.25 mmol). The crude reaction mixture was purified by flash column chromatography (98:2 → 85:15 pentane/acetone) and then further purified by flash column chromatography (100:0 → 90:10 DCM/ethyl acetate) to give **7l** (56 mg, 63%) as a white solid.

R_F 0.50 (90:10 DCM/ethyl acetate); **IR** (film) ν_{max}/cm^{-1}: 2974, 1766, 1696, 1682, 1415, 1384, 1194; **HRMS** (ESI): Calcd. for $C_{12}H_{17}F_3N_2O_5SNa^+$ ($[M+Na]^+$) *m/z* 381.0702, found *m/z* 381.0709.

^{1}H NMR ($CDCl_3$, 400 MHz) δ 4.39 (d, $^2J_{HH}$ = 8.0, 2H, 2 × C_aH), 4.10 (d, $^2J_{HH}$ = 8.0, 2H, 2 × C_aH), 3.99 (s, 2H, C_cH_2), 3.86 (s, 2H, C_dH_2), 1.47 (s, 9H, 3 × CH_3); **^{13}C NMR** ($CDCl_3$, 101 MHz) δ 208.31 (br., $C_cC(O)C_b$), 153.98 ($R_2NC(O)R$) , 119.78 (q, $^1J_{CF}$ = 322.2, CF_3), 81.50

(C_e), 58.83 (2 × C_a), 52.97 (br., C_c), 52.03 (br., C_d), 43.92 (br., C_b), 28.36 (C(CH_3)$_3$); **^{19}F NMR** ($CDCl_3$, 377 MHz) δ –75.07. See spectra

1-(3-Acetyl-3-cyclohexylazetidin-1-yl)-2,2,2-trifluoroethan-1-one, 6m

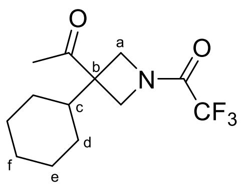

Prepared following general procedure 2a using the crude 1-(1-azabicyclo[1.1.0]butan-3-yl)-1-cyclohexylethan-1-ol, **5m** (0.25 mmol) with the following alteration: the reaction was stirred for 15 minutes at –78 °C then 30 minutes at 0 °C. The crude reaction mixture was purified by flash column chromatography (100:0 → 80:20 pentane/acetone) to give **6m** (18 mg, 26%) as a colourless oil.

R$_F$ 0.13 (90:10 pentane/acetone); **IR** (film) ν_{max}/cm^{-1}: 2929, 2856, 1697, 1469, 1359, 1248, 1202, 1149; **HRMS** (ESI): Calcd. for $C_{13}H_{18}NO_2F_3Na^+$ ([M+Na]$^+$) *m/z* 300.1182, found *m/z* 300.1194;

^{1}H NMR (400 MHz, Chloroform-*d*) δ 4.46 (d, $^2J_{HH}$ = 9.8, 1H, C_aH), 4.14 (d, $^2J_{HH}$ = 9.9, 1.2, 1H, C_aH), 4.11 (d, $^2J_{HH}$ = 11.5, 1H, C_aH), 4.00 (d, $^2J_{HH}$ = 10.9, 1H, C_aH), 2.10 (s, 3H, CH_3), 1.84 – 1.70 (m, 3H, C_cH, 2 × C_eH), 1.72 – 1.61 (m, 2H, C_fH, C_dH), 1.55 (m, 1H, C_dH), 1.28 – 1.04 (m, 3H, 2 × C_eH, C_fH), 0.94 (app. dqd, $^2J_{HH}$ = 20.9, $^3J_{HH}$ = 12.6, 3.5, 2H, 2 × C_dH); **^{13}C NMR** ($CDCl_3$, 126 MHz) δ 207.05 (CH_3C(O)C_b), 156.50 (q, $^2J_{CF}$ = 37.8, CF_3C(O)NR_2), 116.07 (q, $^1J_{CF}$ = 288.5, CF_3), 54.60 (q, $^4J_{CF}$ = 2.5, C_a), 52.26 (C_a), 41.38 (C_c), 27.41 (C_d), 27.07 (C_d), 26.25 (C_e), 26.21 (C_e), 26.00 (C_f), 25.75 (CH_3). See spectra

1-(3-Cyclohexyl-1-((trifluoromethyl)sulfonyl)azetidin-3-yl)ethan-1-one, 7m

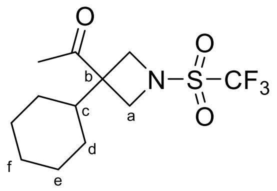

Prepared following general procedure 2b using the crude 1-(1-azabicyclo[1.1.0]butan-3-yl)-1-cyclohexylethan-1-ol, **5m** (0.25 mmol) with the following alteration: the reaction was stirred for 15 minutes at –78 °C then 30 minutes at 0 °C. The crude reaction mixture was purified by flash column chromatography (100:0 → 98:2 DCM/ethyl acetate) to give **7m** (76 mg, 97%) as a white solid.

R$_F$ 0.72 (98:2 DCM/ethyl acetate); **IR** (film) ν_{max}/cm^{-1}: 2933. 2857, 1702, 1379, 1209, 1197, 1177; **HRMS** (ESI): Calcd. for $C_{12}H_{18}F_3NO_3SNa^+$ ([M+Na]$^+$) *m/z* 336.0852, found *m/z* 336.0867;

^{1}H NMR ($CDCl_3$, 400 MHz) δ 4.31 (d, $^{2}J_{HH}$ = 8.3, 2H, 2 × C_aH), 4.08 (d, $^{2}J_{HH}$ = 8.3, 2H, 2 × C_aH), 2.15 (s, 3H, CH_3), 1.91 – 1.77 (m, 3H, C_cH, 2 × C_eH), 1.77 – 1.63 (m, 3H, C_fH, 2 × C_dH), 1.27 (app. qt, $^{2}J_{HH}$ = 12.5, $^{3}J_{HH}$ = 12.5, 3.2, 2H, 2 × C_eH), 1.16 (app. tt, $^{3}J_{HH}$ = 12.9, 3.1, 1H, C_fH), 1.02 (app. qd, $^{2}J_{HH}$ = 12.5, $^{3}J_{HH}$ = 12.5, 3.4, 2H, 2 × C_dH); **^{13}C NMR** ($CDCl_3$, 101 MHz) δ 206.43 ($CH_3C(O)C_b$), 119.90 (q, $^{1}J_{CF}$ = 322.3, CF_3), 55.12 (2 × C_a), 52.77 (C_b), 41.28 (C_c), 27.18 (2 × C_d), 26.23 (2 × C_e), 25.97 (C_f), 25.71 (CH_3); **^{19}F NMR** ($CDCl_3$, 377 MHz) δ –75.47. See spectra

3-Phenyl-1-(2,2,2-trifluoroacetyl)azetidine-3-carbaldehyde, 6n

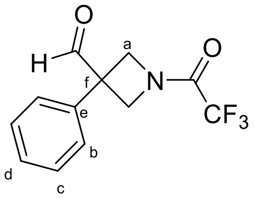

Prepared following general procedure 2a using the crude (1-azabicyclo[1.1.0]butan-3-yl)(phenyl)methanol, **5n** (0.25 mmol). The crude reaction mixture was purified by flash column chromatography with florisil (30 – 60 mesh) as the stationary phase (95:5 → 70:30 pentane/ethyl acetate) to give **6n** (33 mg, 51%) as a colourless oil.

R_F 0.33 (70:30 pentane/ethyl acetate); **IR** (film) ν_{max}/cm^{-1}: 2969, 2922, 1736, 1365, 1228, 1216, 1206; **HRMS** (APCI): Calcd. for $C_{12}H_{10}F_3NO_2^+$ ($[M+H]^+$) *m/z* 258.0736, found *m/z* 258.0729;

^{1}H NMR ($CDCl_3$, 400 MHz) δ 9.61 (s, 1H, RC(O)*H*), 7.52 – 7.44 (m, 2H, 2 × C_cH), 7.44 – 7.37 (m, 1H, C_dH), 7.20 – 7.14 (m, 2H, 2 × C_bH), 5.04 (dt, $^{2}J_{HH}$ = 9.5, $^{4}J_{HH}$ = 1.2, 1H, C_aH), 4.69 (dd, $^{2}J_{HH}$ = 10.5, $^{4}J_{HH}$ = 1.2, 1H, C_aH), 4.62 (d, $^{2}J_{HH}$ = 9.5, 1H, C_aH), 4.45 (d, $^{2}J_{HH}$ = 10.5, 1H, C_aH); **^{13}C NMR** ($CDCl_3$, 101 MHz) δ 194.80 ($HC(O)C_f$), 156.49 (q, $^{2}J_{CF}$ = 37.9, $CF_3C(O)NR_2$), 135.18 (C_e), 129.82 (2 × C_c), 128.88 (C_d), 126.54 (2 × C_b), 115.99 (q, $^{1}J_{CF}$ = 288.1, CF_3), 56.90 (q, $^{3}J_{CF}$ = 2.5, C_a), 54.44 (C_a), 52.66 (C_f); **^{19}F NMR** ($CDCl_3$, 377 MHz) δ –72.64. See spectra

(3-Phenyl-1-((trifluoromethyl)sulfonyl)azetidin-3-yl)methanol, 7n

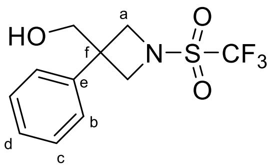

Prepared following general procedure 2b using the crude (1-azabicyclo[1.1.0]butan-3-yl)(phenyl)methanol, **5n** (0.25 mmol). Due to instability of the aldehyde on silica, the crude reaction mixture was oxidised prior to purification. The crude mixture was dissolved in anhydrous methanol (2.5 mL) under an inert atmosphere and cooled to 0 °C before the addition of sodium borohydride (19 mg, 0.5 mmol, 2.0 equiv). The reaction was stirred for 1 hour at 0 °C and a further 45 minutes at room temperature before the addition of water (10 mL) and ethyl acetate (10 mL). The phases were separated, and the aqueous phase was extracted with

diethyl ether (3 × 10 mL) and the combined organic phases were washed with brine, dried ($MgSO_4$), filtered and concentrated under reduced pressure. The crude reaction mixture was purified by flash column chromatography (95:5 → 60:40 pentane/ethyl acetate) to give **7n** (62 mg, 82%) as a colourless oil.

R_F 0.35 (70:30 pentane/ethyl acetate); **IR** (film) ν_{max}/cm^{-1}: 3391, 2969, 2871, 1382, 1219, 1193; **HRMS** (ESI): Calcd. for $C_{11}H_{12}F_3NO_3SNa^+$ ($[M+Na]^+$) *m/z* 318.0382, found *m/z* 318.0395;

^{1}H NMR ($CDCl_3$, 400 MHz) δ 7.42 (ddd, $^3J_{HH}$ = 7.8, 6.3, $^4J_{HH}$ = 1.4, 2H, 2 × C_cH), 7.37 – 7.31 (m, 1H, C_dH), 7.13 – 7.08 (m, 2H, 2 × C_bH), 4.47 (d, $^2J_{HH}$ = 8.1, 2H, 2 × C_aH), 4.42 (d, $^2J_{HH}$ = 7.7, 2H, 2 × C_aH), 3.87 (s, 2H, RCH_2OH), 1.82 (br. s, 1H, O*H*); **^{13}C NMR** ($CDCl_3$, 101 MHz) δ 140.66 (C_e), 129.17 (2 × C_c), 127.89 (C_d), 125.96 (2 × C_b), 119.97 (q, $^1J_{CF}$ = 322.4, CF_3) 68.82 (RCH_2OH), 59.40 (2 × C_a), 43.55 (C_f); **^{19}F NMR** ($CDCl_3$, 377 MHz) δ –75.26.

1-(3-Phenyl-1-(2,2,2-trifluoroacetyl)azetidin-3-yl)propan-1-one, 6o

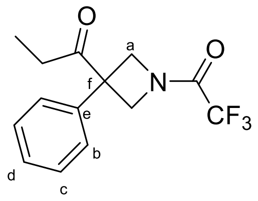

Prepared following general procedure 2a using the crude 1-(1-azabicyclo[1.1.0]butan-3-yl)-1-phenylpropan-1-ol, **5o** (0.25 mmol). The crude reaction mixture was purified by flash column chromatography (95:5 → 80:20 pentane/ethyl acetate) to give **6o** (63 mg, 88%) as a colourless oil.

R_F 0.12 (90:10 pentane/ethyl acetate); **IR** (film) ν_{max}/cm^{-1}: 2981, 2870, 1697, 1467, 1203, 1145; **HRMS** (ESI): Calcd. for $C_{14}H_{14}F_3NO_2Na^+$ ($[M+Na]^+$) *m/z* 308.0869, found *m/z* 308.0870;

^{1}H NMR ($CDCl_3$, 400 MHz) δ 7.45 – 7.39 (m, 2H, 2 × C_cH), 7.38 – 7.32 (m, 1H, C_dH), 7.22 – 7.17 (m, 2H, 2 × C_bH), 5.11 (d, $^2J_{HH}$ = 9.5, 1H, C_aH), 4.65 (d, $^2J_{HH}$ = 10.6, 1H, C_aH), 4.58 (d, $^2J_{HH}$ = 9.6, 1H, C_aH), 4.54 (d, $^2J_{HH}$ = 10.6, 1H, C_aH), 2.44 – 2.20 (m, 2H, CH_2CH_3), 0.96 (t, $^3J_{HH}$ = 7.2, 3H, CH_3); **^{13}C NMR** ($CDCl_3$, 101 MHz) δ 206.85 (Et*C*(O)C_f), 156.35 (q, $^2J_{CF}$ = 37.8, $CF_3C(O)NR_2$), 138.13 (C_e), 129.51 (2 × C_c), 128.38 (C_d), 126.18 (2 × C_b), 116.02 (q, $^1J_{CF}$ = 288.4, CF_3), 59.00 (q, $^4J_{CF}$ = 2.6, C_a), 55.83 (C_a), 53.83 (C_f), 30.30 (CH_3), 8.07 (CH_2CH_3); **^{19}F NMR** ($CDCl_3$, 377 MHz) δ –72.56. See spectra

1-(3-Phenyl-1-((trifluoromethyl)sulfonyl)azetidin-3-yl)propan-1-one, 7o

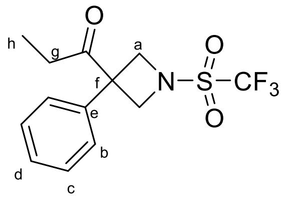

Prepared following general procedure 2b using the crude 1-(1-azabicyclo[1.1.0]butan-3-yl)-1-phenylpropan-1-ol, **5o** (0.25 mmol). The crude reaction mixture was purified by flash column chromatography (100:0 → 85:15 pentane/ethyl acetate) to give **7o** (50 mg, 62%) as a colourless oil.

R_F 0.28 (90:10 pentane/ethyl acetate); **IR** (film) ν_{max}/cm^{-1}: 2981, 2870, 1716, 1387, 1192, 1225, 1142; **HRMS** (ESI): Calcd. for $C_{13}H_{14}F_3NO_3SNa^+$ ($[M+Na]^+$) *m*/*z* 344.0539, found *m*/*z* 344.0537;

^{1}H NMR ($CDCl_3$, 400 MHz) δ 7.48 – 7.41 (m, 2H, 2 × C_cH), 7.40 – 7.34 (m, 1H, C_dH), 7.20 – 7.14 (m, 2H, 2 × C_bH), 4.81 (d, $^2J_{HH}$ = 8.0, 2H, 2 × C_aH), 4.53 (d, $^2J_{HH}$ = 8.0, 2H, 2 × C_aH), 2.32 (q, $^3J_{HH}$ = 7.3, 2H, CH_2CH_3), 0.98 (t, *J* = 7.2, 3H, CH_3); **^{13}C NMR** ($CDCl_3$, 101 MHz) δ 206.32 (Et*C*(O)C_f), 137.71 (C_e), 129.66 (2 × C_c), 128.61 (C_d), 126.03 (2 × C_b), 119.89 (q, $^1J_{CF}$ = 322.4, CF_3), 58.98 (C_a), 52.81 (C_f), 30.37 (CH_2CH_3), 8.16 (CH_3); **^{19}F NMR** ($CDCl_3$, 377 MHz) δ –75.14. See spectra

3-Phenyl-1-(3-phenyl-1-(2,2,2-trifluoroacetyl)azetidin-3-yl)prop-2-yn-1-one, 6p

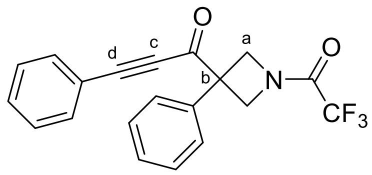

Prepared following general procedure 2a using the crude 1-(1-azabicyclo[1.1.0]butan-3-yl)-1,3-diphenylprop-2-yn-1-ol, **5p** (0.25 mmol). The crude reaction mixture was purified by flash column chromatography (100:0 → 80:20 pentane/ethyl acetate) to give **6p** (81 mg, 91%) as a yellow solid.

R_F 0.55 (80:20 pentane/ethyl acetate); **IR** (film) ν_{max}/cm^{-1}: 3062, 2955, 2884, 2194, 1698, 1668, 1466, 1264, 1244; **HRMS** (ESI): Calcd. for $C_{20}H_{15}F_3NO_2^+$ ($[M+H]^+$) *m*/*z* 380.0869, found *m*/*z* 380.0878;

^{1}H NMR ($CDCl_3$, 400 MHz) δ 7.51 – 7.43 (m, 5H, ArC*H*), 7.42 – 7.36 (m, 3H, ArC*H*), 7.33 – 7.29 (m, 2H, ArC*H*), 5.21 (dt, $^2J_{HH}$ = 9.7, $^4J_{HH}$ = 1.3, 1H, C_aH), 4.92 (dd, $^2J_{HH}$ = 10.7, $^4J_{HH}$ = 1.1, 1H, C_aH), 4.75 (dq, $^2J_{HH}$ = 9.8, $^4J_{HH}$ = 1.0, 1H, C_aH), 4.61 (d, $^2J_{HH}$ = 10.7, 1H, C_aH); **^{13}C NMR** ($CDCl_3$, 101 MHz) δ 183.97 (C_c*C*(O)C_b), 156.50 (q, $^2J_{CF}$ = 37.8, CF_3*C*(O)NR_2), 137.17 (Ar*C*), 133.42 (Ar*C*H), 131.72 (Ar*C*H), 129.47 (Ar*C*H), 129.00 (Ar*C*H), 128.83

(Ar*C*H), 126.99 (Ar*C*H), 119.21 (Ar*C*), 116.17 (q, $^{1}J_{CF}$ = 288.0, CF_3), 97.33 (C_d), 84.91 (C_c), 59.22 (q, $^{1}J_{CF}$ = 2.0, C_a), 56.60 (C_a), 54.82 (C_b); **^{19}F NMR** ($CDCl_3$, 377 MHz) δ –72.59. See spectra

3-Phenyl-1-(3-phenyl-1-((trifluoromethyl)sulfonyl)azetidin-3-yl)prop-2-yn-1-one, 7p

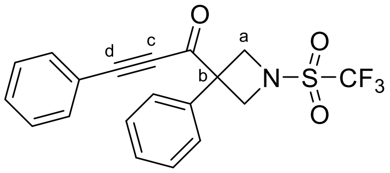

Prepared following general procedure 2b using the crude 1-(1-azabicyclo[1.1.0]butan-3-yl)-1,3-diphenylprop-2-yn-1-ol, **5p** (0.25 mmol). The crude reaction mixture was purified by flash column chromatography (100:0 → 80:20 pentane/ethyl acetate) to give **7p** (88 mg, 89%) as a yellow solid.

R$_F$ 0.40 (90:10 pentane/ethyl acetate); **IR** (film) ν_{max}/cm^{-1}: 2965, 2870, 2199, 1668, 1383, 1229, 1198; **HRMS** (ESI): Calcd. for $C_{19}H_{14}F_3NO_3SNa^+$ ($[M+Na]^+$) *m*/*z* 416.0539, found *m*/*z* 416.0527;

^{1}H NMR ($CDCl_3$, 400 MHz) δ 7.52 – 7.43 (m, 5H, ArC*H*)), 7.43 – 7.35 (m, 3H, ArC*H*)), 7.31 – 7.25 (m, 2H, ArC*H*)), 4.98 (d, $^{2}J_{HH}$ = 8.1, 2H, 2 × C_aH), 4.65 (d, $^{2}J_{HH}$ = 8.1, 2H, 2 × C_aH); **^{13}C NMR** ($CDCl_3$, 101 MHz) δ 183.24 (C_c*C*(O)C_b), 136.64 (Ar*C*), 133.34 (Ar*C*H), 131.69 (Ar*C*H), 129.46 (Ar*C*H), 128.92 (Ar*C*H), 128.89 (Ar*C*H), 126.66 (Ar*C*H), 119.86 (q, $^{1}J_{CF}$ = 322.2, CF_3), 119.05 (Ar*C*), 97.34 (C_d), 84.71 (C_c), 59.25 (2 × C_a), 53.62 (C_b); **^{19}F NMR** ($CDCl_3$, 377 MHz) δ –75.29. See spectra

1-(3-(Cyclohexanecarbonyl)-3-phenylazetidin-1-yl)-2,2,2-trifluoroethan-1-one, 6q

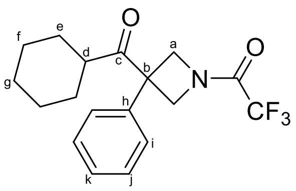

Prepared following general procedure 2a using the crude (1-azabicyclo[1.1.0]butan-3-yl)(cyclohexyl)(phenyl)methanol, **5q** (0.25 mmol). The crude reaction mixture was purified by flash column chromatography (98:2 → 80:20 DCM/ethyl acetate) to give **6q** (22 mg, 26%) as a colourless oil.

R$_F$ 0.42 (90:10 DCM/ethyl acetate); **IR** (film) ν_{max}/cm^{-1}: 2933, 2858, 1698, 1467, 1449, 1238, 1203, 1146; **HRMS** (ESI): Calcd. for $C_{18}H_{20}F_3NO_2Na^+$ ($[M+Na]^+$) *m*/*z* 362.1338, found *m*/*z* 362.1349;

^{1}**H NMR** ($CDCl_3$, 400 MHz) δ 7.47 – 7.39 (m, 2H, 2 × C_jH), 7.39 – 7.33 (m, 1H, C_kH), 7.24 – 7.19 (m, 2H, C_iH), 5.13 (dt, $^2J_{HH}$ = 9.5, $^4J_{HH}$ = 1.3, 1H, C_aH), 4.66 (d, $^2J_{HH}$ = 10.7, 1H, C_aH), 4.55 (dt, $^2J_{HH}$ = 9.4, $^4J_{HH}$ = 1.2, 2H, C_aH), 2.46 (tt, $^3J_{HH}$ = 10.1, $^3J_{HH}$ = 3.3, 1H, C_dH), 1.73 – 1.63 (m, 2H, 2 × C_fH), 1.63 – 1.52 (m, 2H, 2 × C_fH), 1.39 (m, 2H, 2 × C_eH), 1.30 – 1.20 (m, 1H, C_eH), 1.09 (m, 3H, C_eH, C_gH_2); ^{13}**C NMR** ($CDCl_3$, 101 MHz) δ 208.95 (C_c), 156.49 (d, $^2J_{CF}$ = 37.6, $R_2NC(O)CF_3$), 137.40 (C_h), 129.51 (2 × C_i), 128.49 (C_k), 126.38 (2 × C_j), 116.08 (d, $^1J_{CF}$ = 288.1, CF_3), 58.83 (C_a), 55.32 (C_a), 54.20 (C_b), 46.70 (C_d), 30.32 (C_e), 30.24 (C_e), 25.48 (2 × C_f, C_g); ^{19}**F NMR** ($CDCl_3$, 377 MHz) δ –72.52. See spectra

Semipinacol rearrangement of 5q with Tf_2O

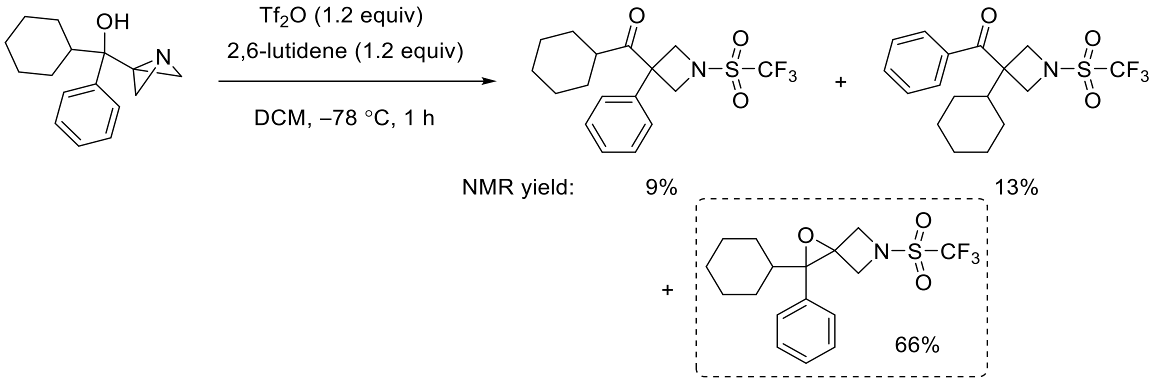

Following general procedure 2b using the crude (1-azabicyclo[1.1.0]butan-3-yl)(cyclohexyl)(phenyl)methanol, **5q** (0.25 mmol). The crude reaction mixture was purified by flash column chromatography (100:0 → 90:10 DCM/ethyl acetate), mixed fractions were further purified by flash column chromatography (99:1 → 60:40 pentane/DCM).

2-Cyclohexyl-2-phenyl-5-((trifluoromethyl)sulfonyl)-1-oxa-5-azaspiro[2.3]hexane, 13

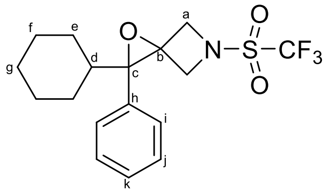

Mixed fractions were discarded and **13** was isolated as a white solid (30 mg, 32%).

R$_F$ 0.51 (50:50 pentane/DCM); **IR** (film) ν_{max}/cm^{-1}: 2930, 2855, 1446, 1377, 1226, 1195; **HRMS** (ESI): Calcd. for $C_{17}H_{20}F_3NO_3SNa^+$ ($[M+Na]^+$) *m/z* 398.1021, found *m/z* 398.1023;

^{1}**H NMR** ($CDCl_3$, 400 MHz) δ 7.39 – 7.29 (m, 3H, C_iH, C_kH), 7.27 – 7.21 (m, 2H, C_jH), 4.66 (d, $^2J_{HH}$ = 10.0, 1H, C_aH), 4.56 (d, $^2J_{HH}$ = 10.0, 1H, C_aH), 4.14 (d, $^2J_{HH}$ = 10.2, 1H, C_aH), 4.08 (d, $^2J_{HH}$ = 10.2, 1H, C_aH), 1.91 – 1.67 (m, 4H, 2 × C_eH, C_fH, C_gH), 1.67 – 1.53 (m, 1H, C_gH), 1.33 – 1.10 (m, 4H, C_eH, 2 × C_fH, C_dH), 1.00 – 1.69 (m, C_eH, C_fH); ^{13}**C NMR** ($CDCl_3$,

101 MHz) δ 134.43 (C_h), 128.38 (C_k), 128.34 (2 × C_i), 127.54 (2 × C_j), 120.04 (d, $^1J_{HH}$ = 322.6), 69.70 (C_c), 63.48 (C_b), 59.64 (C_a), 59.31 (C_a), 43.63 (C_d), 29.43 (C_e), 27.78 (C_e), 26.19 (C_f), 25.92 (C_f), 25.74 (C_g); **^{19}F NMR** ($CDCl_3$, 377 MHz) δ -74.82. See spectra

Cyclohexyl(3-phenyl-1-((trifluoromethyl)sulfonyl)azetidin-3-yl)methanone, 7q and (3-Cyclohexyl-1-((trifluoromethyl)sulfonyl)azetidin-3-yl)(phenyl)methanone, 7q'

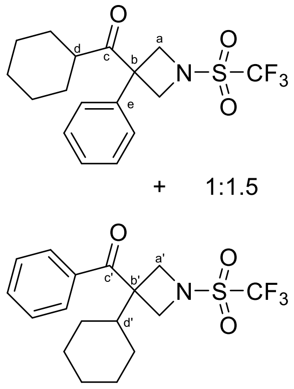

Compounds **7q** and **7q'** were isolated as a mixture in a 1:1.5 ratio (22 mg, 23%).

R$_F$ 0.33 and 0.29 (95:5 DCM/ethyl acetate); **IR** (film) ν_{max}/cm^{-1}: 2932, 2855, 1710, 1678, 1448, 1385, 1223, 1192; **HRMS** (ESI): Calcd. for $C_{17}H_{20}F_3NO_3SNa^+$ ([M+Na]$^+$) *m/z* 398.1021, found *m/z* 398.1008;

^{1}H NMR ($CDCl_3$, 400 MHz) δ 7.73 – 7.67 (m, ArC*H*), 7.64 – 7.58 (m, ArC*H*), 7.53 – 7.46 (m, ArC*H*), 7.46 – 7.40 (m, ArC*H*), 7.40 – 7.34 (m, ArC*H*), 7.20 – 7.13 (m, ArC*H*), 4.83 (d, $^2J_{HH}$ = 8.0, 2H, 2 × C$_a$*H*), 4.62 (d, $^2J_{HH}$ = 8.2, 2H, 2 × C$_{a'}$*H*), 4.52 (d, $^2J_{HH}$ = 8.0, 2H, 2 × C$_a$*H*), 4.35 (d, $^2J_{HH}$ = 8.3, 2H, 2 × C$_{a'}$*H*), 2.44 (tt, $^3J_{HH}$ = 11.4, 3.6, 1H, C$_d$*H*), 2.15 – 2.02 (m, 1H, C$_{d'}$*H*), 1.92 – 1.53 (m, C$_{alkyl}$*H*), 1.41 – 0.98 (m, C$_{alkyl}$*H*); **^{13}C NMR** ($CDCl_3$, 101 MHz) δ 208.33 (C_c), 198.73 ($C_{c'}$), 136.87 (C_e), 133.99 (Ar*C*), 132.82 (Ar*C*), 129.55 (Ar*C*), 129.15 (Ar*C*), 129.09 (Ar*C*), 128.60 (Ar*C*), 126.13 (Ar*C*), 119.90 (q, $^1J_{CF}$ = 322.6, CF_3), 119.83 (q, $^1J_{CF}$ = 321.9, CF_3), 58.59 (2 ×C_a), 55.74 (2 ×$C_{a'}$), 53.16 (C_b), 51.45 ($C_{b'}$), 46.71 (C_d), 41.71 ($C_{d'}$), 30.25 (C_{alkyl}), 26.85 (C_{alkyl}), 26.11 (C_{alkyl}), 25.88 (C_{alkyl}), 25.45 (C_{alkyl}), 25.42 (C_{alkyl}); **^{19}F NMR** ($CDCl_3$, 377 MHz) δ –75.01, –75.61. See spectra

1,1'-(3-Phenylazetidine-1,3-diyl)bis(2,2,2-trifluoroethan-1-one), 6r

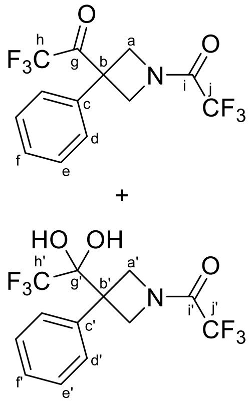

Prepared following general procedure 2a using the purified 1-(1-azabicyclo[1.1.0]butan-3-yl)-2,2,2-trifluoro-1-phenylethan-1-ol, **5r** (0.25 mmol) with the following alteration: the reaction was stirred for 15 minutes at –78 °C then 30 minutes at 0 °C. The crude reaction mixture was purified by flash column chromatography (95:5 → 60:40 pentane/ethyl acetate) to give **6r** (20 mg, 25%) as a white solid. In solution it exists as a mixture of the ketone and the hydrate.

R$_F$ 0.24 (70:30 pentane/ethyl acetate); **IR** (film) ν_{max}/cm^{-1}: 3380, 2963, 1754, 1697, 1458, 1260, 1204, 1151; **HRMS** (APCI): Calcd. for $C_{13}H_9F_6NO_2^+$ ([M+H]$^+$)

m/z 326.0610, found *m/z* 326.0600; Calcd. for $C_{13}H_{11}F_6NO_3^+$ (hydrate $[M+H]^+$) *m/z* 344.0716, found *m/z* 344.0701;

^{1}H NMR ($CDCl_3$, 400 MHz) δ 7.52 – 7.37 (m, 3H, 2 × C_dH, C_fH), 7.31 – 7.26 (m, 2H, 2 × C_eH), 5.18 (d, $^2J_{HH}$ = 10.1, 1H, C_aH), 5.08 (d, $^2J_{HH}$ = 9.9, 1H, $C_{a'}H$), 4.84 (d, $^2J_{HH}$ = 11.2, 1H, C_aH), 4.78 (d, $^2J_{HH}$ = 10.1, 1H, C_aH), 4.70 (d, $^2J_{HH}$ = 9.9, 1H, $C_{a'}H$), 4.67 (d, $^2J_{HH}$ = 11.2, 1H, C_aH), 4.41 (d, $^2J_{HH}$ = 10.7, 1H, C_aH), 4.08 (s, 2H, 2 × O*H*); **^{13}C NMR** (CD_2Cl_2, 126 MHz) δ 188.46 (q, $^2J_{CF}$ = 33.7, C_g), 156.47 (q, $^2J_{CF}$ = 37.9, C_i), 156.05 (q, $^2J_{CF}$ = 36.8, $C_{i'}$), 138.40 ($C_{c'}$), 133.94 (C_c), 130.11 (2 × C_d), 129.75 (C_f), 128.88 (2 × $C_{e'}$), 128.59 (2 × $C_{d'}$), 128.44 (C_f), 127.14 (2 × C_e), 125.81 (q, $^1J_{CF}$ = 268.9, $C_{h'}$), 116.55 (q, $^1J_{CF}$ = 287.7, $C_{j'}$), 116.27 (q, $^1J_{CF}$ = 290.0, C_h), 116.08 (q, $^1J_{CF}$ = 293.7, C_j), 95.38 (q, $^2J_{CF}$ = 30.5, $C_{g'}$), 59.71 ($C_{a'}$), 58.89 (C_a), 57.00 ($C_{a'}$), 55.51 (C_a), 51.60 (C_b), 48.09 ($C_{b'}$). See spectra

2,2,2-Trifluoro-1-(3-phenyl-1-((trifluoromethyl)sulfonyl)azetidin-3-yl)ethan-1-one, 7r

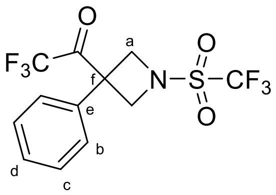

Prepared following general procedure 2b using the purified 1-(1-azabicyclo[1.1.0]butan-3-yl)-2,2,2-trifluoro-1-phenylethan-1-ol, **5r** (0.25 mmol) with the following alteration: the reaction was stirred for 15 minutes at –78 °C then 30 minutes at 0 °C. The crude reaction mixture was purified by flash column chromatography (100:0 → 70:30 pentane/acetone) to give **7r** (65 mg, 72%) as a white solid. In solution it exists as a mixture of the ketone and the hydrate.

R_F 0.22 (80:20 pentane/acetone); **IR** (film) ν_{max}/cm^{-1}: 2982, 1750, 1382, 1229, 1190, 1160; **HRMS** (APCI): Calcd. for $C_{12}H_9F_6NO_3S^+$ ($[M+H]^+$) *m/z* 362.0280, found *m/z* 362.0273;

^{1}H NMR ($CDCl_3$, 400 MHz) δ 7.53 – 7.41 (m, 3H, 2 × C_cH, C_dH), 7.25 – 7.19 (m, 2H, C_bH), 4.91 (d, $^2J_{HH}$ = 8.7, 2H, 2 × C_aH), 4.66 (d, $^2J_{HH}$ = 8.7, 1H, 2 × C_aH); **^{13}C NMR** ($CDCl_3$, 101 MHz) δ 187.84 (q, $^2J_{HH}$ = 34.2, $CF_3C(O)C_f$), 133.33 (C_e), 130.13 (2 × C_c), 129.82 (C_d), 126.58 (2 × C_b), 119.85 (d, $^1J_{CF}$ = 321.8, RSO_2CF_3), 115.82 (d, $^1J_{CF}$ = 293.6, RC(O)CF_3), 58.37 (d, $^3J_{CF}$ = 1.7, C_a), 50.41 (C_f); **^{19}F NMR** ($CDCl_3$, 377 MHz) δ -71.33, –75.43. See spectra

1-(3-benzoyl-3-phenylazetidin-1-yl)-2,2,2-trifluoroethan-1-one, 6s

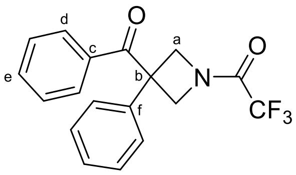

Prepared following general procedure 2a using the crude (1-azabicyclo[1.1.0]butan-3-yl)diphenylmethanol, **5s** (0.25 mmol). The crude reaction mixture was purified by flash column chromatography (100:0 → 90:10 toluene/acetone) to give **6s** (35 mg, 42%) as a colourless oil.

R$_F$ 0.53 (90:10 toluene/acetone); **IR** (film) ν_{max}/cm^{-1}: 2969, 2870, 1696, 1681, 1448, 1469, 1255, 1229, 1204, 1145; **HRMS** (ESI): Calcd. for $C_{18}H_{15}F_3NO_2^+$ ($[M+H]^+$) *m/z* 334.1049, found *m/z* 334.1050;

^{1}H NMR ($CDCl_3$, 400 MHz) δ 7.67 (dd, $^3J_{HH}$ = 8.5, $^4J_{HH}$ =1.3, 2H, C_dH), 7.51 (tt, $^3J_{HH}$ = 7.4, $^4J_{HH}$ =1.3, 1H, C_eH), 7.44 – 7.30 (m, 7H, ArC*H*), 5.22 (d, $^2J_{HH}$ = 9.9, 1H, C_aH), 4.81 (d, $^2J_{HH}$ = 11.1, 1H, C_aH), 4.70 (d, $^2J_{HH}$ = 11.1, 1H, C_aH), 4.64 (dt, $^2J_{HH}$ = 9.9, 1.2, 1H, C_aH); **^{13}C NMR** ($CDCl_3$, 101 MHz) δ 196.99 (Ar*C*(O)C_b), 156.61 (q, $^2J_{CF}$ = 37.8, CF_3C(O)NR_2), 139.17 (C_c), 133.86 (Ar*C*H), 132.67 (C_f), 129.82 (Ar*C*H), 129.76 (Ar*C*H), 128.98 (Ar*C*H), 128.41 (Ar*C*H), 125.55 (Ar*C*H), 116.09 (q, $^1J_{CF}$ = 288.0, CF_3), 60.96 (C_a), 57.28 (C_a), 52.12 (C_b); **^{19}F NMR** ($CDCl_3$, 377 MHz) δ –72.51. See spectra

Phenyl(3-phenyl-1-((trifluoromethyl)sulfonyl)azetidin-3-yl)methanone, 7s

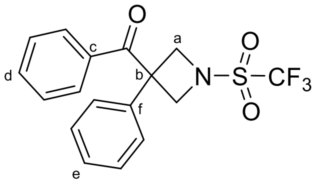

Prepared following general procedure 2b using the crude (1-azabicyclo[1.1.0]butan-3-yl)diphenylmethanol, **5s** (0.25 mmol). The crude reaction mixture was purified by flash column chromatography (100:0 → 90:10 toluene/acetone) to give **7s** (83 mg, 90%) as a white solid.

R$_F$ 0.41 (80:20 petroleum ether/ethyl acetate); **IR** (film) ν_{max}/cm^{-1}: 2981, 2869, 1673, 1384, 1227, 1197; **HRMS** (ESI): Calcd. for $C_{17}H_{14}F_3NO_3SNa^+$ ($[M+Na]^+$) *m/z* 392.0539, found *m/z* 392.0544;

^{1}H NMR (400 MHz, Chloroform-*d*) δ 7.61 – 7.51 (m, 2H, C_dH), 7.47 – 7.38 (m, 1H, C_dH), 7.37 – 7.23 (m, 7H, ArC*H*), 4.88 (d, $^2J_{HH}$ = 8.2, 2H, 2 × C_aH), 4.49 (d, $^2J_{HH}$ = 8.2, 2H, 2 × C_aH); **^{13}C NMR** (101 MHz, $CDCl_3$) δ 196.54 (Ar*C*(O)C_b), 138.75 (C_c), 133.95 (Ar*C*H), 132.59 (C_f), 129.90 (Ar*C*H), 129.71 (Ar*C*H), 129.03 (Ar*C*H), 128.57 (Ar*C*H), 125.46 (Ar*C*H), 119.92 (q,

$^1J_{CF}$ = 322.6, CF_3) 60.56 (2 × C_a), 50.91 (C_b); **^{19}F NMR** ($CDCl_3$, 377 MHz) δ –75.21. See spectra

1-(3-Benzoyl-3-(4-methoxyphenyl)azetidin-1-yl)-2,2,2-trifluoroethan-1-one, 6t and 2,2,2-trifluoro-1-(3-(4-methoxybenzoyl)-3-phenylazetidin-1-yl)ethan-1-one, 6t'

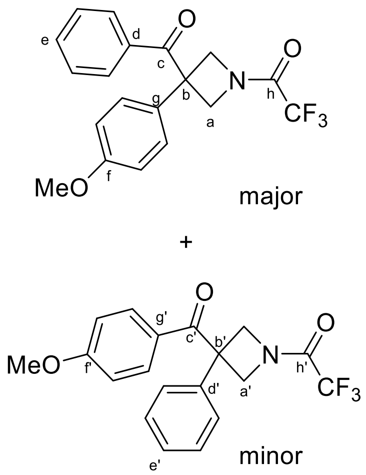

Prepared following general procedure 2a using the crude (1-azabicyclo[1.1.0]butan-3-yl)(4-methoxyphenyl)(phenyl)methanol, **5t** (0.25 mmol). The crude reaction mixture was purified by flash column chromatography (100:0 → 90:10 pentane/acetone) and further purified by flash column chromatography (90:10 DCM/ethyl actetate) to give a mixture of **6t** and **6t'** in a 1:0.3 ratio respectively (36 mg, 42%).

R_F 0.38 (80:20 pentane/acetone); **IR** (film) ν_{max}/cm^{-1}: 2981, 2870, 1698, 1682, 1514, 1467, 1251, 1146; **HRMS** (ESI): Calcd. for $C_{19}H_{17}F_3NO_3^+$ ([M+H]$^+$) m/z 364.1155, found m/z 364.1161;

^{1}H NMR ($CDCl_3$, 500 MHz) δ 7.67 (dd, $^3J_{HH}$ = 8.4, $^4J_{HH}$ = 1.2, 2H, 2 × ArCH), 7.64 (d, $^3J_{HH}$ = 9.0, 2H, 2 × ArCH'), 7.51 (tt, $^3J_{HH}$ = 7.0, $^4J_{HH}$ = 1.2, 1H, C_eH), 7.38 (m, 2H, 2 × ArCH), 7.29 (d, $^3J_{HH}$ = 8.9, 2H, 2 × ArCH), 6.92 (d, $^3J_{HH}$ = 8.9, 2H, 2 × ArCH), 6.84 (d, $^3J_{HH}$ = 9.0, 2H, 2 × ArCH'), 5.22 (d, $^2J_{HH}$ = 9.8, 1H, $C_{a'}H$), 5.20 (d, J = 9.9, 1H, C_aH), 4.80 (d, $^2J_{HH}$ = 11.1, 1H, $C_{a'}H$), 4.78 (d, J = 11.0, 1H, C_aH), 4.68 (d, $^2J_{HH}$ = 11.1, 1H, $C_{a'}H$), 4.65 (d, J = 10.9, 1H, C_aH), 4.60 (d, $^2J_{HH}$ = 9.8, 1H, $C_{a'}H$), 4.59 (d, J = 9.9, 1H, C_aH), 3.82 (OCH_3'), 3.79 (s, 3H, OCH_3); **^{13}C NMR** ($CDCl_3$, 101 MHz) δ 197.14 (C_c), 195.53 ($C_{c'}$), 163.90 ($C_{f'}$), 159.45 (C_f), 156.56 (q, $^2J_{CF}$ = 37.7, C_h), 156.55 (q, $^2J_{CF}$ = 37.8, $C_{h'}$), 139.58 ($C_{g'}$), 133.74 (C_e), 132.69 (C_d), 132.10 (ArCH'), 131.01 (C_g), 129.73 (ArCH), 129.69 (ArCH'), 128.91 (ArCH), 128.24 ($C_{e'}$), 126.80 (ArCH), 125.46 (ArCH'), 115.70 (q, $^1J_{CF}$ = 289.0, CF_3) 115.09 (ArCH), 114.18 (ArCH'), 61.11 (q, $^4J_{CF}$ = 2.2, C_a), 61.06 ($C_{a'}$), 57.43 (C_a), 57.41 ($C_{a'}$), 55.61 (OCH$_3$'), 55.42 (OCH$_3$), 51.82 ($C_{b'}$), 51.60 (C_b); **^{19}F NMR** ($CDCl_3$, 377 MHz) δ –72.50. See spectra

(3-(4-Methoxyphenyl)-1-((trifluoromethyl)sulfonyl)azetidin-3-yl)(phenyl)methanone, 7t and (4-methoxyphenyl)(3-phenyl-1-((trifluoromethyl)sulfonyl)azetidin-3-yl)methanone 7t'

Prepared following general procedure 2b using the crude (1-azabicyclo[1.1.0]butan-3-yl)(4-methoxyphenyl)(phenyl)methanol, **5t** (0.25 mmol). The crude reaction mixture was purified by flash column chromatography (100:0 → 90:10 pentane/acetone) and further purified by flash column chromatography (90:10 DCM/ethyl actetate) to give a mixture of **7t** and **7t'** in a 1:0.8 ratio respectively (88 mg, 80%).

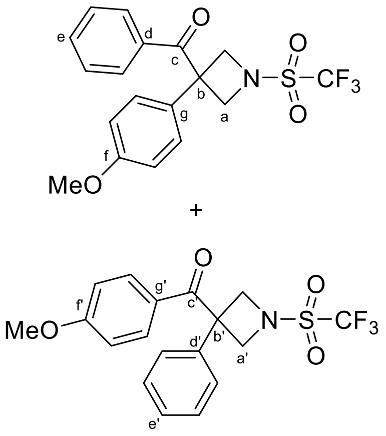

R$_F$ 0.15 (90:10 pentane/acetone); **IR** (film) ν_{max}/cm^{-1}: 2968, 2906, 1676, 1599, 1513, 1386, 1257, 1224, 1203; **HRMS** (ESI): Calcd. for $C_{18}H_{17}F_3NO_4S^+$ ([M+H]$^+$) *m/z* 400.0825, found *m/z* 400.0840;

^{1}H NMR ($CDCl_3$, 400 MHz) δ 7.63 (dd, $^3J_{HH}$ = 8.3, $^4J_{HH}$ = 1.4, 2H, ArC*H*), 7.59 (d, $^3J_{HH}$ = 8.9, 2H, ArC*H*), 7.51 (t, $^3J_{HH}$ = 7.6, 1H, C$_e$*H*), 7.43 – 7.24 (m, 9H, ArC*H*), 6.93 (d, $^3J_{HH}$ = 8.9, 2H, ArC*H*), 6.83 (dd, $^3J_{HH}$ = 9.0, $^4J_{HH}$ = 2.4, 2H, ArC*H*), 4.94 (m, 4H, C$_a$*H*, C$_a$*H*'), 4.53 (m, 4H, C$_a$*H*, C$_a$*H*'), 3.82 (s, 3H, OC*H$_3$*), 3.80 (s, 3H, OC*H$_3$*); **^{13}C NMR** ($CDCl_3$, 101 MHz) δ 196.72 (*C$_c$*), 195.09 (*C$_{c'}$*), 164.00 (*C$_{f'}$*), 159.60 (*C$_f$*), 139.18 (*C$_{g'}$*), 133.84 (*C$_e$*), 132.64 (*C$_d$*), 132.07 (Ar*C*H'), 130.60 (*C$_g$*), 129.78 (Ar*C*H'), 129.69 (Ar*C*H), 128.97 (Ar*C*H), 128.42 (*C$_{e'}$*), 126.74 (Ar*C*H), 125.39 (Ar*C*H'), 119.91 (q, $^1J_{CF}$ = 322.4, *C*F_3), 115.20 (Ar*C*H), 114.25(Ar*C*H'), 60.78 (*C$_a$*), 60.73 (*C$_{a'}$*), 55.62 (O*C*H_3'), 55.43 (O*C*H_3), 50.63 (*C$_{b'}$*), 50.39 (*C$_b$*); **^{19}F NMR** ($CDCl_3$, 377 MHz) δ –75.07. See spectra

1.7. Scope of the epoxide formation reaction

Benzyl 2-methyl-2-phenyl-1-oxa-5-azaspiro[2.3]hexane-5-carboxylate, 8a

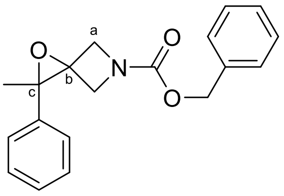

Prepared following general procedure 3a using the crude 1-(1-azabicyclo[1.1.0]butan-3-yl)-1-phenylethan-1-ol **5a** (0.25 mmol) and CbzCl (0.04 mL, 1.2 equiv). The crude reaction mixture was purified by flash column chromatography (98:2 → 85:15 pentane/acetone) to give **8a** (74 mg, 96%) as a colourless oil.

The same procedure was repeated on a 1.0 mmol scale (4 × scale) to give **8a** (289 mg, 93%) as a colourless oil.

R$_F$ 0.5 (80:20 pentane/acetone); **IR** (film) ν_{max}/cm^{-1}: 2959, 2871, 1711, 1445, 1405, 1351; **HRMS** (ESI): Calcd. for $C_{19}H_{19}NO_3Na^+$ ($[M+Na]^+$) *m/z* 332.1257, found *m/z* 332.1255;

^{1}H NMR ($CDCl_3$, 400 MHz) δ 7.35 – 7.25 (m, 8H, ArC*H*), 7.25 – 7.17 (m, 2H, ArC*H*), 5.08 (s, 2H, ROCH_2Ph), 4.35 (dd, $^2J_{HH}$ = 10.4, $^4J_{HH}$ = 1.5, 1H, C_aH), 4.26 (ddd, $^2J_{HH}$ = 10.5, $^4J_{HH}$ = 1.6, 0.8, 1H, C_aH), 4.12 (ddd, $^2J_{HH}$ = 10.6, $^4J_{HH}$ = 1.1, 0.8, 1H, C_aH), 3.76 (dd, $^2J_{HH}$ = 10.5, $^4J_{HH}$ = 1.6, 1H, C_aH), 1.63 (s, 3H, CH_3); **^{13}C NMR** ($CDCl_3$, 101 MHz) δ 156.31 (R_2N*C*(O)OR), 138.03 (Ar*C*), 136.46 (Ar*C*), 128.61 (Ar*C*H), 128.59 (Ar*C*H), 128.26 (Ar*C*H), 128.18 (Ar*C*H), 128.02 (Ar*C*H), 125.52 (Ar*C*H), 67.13 (RO*C*H_2Ph), 66.04 (C_b), 62.86 (C_c), 56.15 (br., 2 × C_a), 19.31 (*C*H_3). See spectra

tert-Butyl 3-(1-(1-((benzyloxy)carbonyl)-3-iodoazetidin-3-yl)-1-hydroxyethyl)-1H-indole-1-carboxylate, 8d' and benzyl 2-(1-(_tert_-butoxycarbonyl)-1H-indol-3-yl)-2-methyl-1-oxa-5-azaspiro[2.3]hexane-5-carboxylate, 8d

Prepared following general procedure 3b using the crude *tert*-Butyl 3-(1-(1-azabicyclo[1.1.0]butan-3-yl)-1-hydroxyethyl)-1H-indole-1-carboxylate **5d** (0.25 mmol). The crude reaction mixture was purified by flash column chromatography (98:2 → 85:15 pentane/acetone) and further purified by flash column chromatography (100:0 → 95:5 DCM/methanol) to give **8d'** (88 mg, 61%) as a colourless oil. After the addition of potassium carbonate and methanol, the crude mixture did not require purification and gave **8s** (69 mg, 62%) as a colourless oil.

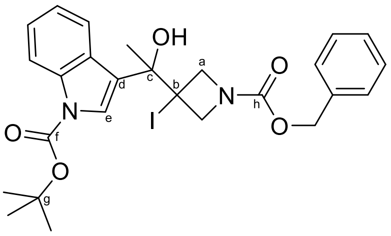

R$_F$ 0.32 (98:2 pentane/methanol); **IR** (film) ν_{max}/cm^{-1}: 3429, 2977, 1690, 1449, 1368, 1254, 1151; **HRMS** (ESI): Calcd. for $C_{26}H_{29}IN_2O_5Na^+$ ($[M+Na]^+$) *m/z* 599.1013, found *m/z* 599.0990;

^{1}H NMR ($CDCl_3$, 400 MHz) δ 8.06 (d, $^3J_{HH}$ = 8.3, 1H, ArC*H*), 7.85 (d, $^3J_{HH}$ = 7.9, 1H, ArC*H*), 7.59 (s, 1H, C_eH), 7.29 – 7.07 (m, 7H, ArC*H*), 4.93 (s, 2H, ROCH_2Ph), 4.83 (app. d, $^2J_{HH}$ = 10.0, 2H, 2 × C_aH), 4.45 (dd, $^2J_{HH}$ = 10.0, $^4J_{HH}$ = 1.2, 1H, C_aH), 4.33 (dd, $^2J_{HH}$ = 10.3, $^4J_{HH}$ = 1.2, 1H, C_aH), 2.64 (br. s, 1H, O*H*), 1.78 (s, 3H, CH_3), 1.60 (s, 9H, $C_g(CH_3)_3$); **^{13}C NMR** ($CDCl_3$, 101 MHz) δ 156.33 (C_h), 149.60 (C_f), 136.31 (Ar*C*), 135.55 (Ar*C*), 128.73 (Ar*C*), 128.55 (Ar*C*),

128.18 (Ar*C*H), 128.00 (Ar*C*H), 124.63 (Ar*C*H), 124.58 (C_e), 122.93 (Ar*C*H), 122.33 (Ar*C*H), 121.47 (C_d), 115.42 (Ar*C*H), 84.42 (C_g), 76.17 (C_c), 67.06 (RO*C*H_2Ph), 65.21 (br., 2 × C_a), 45.31 (C_b), 28.27 (C_g(*C*H_3)$_3$), 27.29 (*C*H_3). See spectra

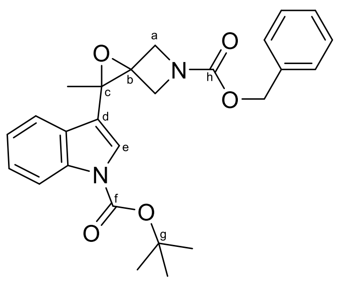

IR (film) ν_{max}/cm^{-1}: 2978, 1709, 1450, 1372, 1243, 1153; **HRMS** (ESI): Calcd. for $C_{26}H_{28}N_2O_5Na^+$ ($[M+Na]^+$) *m/z* 471.1890, found *m/z* 471.1907;

^{1}H NMR ($CDCl_3$, 400 MHz) δ 8.16 (d, $^3J_{HH}$ = 8.4, 1H, ArC*H*), 7.65 (dt, $^3J_{HH}$ = 7.9, $^4J_{HH}$ = 1.1, 1H, ArC*H*), 7.53 (s, 1H, C_e*H*), 7.33 (m, 6H, ArC*H*), 7.23 (ddd, $^3J_{HH}$ = 8.2, 7.2, $^4J_{HH}$ = 1.1, 1H, ArC*H*), 5.09 (s, 2H, ROC*H*$_2$Ph), 4.42 (dd, $^2J_{HH}$ = 10.4, $^4J_{HH}$ = 1.5, 1H, C_a*H*), 4.32 (ddd, $^2J_{HH}$ = 10.4, $^4J_{HH}$ = 1.5, 0.8, 1H, C_a*H*), 4.20 (ddd, $^2J_{HH}$ = 10.6, $^4J_{HH}$ = 1.5, 0.8, 1H, C_a*H*), 3.88 (dd, $^2J_{HH}$ = 10.6, $^4J_{HH}$ = 1.5, 1H, C_a*H*), 1.71 (s, 3H, C*H*$_3$), 1.68 (s, 9H, C_g(C*H*$_3$)$_3$); **^{13}C NMR** ($CDCl_3$, 101 MHz) δ 156.32 (C_h), 149.44 (C_f), 136.41 (Ar*C*), 135.66 (Ar*C*), 128.58 (Ar*C*H), 128.23 (Ar*C*), 128.15 (Ar*C*H), 128.07 (Ar*C*H), 124.85 (Ar*C*H), 123.97 (C_e), 122.97 (Ar*C*H), 120.22 (Ar*C*H), 118.53 (C_d), 115.50 (Ar*C*H), 84.25 (C_g), 67.12 (RO*C*H_2Ph), 65.55 (C_b), 60.36 (C_c), 56.33 (br., C_a), 56.04 (br., C_a), 28.26 (C_g(*C*H_3)$_3$), 20.27 (*C*H_3). See spectra

Benzyl 2-(6-bromopyridin-3-yl)-2-methyl-1-oxa-5-azaspiro[2.3]hexane-5-carboxylate, 8f

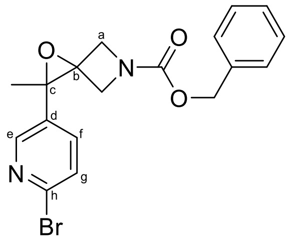

Prepared following general procedure 3a using the crude 1-(1-azabicyclo[1.1.0]butan-3-yl)-1-(6-bromopyridin-3-yl)ethan-1-ol **5f** (0.25 mmol) and CbzCl (0.04 mL, 1.2 equiv). The crude reaction mixture was purified by flash column chromatography (95:5 → 80:20 pentane/acetone) to give **8f** (64 mg, 66%) as a white solid.

R$_F$ 0.35 (80:20 pentane/acetone); **IR** (film) ν_{max}/cm^{-1}: 2967, 1737, 1715, 1454, 1408, 1353, 1216; **HRMS** (ESI): Calcd. for $C_{18}H_{17}BrN_2O_3Na^+$ ($[M+Na]^+$) *m/z* 411.0315, found *m/z* 411.0317

^{1}H NMR ($CDCl_3$, 400 MHz) δ 8.30 (dd, $^4J_{HH}$ = 2.5, $^5J_{HH}$ = 0.8, 1H, C_e*H*), 7.46 (dd, $^3J_{HH}$ = 8.3, $^5J_{HH}$ = 0.8, 1H, C_g*H*), 7.37 (dd, $^3J_{HH}$ = 8.3, $^4J_{HH}$ = 2.5, 1H, C_f*H*), 7.35 – 7.28 (m, 5H, ArC*H*), 5.09 (s, 2H, ROC*H*$_2$Ph), 4.36 (dd, $^2J_{HH}$ = 10.5, $^4J_{HH}$ = 1.6, 1H, C_a*H*), 4.27 (ddd, $^2J_{HH}$ = 10.5, $^4J_{HH}$ = 0.8, 0.8, 1H, C_a*H*), 4.13 (ddd, $^2J_{HH}$ = 10.5, $^4J_{HH}$ = 0.8, 0.8, 1H, C_a*H*), 3.77 (dd, $^2J_{HH}$ = 10.5, $^4J_{HH}$ = 1.6, 1H, C_a*H*), 1.64 (s, 3H, C*H*$_3$); **^{13}C NMR** ($CDCl_3$, 101 MHz) δ 156.17

(R_2N*C*(O)OR), 147.99 (*C_e*), 141.98 (*C_h*), 136.25 (Ar*C*), 135.78 (*C_e*), 133.37 (*C_d*), 128.60 (Ar*C*H), 128.31 (Ar*C*H), 128.19 (Ar*C*H), 127.91 (*C_g*), 67.26 (RO*C*H_2Ph), 66.26 (*C_c*), 60.87 (*C_d*), 55.94 (br., *C_a*), 55.76 (br., *C_a*), 18.92 (*C*H_3). See spectra

Benzyl 11-oxa-2-azadispiro[3.0.5.1]undecane-2-carboxylate, 8j

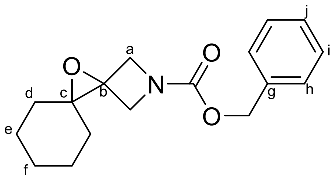

Prepared following general procedure 3a using the crude 1-(1-azabicyclo[1.1.0]butan-3-yl)cyclohexan-1-ol **5j** (0.25 mmol) and CbzCl (0.04 mL, 1.2 equiv). The crude reaction mixture was purified by flash column chromatography (95:5 → 80:20 pentane/acetone) to give **8j** (64 mg, 90%) as a colourless oil.

R$_F$ 0.35 (80:20 pentane/acetone); **IR** (film) ν_{max}/cm^{-1}: 2933, 2855, 1712, 1448, 1407, 1354, 1216; **HRMS** (ESI): Calcd. for $C_{17}H_{21}NO_3Na^+$ ([M+Na]$^+$) *m/z* 310.1414, found *m/z* 310.1412;

^{1}H NMR (CDCl$_3$, 400 MHz) δ 7.42 – 7.28 (m, 2H, ArC*H*), 5.12 (s, 2H, ROC*H_2*Ph), 4.22 (d, $^2J_{HH}$ = 11.0, 1H, 2 × C_a*H*), 4.17 (d, $^2J_{HH}$ = 11.0, 1H, 2 × C_a*H*), 1.79 – 1.67 (m, 2H, 2 × C_e*H*), 1.61 – 1.48 (m, 4H, 2 × C_e*H*, C_f*H_2*), 1.46 – 1.40 (m, 4H, 2 × C_d*H_2*); **^{13}C NMR** (CDCl$_3$, 101 MHz) δ 156.25 (R_2N*C*(O)OR), 136.48 (*C_g*), 128.53 (Ar*C*H), 128.15 (*C_j*), 128.07 (Ar*C*H), 67.01 (RO*C*H_2Ph), 64.10 (*C_b*), 63.96 (*C_c*), 55.88 (br., 2 × *C_a*), 30.93 (2 × *C_d*), 25.11 (*C_f*), 24.57 (2 × *C_d*). See spectra

2-Benzyl 7-(*tert*-butyl) 9-oxa-2,7-diazadispiro[3.0.3^5.1^4]nonane-2,7-dicarboxylate, 8l

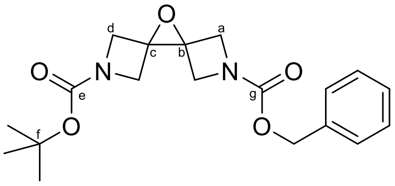

Prepared following general procedure 3a using the crude *tert*-butyl 3-(1-azabicyclo[1.1.0]butan-3-yl)-3-hydroxyazetidine-1-carboxylate **5l** (0.25 mmol) and CbzCl (0.04 mL, 1.2 equiv). The crude reaction mixture was purified by flash column chromatography (98:2 → 85:15 pentane/acetone) to give **8l** (82 mg, 91%) as a colourless oil.

R$_F$ 0.39 (80:20 pentane/acetone); **IR** (film) ν_{max}/cm^{-1}: 2969. 2942, 1697, 1454, 1390, 1352, 1143; **HRMS** (ESI): Calcd. for $C_{19}H_{24}N_2O_5Na^+$ ([M+Na]$^+$) *m/z* 383.1577, found *m/z* 383.1583;

^{1}H NMR (CDCl$_3$, 400 MHz) δ 7.39 – 7.28 (m, 5H, ArC*H*), 5.12 (s, 2H, ROC*H_2*Ph), 4.20 (s, 4H, 2 × C_a*H_2*), 4.11 (s, 2H, 2 × C_d*H_2*), 1.45 (s, 9H, C_f(C*H_3*)$_3$); **^{13}C NMR** (CDCl$_3$, 101 MHz) δ

156.26 (C_g), 155.95 (C_e), 136.27 (Ar*C*), 128.60 (Ar*C*H), 128.31 (Ar*C*H), 128.17 (Ar*C*H), 80.49 (C_f), 67.29 (RO*C*H$_2$Ph), 61.34 (C_c/C_b), 61.31 (C_c/C_b), 55.66 (br., 2 × C_a), 55.35 (br., 2 × C_a), 28.35 (C$_f$(*C*H$_3$)$_3$). See spectra

Benzyl 2-phenyl-1-oxa-5-azaspiro[2.3]hexane-5-carboxylate, 8n

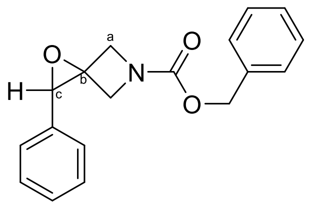

Prepared following general procedure 3a using the crude (1-Azabicyclo[1.1.0]butan-3-yl)(phenyl)methanol **5n** (0.25 mmol) and CbzCl (0.04 mL, 1.2 equiv). After the addition of potassium carbonate and methanol, the reaction was stirred for 42 hours at room temperature. The crude reaction mixture was purified by flash column chromatography (95:5 → 80:20 pentane/acetone) to give **8n** (59 mg, 80%) as a colourless oil.

R$_F$ 0.46 (80:20 pentane/acetone); **IR** (film) ν_{max}/cm^{-1}: 2945, 1735, 1712, 1446, 1409, 1354, 1217; **HRMS** (ESI): Calcd. for $C_{18}H_{17}NO_3Na^+$ ([M+H]$^+$) *m/z* 318.1101, found *m/z* 318.1103;

^{1}H NMR (CDCl$_3$, 400 MHz) δ 7.40 – 7.29 (m, 8H, ArC*H*), 7.17 – 7.10 (m, 2H, ArC*H*), 5.11 (s, 2H, ROC*H*$_2$Ph), 4.43 (dd, $^2J_{HH}$ = 10.4, $^4J_{HH}$ = 1.5, 1H, C$_a$*H*), 4.36 (d, $^2J_{HH}$ = 10.4, 1H, C$_a$*H*), 4.31 (dt, $^2J_{HH}$ = 10.5, $^4J_{HH}$ = 1.0, 1H, C$_a$*H*), 4.01 (s, 1H, C$_c$*H*), 3.97 (dd, $^2J_{HH}$ = 10.5, $^4J_{HH}$ = 1.6, 1H, C$_a$*H*); **^{13}C NMR** (CDCl$_3$, 101 MHz) δ 156.24 (R$_2$N*C*(O)OR), 136.41 (Ar*C*), 134.47 (Ar*C*), 128.71 (Ar*C*H), 128.69 (Ar*C*H), 128.58 (Ar*C*H), 128.24 (Ar*C*H), 128.15 (Ar*C*H), 125.78 (Ar*C*H), 67.14 (RO*C*H$_2$Ph), 62.55 (C_b), 60.06 (C_c), 57.22 (br., C_a), 55.90 (br., C_a). See spectra

Benzyl 3-(1-hydroxy-1,3-diphenylprop-2-yn-1-yl)-3-iodoazetidine-1-carboxylate, 8p' and benzyl 2-phenyl-2-(phenylethynyl)-1-oxa-5-azaspiro[2.3]hexane-5-carboxylate 8p

Prepared following general procedure 3b using the crude 1-(1-azabicyclo[1.1.0]butan-3-yl)-1,3-diphenylprop-2-yn-1-ol **5p** (0.25 mmol), a modification to the general procedure was that 5 mL of acetonitrile was required to dissolve **5p** rather than 2.5 mL. The crude reaction mixture was purified by flash column chromatography (100:0 → 95:5 DCM/ethyl acetate) to give **8p'** (121 mg, 98%) as a colourless oil. After the addition of potassium carbonate and methanol, the crude mixture did not require purification and gave **8p** (85 mg, 86%) as a colourless oil.

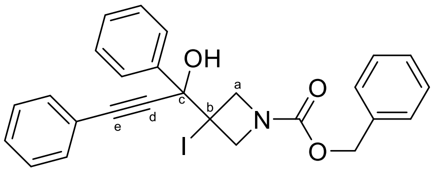

R_F 0.46 (95:5 DCM/ethyl acetate); **IR** (film) ν_{max}/cm^{-1}: 3339, 2969, 1737, 1715, 1685, 1420, 1354, 1216; **HRMS** (ESI): Calcd. for $C_{26}H_{22}INO_3Na^+$ ($[M+Na]^+$) *m/z* 546.0537, found *m/z* 546.0510;

^{1}H NMR ($CDCl_3$, 400 MHz) δ 7.70 (dd, $^3J_{HH}$ = 7.4, $^4J_{HH}$ = 2.3, 2H, ArC*H*), 7.51 – 7.41 (m, 2H, ArC*H*), 7.40 – 7.21 (m, 11H, ArC*H*), 5.16 (d, $^2J_{HH}$ = 10.2, 1H, C_aH), 5.09 (d, $^2J_{HH}$ = 10.2, 1H, C_aH), 5.00 (s, 2H, ROCH_2Ph), 4.47 (d, $^2J_{HH}$ = 10.0, 1H, C_aH), 4.45 (d, $^2J_{HH}$ = 10.0, 1H, C_aH), 3.38 (s, 1H, O*H*); **^{13}C NMR** ($CDCl_3$, 101 MHz) δ 156.42 (R_2NC(O)OR), 140.51 (Ar*C*), 136.30 (Ar*C*), 131.98 (Ar*C*H), 129.26 (Ar*C*H), 128.93 (Ar*C*H), 128.54 (Ar*C*H), 128.52 (Ar*C*H), 128.17 (Ar*C*H), 128.09 (Ar*C*H), 128.01 (Ar*C*H), 127.25 (Ar*C*H), 121.55 (Ar*C*), 88.16 (C_e), 86.81 (C_d), 76.69 (C_c), 67.12 (RO*C*H_2Ph), 65.06 (br., 2 × C_a), 41.44 (C_b). See spectra

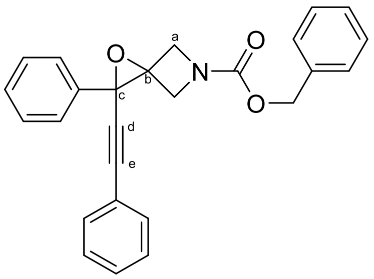

IR (film) ν_{max}/cm^{-1}: 2968, 2943, 1712, 1443, 1403, 1352, 1216; **HRMS** (ESI): Calcd. for $C_{26}H_{21}NO_3Na^+$ ($[M+Na]^+$) *m/z* 418.1414, found *m/z* 418.1428;

^{1}H NMR ($CDCl_3$, 400 MHz) δ 7.53 (dd, $^3J_{HH}$ = 7.9, $^4J_{HH}$ = 1.7, 2H, ArC*H*), 7.46 – 7.29 (m, 13H ArC*H*), 5.12 (s, 2H, ROCH_2Ph), 4.59 (dd, $^2J_{HH}$ = 10.7, $^4J_{HH}$ = 1.6, 1H, C_aH), 4.43 (ddd, $^2J_{HH}$ = 10.7, $^4J_{HH}$ = 0.7, 1H, C_aH), 4.29 (ddd, $^2J_{HH}$ = 10.7, $^4J_{HH}$ = 0.7, 1H, C_aH), 3.91 (dd, $^2J_{HH}$ = 10.7, $^4J_{HH}$ = 1.6 , 1H, C_aH); **^{13}C NMR** ($CDCl_3$, 101 MHz) δ 156.30 (R_2NC(O)OR), 136.35 (Ar*C*), 134.14 (Ar*C*), 132.25 (Ar*C*H), 129.40 (Ar*C*H), 128.92 (Ar*C*H), 128.67 (Ar*C*H), 128.62 (Ar*C*H), 128.54 (Ar*C*H), 128.30 (Ar*C*H), 128.23 (Ar*C*H), 126.04 (Ar*C*H), 121.45 (Ar*C*), 87.26 (C_e), 83.84 (C_d), 68.64 (C_c), 67.27 (RO*C*H_2Ph), 58.34 (C_b), 56.84 (C_a), 55.63 (C_a). See spectra

2,2,2-Trifluoro-1-(2-phenyl-2-(trifluoromethyl)-1-oxa-5-azaspiro[2.3]hexan-5-yl)ethan-1-one, 8s

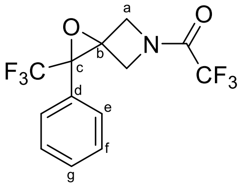

Prepared following general procedure 3a using 1-(1-azabicyclo[1.1.0]butan-3-yl)-2,2,2-trifluoro-1-phenylethan-1-ol **5s** (0.25 mmol) and TFAA (0.04 mL, 1.2 equiv) in place of CbzCl. The crude reaction mixture was purified by flash column chromatography (99:1 → 85:15 pentane/acetone) to give **8s** (40 mg, 49%) as a colourless oil.

R_F 0.34 (90:10 pentane/methanol); **IR** (film) ν_{max}/cm^{-1}: 2950, 1704, 1514, 1494, 1451, 1333, 1291, 1251, 1141; **HRMS** (APCI): Calcd. for $C_{13}H_9NO_2F_6^+$ ([M+H]$^+$) *m/z* 326.0610, found *m/z* 326.0619;

Rotamers observed.

^{1}H NMR ($CDCl_3$, 400 MHz) δ 7.51 – 7.38 (m, 5H, ArC*H*), 4.88 (d, $^2J_{HH}$ = 12.4, 1H), 4.83 (d, $^2J_{HH}$ = 11.8, 1H), 4.69 (d, $^2J_{HH}$ = 13.0, 1H), 4.61 (dd, $^2J_{HH}$ = 13.1, $^4J_{HH}$ = 2.1, 1H), 4.38 (dt, $^2J_{HH}$ = 11.8, $^4J_{HH}$ = 1.1, 1H), 4.27 (ddd, $^2J_{HH}$ = 11.8, $^4J_{HH}$ = 2.2, 1.2, 1H), 4.14 (d, $^2J_{HH}$ = 12.7, 0H), 4.09 (dd, $^2J_{HH}$ = 12.8, $^4J_{HH}$ = 1.9, 1H); **^{13}C NMR** ($CDCl_3$, 101 MHz) δ 156.09 (q, $^2J_{CF}$ = 38.3, R_2NC(O)CF_3), 156.05 (q, $^2J_{CF}$ = 38.3, R_2NC(O)CF_3), 130.42 (C_g), 129.24 (ArCH), 129.18 (ArCH), 127.85 (C_d), 127.69 (C_d), 127.04 (ArCH), 126.97 (ArCH), 122.79 (q, $^1J_{CF}$ = 278.5, C_cCF_3), 122.73 (q, $^1J_{CF}$ = 279.1, C_cCF_3), 115.94 (q, $^1J_{CF}$ = 287.6, $R_2NC(O)CF_3$), 115.87 (q, $^1J_{CF}$ = 287.6, $R_2NC(O)CF_3$), 64.36 (C_b), 64.25 (C_b), 62.52 (q, $^2J_{CF}$ = 37.3, C_c), 62.47 (q, $^2J_{CF}$ = 37.3, C_c), 58.49 (m, C_a), 57.73 (m, C_a), 56.22, (m, C_a), 55.38 (C_a); **^{19}F NMR** ($CDCl_3$, 377 MHz) δ -71.60 (C_cCF_3), -71.63 (C_cCF_3), –72.78 ($R_2NC(O)CF_3$), –72.81 ($R_2NC(O)CF_3$).
See spectra

2,2,2-Trifluoro-1-(2-methyl-2-phenyl-1-oxa-5-azaspiro[2.3]hexan-5-yl)ethan-1-one, 14

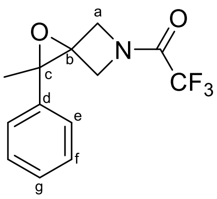

Prepared following general procedure 3a using the crude 1-(1-azabicyclo[1.1.0]butan-3-yl)-1-phenylethan-1-ol **5a** (0.25 mmol) and TFAA (0.04 mL, 1.2 equiv) in place of CbzCl. The crude reaction mixture was purified by flash column chromatography (98:2 → 85:15 pentane/acetone) to give **14** (26 mg, 38%) as a colourless oil. Ketone **6a** was also isolated (24 mg, 36%).

R_F 0.49 (80:20 pentane/acetone); **IR** (film) ν_{max}/cm^{-1}: 2969, 1738, 1446, 1365, 1229, 1216; **HRMS** (ESI): Calcd. for $C_{13}H_{12}F3NO_3Na^+$ ([M+Na]$^+$) *m/z* 294.0712, found *m/z* 294.0723;

Rotamers observed.

^{1}H NMR ($CDCl_3$, 400 MHz) δ 7.52 – 7.15 (m, 5H, ArC*H*), 4.71 (d, $^2J_{HH}$ = 11.1, 1H, C_aH), 4.63 (d, $^2J_{HH}$ = 11.2, 1H, C_aH), 4.51 (d, $^2J_{HH}$ = 12.7, 1H, C_aH), 4.47 (d, $^2J_{HH}$ = 12.1, 1H, C_aH), 4.39 (d, $^2J_{HH}$ = 12.0, 1H, C_aH), 4.24 (d, $^2J_{HH}$ = 12.3, 1H, C_aH), 4.10 (d, $^2J_{HH}$ = 11.1, 1H, C_aH), 3.93 (d, $^2J_{HH}$ = 12.2, 1H, C_aH), 1.69 (s, 6H, CH_3); **^{13}C NMR** ($CDCl_3$, 101 MHz) δ 156.19 (q,

$^{2}J_{CF}$ = 37.8, $CF_3\mathit{C}(O)NR_2$), 156.10 (q, $^{2}J_{CF}$ = 37.8, $CF_3\mathit{C}(O)NR_2$), 137.33 (C_d), 137.16 (C_d), 128.87 (Ar*C*H), 128.81 (Ar*C*H), 128.45 (Ar*C*H), 125.49 (Ar*C*H), 125.43 (Ar*C*H), 116.12 (q, $^{1}J_{CF}$ = 287.8, $\mathit{C}F_3$), 116.03 (q, $^{1}J_{CF}$ = 287.8, $\mathit{C}F_3$), 65.58 (q, $^{4}J_{CF}$ = 1.8, C_b), 63.23 (C_c), 63.18 (C_c), 58.33 (q, $^{3}J_{CF}$ = 2.0, C_a), 55.99 (C_a), 55.95 (C_a), 19.34 ($\mathit{C}H_3$), 19.28 ($\mathit{C}H_3$); **^{19}F NMR** ($CDCl_3$, 377 MHz) δ –72.72, –72.78. See spectra

2-Methyl-2-phenyl-5-tosyl-1-oxa-5-azaspiro[2.3]hexane, 15

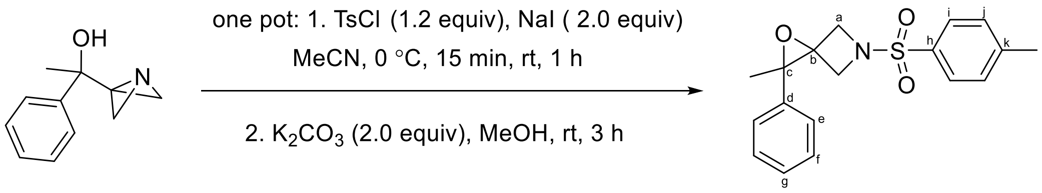

To a round bottom flask containing a mixture of crude 1-(1-azabicyclo[1.1.0]butan-3-yl)-1-phenylethan-1-ol (**5a**, 0.25 mmol, 1.0 equiv) and sodium iodide (75 mg, 0.5 mmol, 2.0 equiv) under an inert atmosphere was added anhydrous acetonitrile (2.5 mL). The flask was cooled to 0 °C and tosyl chloide (57 mg, 0.3 mmol, 1.2 equiv) was added with stirring. After 15 minutes at 0 °C, the reaction was warmed to room temperature and stirred for a further 1 hour. After this time, potassium carbonate (69 mg, 0.5 mmol, 2.0 equiv) was added followed by anhydrous methanol (2.5 mL) and the reaction stirred for 3 hours. Diethyl ether (10 mL) and saturated aqueous sodium bicarbonate solution (10 mL) were added, and the reaction mixture transferred to a separating funnel. The phases were separated, and the aqueous phase was extracted with diethyl ether (3 × 20 mL) and the combined organic phases were washed with brine, dried ($MgSO_4$), filtered and concentrated under reduced pressure. The crude reaction mixture was purified by flash column chromatography (99:1 → 80:20 pentane/acetone) to give **15** (72 mg, 87%) as a colourless oil that crystallised upon freezing.

R_F 0.56 (80:20 pentane/acetone); **IR** (film) ν_{max}/cm^{-1}: 2976, 2937, 1597, 1445, 1346, 1160; **HRMS** (APCI): Calcd. for $C_{18}H_{19}NO_3S^+$ ($[M+H]^+$) *m/z* 330.1158, found *m/z* 330.1160

^{1}H NMR ($CDCl_3$, 400 MHz) δ 7.72 (d, $^{3}J_{HH}$ = 8.2, 2H, 2 × $C_i\mathit{H}$), 7.37 (d, *J* = 8.2, 2H, 2 × $C_j\mathit{H}$), 7.34 – 7.24 (m, 3H, 2 × $C_f\mathit{H}$, $C_g\mathit{H}$), 7.12 – 7.03 (m, 2H, 2 × $C_e\mathit{H}$), 4.17 (d, $^{2}J_{HH}$ = 10.2, 1H, $C_a\mathit{H}$), 4.08 (d, $^{2}J_{HH}$ = 10.2, 1H, $C_a\mathit{H}$), 3.90 (d, $^{2}J_{HH}$ = 10.3, 1H, $C_a\mathit{H}$), 3.55 (d, $^{2}J_{HH}$ = 10.3, 1H, $C_a\mathit{H}$), 2.48 (s, 3H, Ar$C\mathit{H}_3$), 1.53 (s, 3H, $C\mathit{H}_3$); **^{13}C NMR** ($CDCl_3$, 101 MHz) δ 144.46 (C_h), 137.39 (C_d), 131.56 (C_k), 129.94 (2 × C_j), 128.45 (2 × C_i), 128.41 (2 × C_f), 128.05 (C_g), 125.37

(2 × C_e), 64.11 (C_b), 63.10 (C_c), 57.07 (C_a), 56.98 (C_a), 21.64 (ArCH$_3$), 19.13 (CH$_3$). See spectra

tert-Butyl 2-methyl-2-phenyl-1-oxa-5-azaspiro[2.3]hexane-5-carboxylate, 16

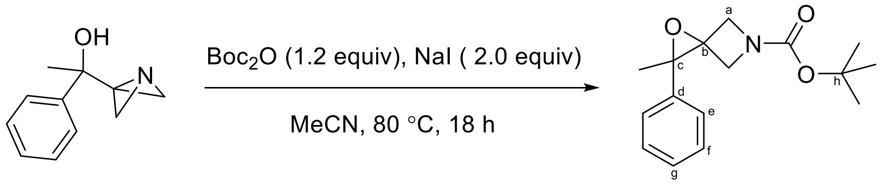

To a round bottom flask containing a mixture of crude 1-(1-azabicyclo[1.1.0]butan-3-yl)-1-phenylethan-1-ol (**5a**, 0.25 mmol, 1.0 equiv) and sodium iodide (75 mg, 0.5 mmol, 2.0 equiv) under an inert atmosphere was added anhydrous acetonitrile (2.5 mL). The flask was cooled to 0 °C and di-*tert*-butyl decarbonate (0.07 mL, 0.3 mmol, 1.2 equiv) was added with stirring and the reaction was then heated to 80 C. After 18 hours, the reaction was cooled to room temperature before the addition of saturated aqueous sodium bicarbonate solution (10 mL) and diethyl ether (10 mL) were added, and the reaction mixture transferred to a separating funnel. The phases were separated, and the aqueous phase was extracted with diethyl ether (3 × 20 mL) and the combined organic phases were washed with brine, dried ($MgSO_4$), filtered and concentrated under reduced pressure. The crude reaction mixture was purified by flash column chromatography (99:1 → 90:10 pentane/acetone) to give **16** (39 mg, 57%) as a colourless oil.

R_F 0.56 (80:20 pentane/acetone); **IR** (film) ν_{max}/cm^{-1}: 2974, 2874, 1702, 1446, 1388, 1366, 1141; **HRMS** (ESI): Calcd. for $C_{16}H_{21}NO_3Na^+$ ([M+Na]$^+$) *m/z* 298.1414, found *m/z* 298.1411;

^{1}H NMR ($CDCl_3$, 400 MHz) δ 7.35 (tt, $^3J_{HH}$ = 6.8, $^4J_{HH}$ = 1.6, 2H, 2 × C$_f$*H*), 7.34 – 7.26 (tt, $^3J_{HH}$ = 7.29, $^4J_{HH}$ = 1.5 1H, C$_g$*H*), 7.25 (m, 2H, 2 × C$_e$*H*), 4.27 (dd, $^2J_{HH}$ = 10.4, $^4J_{HH}$ = 1.6, 1H, C$_a$*H*), 4.18 (ddd, $^2J_{HH}$ = 10.4, $^4J_{HH}$ = 1.6, 0.8, 1H, C$_a$*H*), 4.04 (ddd, $^2J_{HH}$ = 10.5, $^4J_{HH}$ = 1.6, 0.8, 1H, C$_a$*H*), 3.68 (dd, $^2J_{HH}$ = 10.5, $^4J_{HH}$ = 1.6, 1H, C$_a$*H*), 1.65 (s, 3H, C*H*$_3$), 1.42 (s, 9H, C$_h$(C*H*$_3$)$_3$); **^{13}C NMR** ($CDCl_3$, 101 MHz) δ 156.11 (R$_2$N*C*(O)OR), 138.27 (C_d), 128.56 (2 × C_f), 127.93 (C_g), 125.57 (2 × C_e), 80.05 (C_h), 65.96 (C_b), 62.82 (C_c), 55.89 (br., 2 × C_a), 28.42 (C$_h$(CH$_3$)$_3$), 19.39 (CH$_3$). See spectra

2. NMR Spectra

Crude ^{1}H NMR ($CDCl_3$, 400 MHz) of **5a**, See procedure

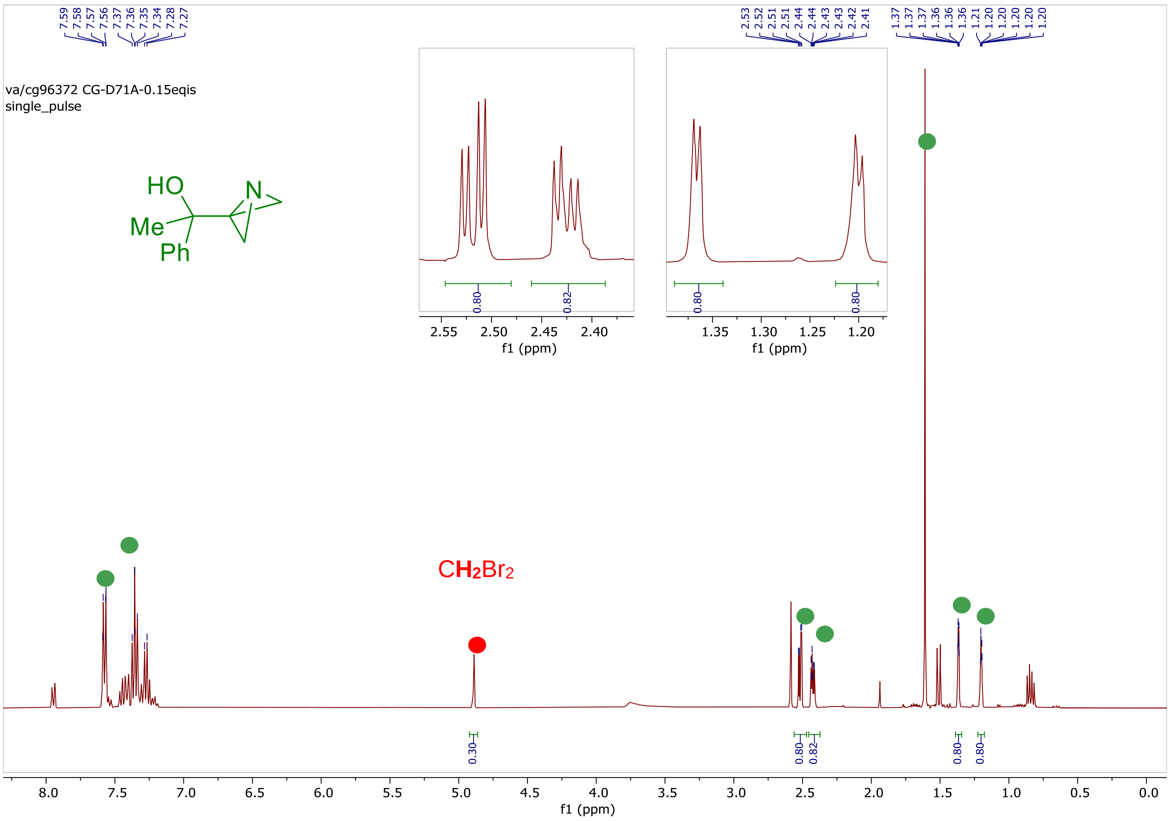

Crude ^{1}H NMR ($CDCl_3$, 400 MHz) of **5b**, See procedure

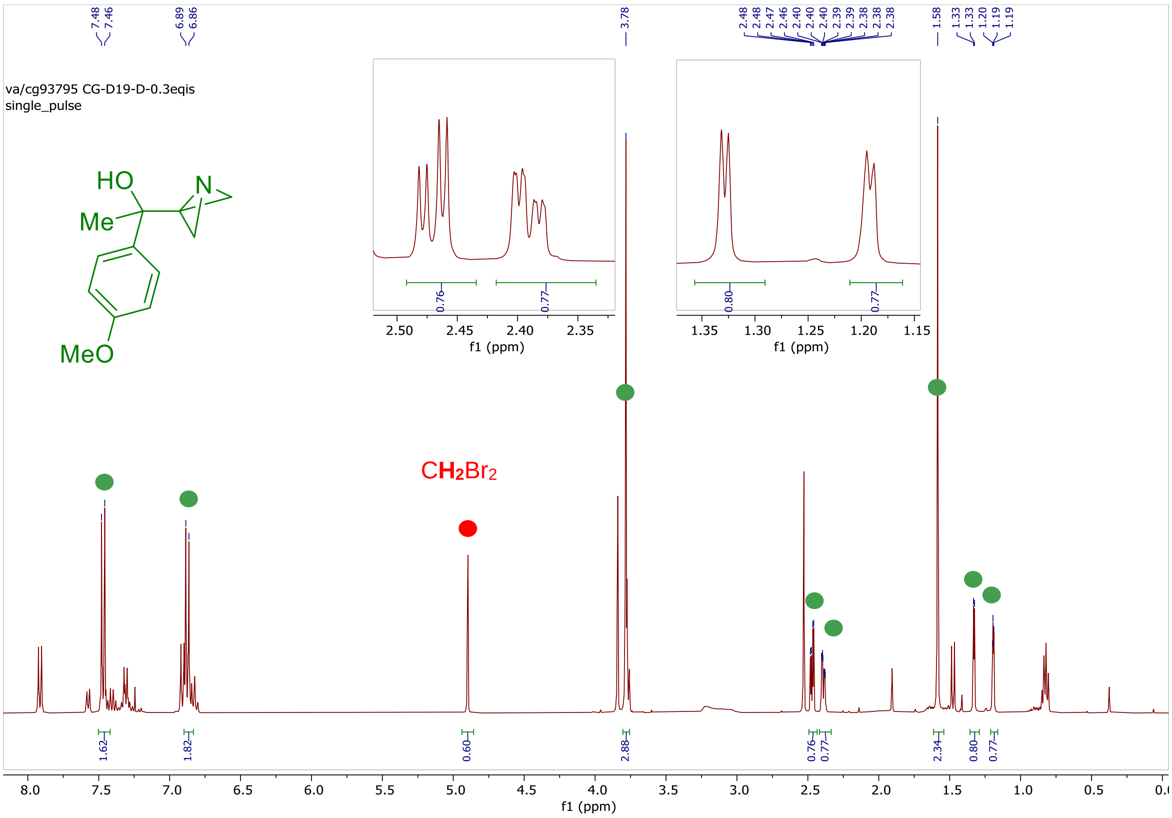

Crude ¹H NMR (CDCl$_3$, 400 MHz) of **5c**, See procedure

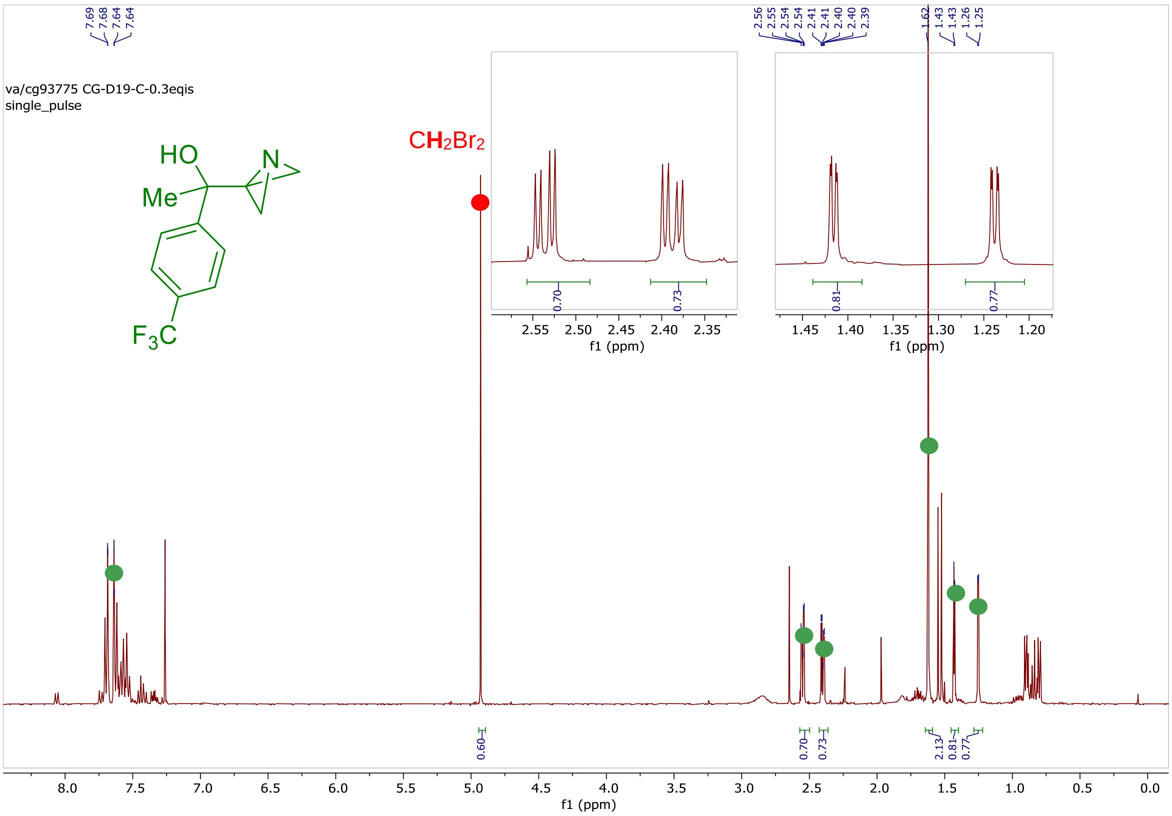

Crude ¹H NMR (CDCl$_3$, 400 MHz) of **5d**, See procedure

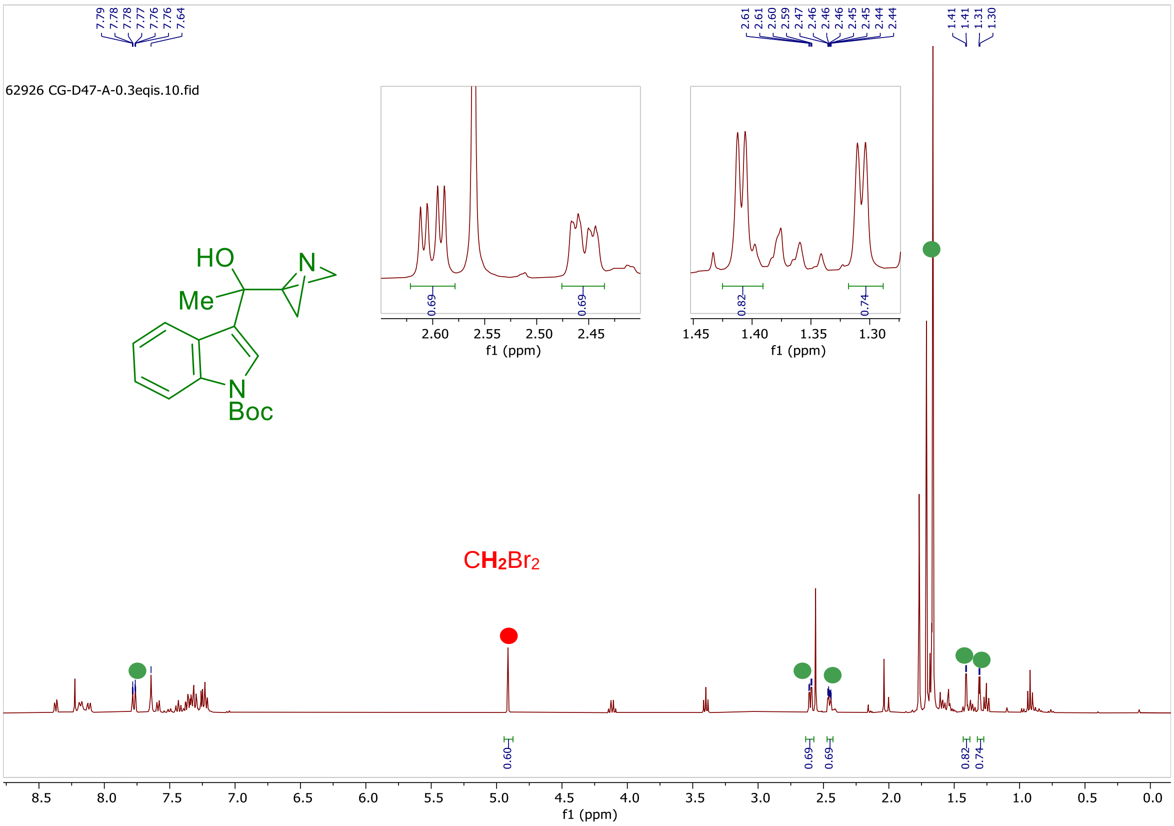

Crude ^{1}H NMR (CDCl$_3$, 400 MHz) of **5e**, See procedure

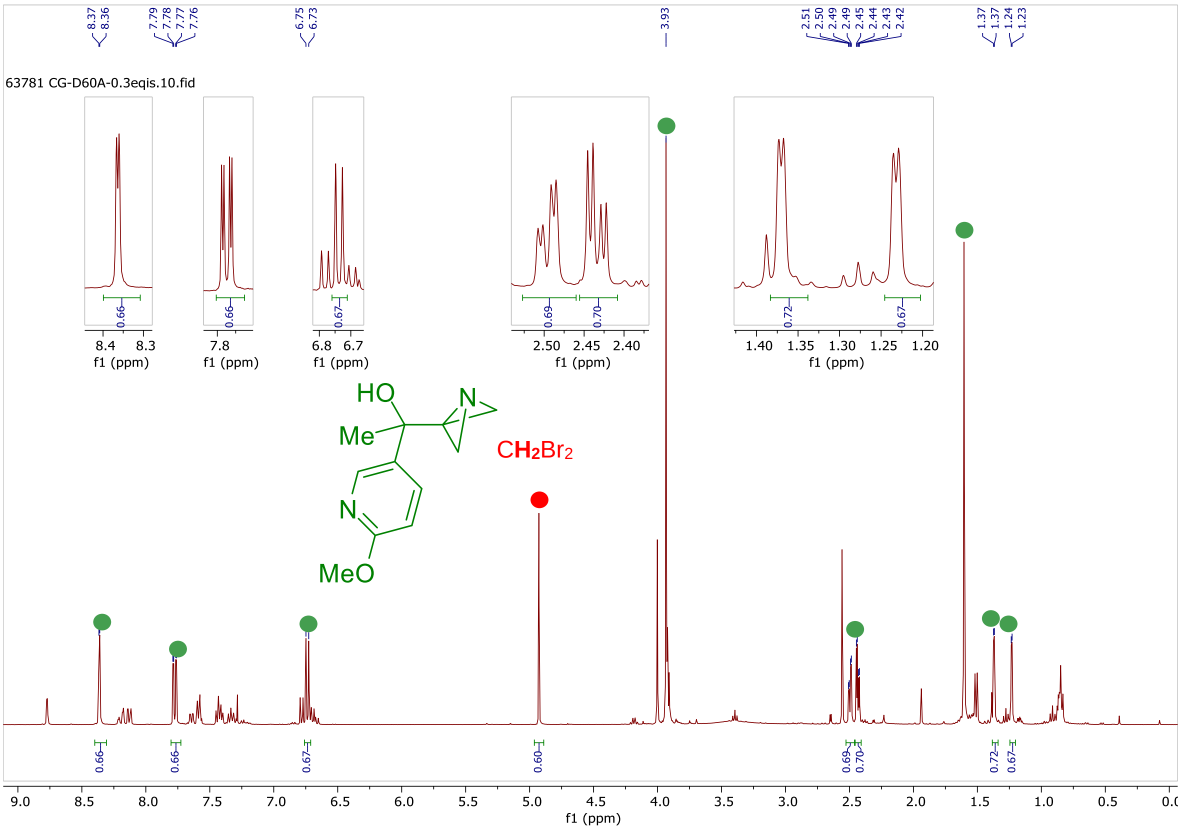

Crude ^{1}H NMR (CDCl$_3$, 400 MHz) of **5f**, See procedure

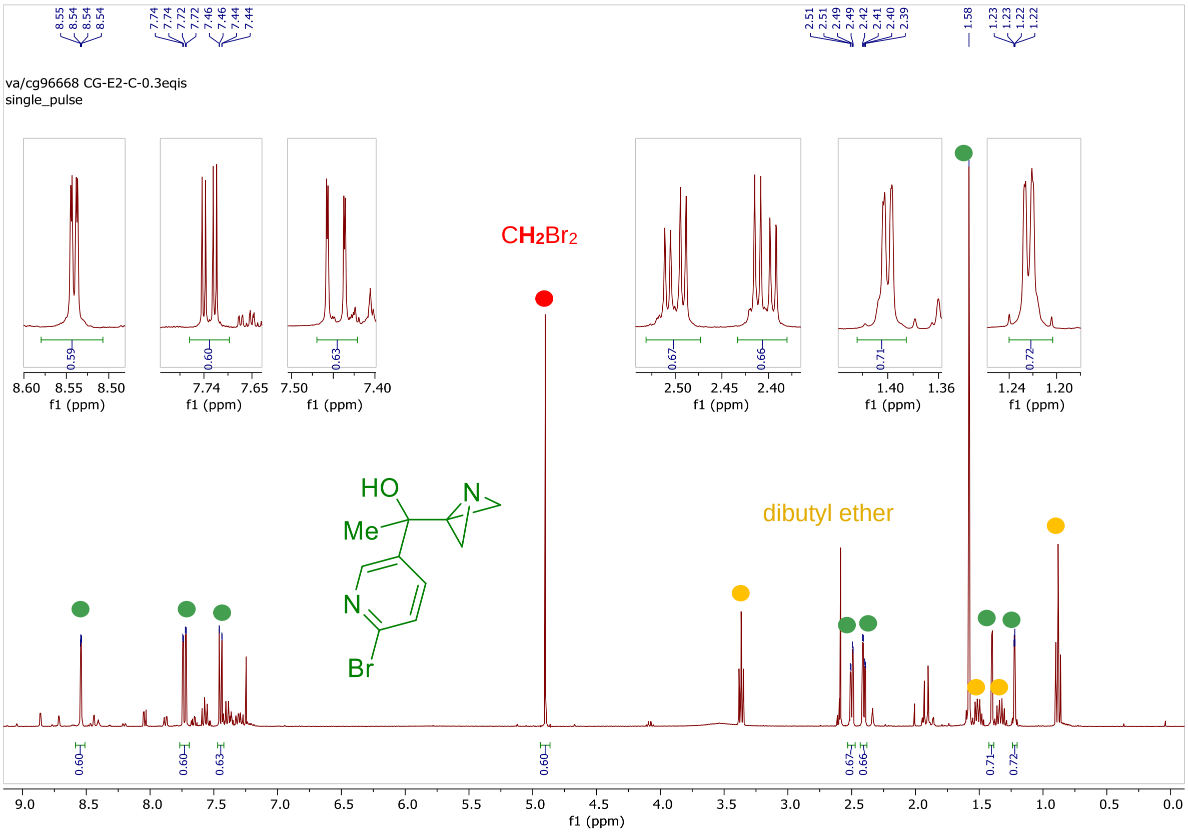

Crude ^{1}H NMR ($CDCl_3$, 400 MHz) of **5g**, See procedure

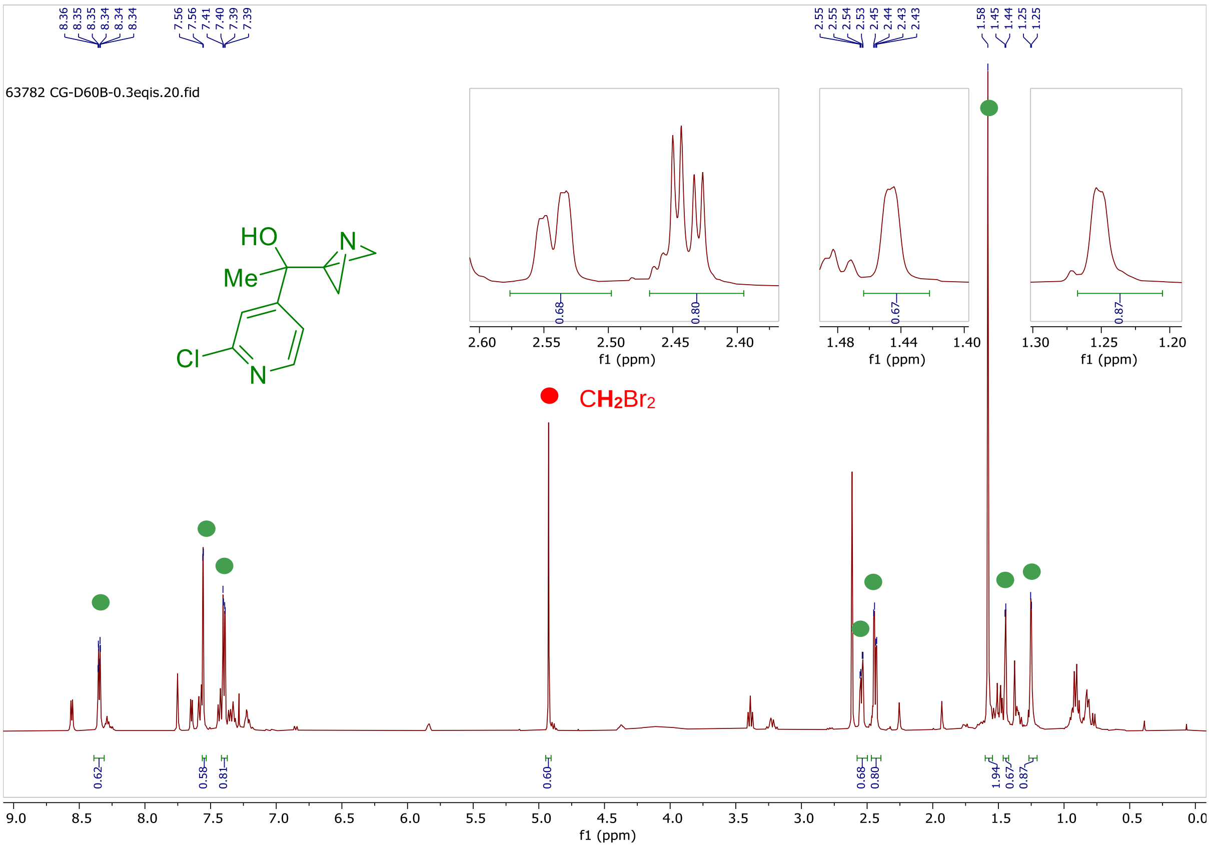

Crude ^{1}H NMR ($CDCl_3$, 400 MHz) of **5h**, See procedure

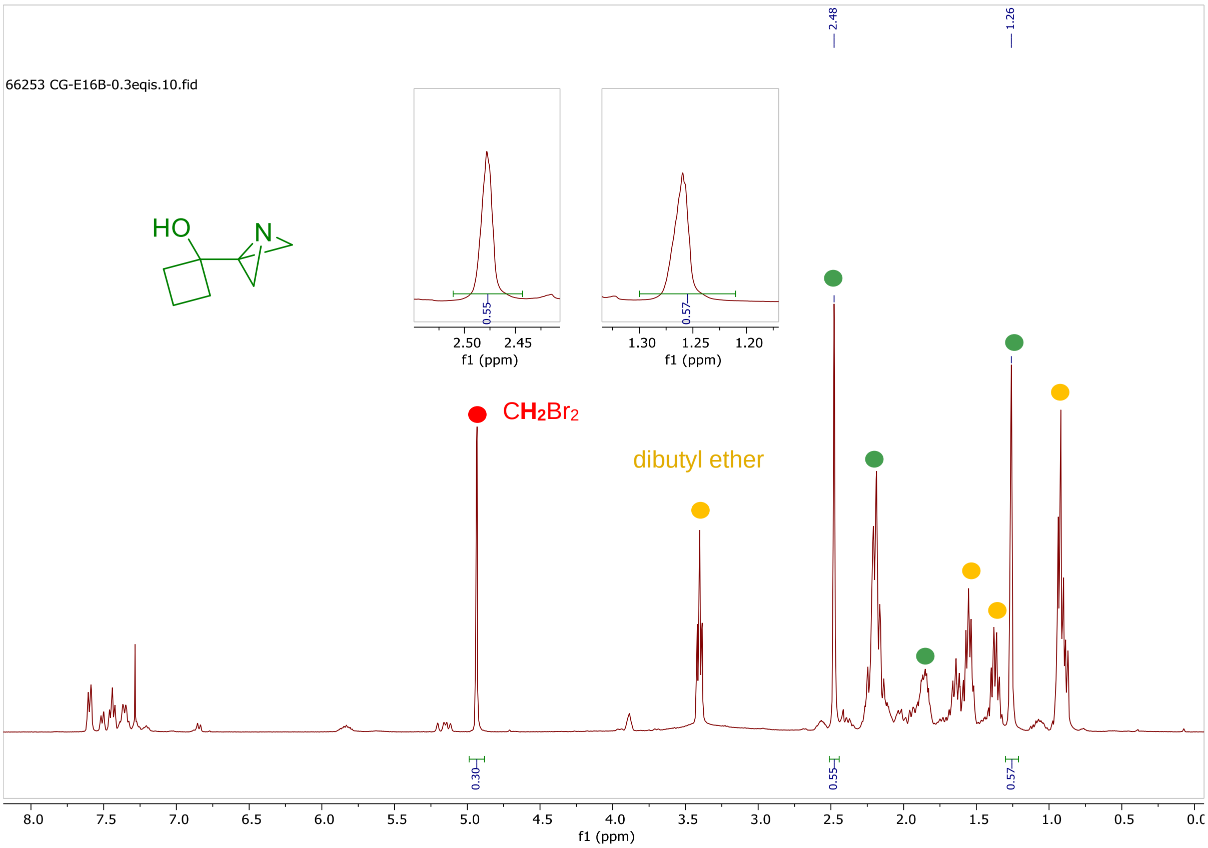

Crude ^{1}H NMR (CDCl$_3$, 400 MHz) of **5i**, See procedure

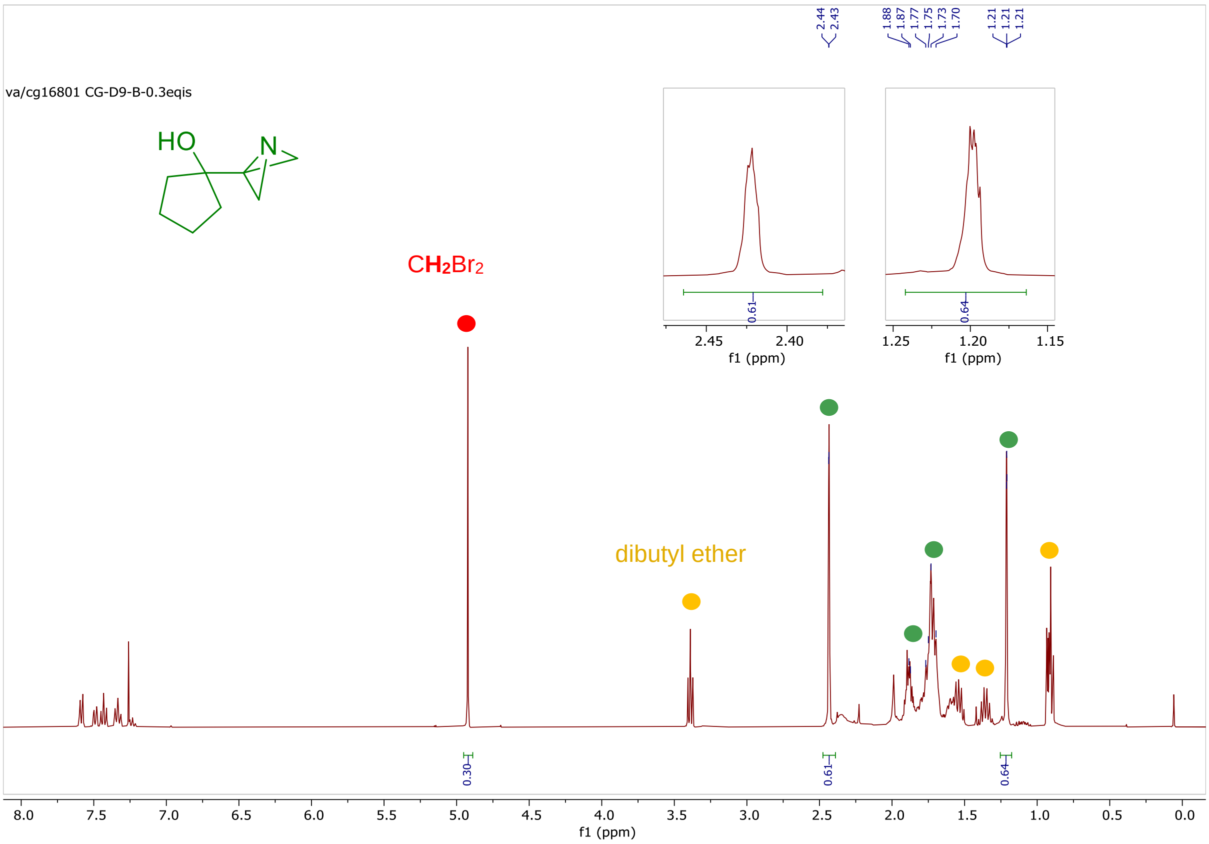

Crude ^{1}H NMR (CDCl$_3$, 400 MHz) of **5j**, See procedure

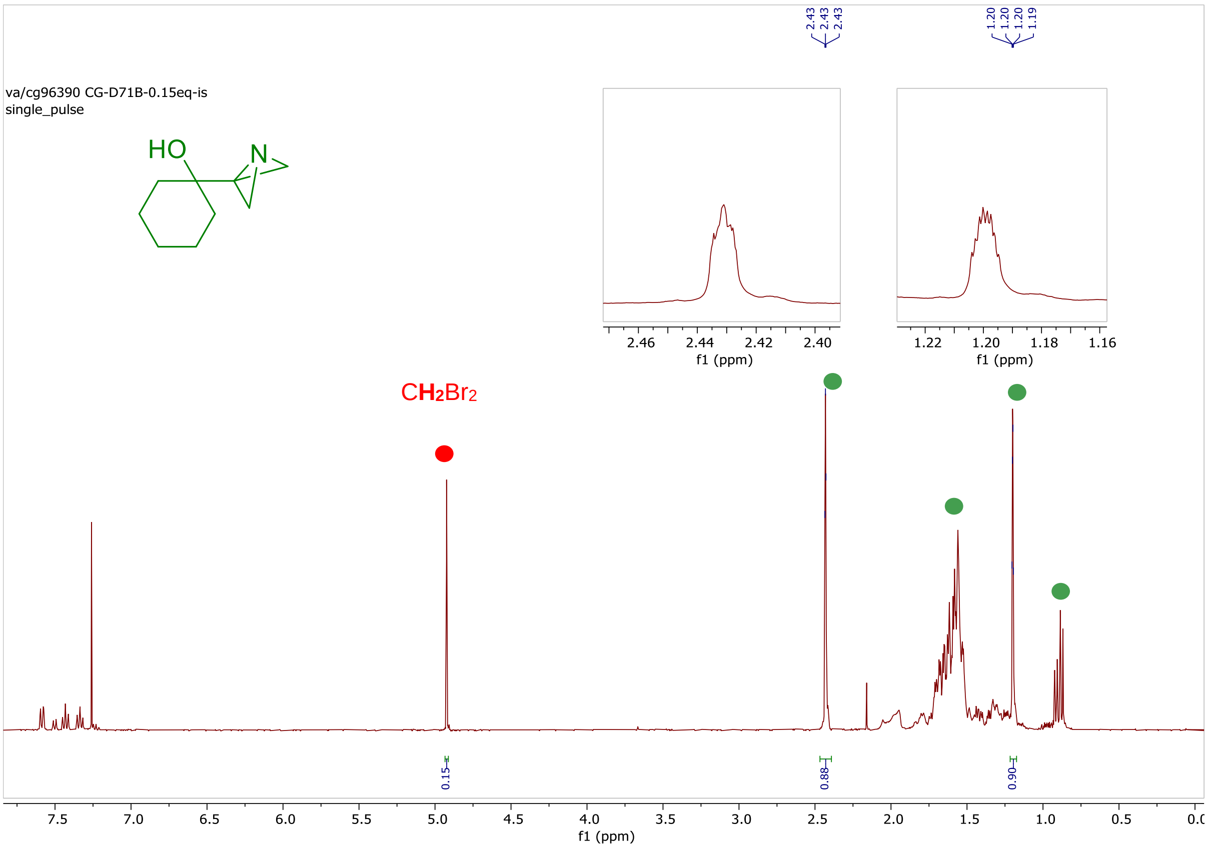

Crude ¹H NMR (CDCl$_3$, 400 MHz) of **5k**, See procedure

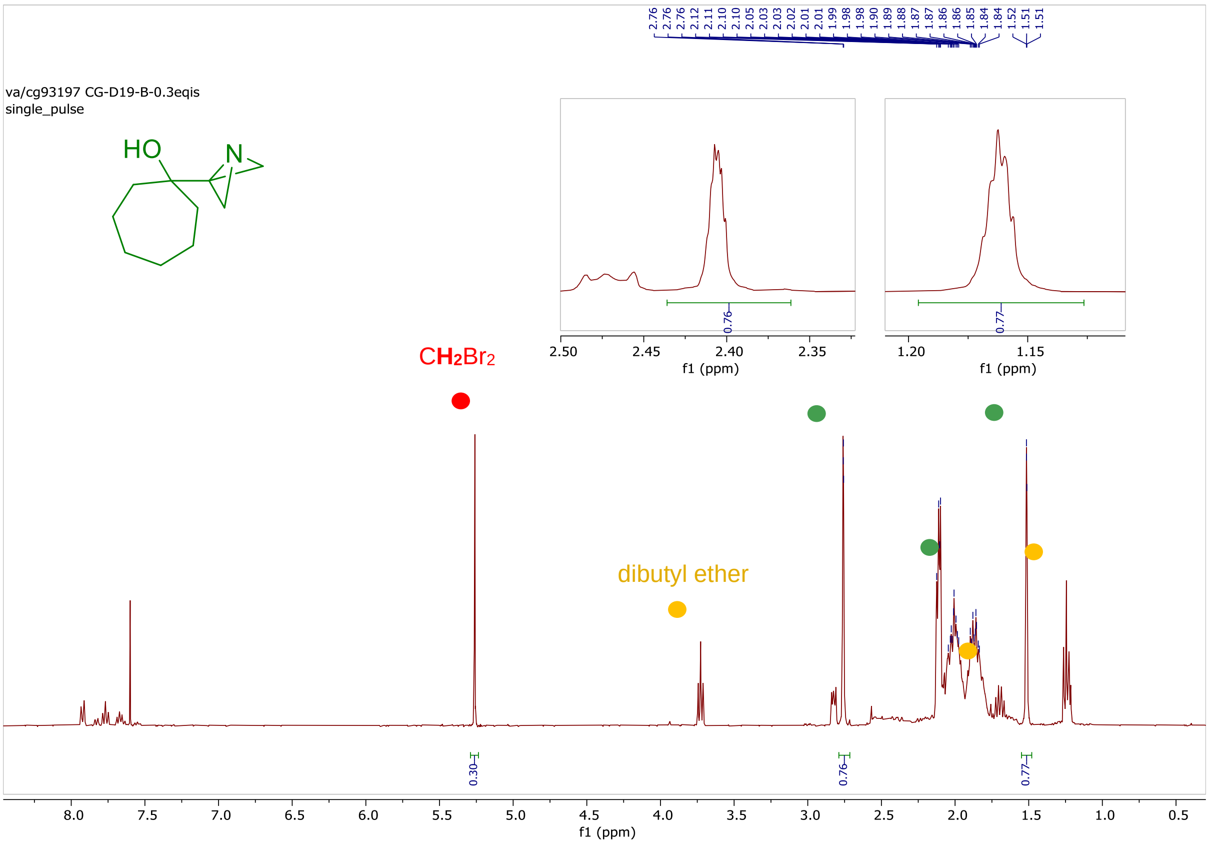

Crude ¹H NMR (CDCl$_3$, 400 MHz) of **5l**, See procedure

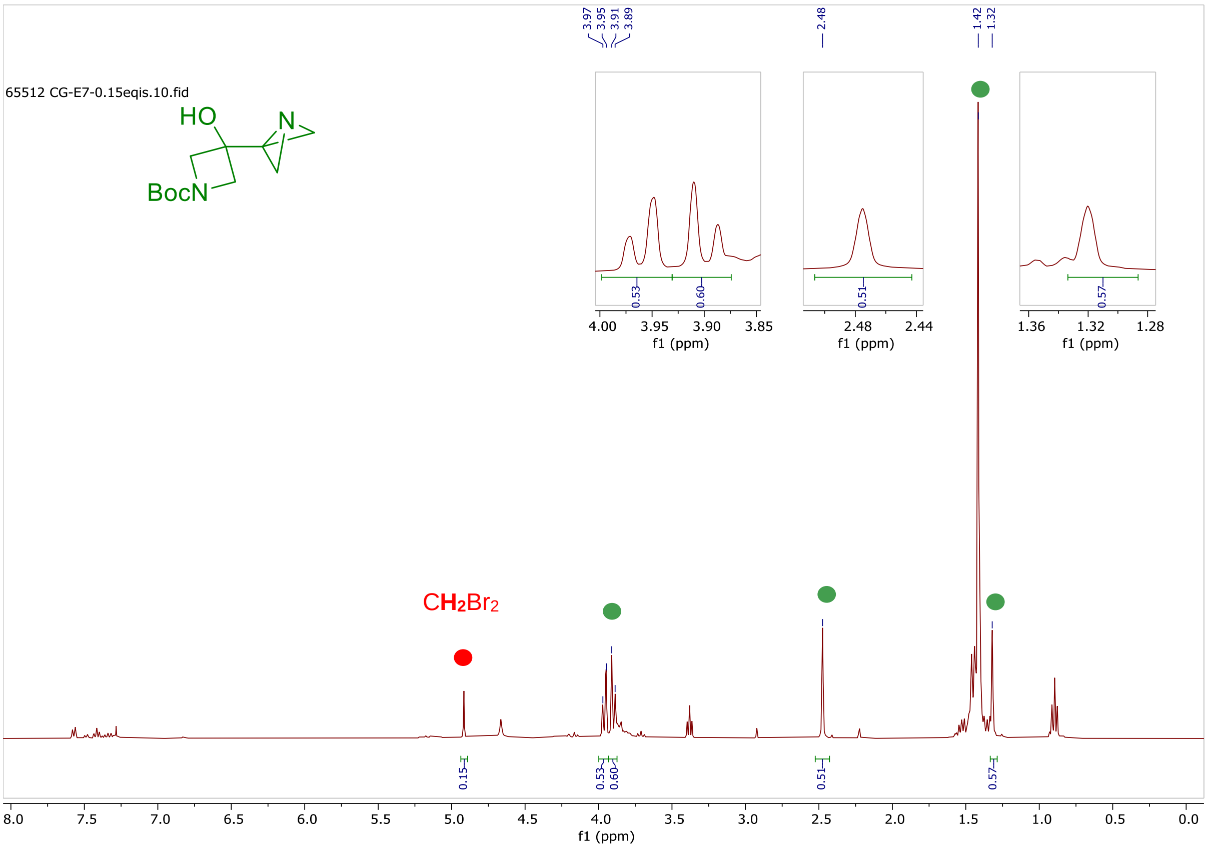

Crude ^{1}H NMR ($CDCl_3$, 400 MHz) of **5m**, See procedure

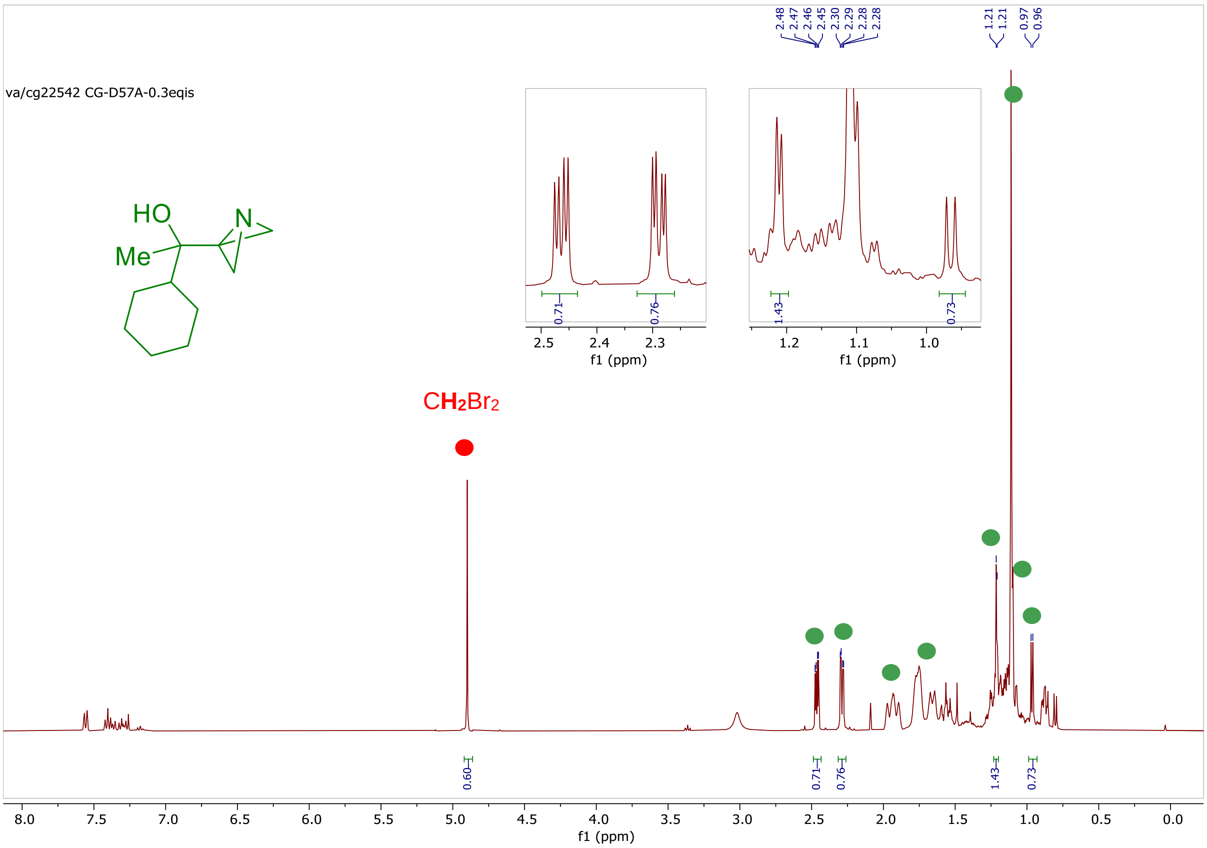

Crude ^{1}H NMR ($CDCl_3$, 400 MHz) of **5n**, See procedure

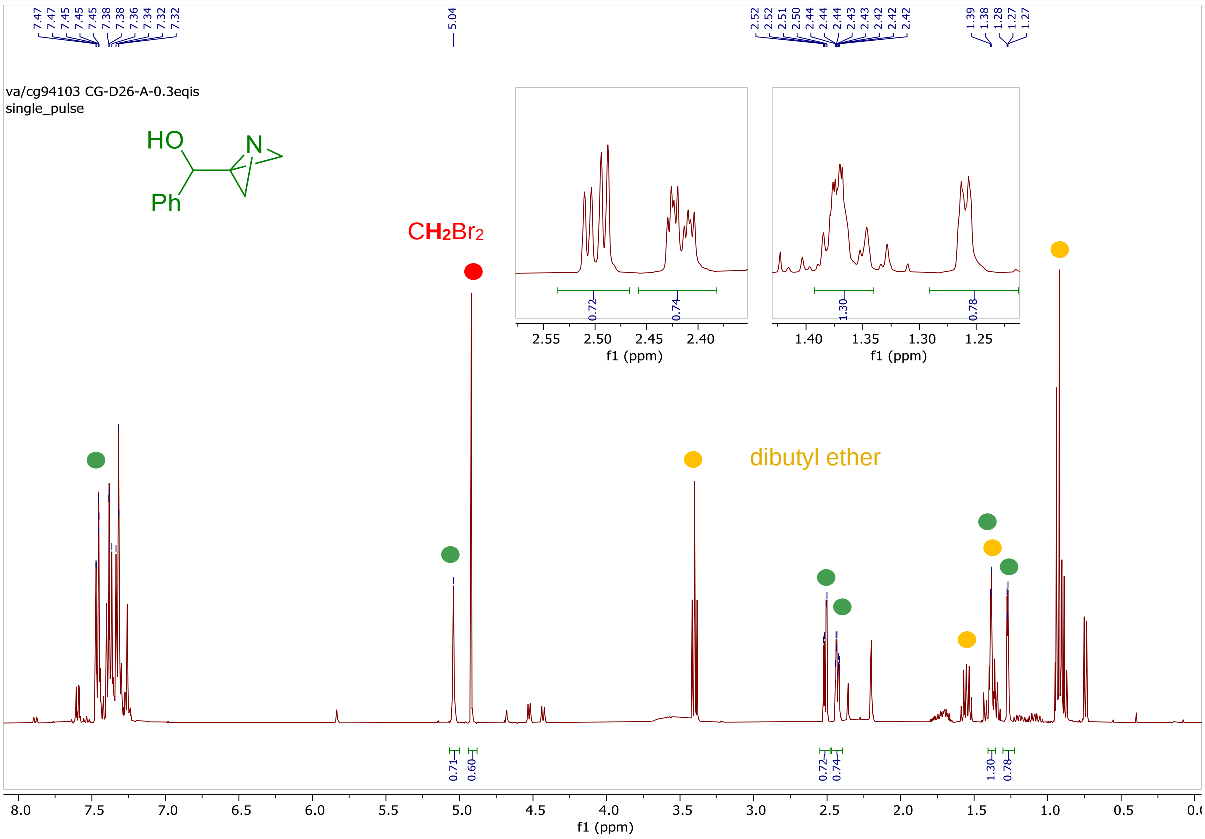

Crude ^{1}H NMR (CDCl$_3$, 400 MHz) of **5o**, See procedure

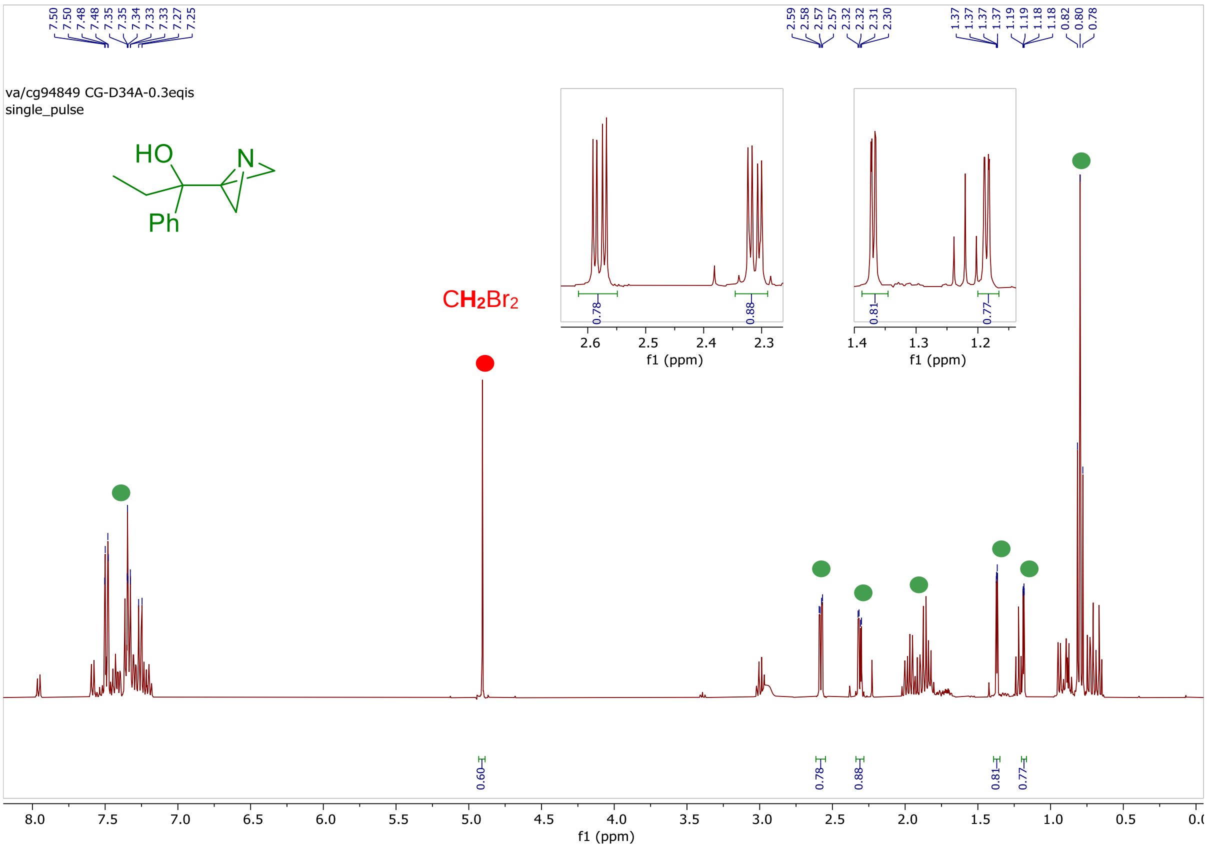

Crude ^{1}H NMR (CDCl$_3$, 400 MHz) of **5p**, See procedure

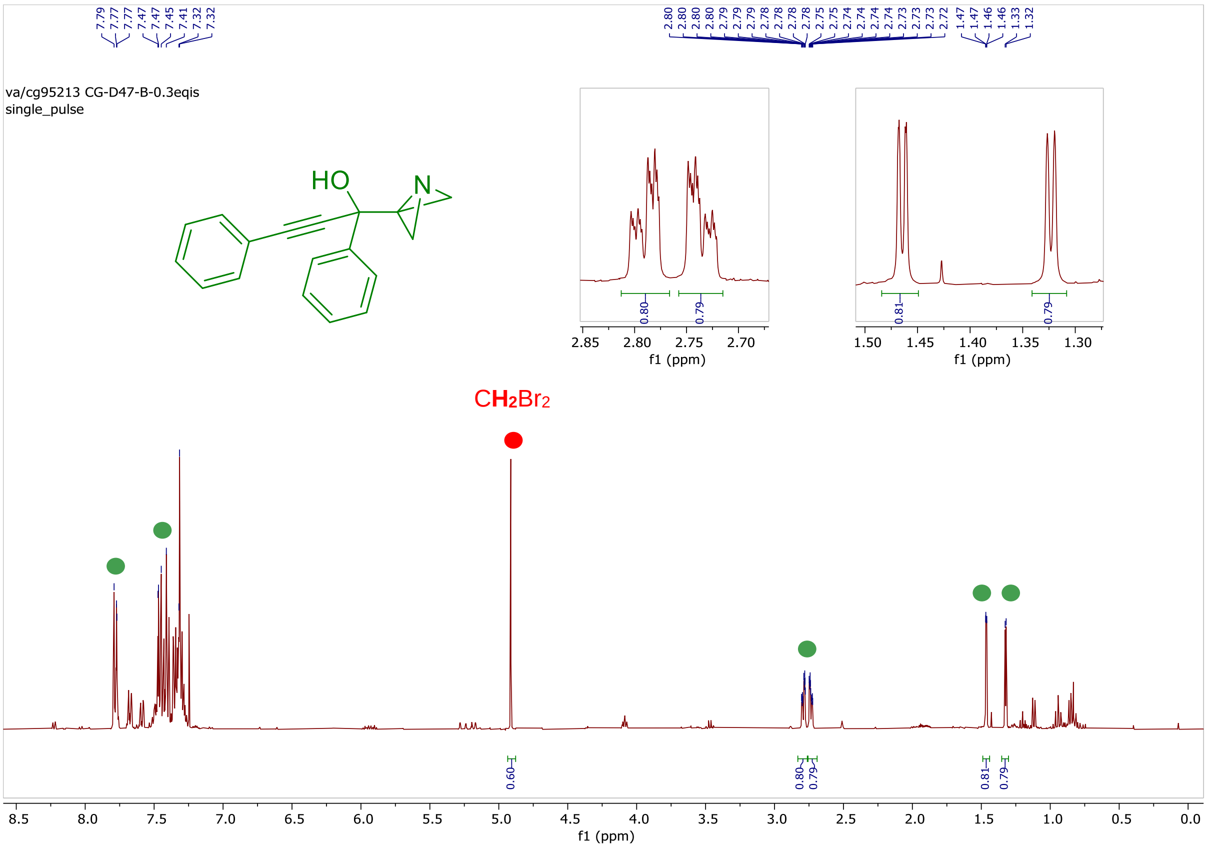

Crude ^{1}H NMR ($CDCl_3$, 400 MHz) of **5q**, See procedure

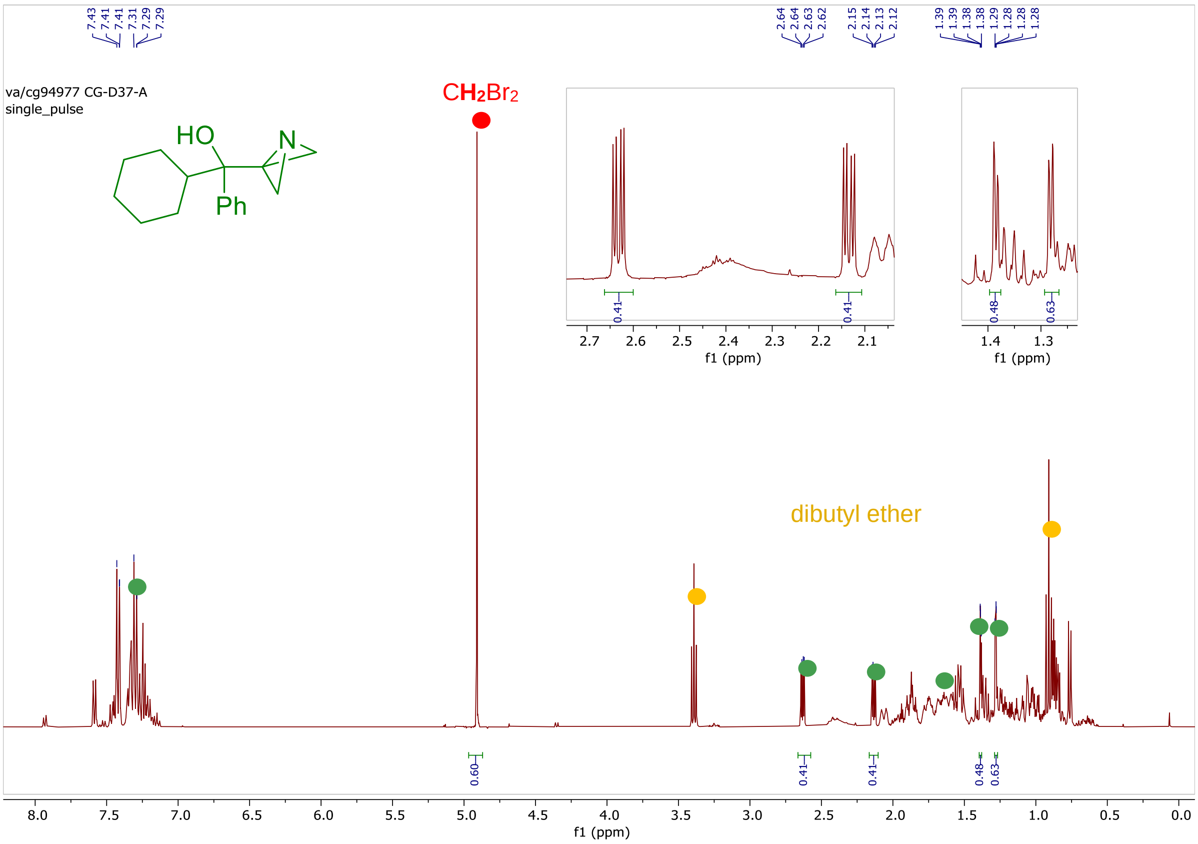

^{1}H NMR ($CDCl_3$, 400 MHz) of **5r**, See procedure

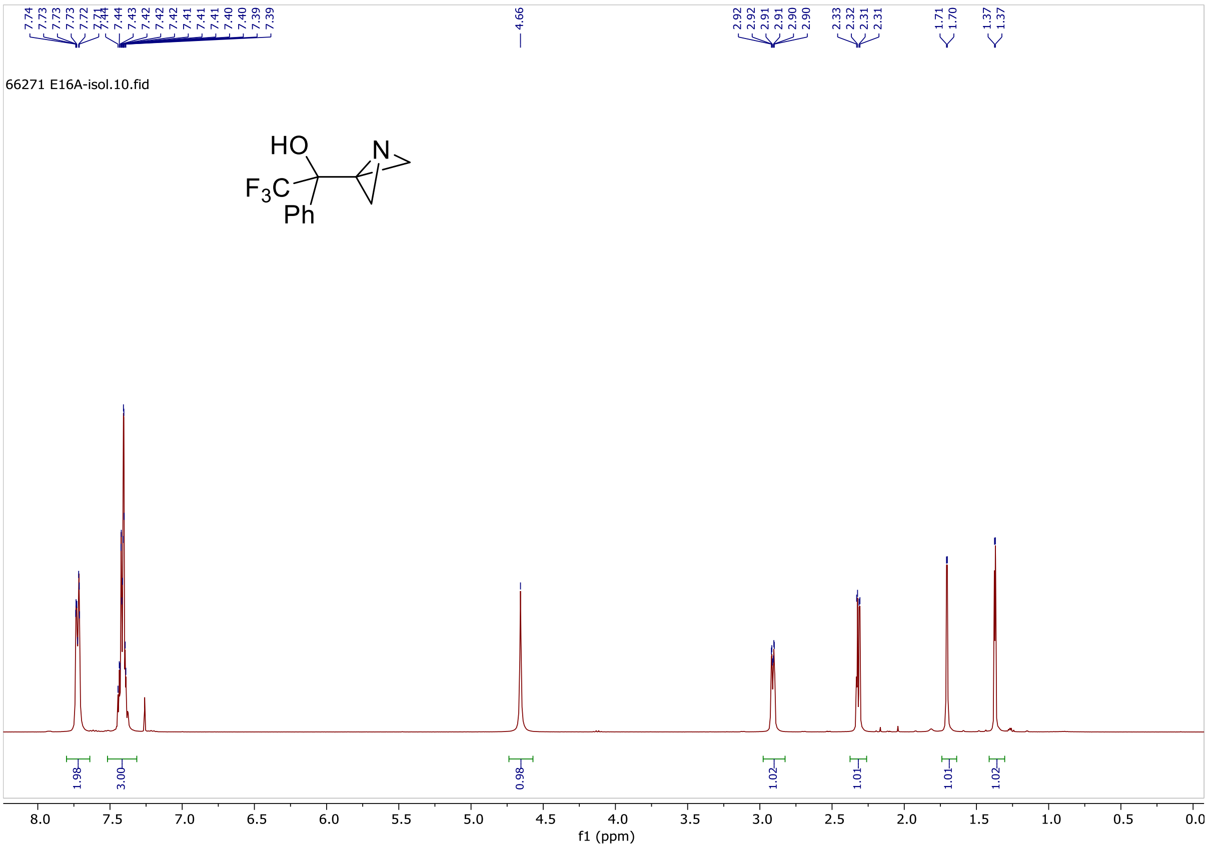

^{13}**C NMR** (CDCl$_3$, 101 MHz) of **5r**

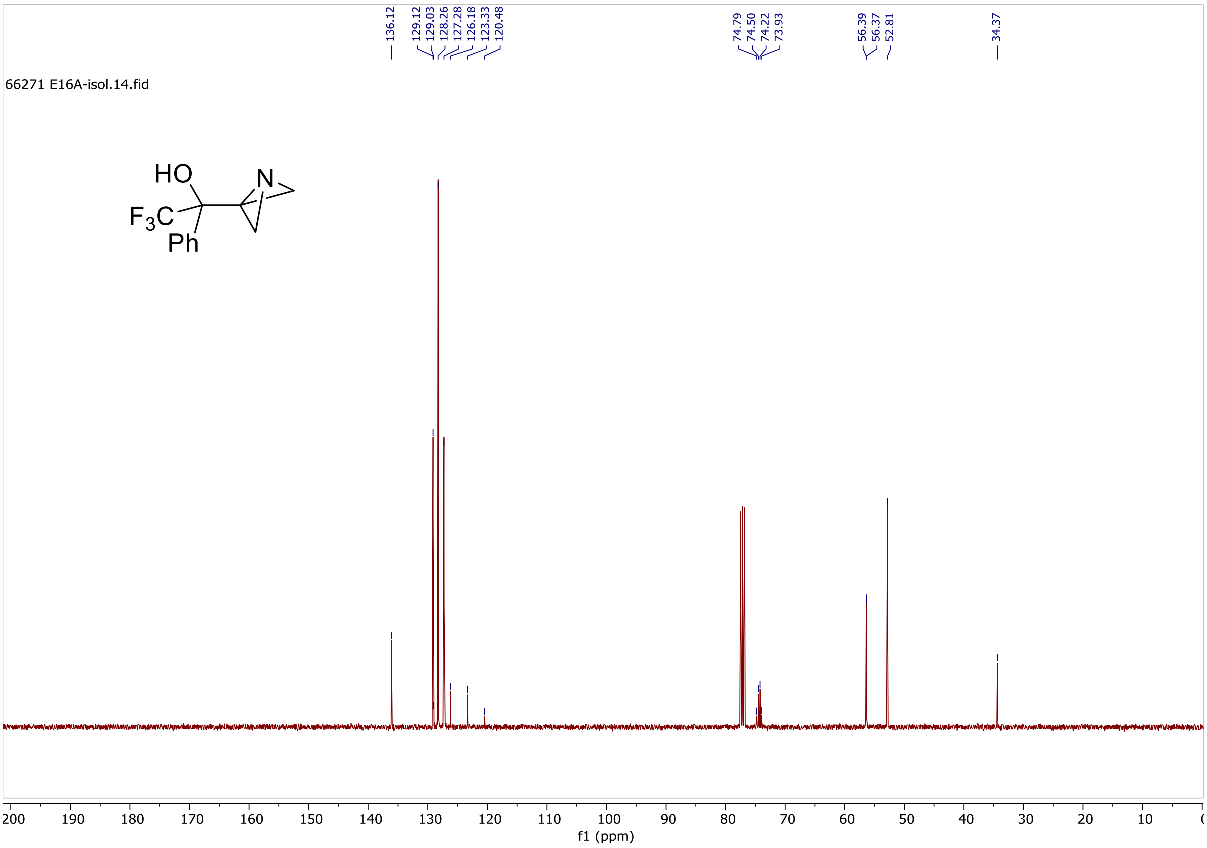

Crude ^{1}**H NMR** (CDCl$_3$, 400 MHz) of **5s**, See procedure

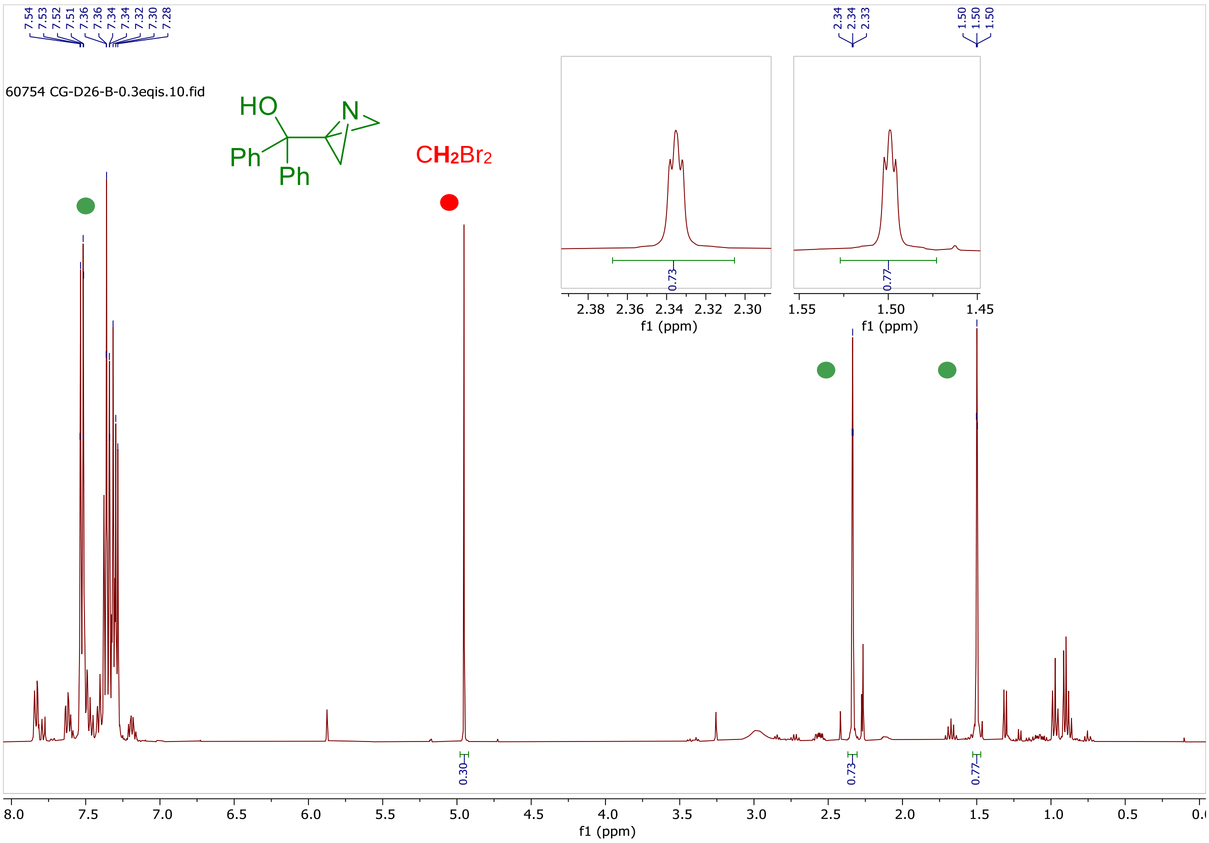

Crude ^{1}H NMR ($CDCl_3$, 400 MHz) of **5t**, See procedure

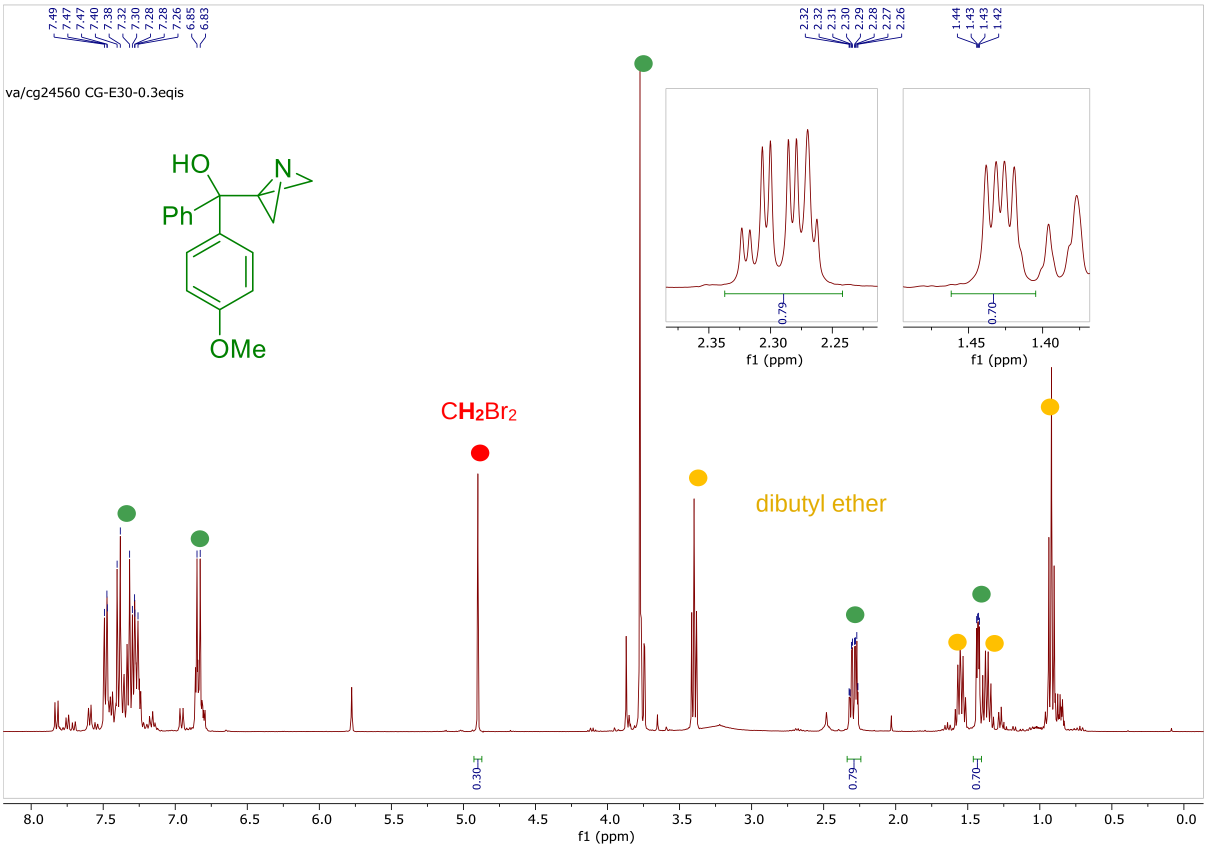

^{1}H NMR ($CDCl_3$, 400 MHz) of **10**, See procedure

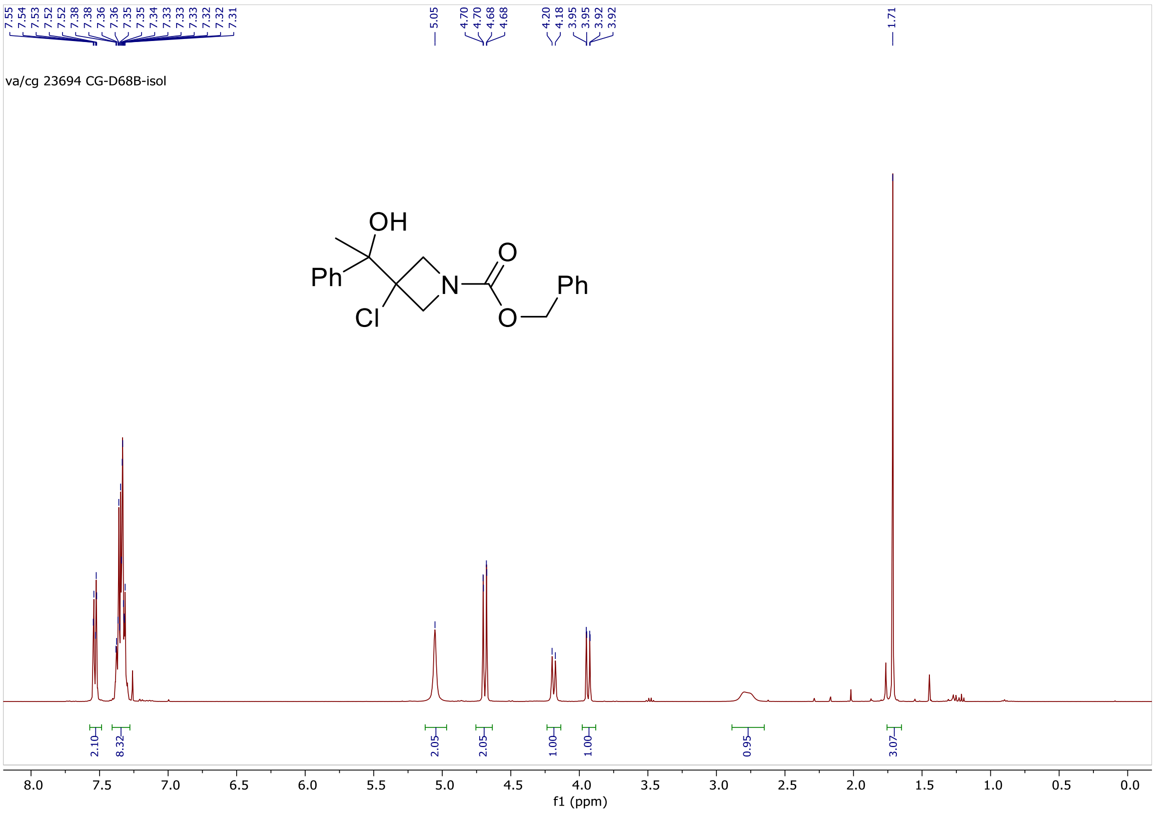

^{13}C NMR (CDCl$_3$, 101 MHz) of **10**

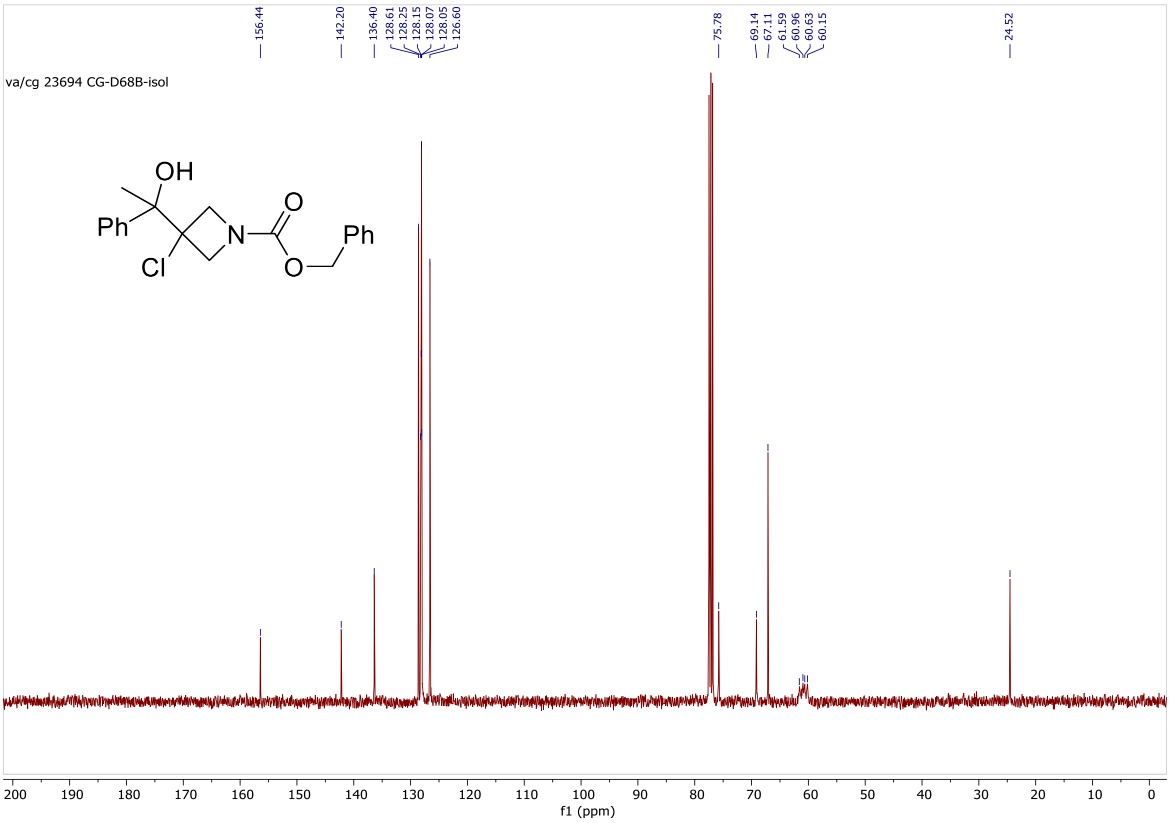

^{1}H NMR (CDCl$_3$, 400 MHz) of **11**, See procedure

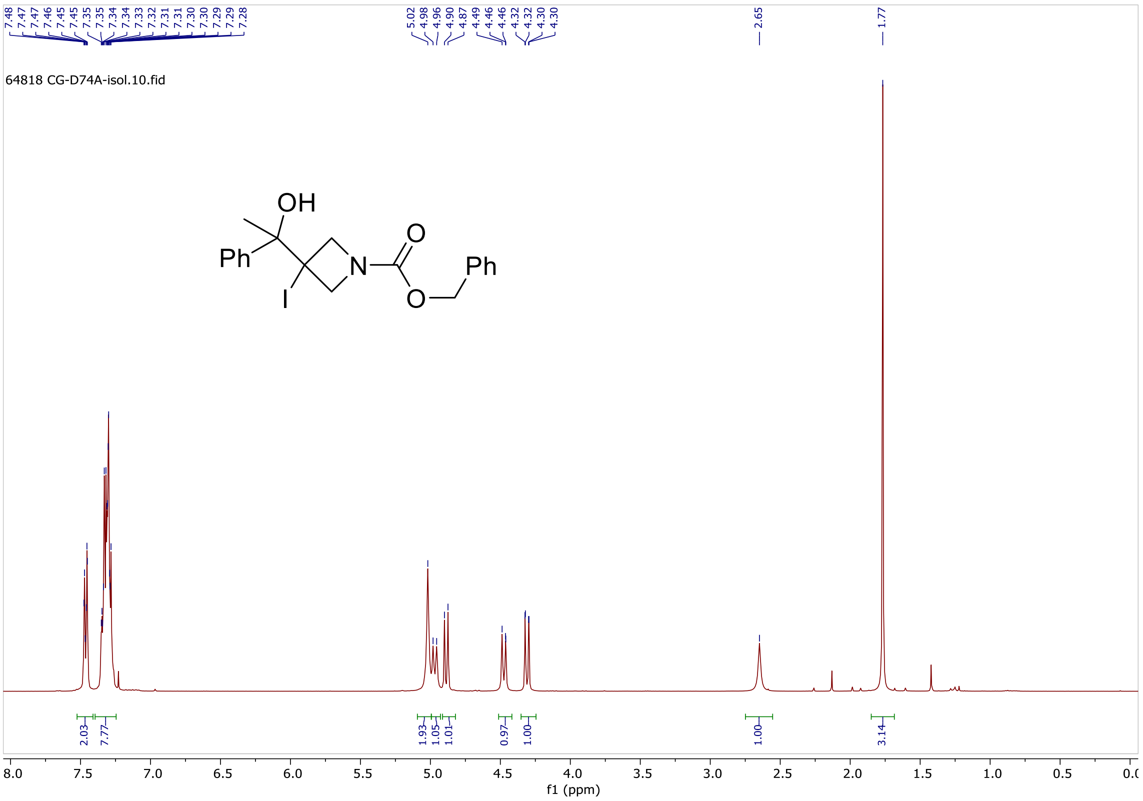

^{13}C NMR (CDCl$_3$, 101 MHz) of **11**

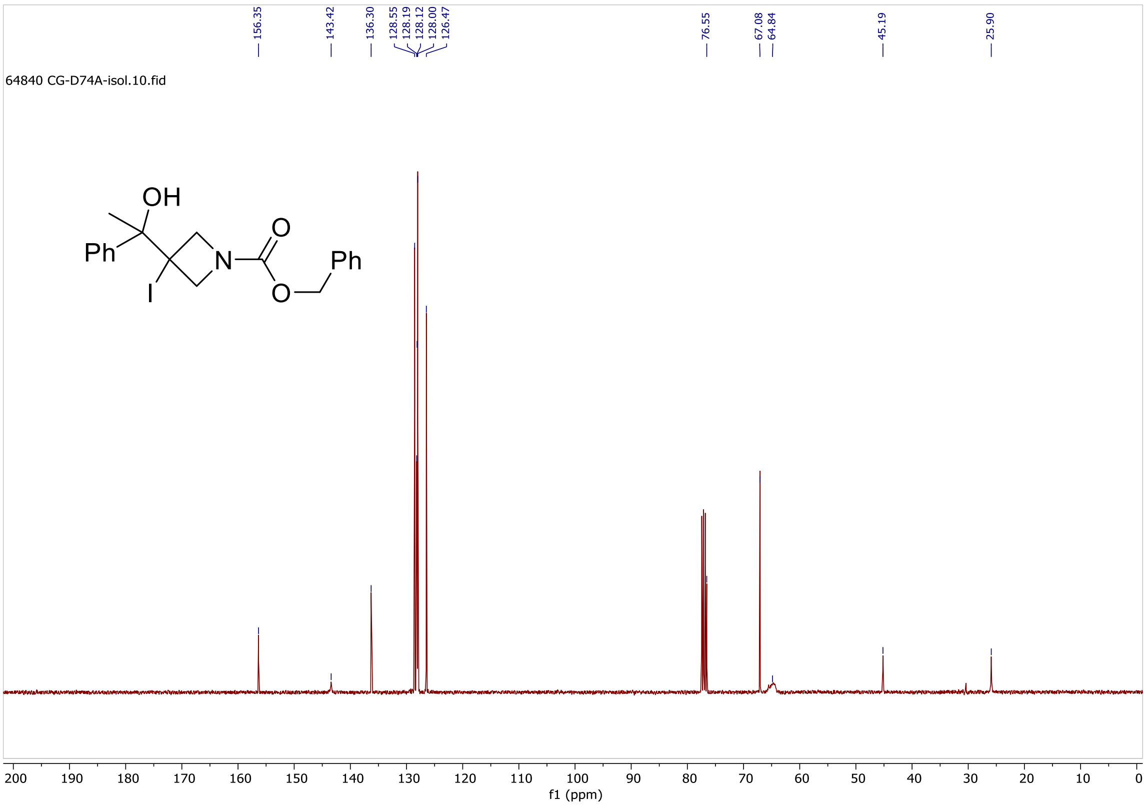

^{1}H NMR (CDCl$_3$, 400 MHz) of **12**, See procedure

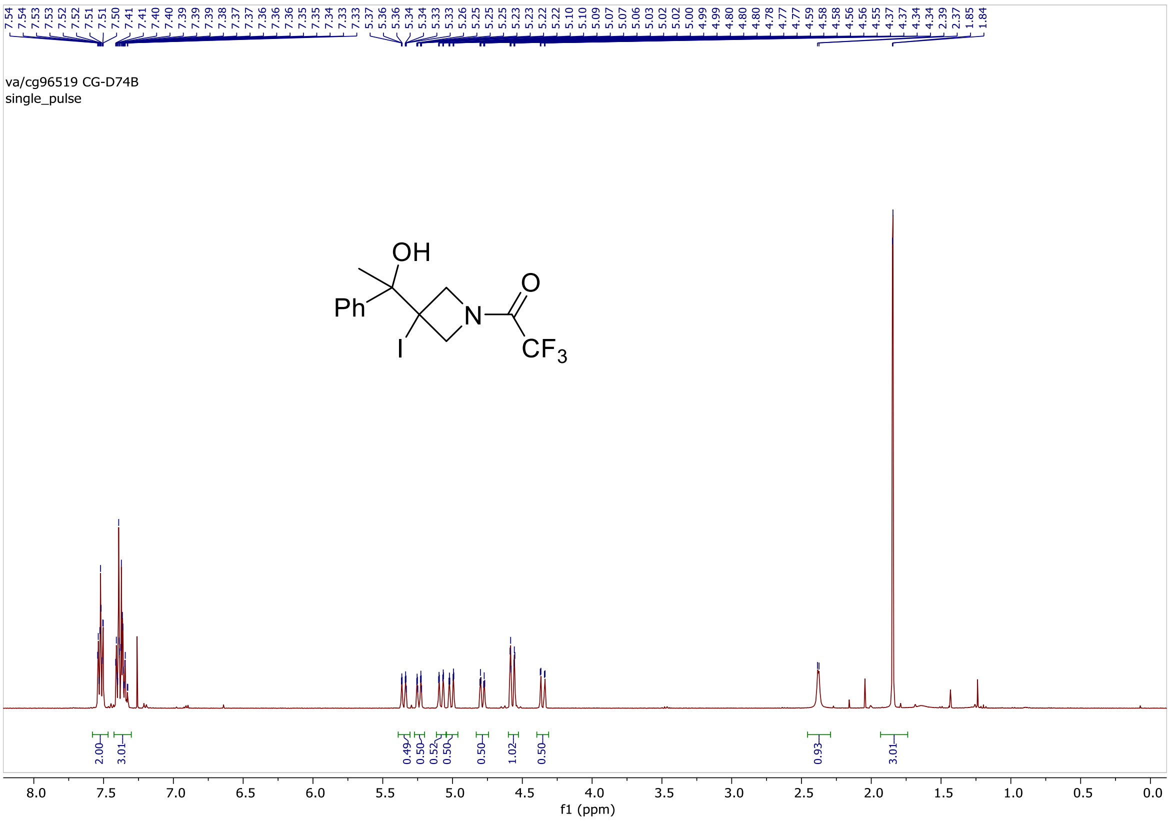

^{13}C NMR ($CDCl_3$, 101 MHz) of **12**

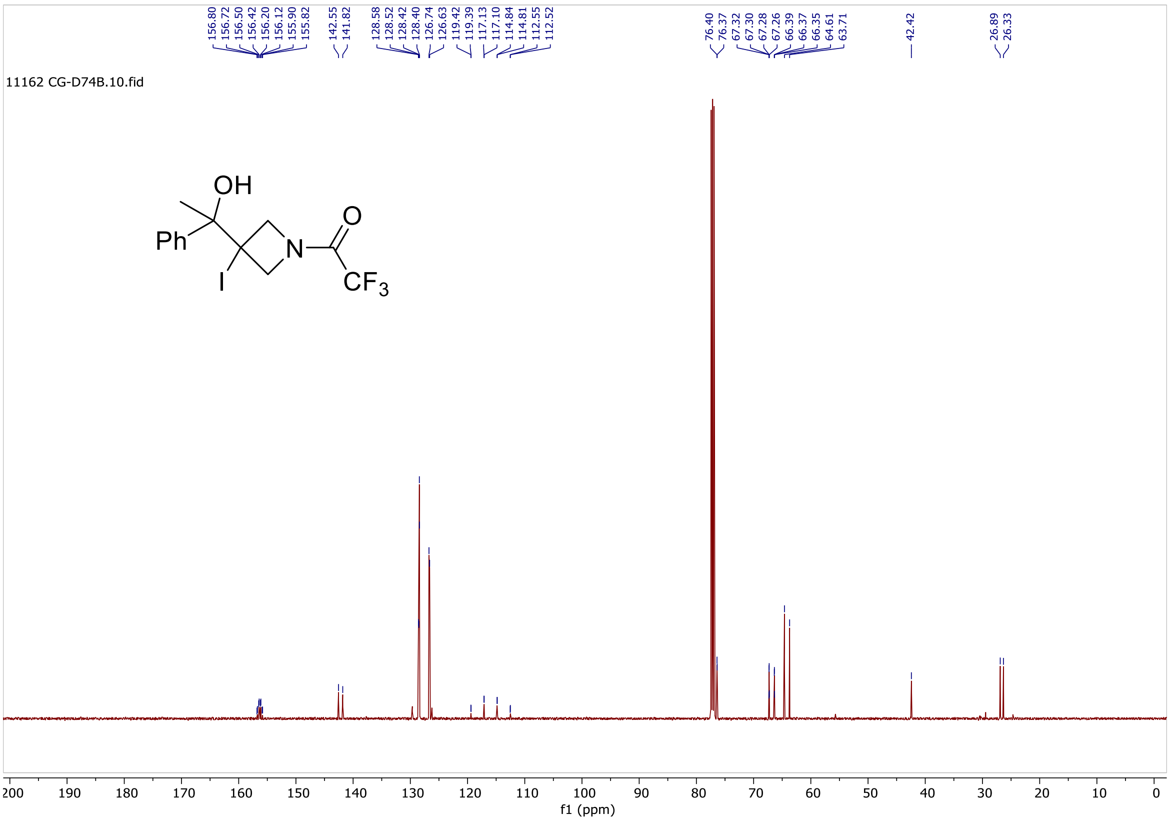

^{1}H NMR ($CDCl_3$, 400 MHz) of **6a**, See procedure

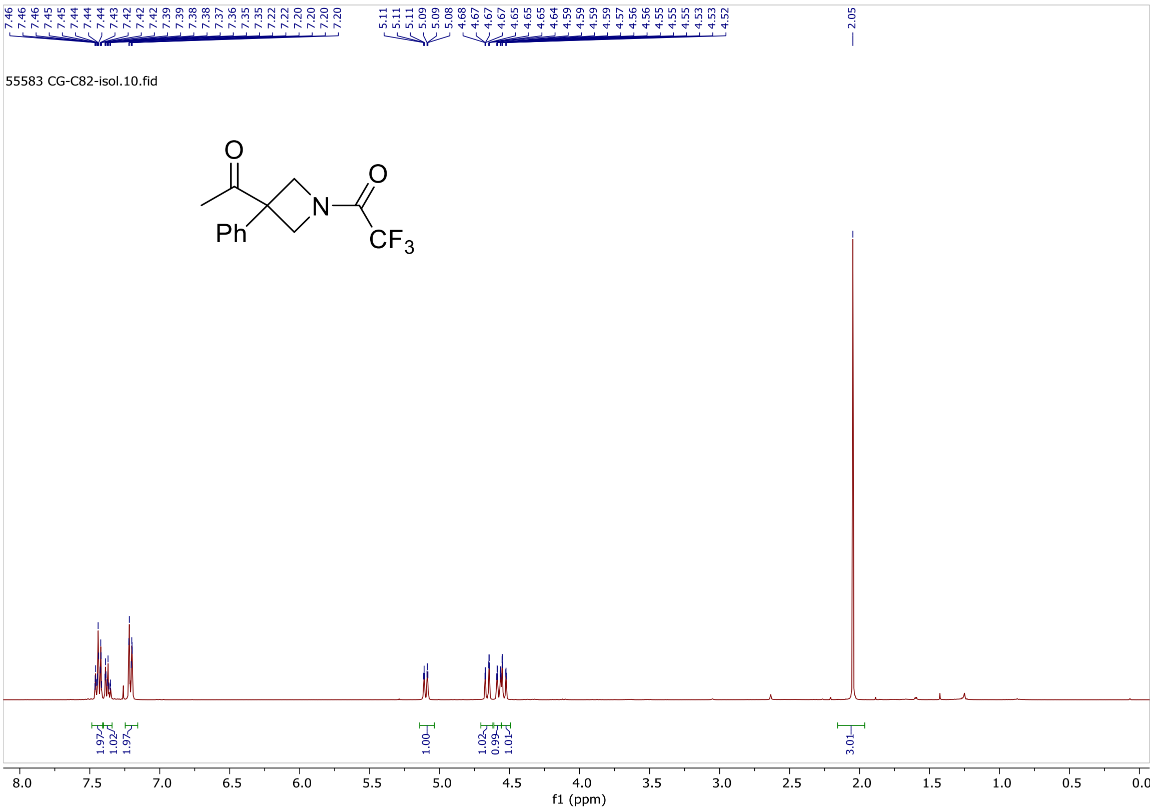

^{13}C NMR ($CDCl_3$, 101 MHz) of **6a**

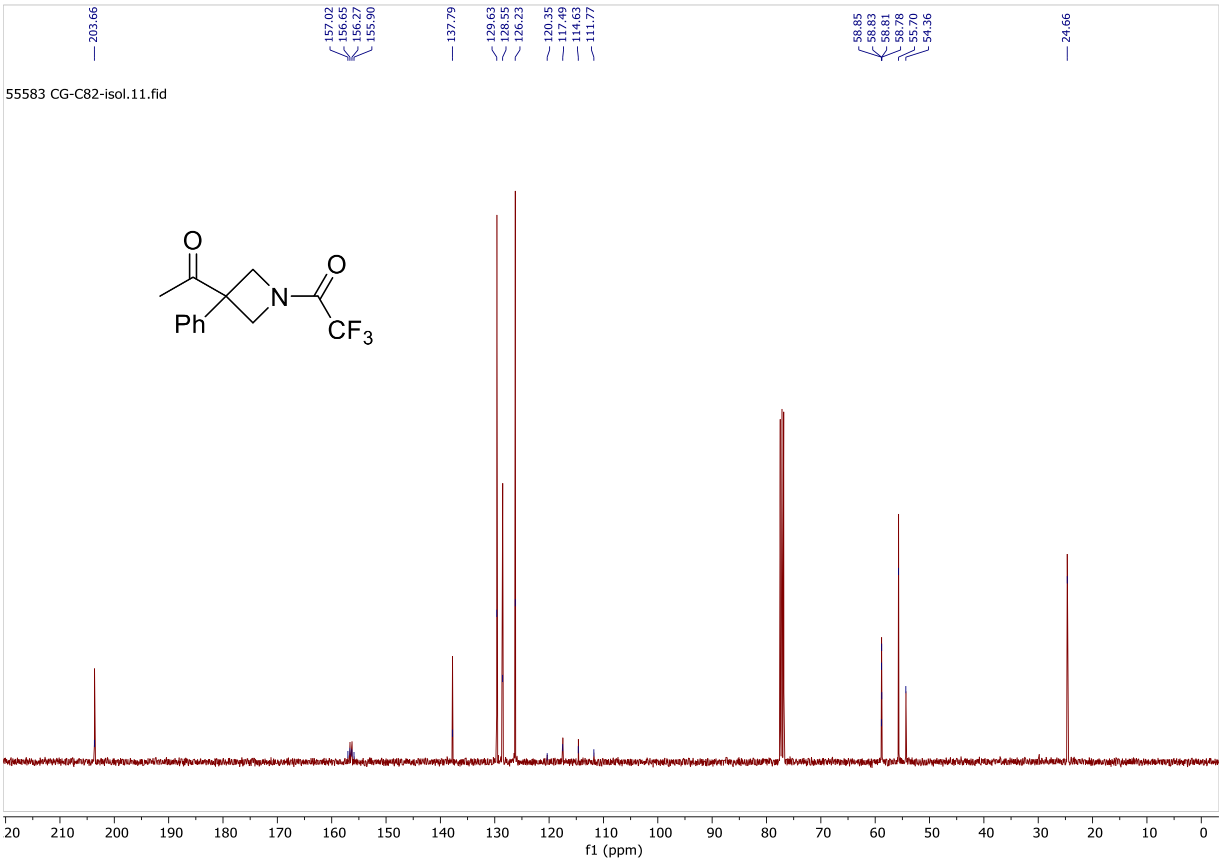

^{1}H NMR ($CDCl_3$, 400 MHz) of **7a**, See procedure

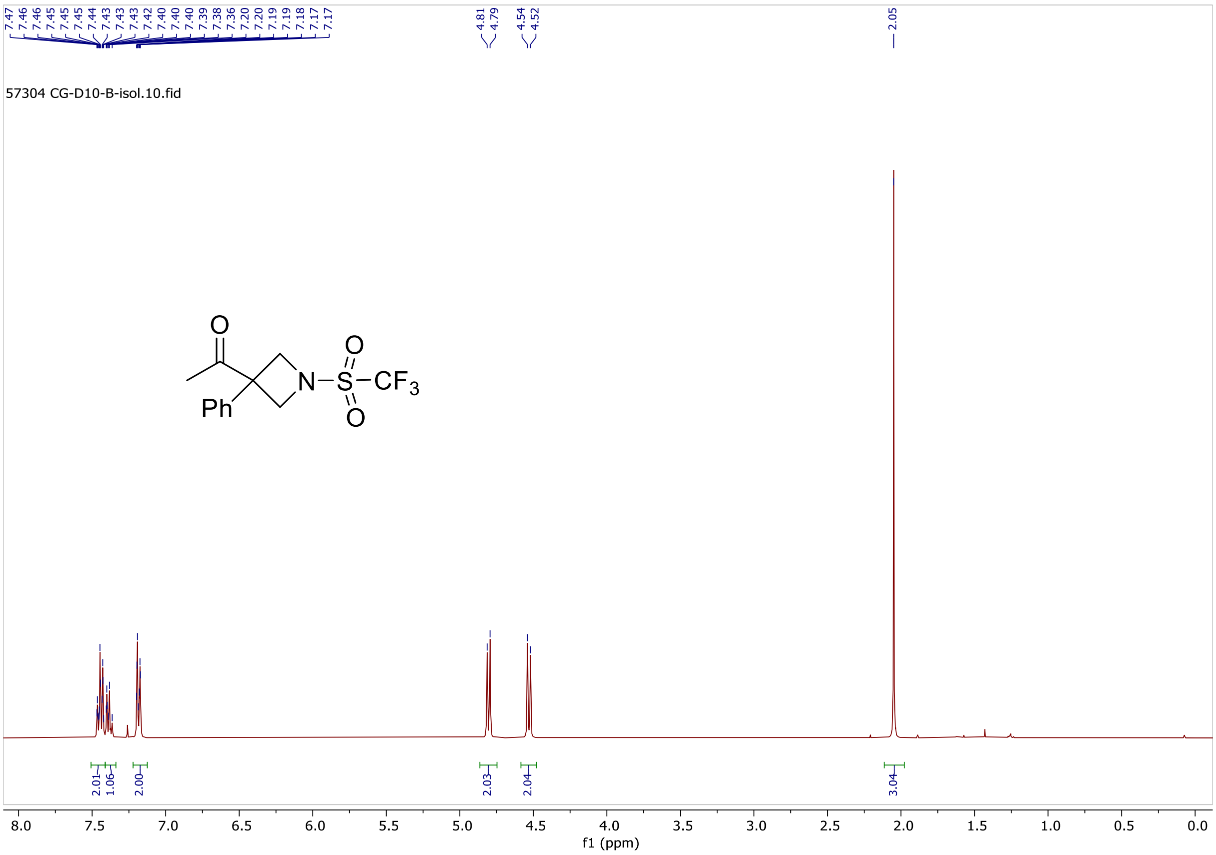

^{13}C NMR ($CDCl_3$, 101 MHz) of **7a**

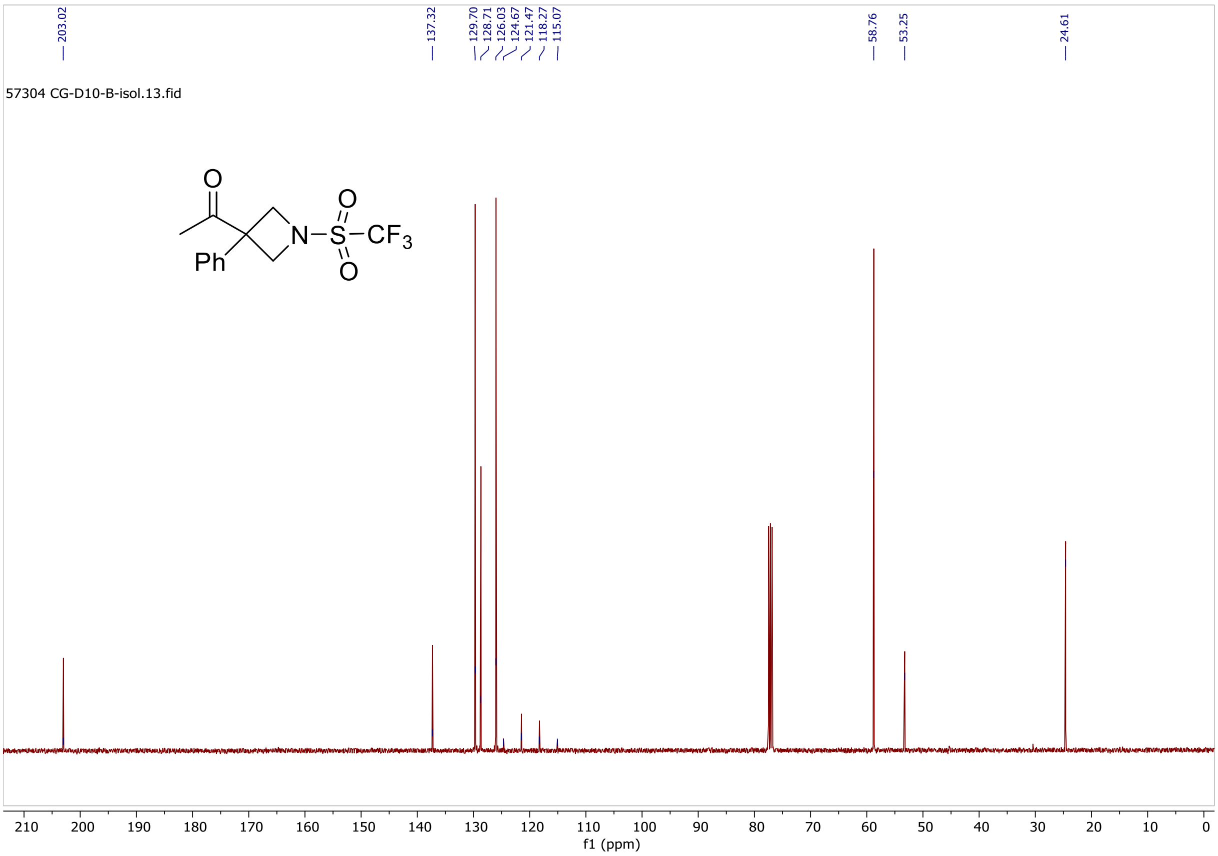

^{1}H NMR ($CDCl_3$, 400 MHz) of **6b**, See procedure

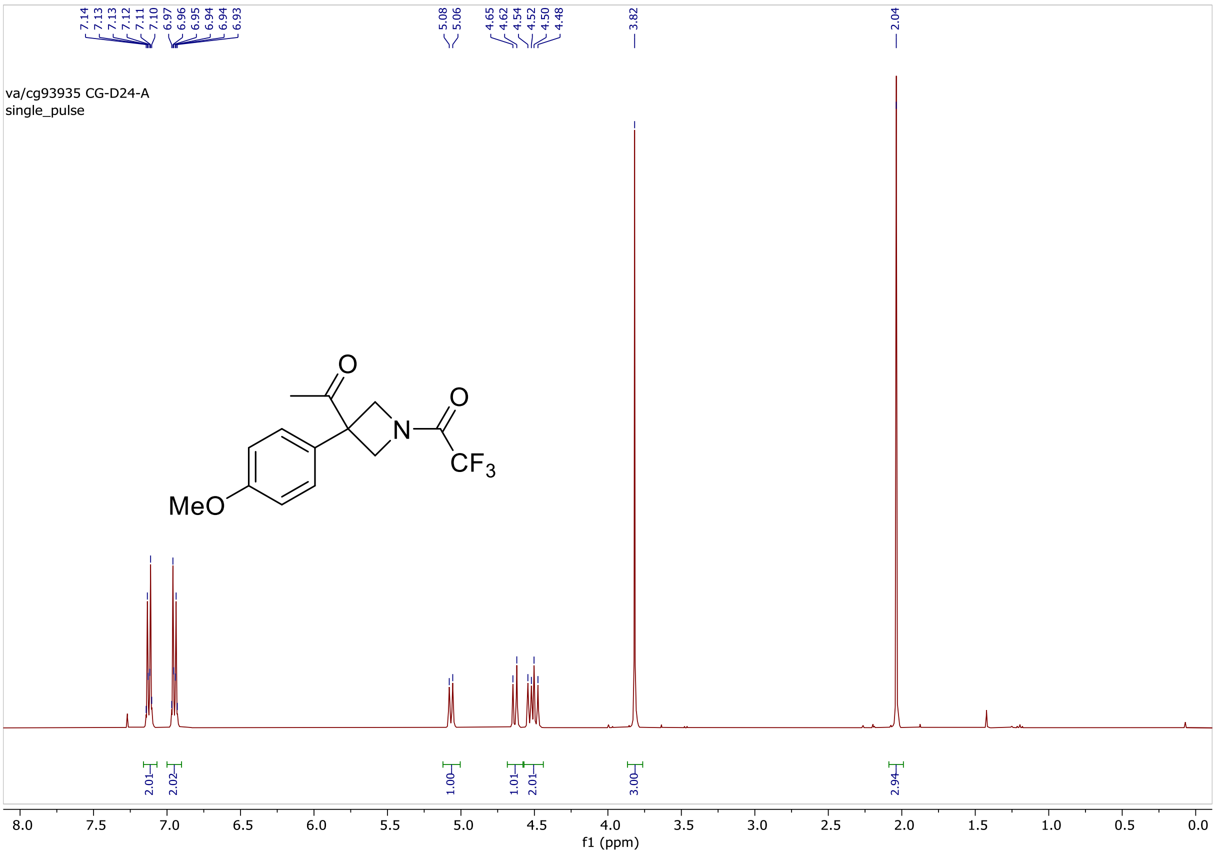

^{13}C NMR (CDCl$_3$, 101 MHz) of **6b**

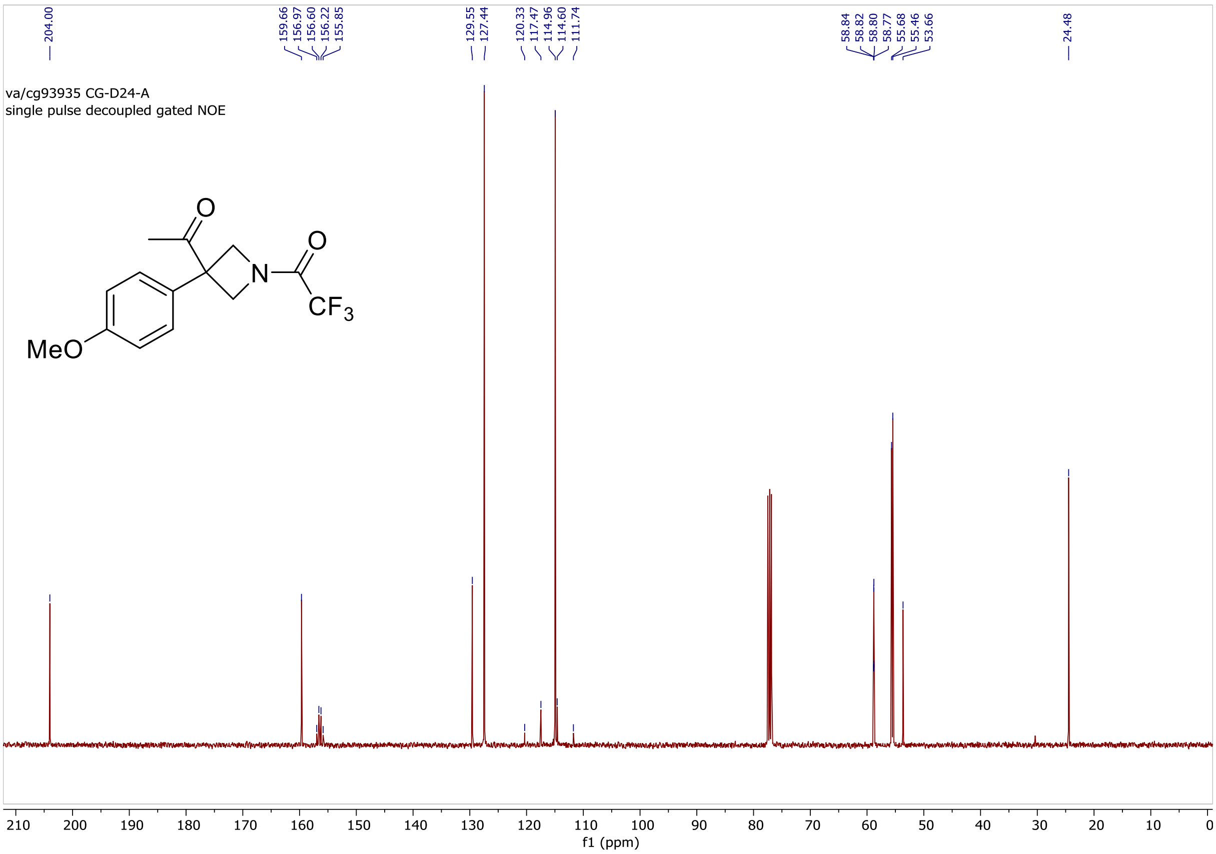

^{1}H NMR (CDCl$_3$, 400 MHz) of **7b**, See procedure

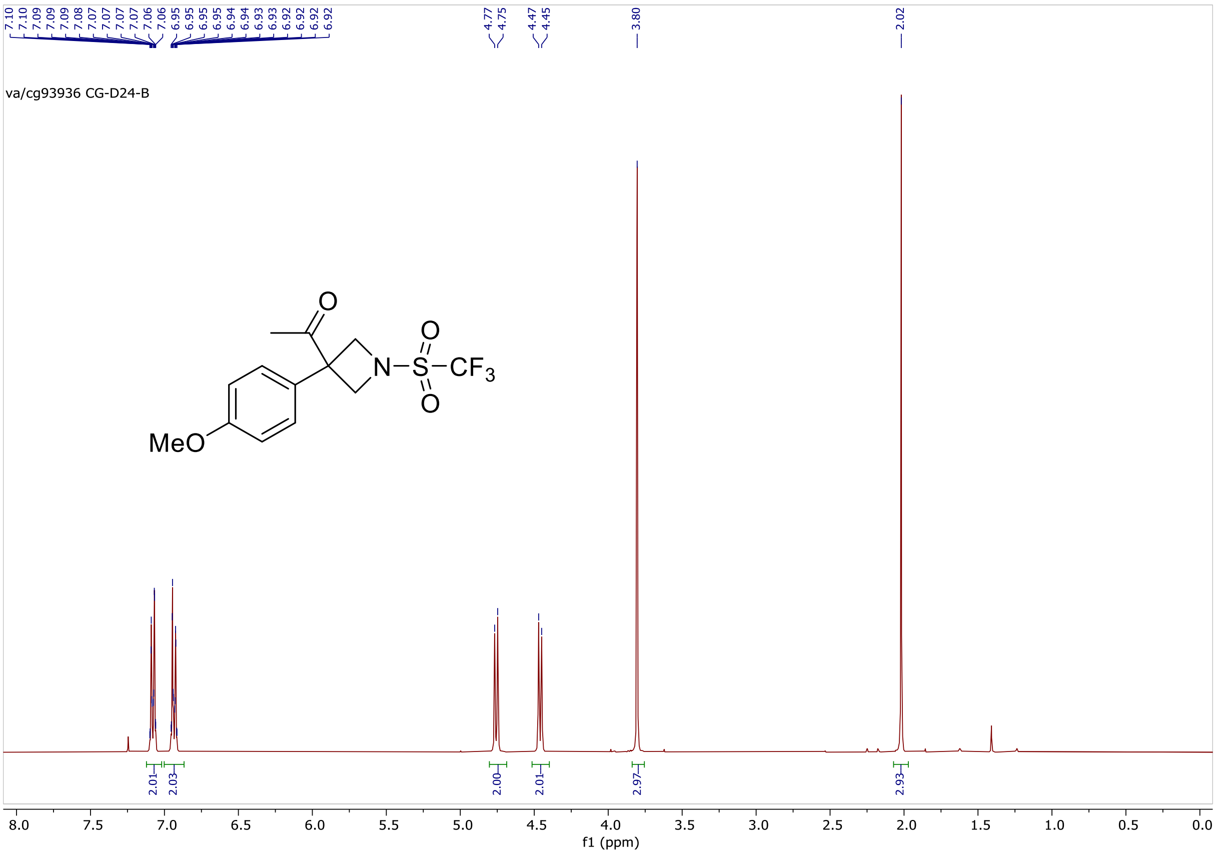

^{13}C NMR ($CDCl_3$, 101 MHz) of **7b**

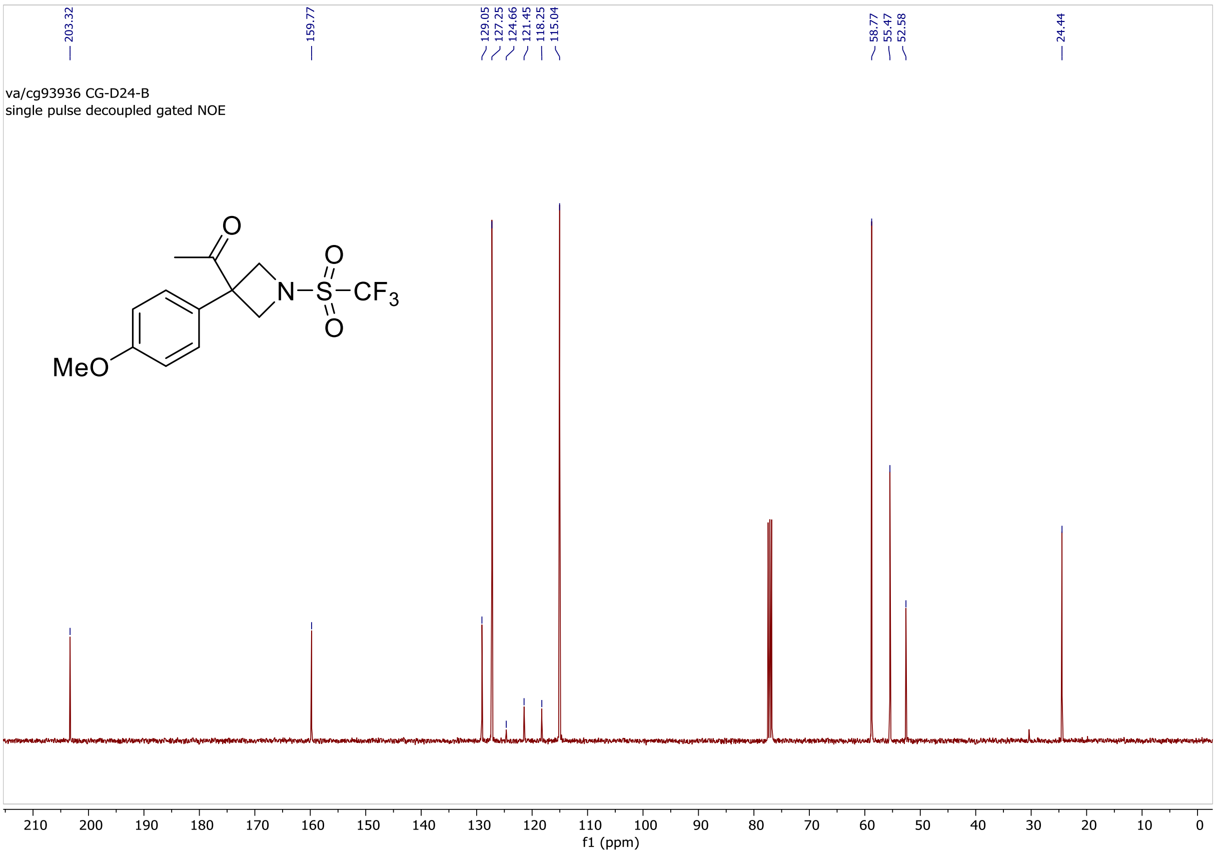

^{1}H NMR ($CDCl_3$, 400 MHz) of **6c**, See procedure

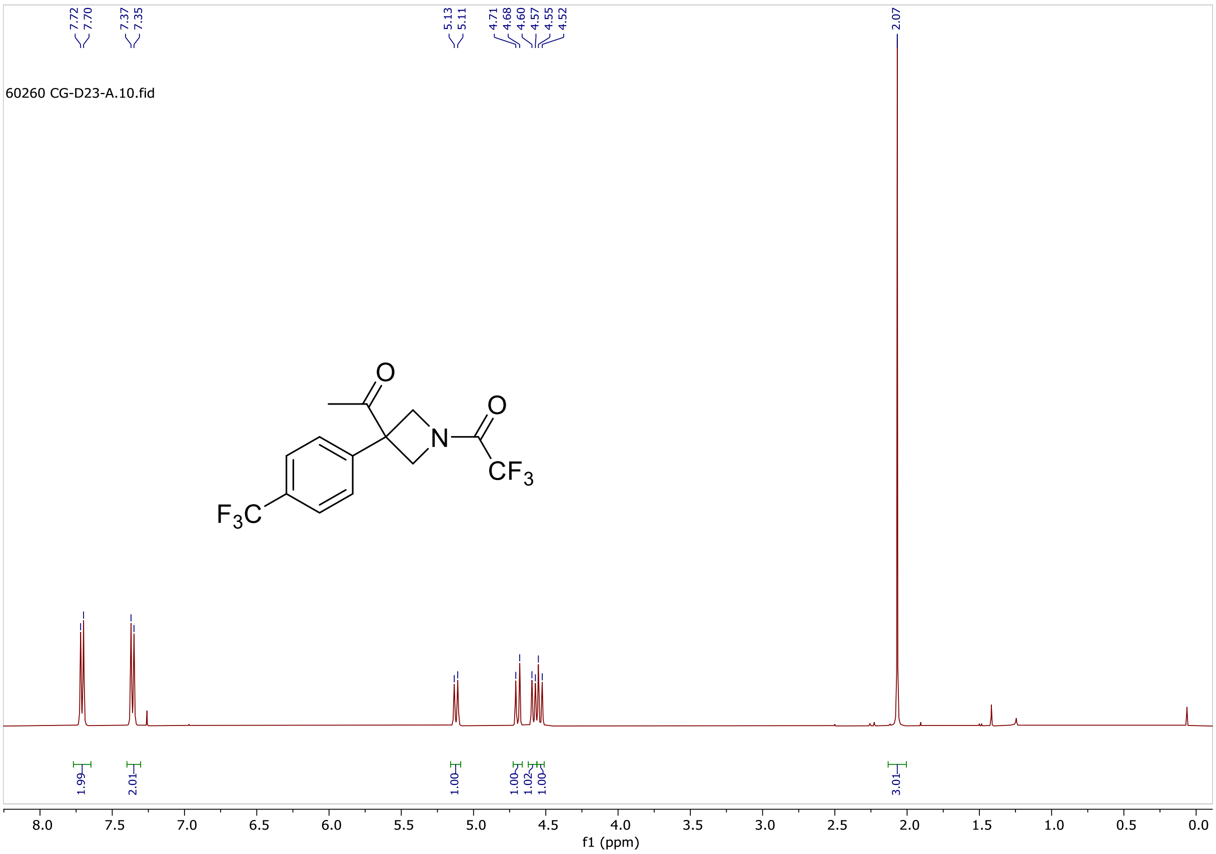

[13] **C NMR** ($CDCl_3$, 101 MHz) of **6c**

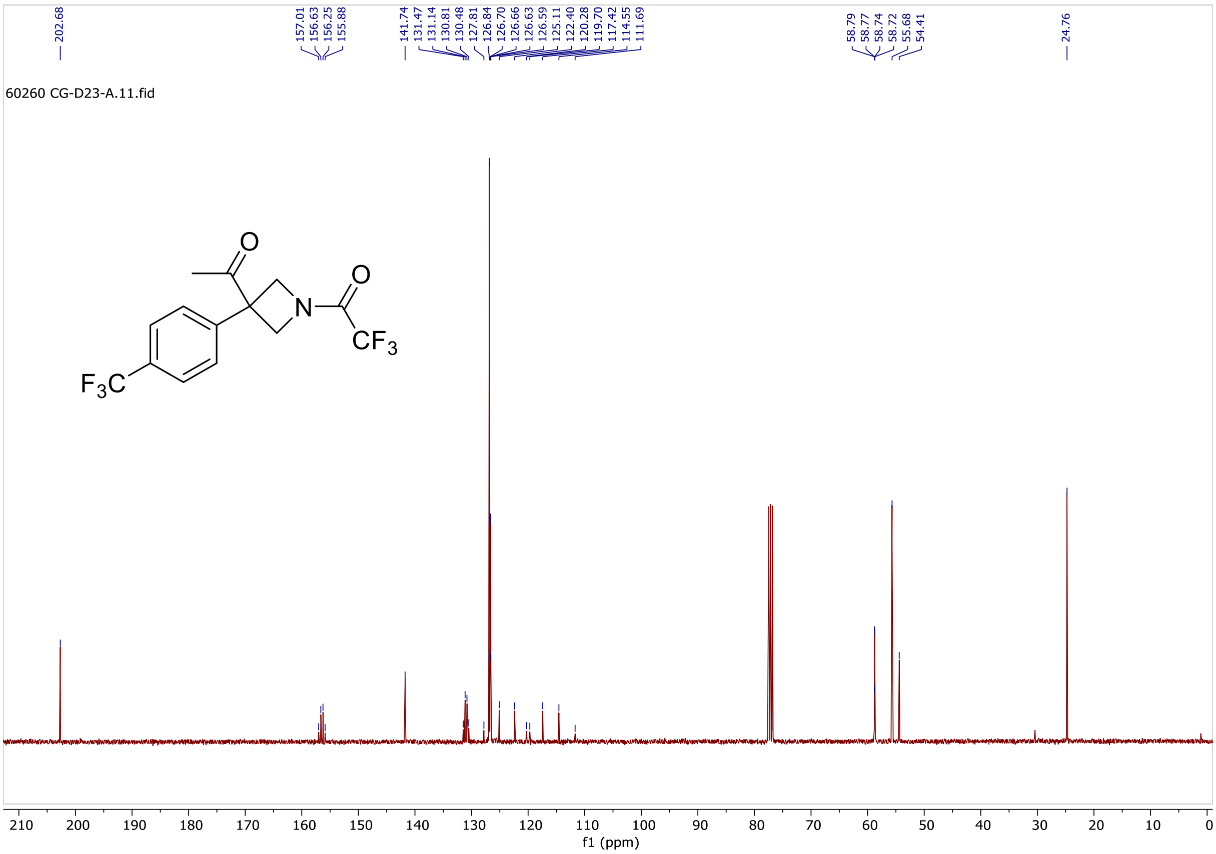

[1] **H NMR** ($CDCl_3$, 400 MHz) of **7c**, See procedure

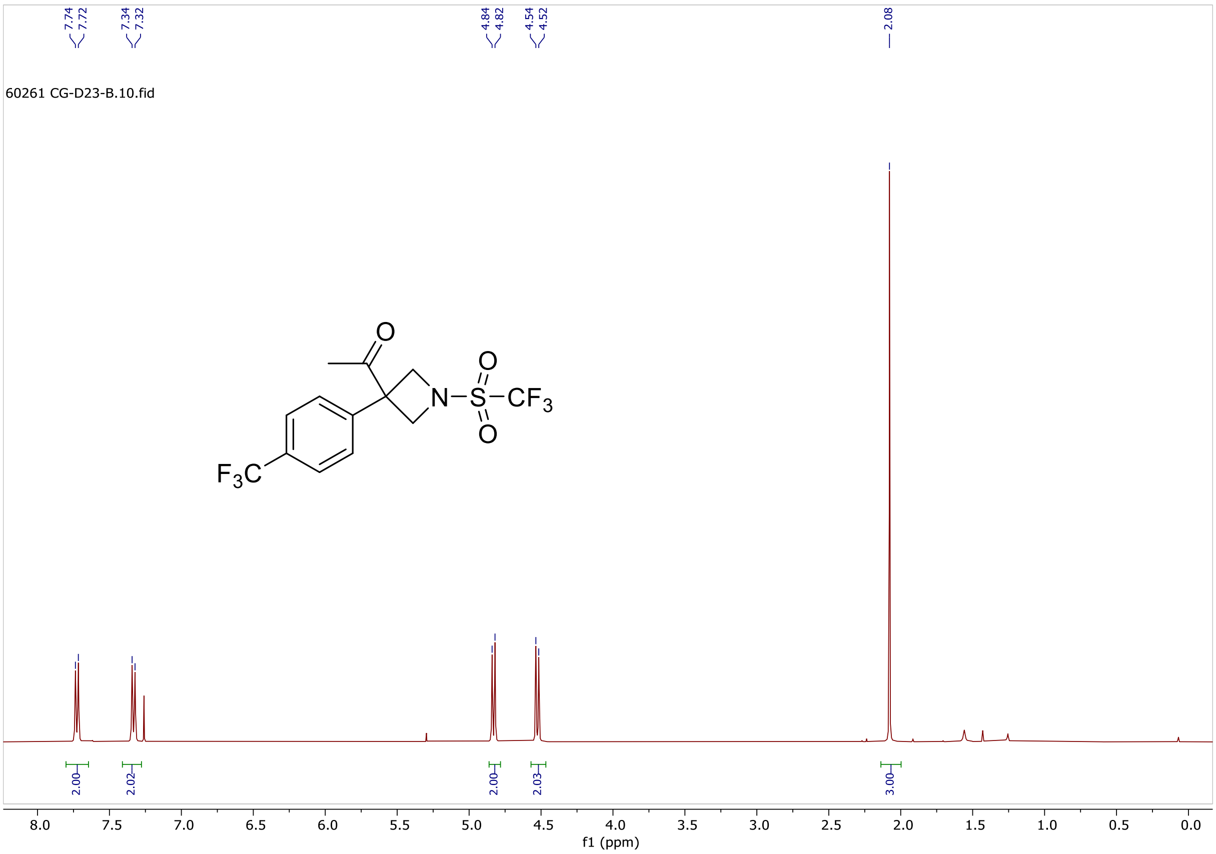

^{13}**C NMR** (CDCl$_3$, 101 MHz) of **7c**

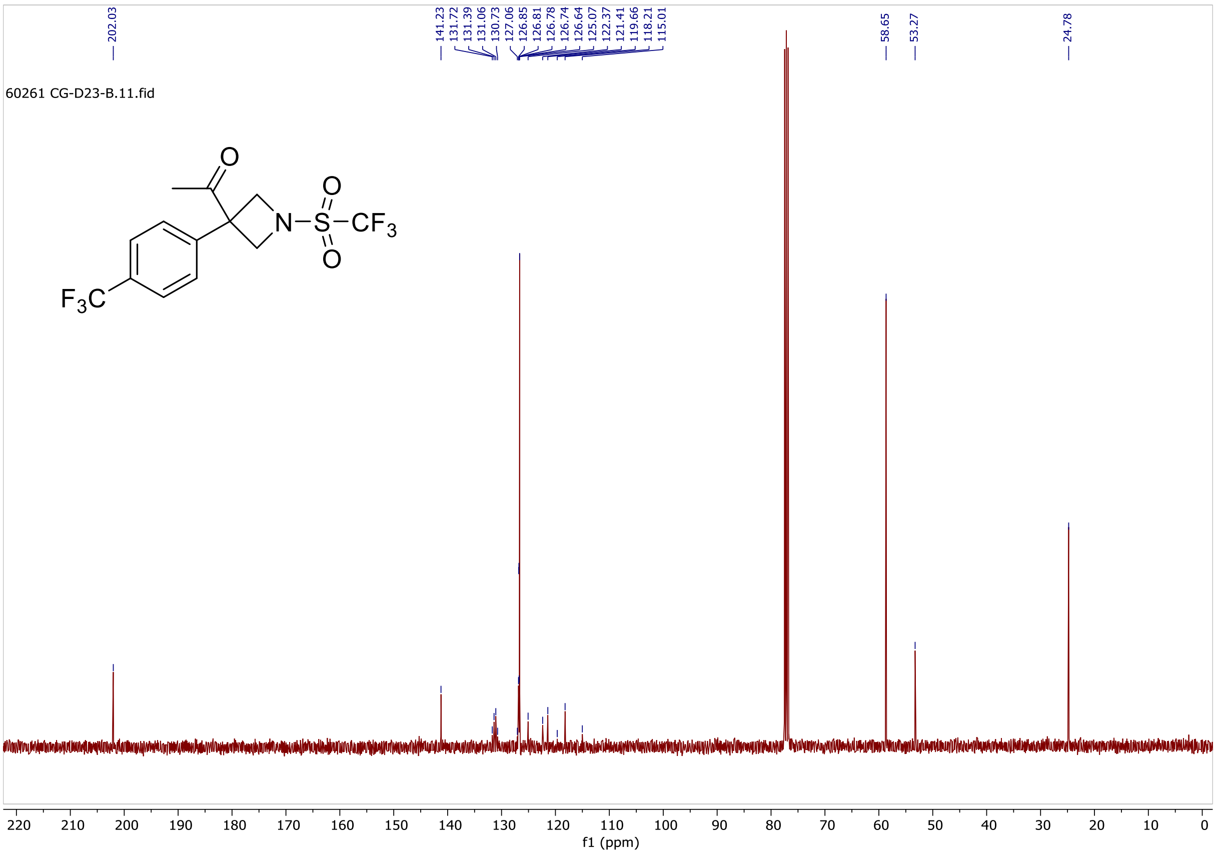

^{1}**H NMR** (CDCl$_3$, 400 MHz) of **6d**, See procedure

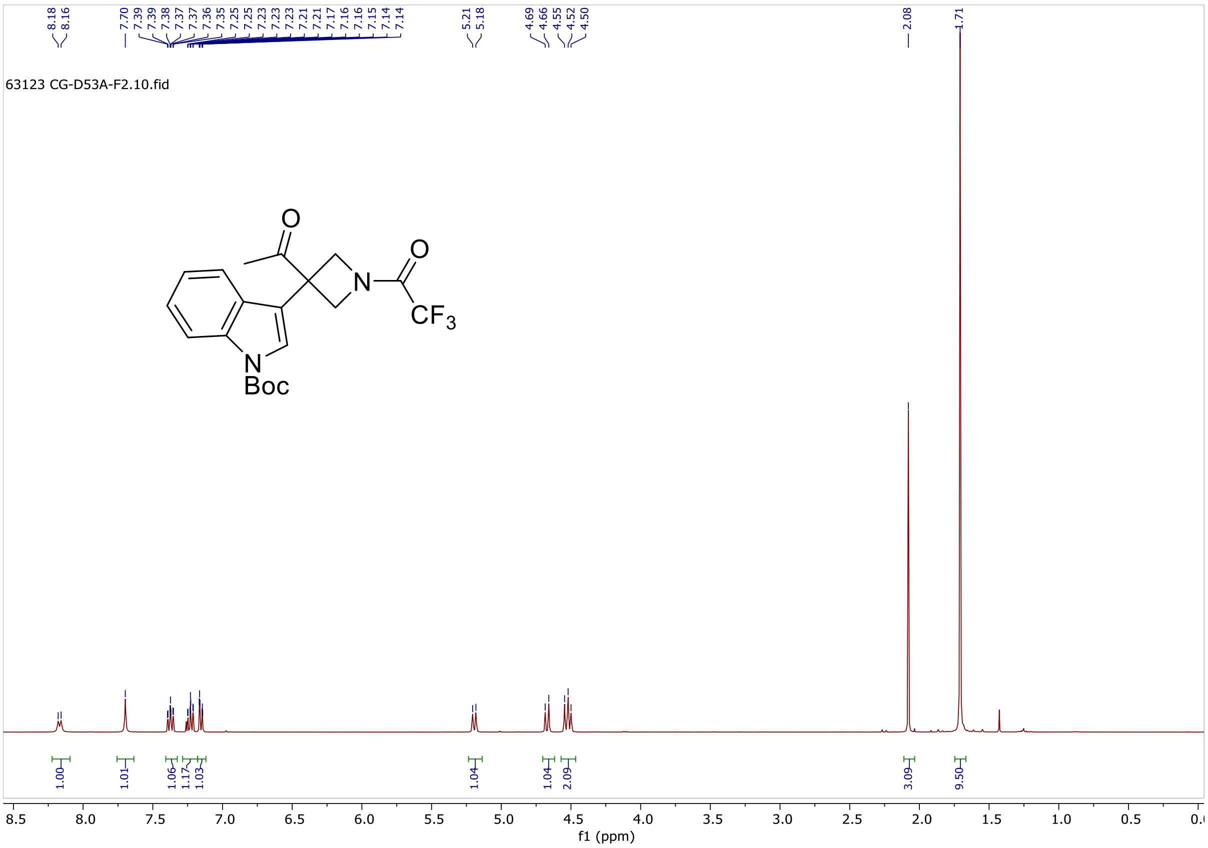

^{13}C NMR ($CDCl_3$, 101 MHz) of **6d**

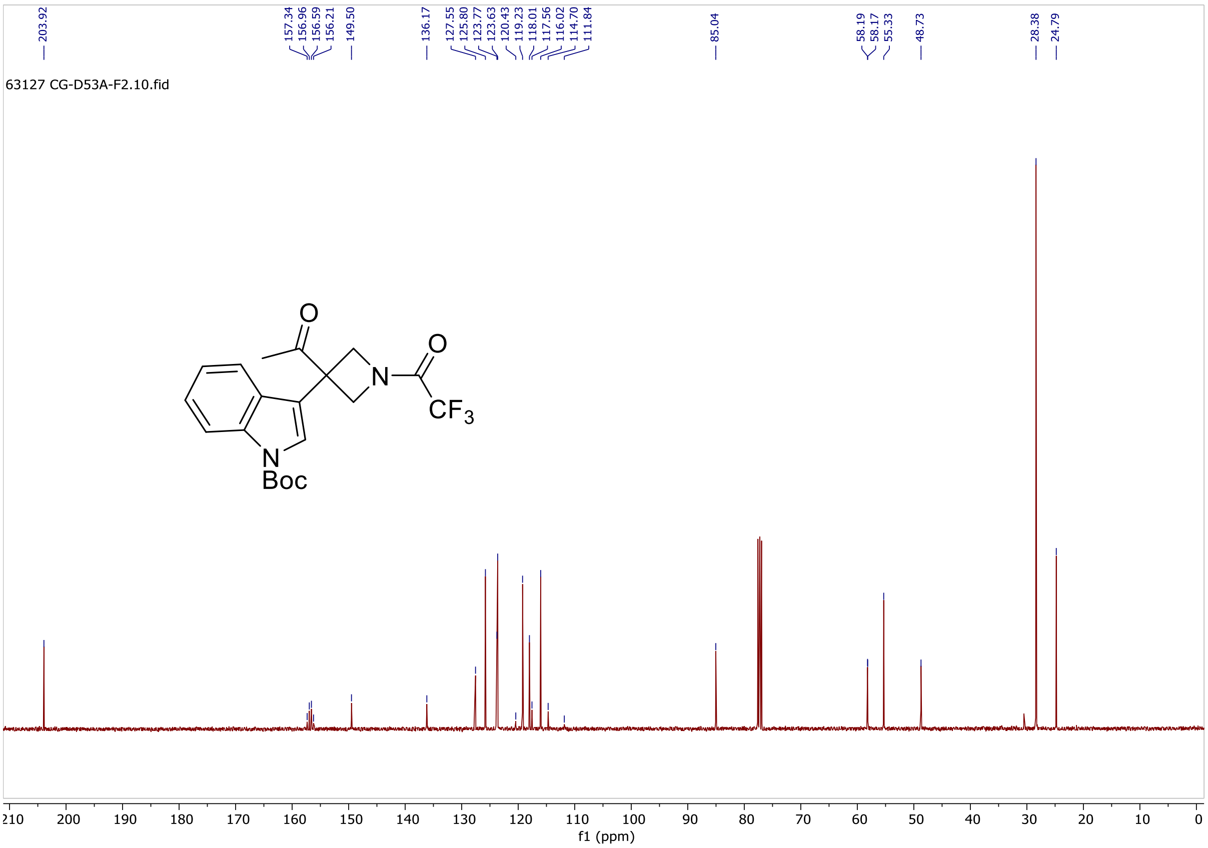

^{1}H NMR ($CDCl_3$, 400 MHz) of **7d**, See procedure

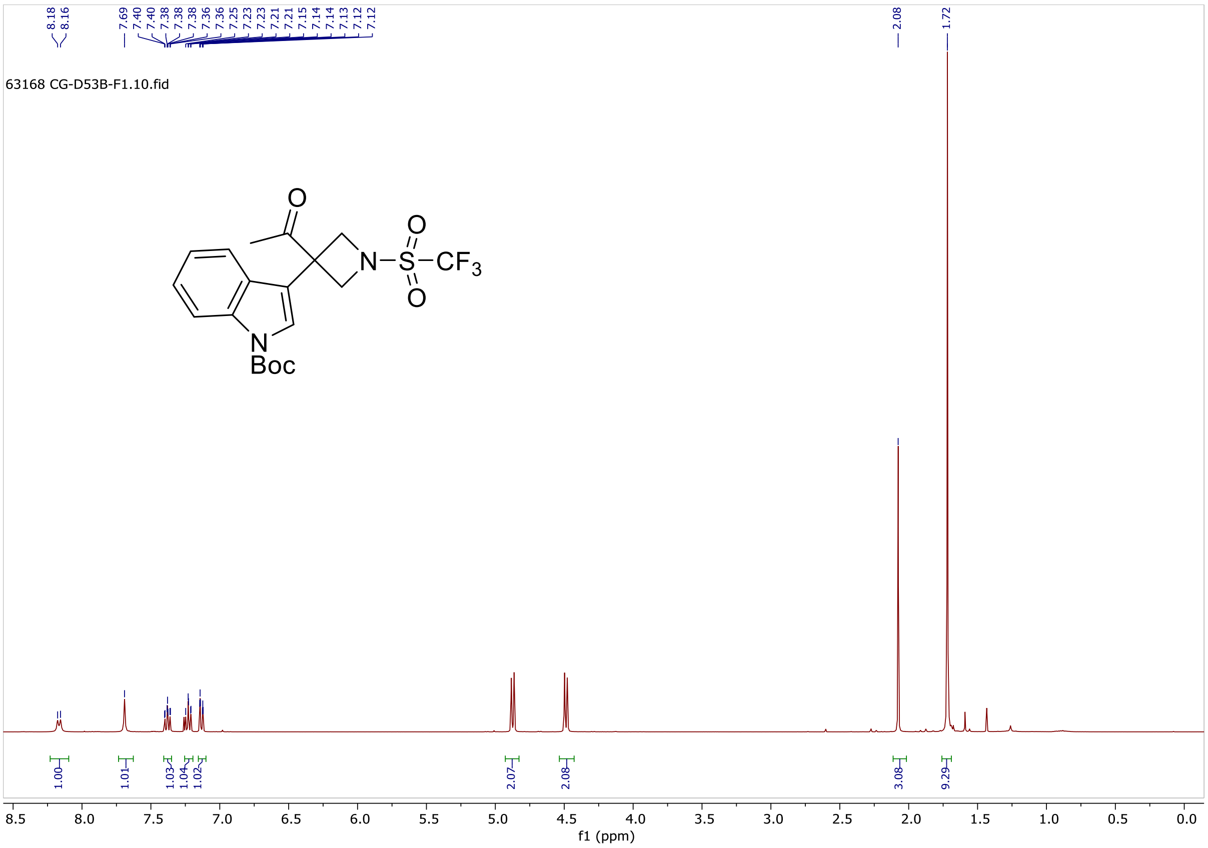

^{13}C NMR ($CDCl_3$, 101 MHz) of **7d**

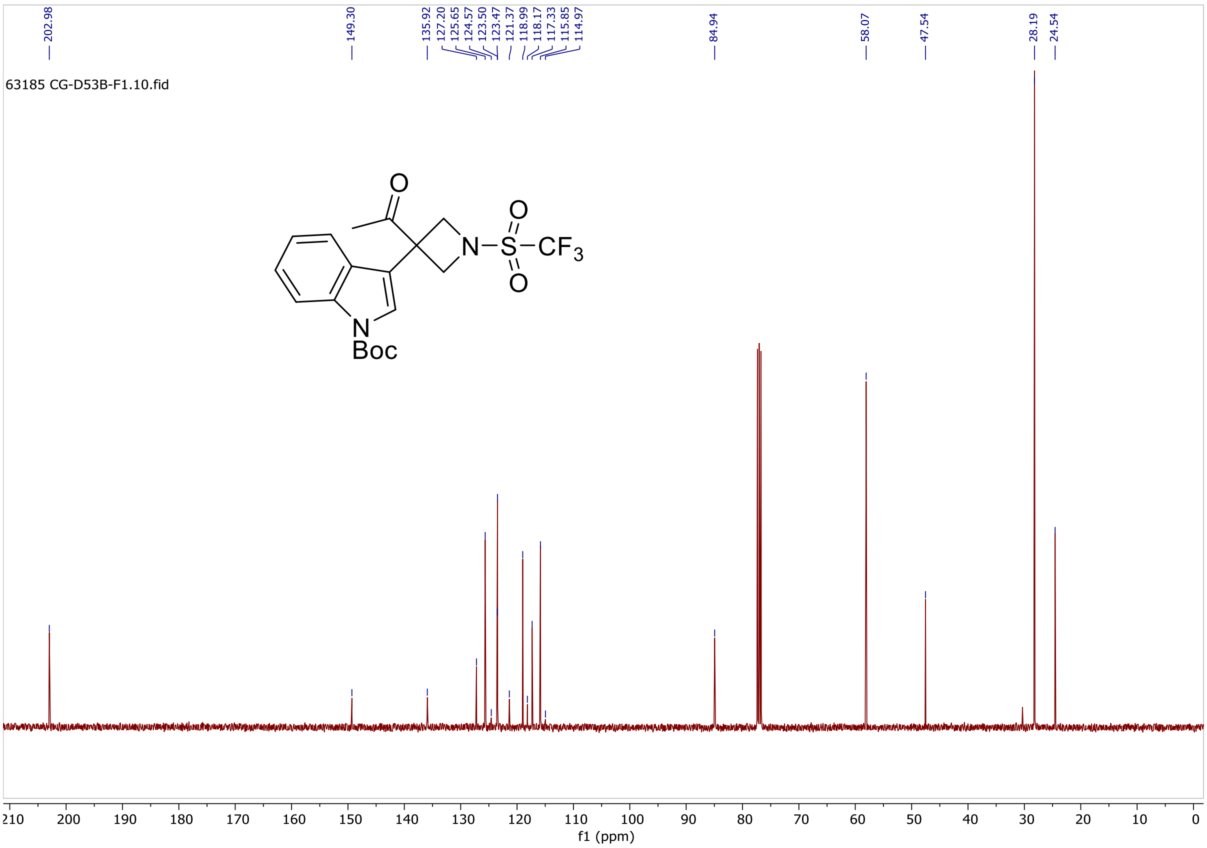

^{1}H NMR ($CDCl_3$, 400 MHz) of **6e**, See procedure

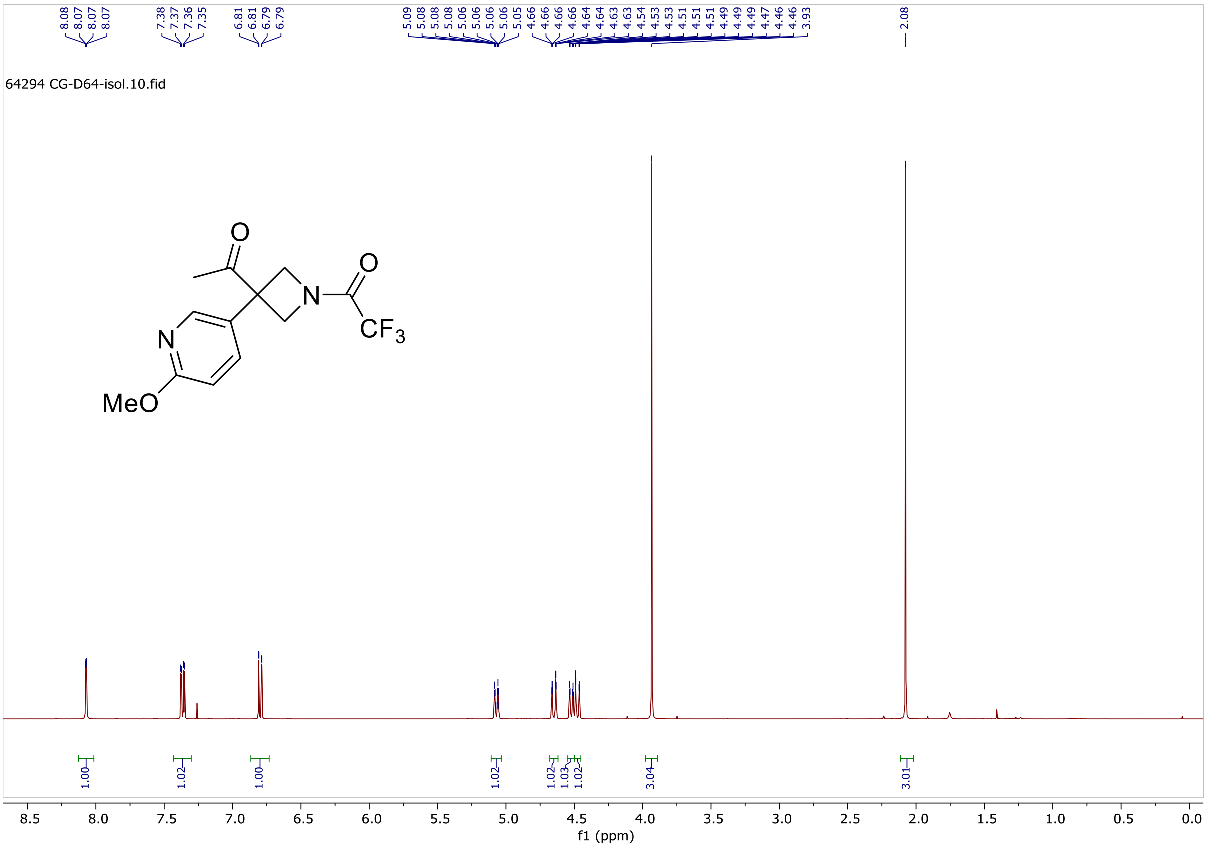

^{13}C NMR (CDCl3, 101 MHz) of **6e**

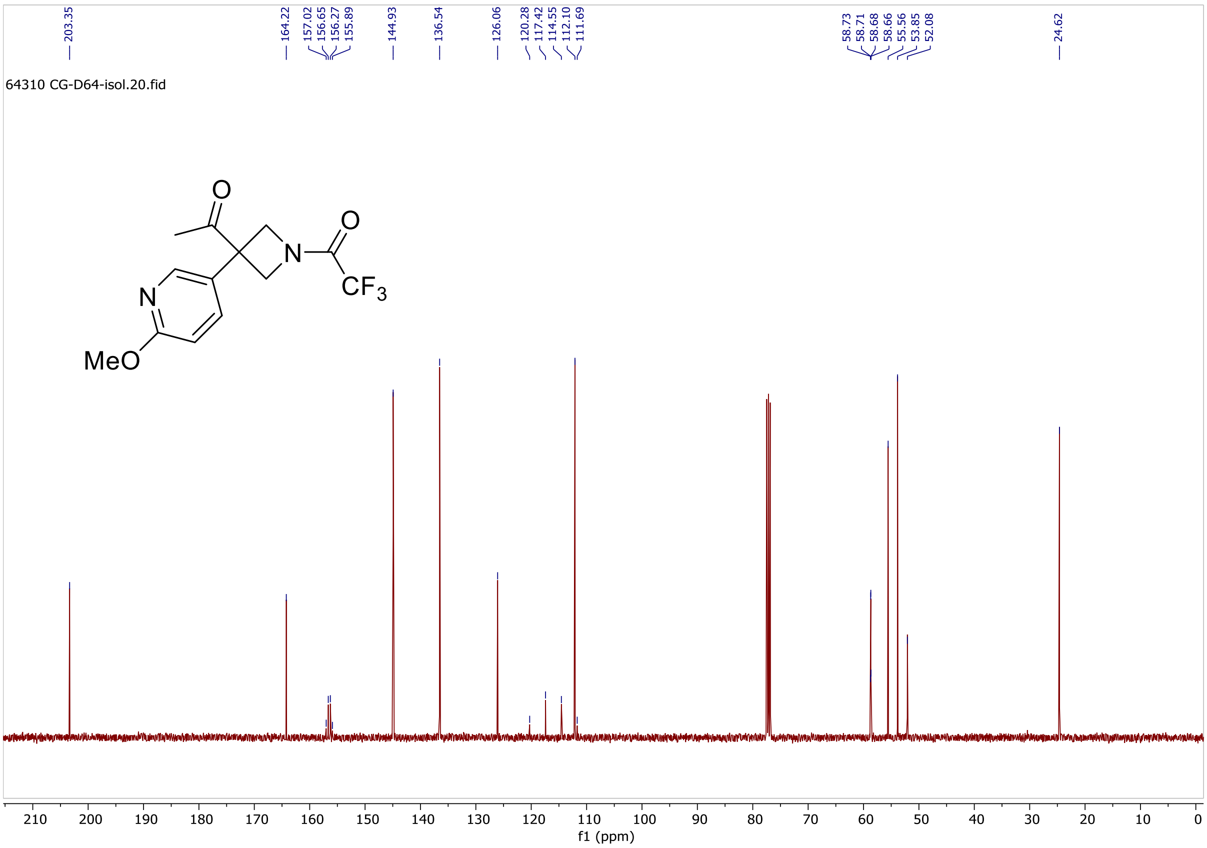

^{1}H NMR (CDCl3, 400 MHz) of **7e**, See procedure

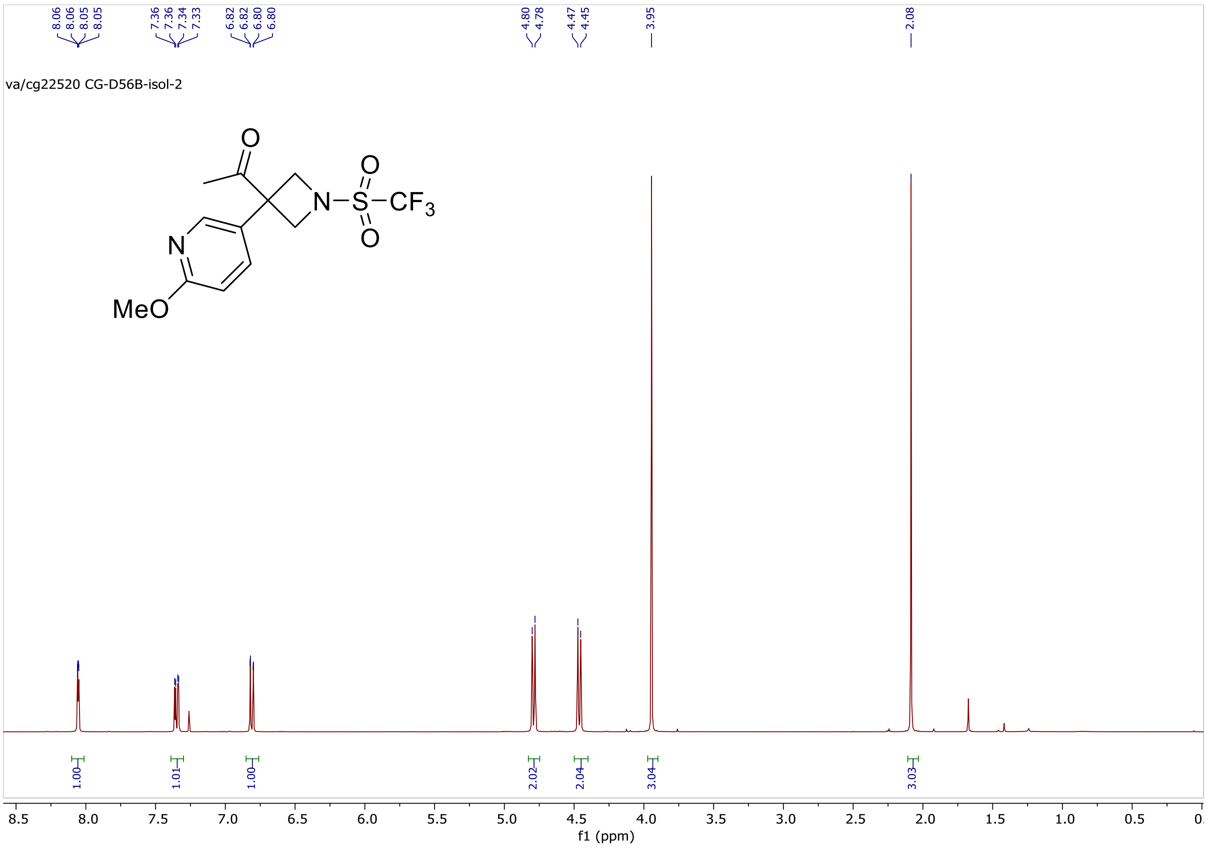

^{13}C NMR ($CDCl_3$, 101 MHz) of **7e**

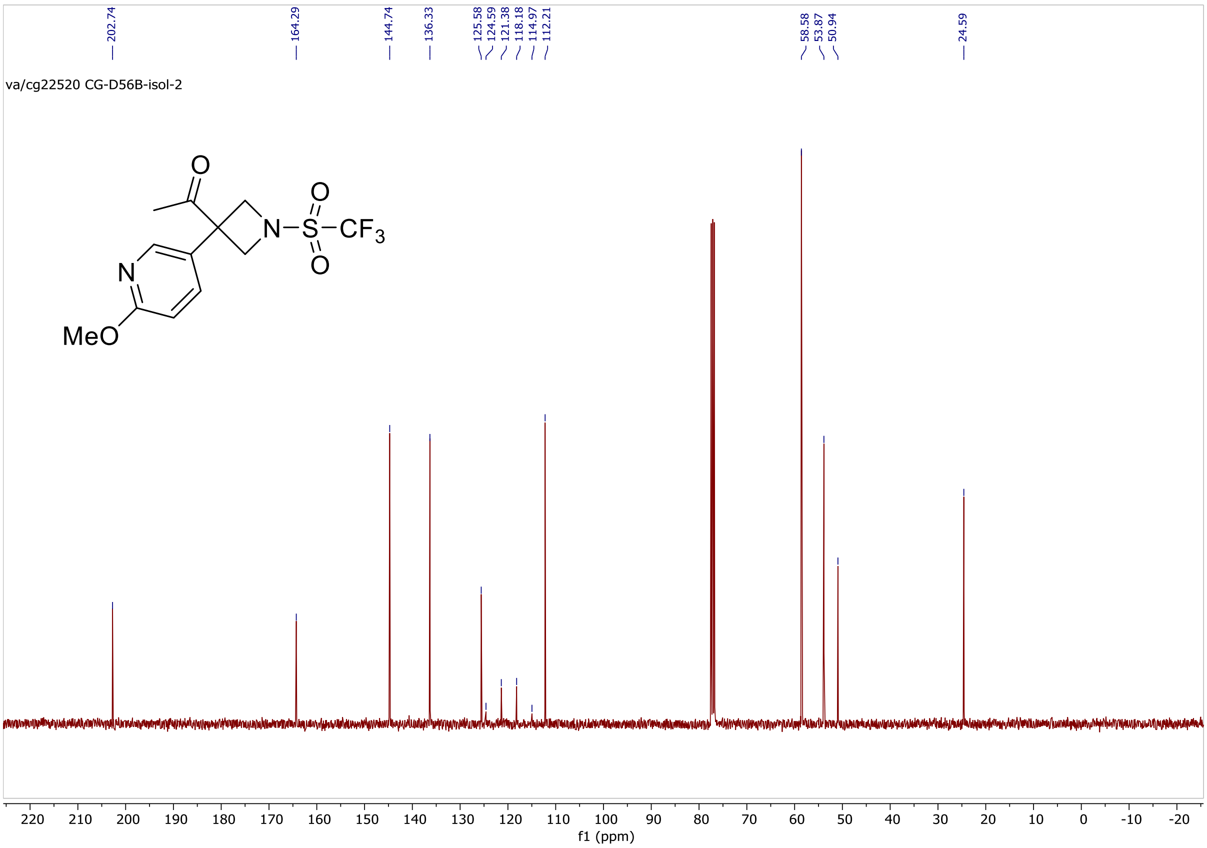

^{1}H NMR ($CDCl_3$, 400 MHz) of **6f**, See procedure

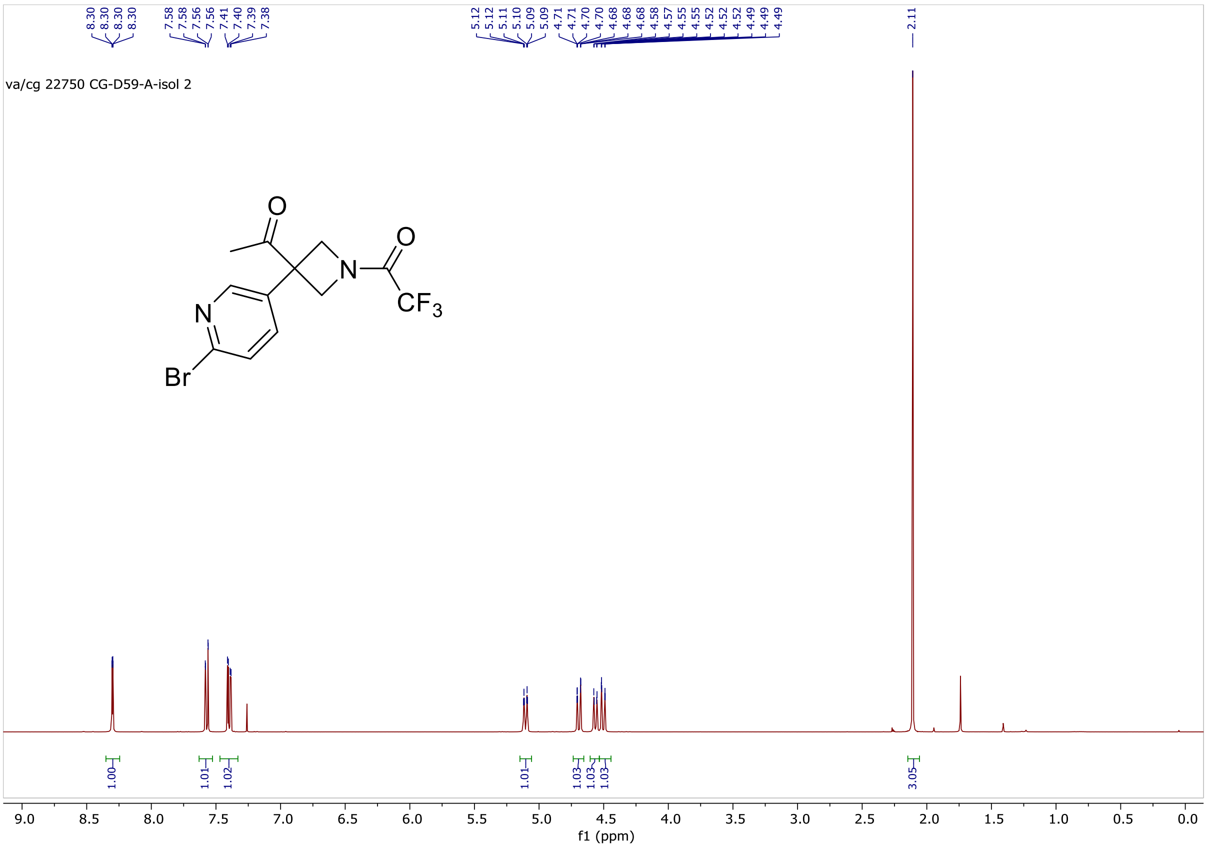

^{13}C NMR ($CDCl_3$, 101 MHz) of **6f**

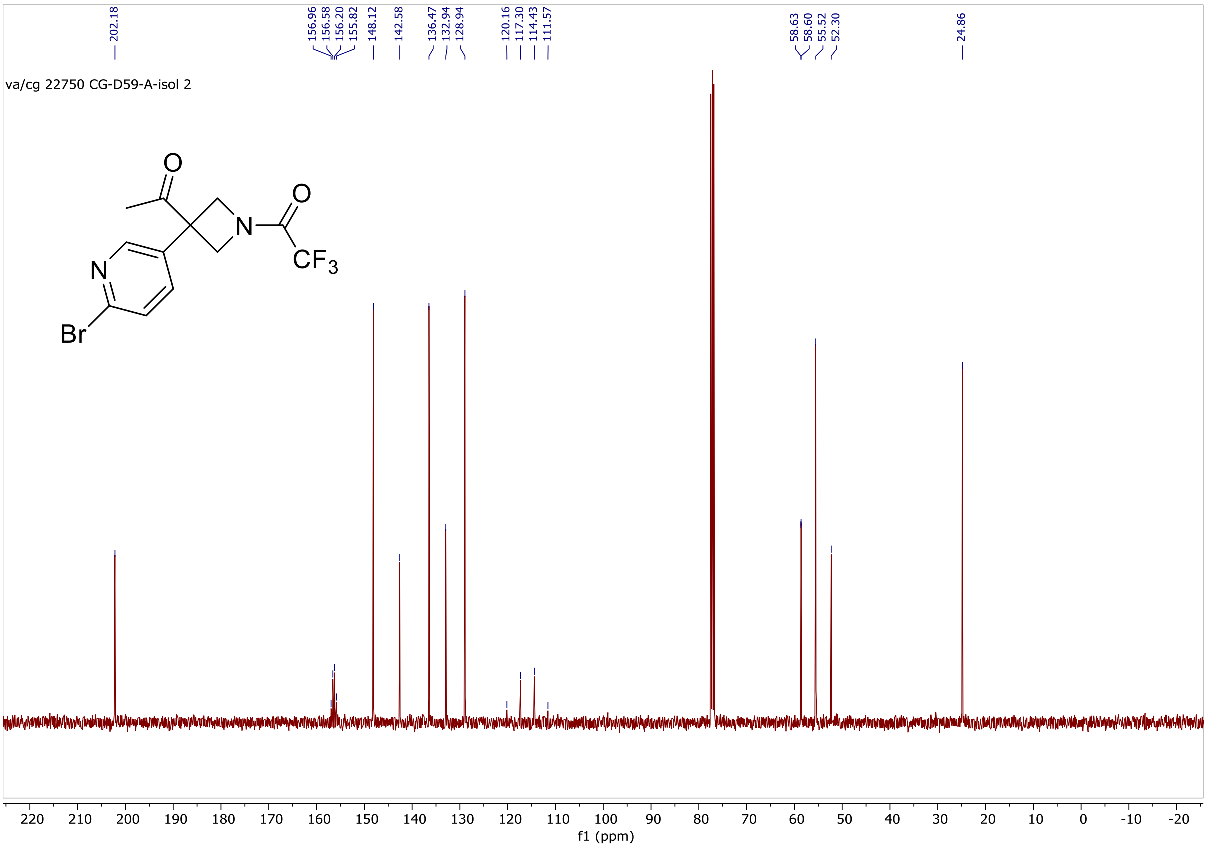

^{1}H NMR ($CDCl_3$, 400 MHz) of **7f**, See procedure

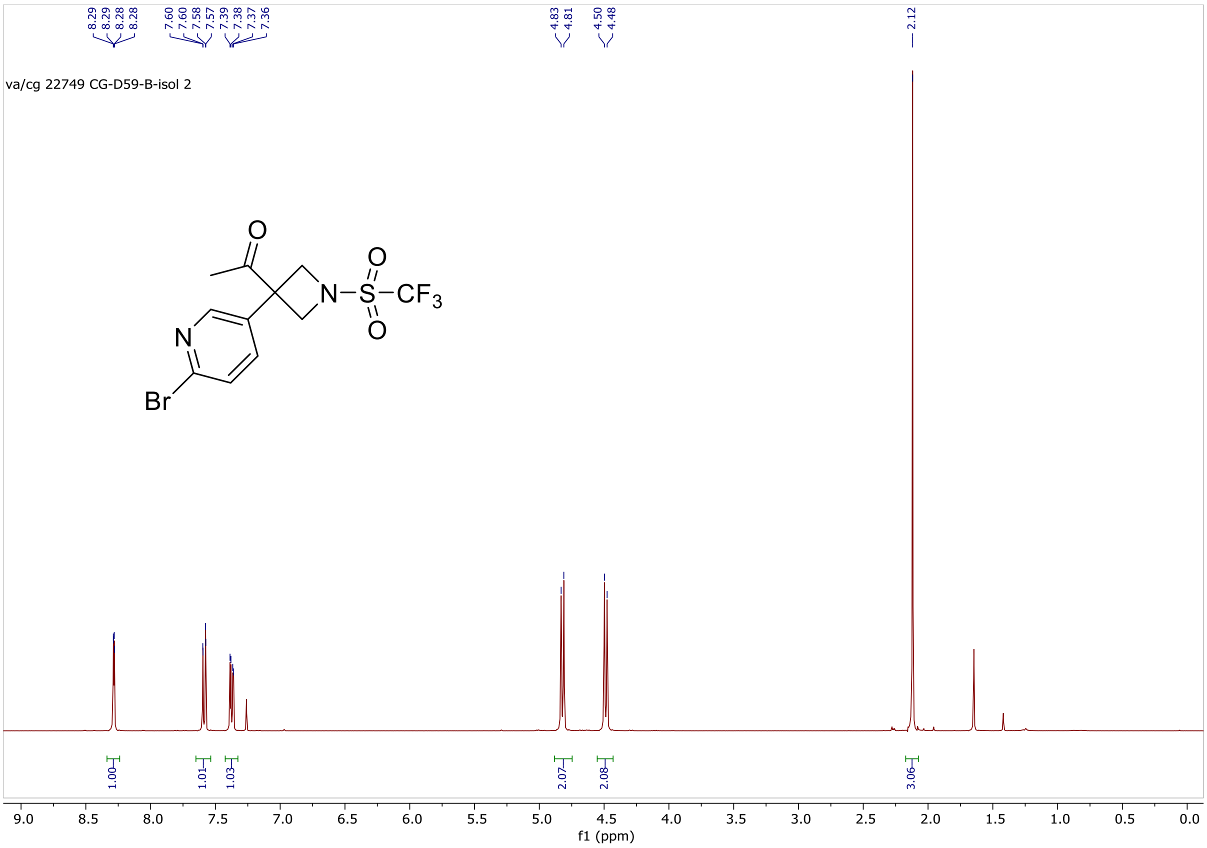

^{13}C NMR (CDCl$_3$, 101 MHz) of **7f**

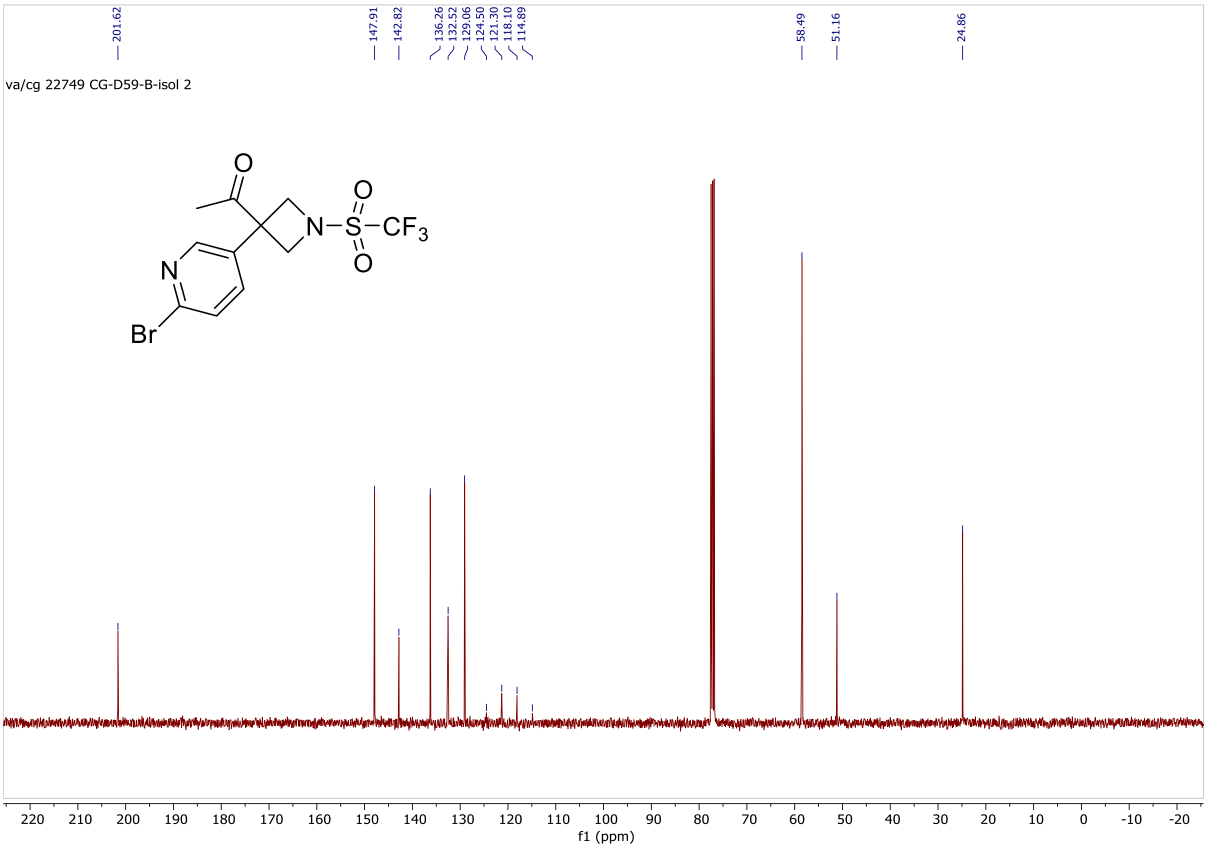

^{1}H NMR (CDCl$_3$, 400 MHz) of **7g**, See procedure

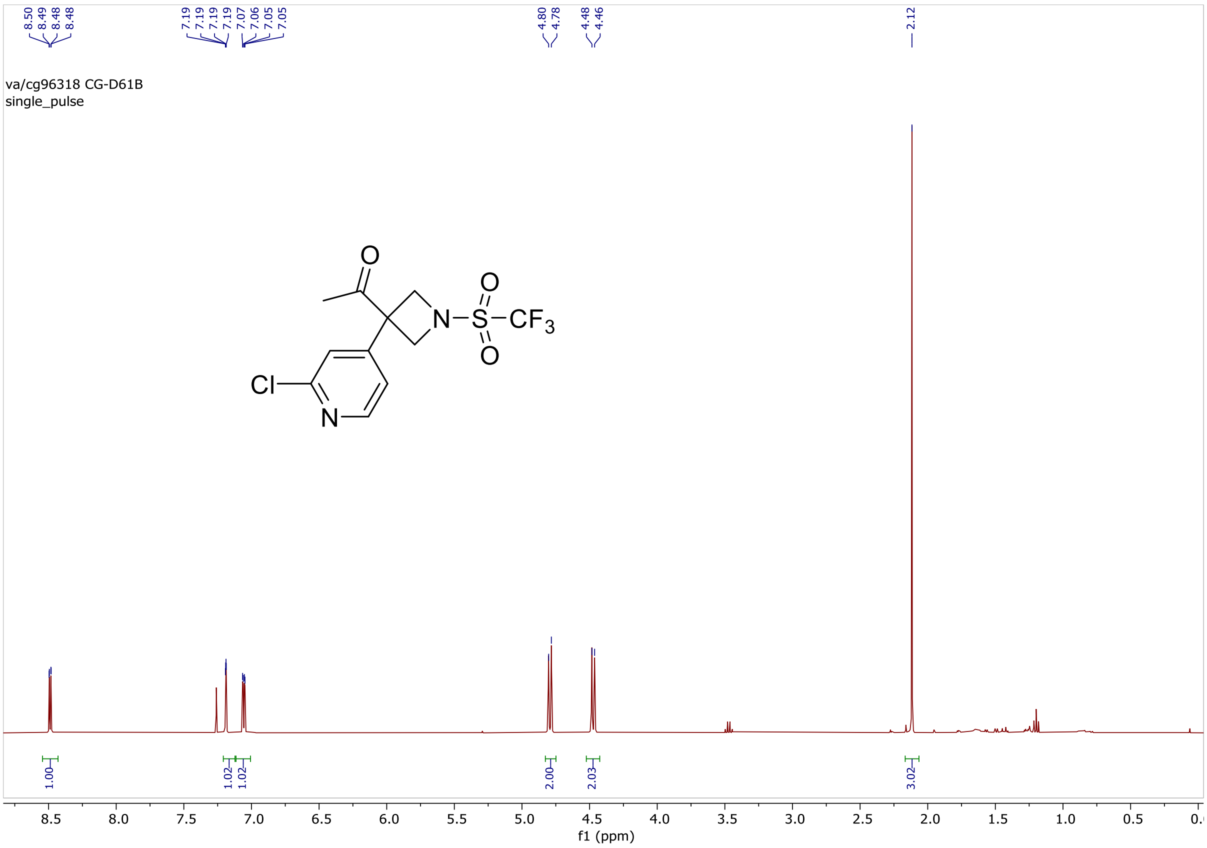

[13] **C NMR** ($CDCl_3$, 101 MHz) of **7g**

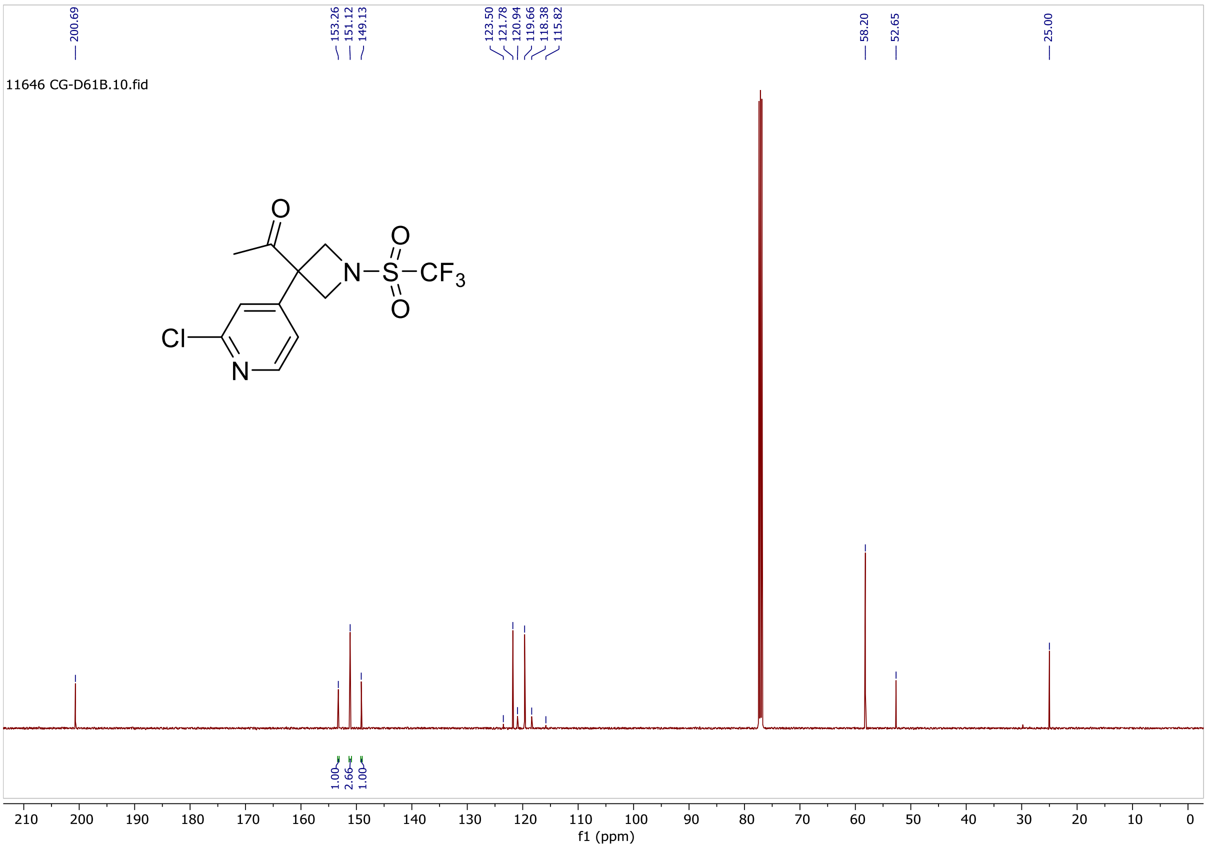

[1] **H NMR** ($CDCl_3$, 400 MHz) of **6h**, See procedure

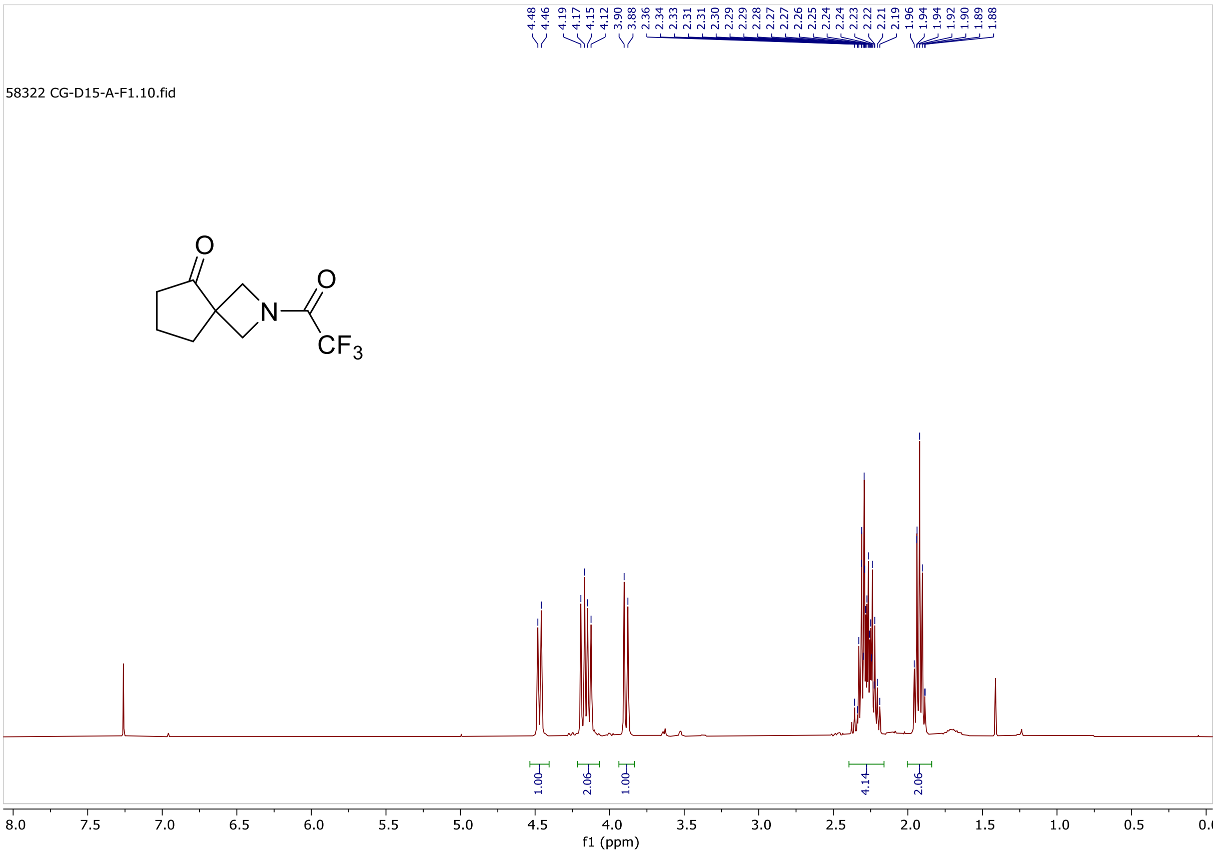

^{13}C NMR (CDCl$_3$, 101 MHz) of **6h**

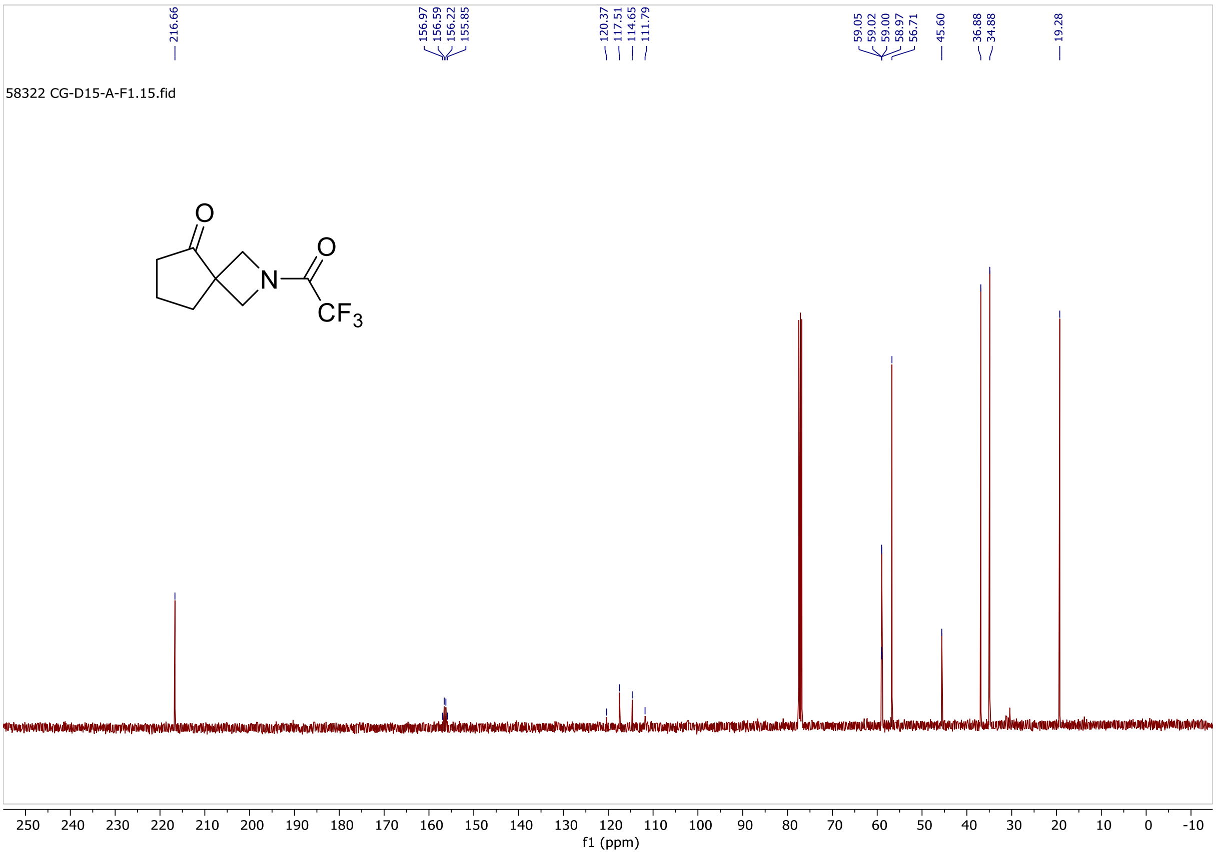

^{1}H NMR (CDCl$_3$, 400 MHz) of **7h**, See procedure

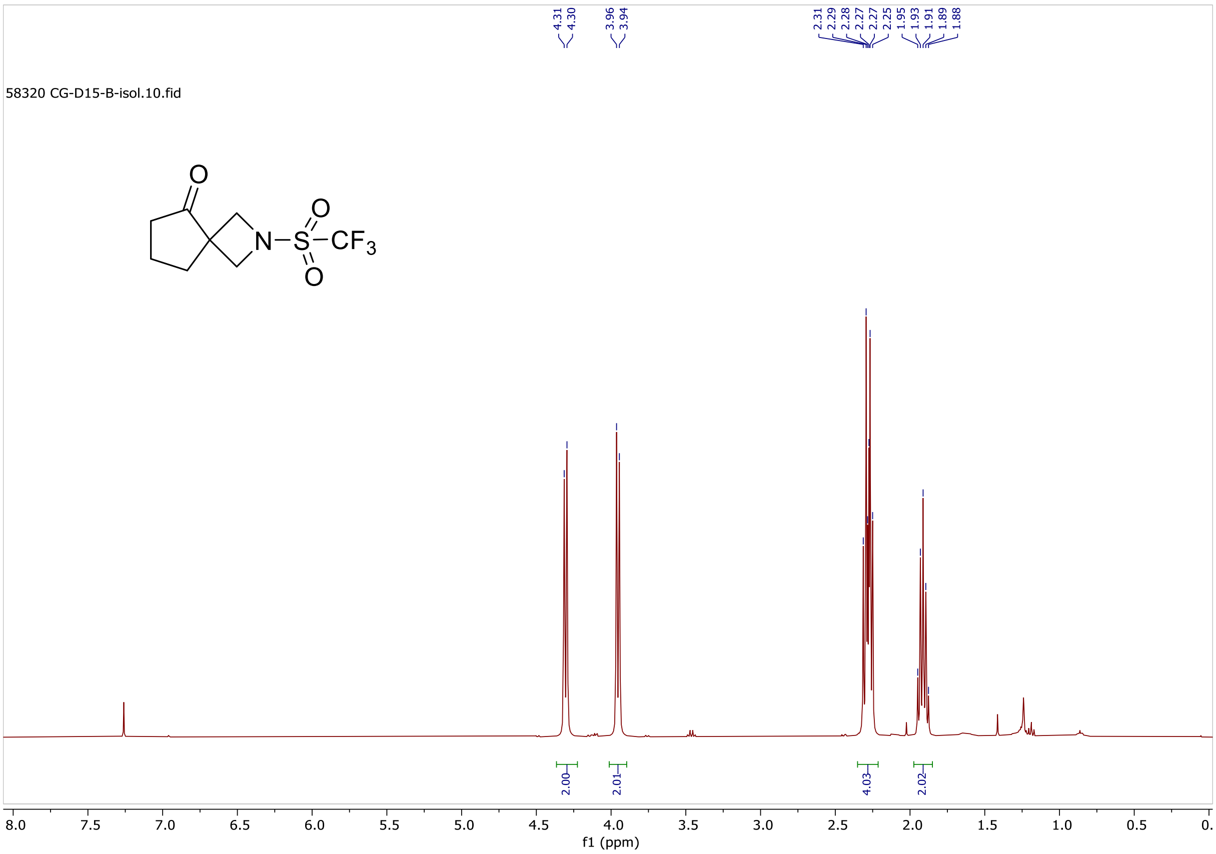

^{13}C NMR (CDCl$_3$, 101 MHz) of **7h**

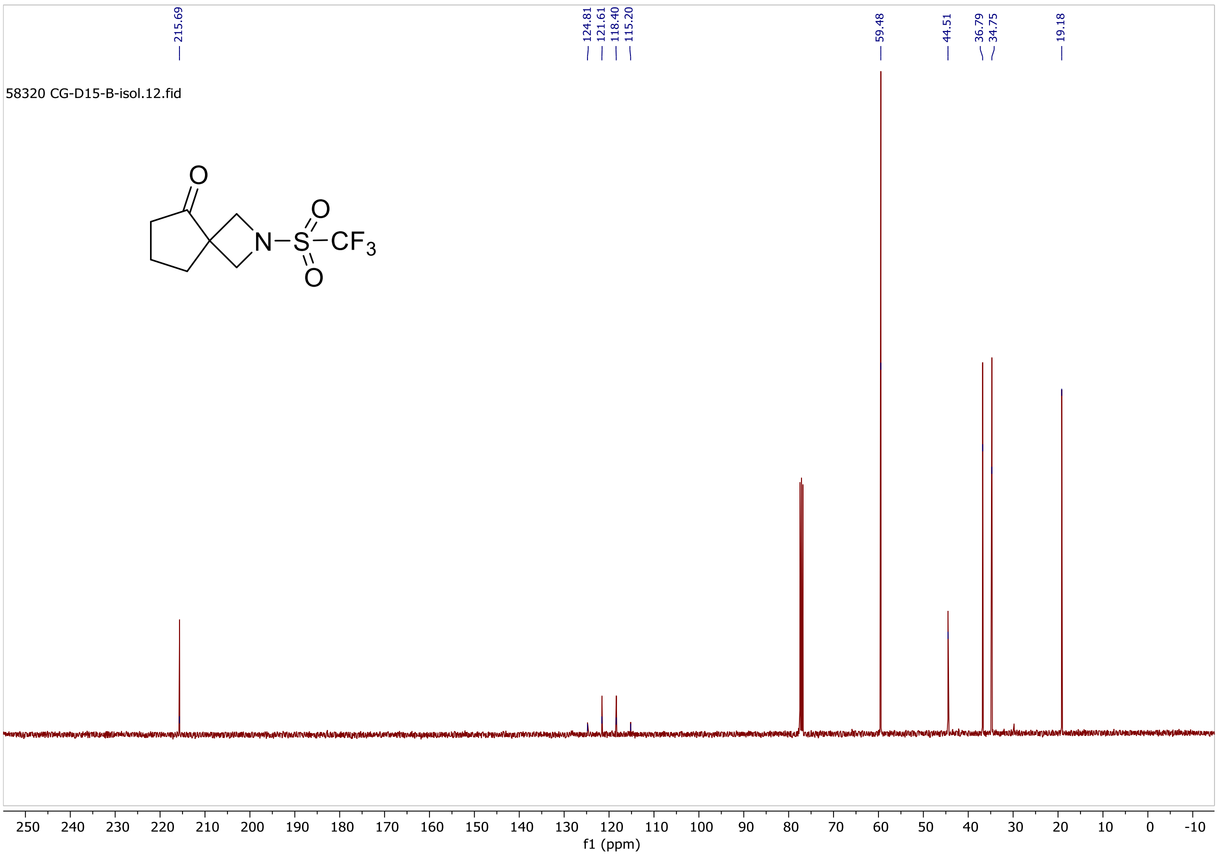

^{1}H NMR (CDCl$_3$, 400 MHz) of **6i**, See procedure

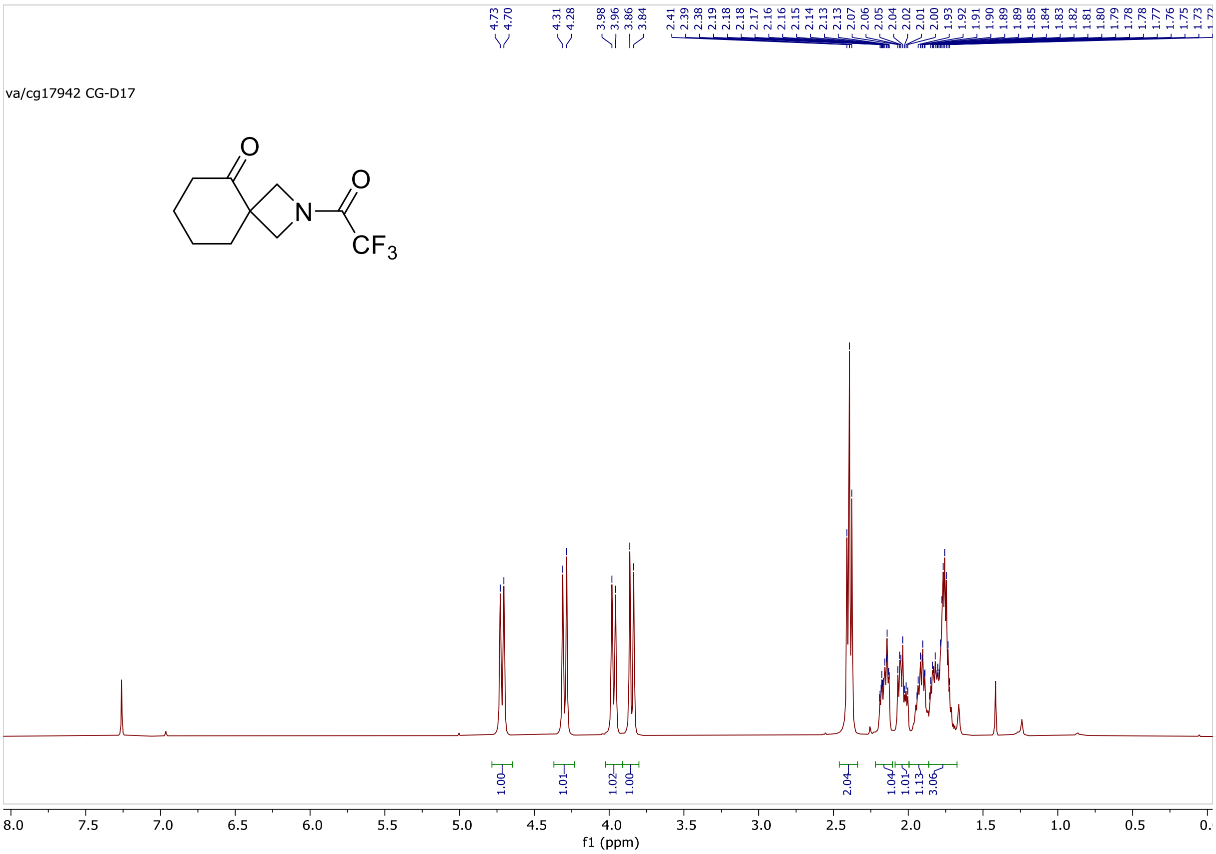

^{13}C NMR ($CDCl_3$, 101 MHz) of **6i**

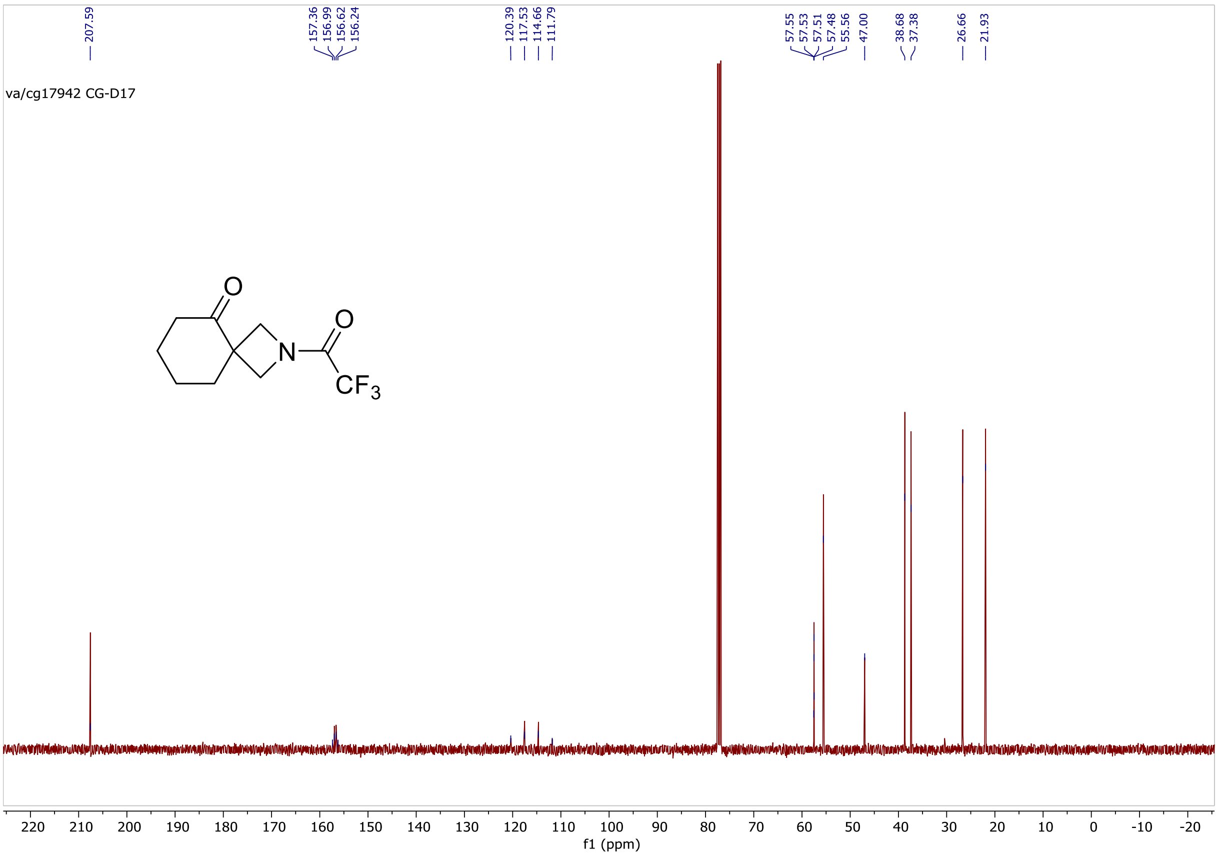

^{1}H NMR ($CDCl_3$, 400 MHz) of **7i**, See procedure

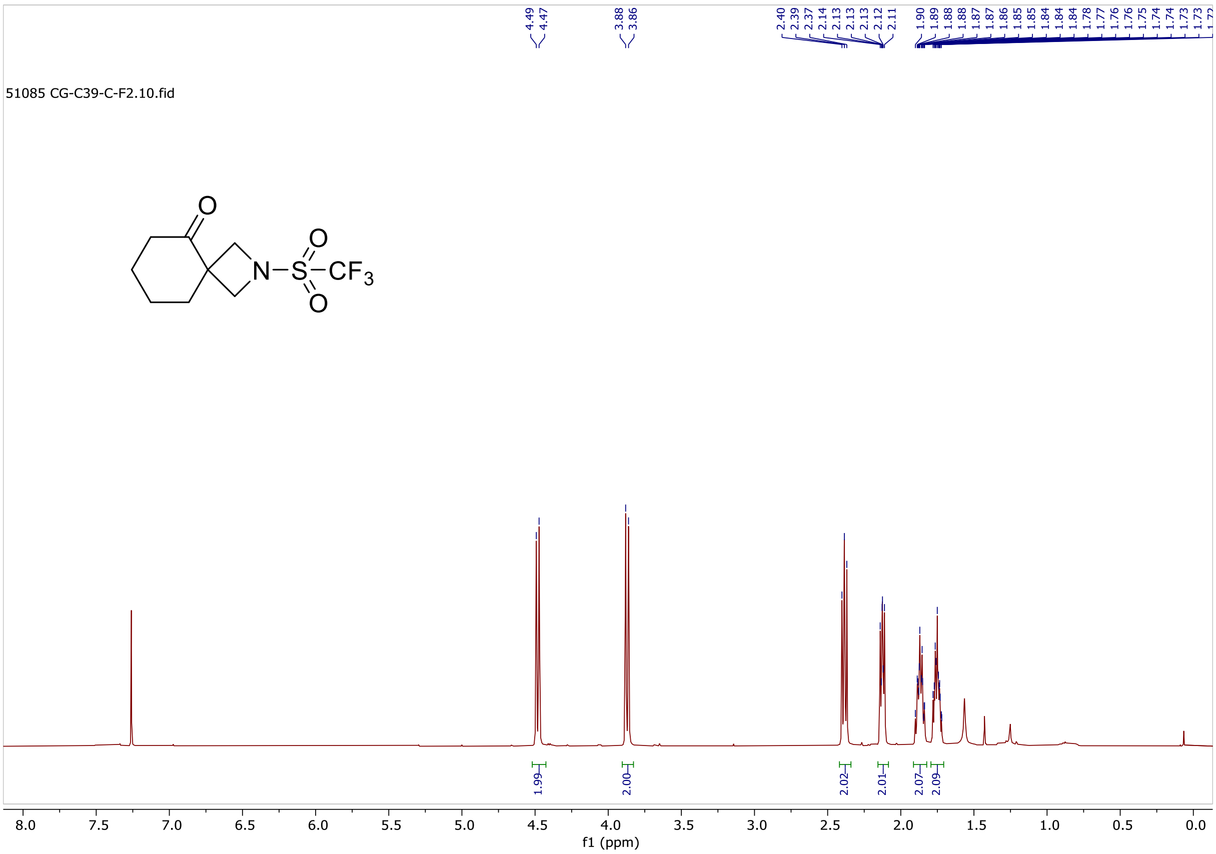

^{13}C NMR ($CDCl_3$, 101 MHz) of **7i**

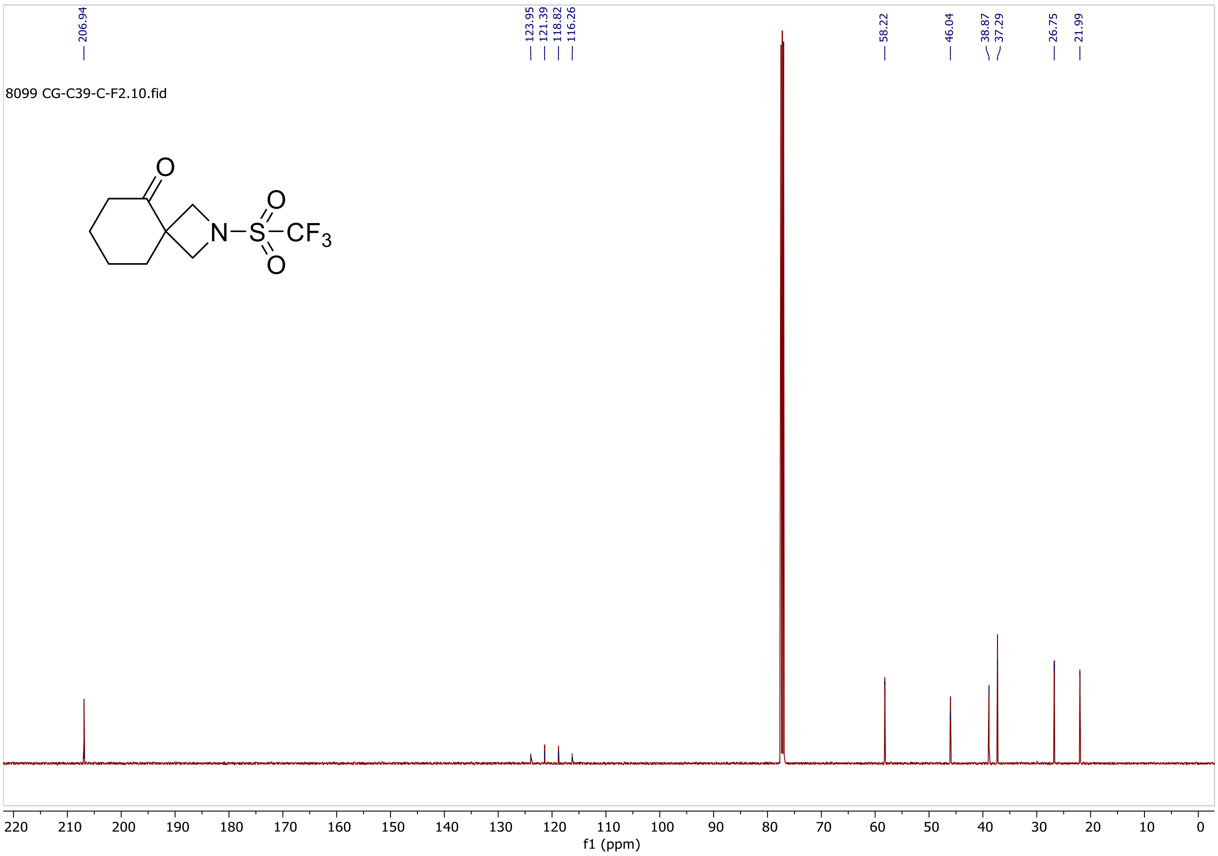

^{1}H NMR ($CDCl_3$, 400 MHz) of **6j**, See procedure

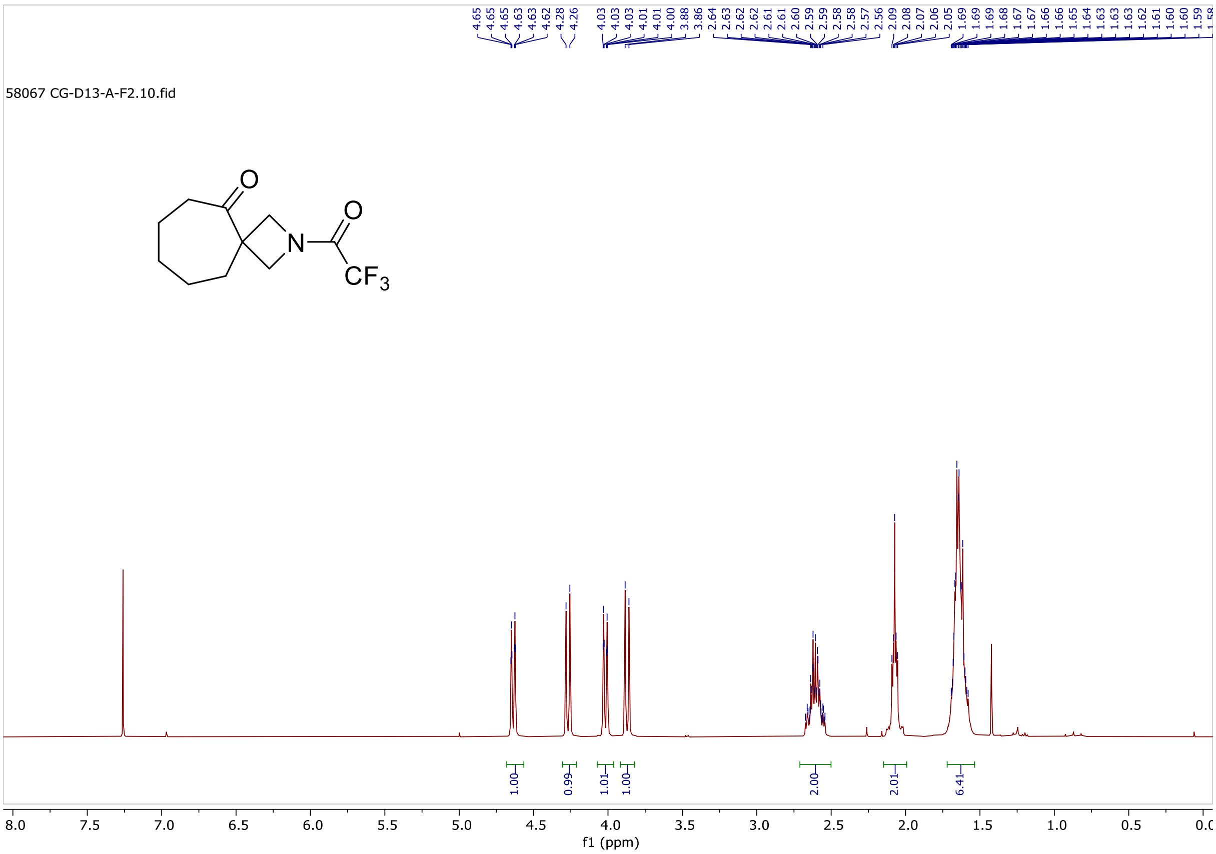

^{13}C NMR ($CDCl_3$, 101 MHz) of **6j**

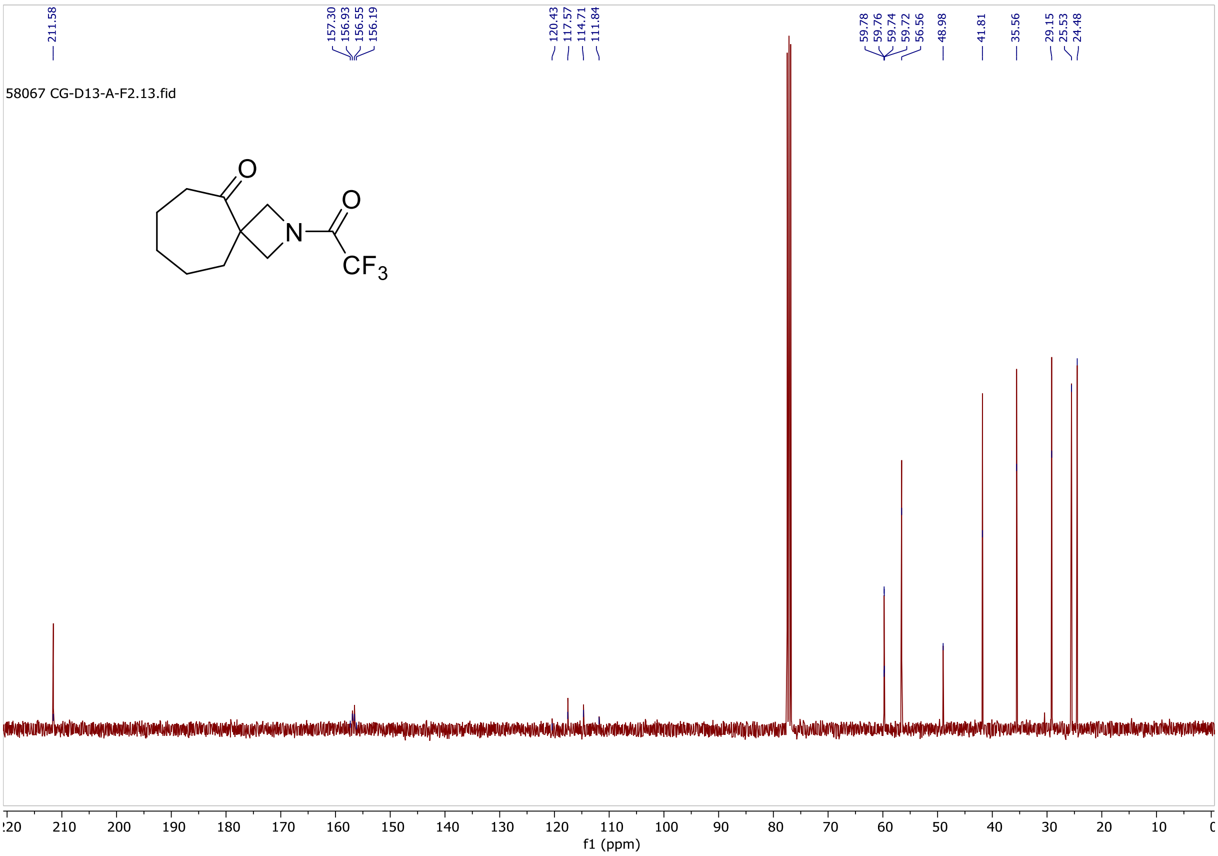

^{1}H NMR ($CDCl_3$, 400 MHz) of **7j**, See procedure

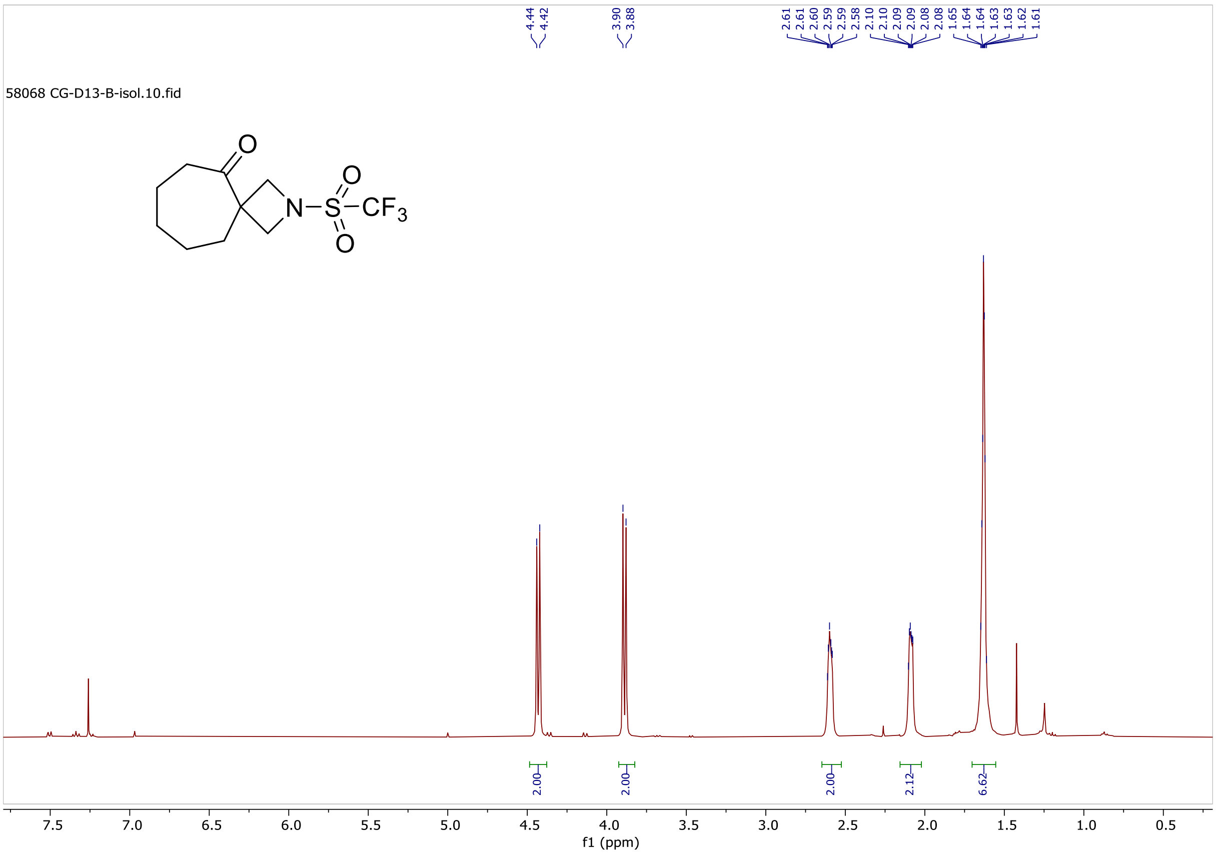

^{13}**C NMR** ($CDCl_3$, 101 MHz) of **7j**

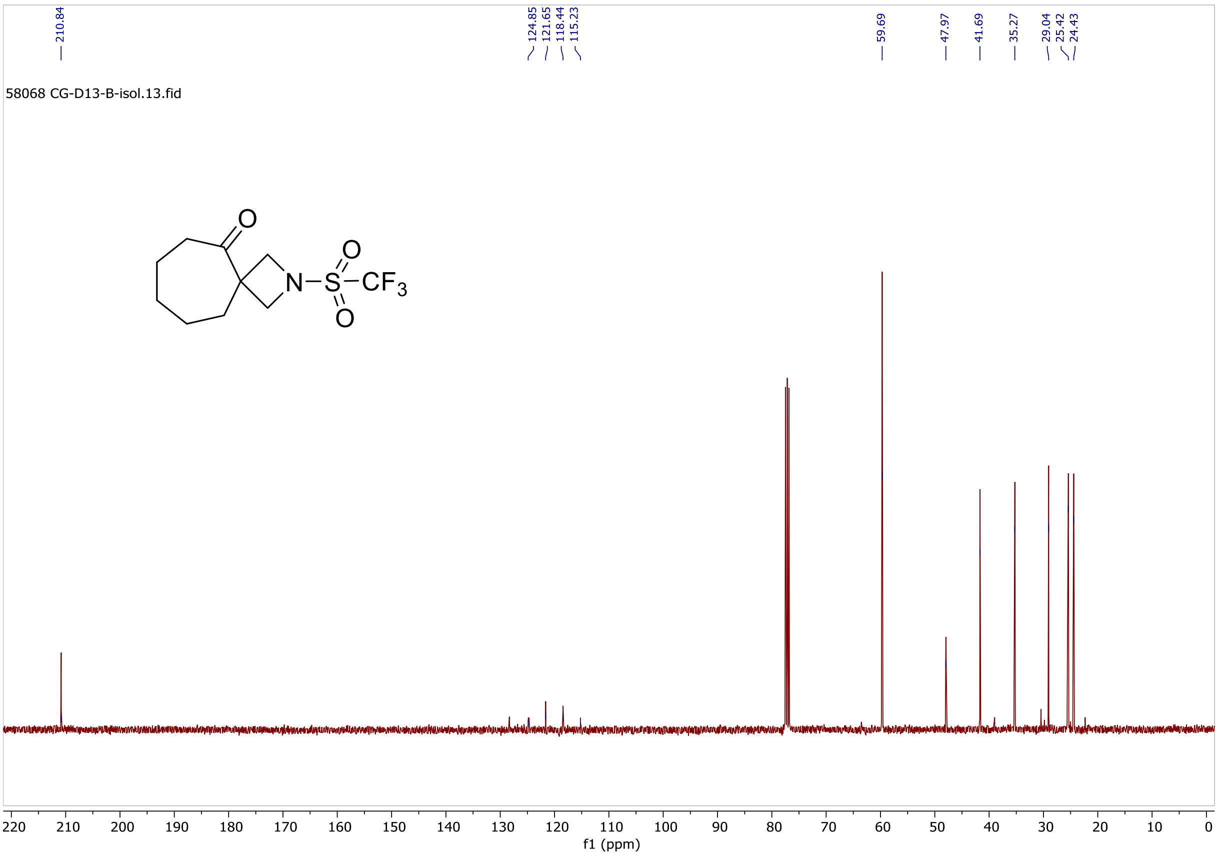

^{1}**H NMR** ($CDCl_3$, 400 MHz) of **6k**, See procedure

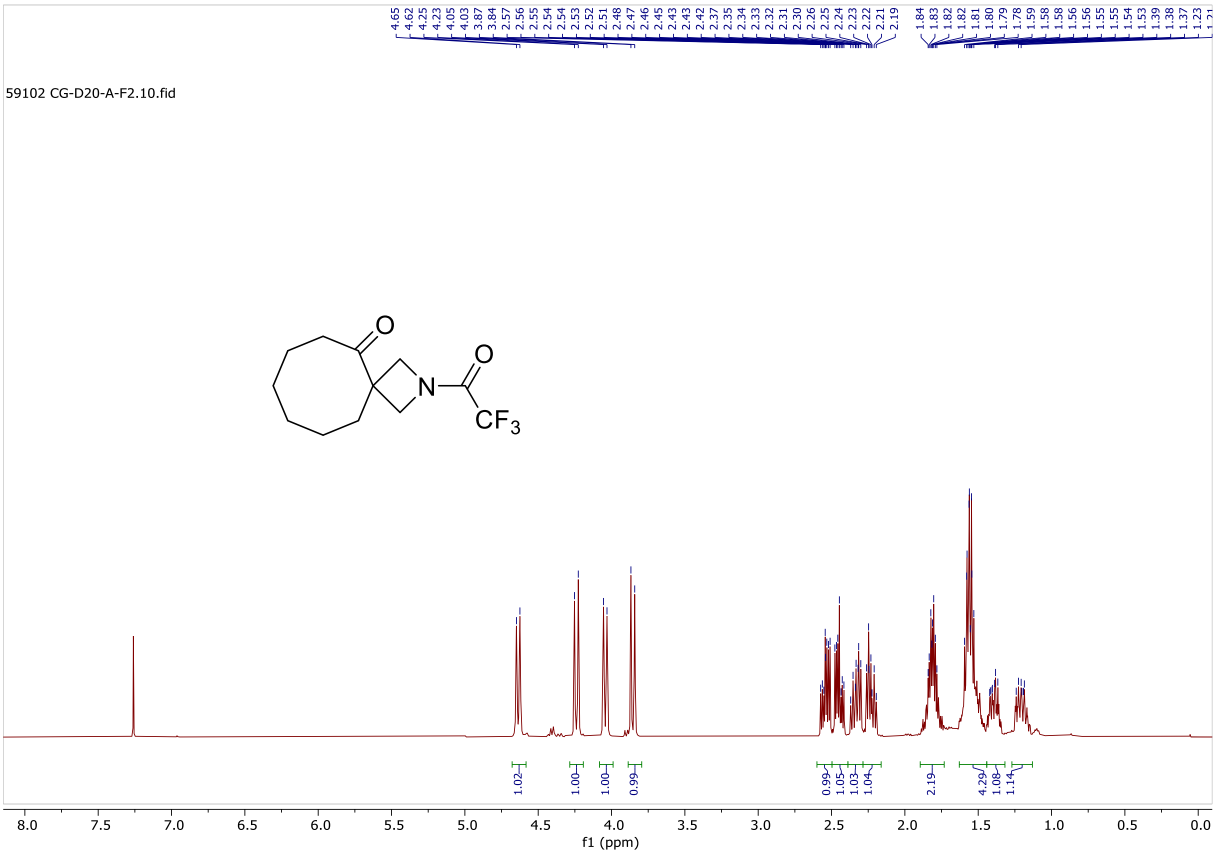

^{13}C NMR ($CDCl_3$, 101 MHz) of **6k**

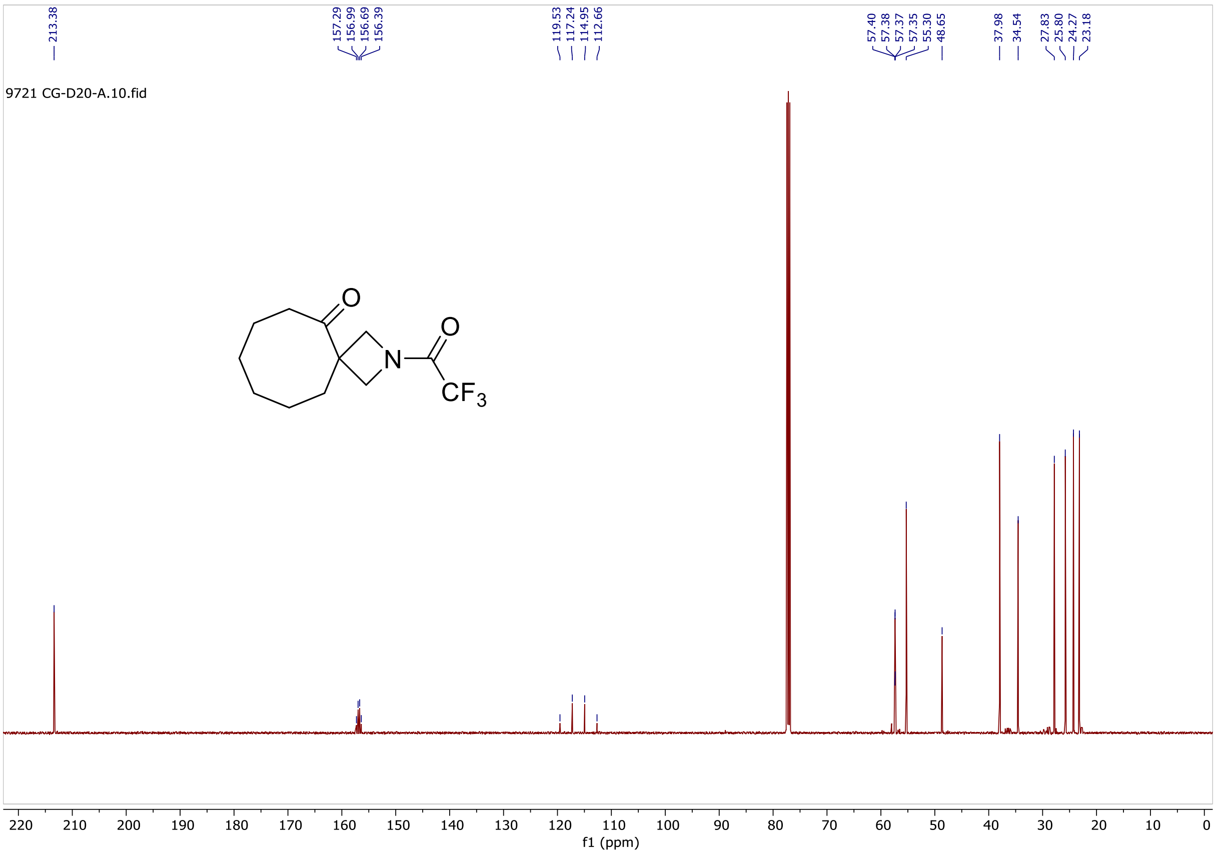

^{1}H NMR ($CDCl_3$, 400 MHz) of **7k**, See procedure

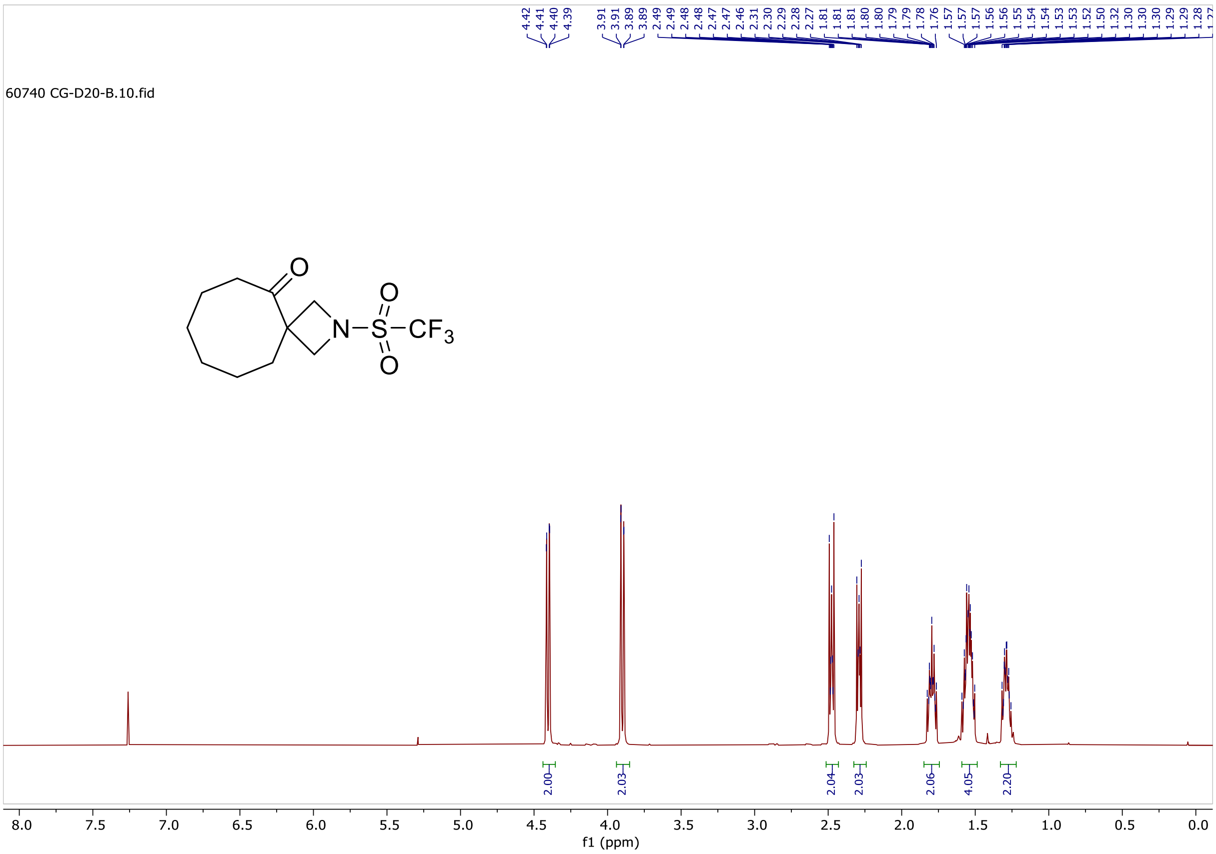

^{13}**C NMR** (CDCl$_3$, 101 MHz) of **7k**

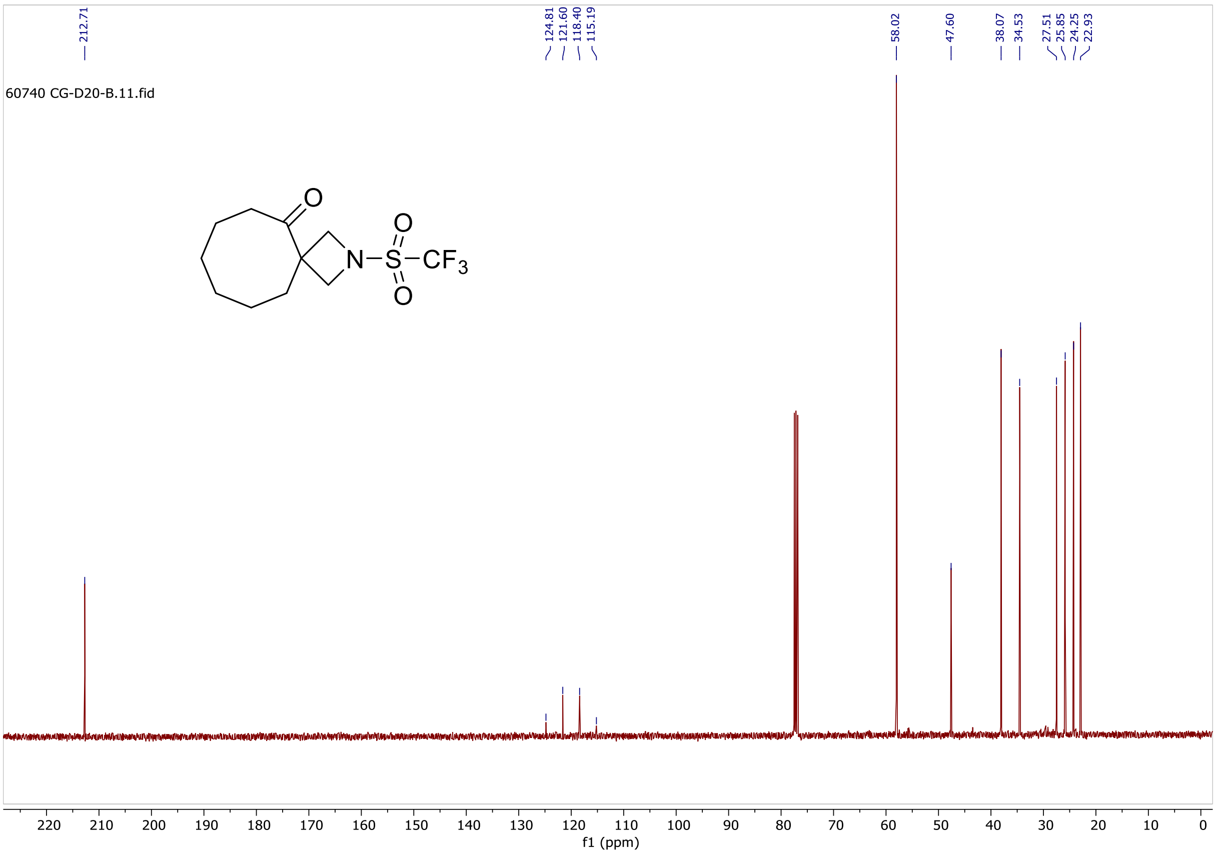

^{1}**H NMR** (CDCl$_3$, 500 MHz) of **6l**, See procedure

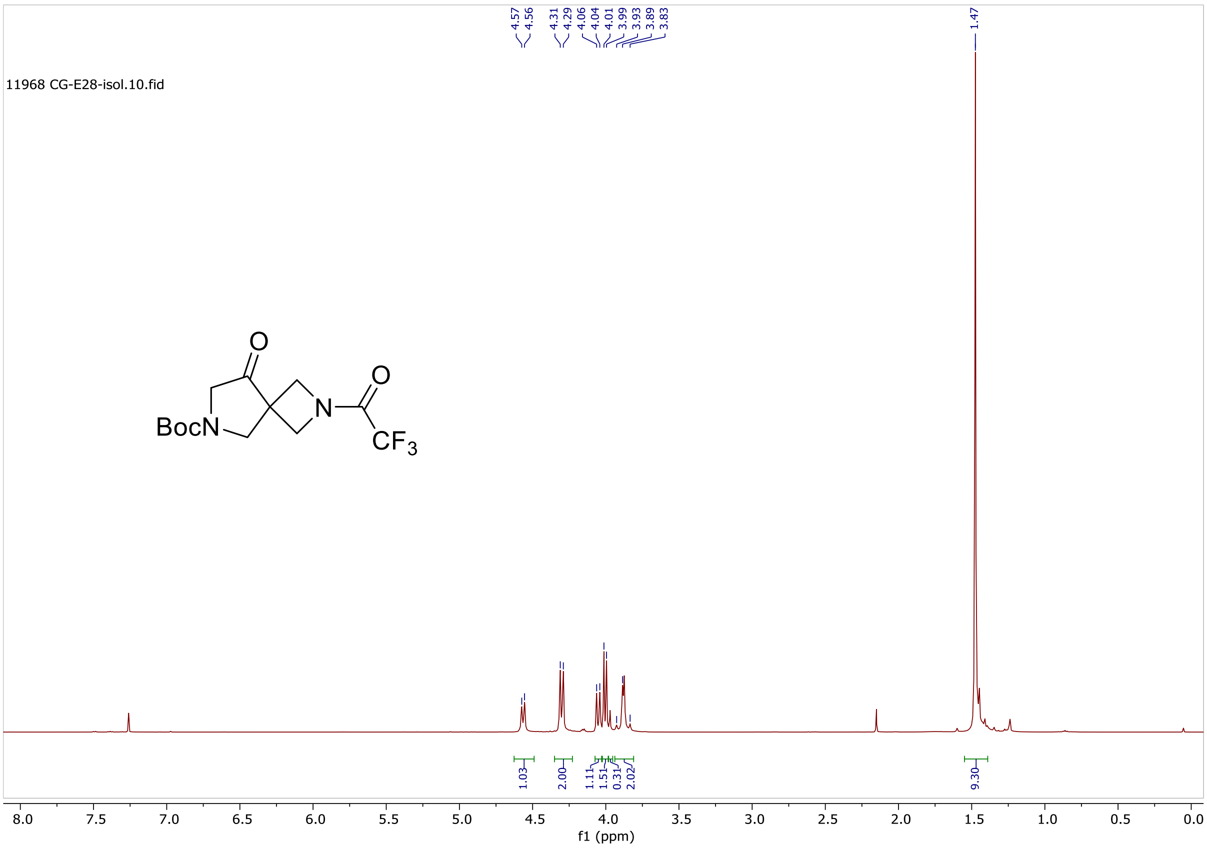

^{13}C NMR ($CDCl_3$, 101 MHz) of **6l**

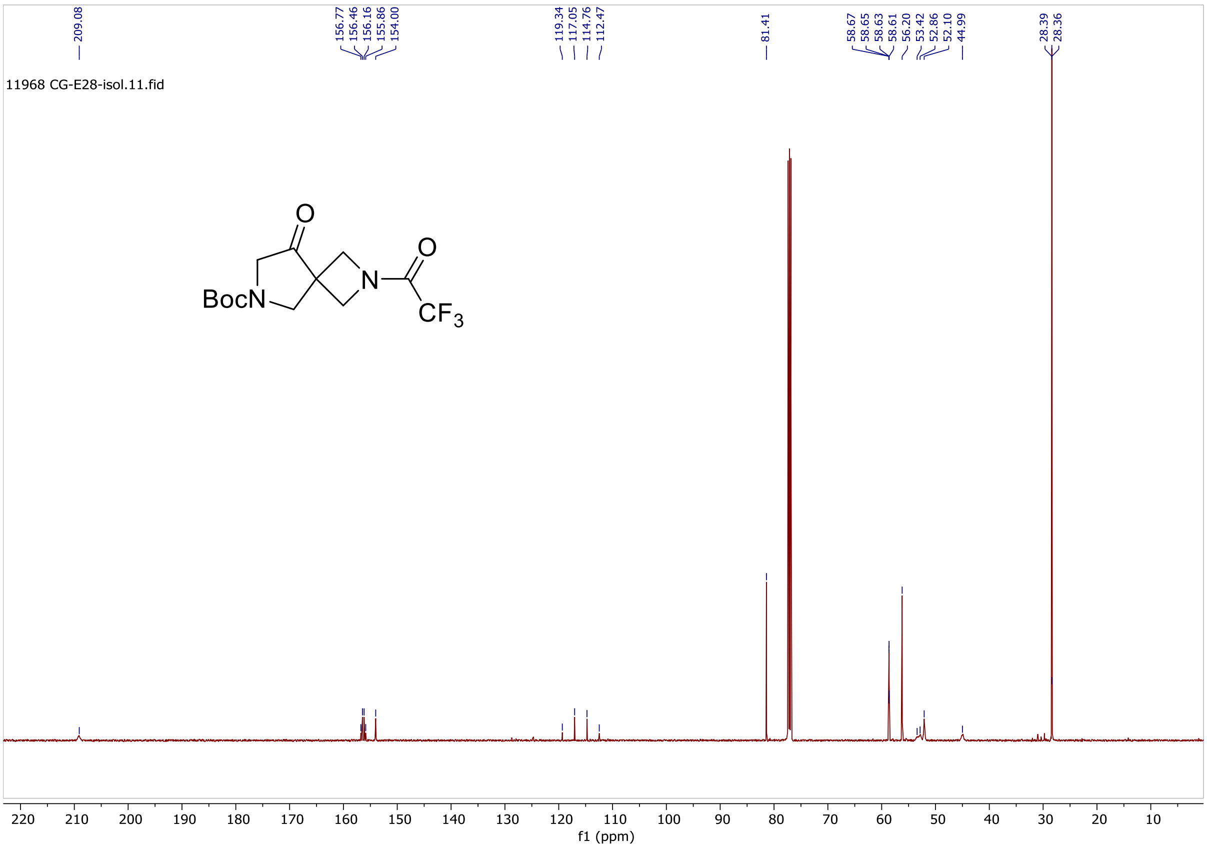

^{1}H NMR ($CDCl_3$, 400 MHz) of **7l**, See procedure

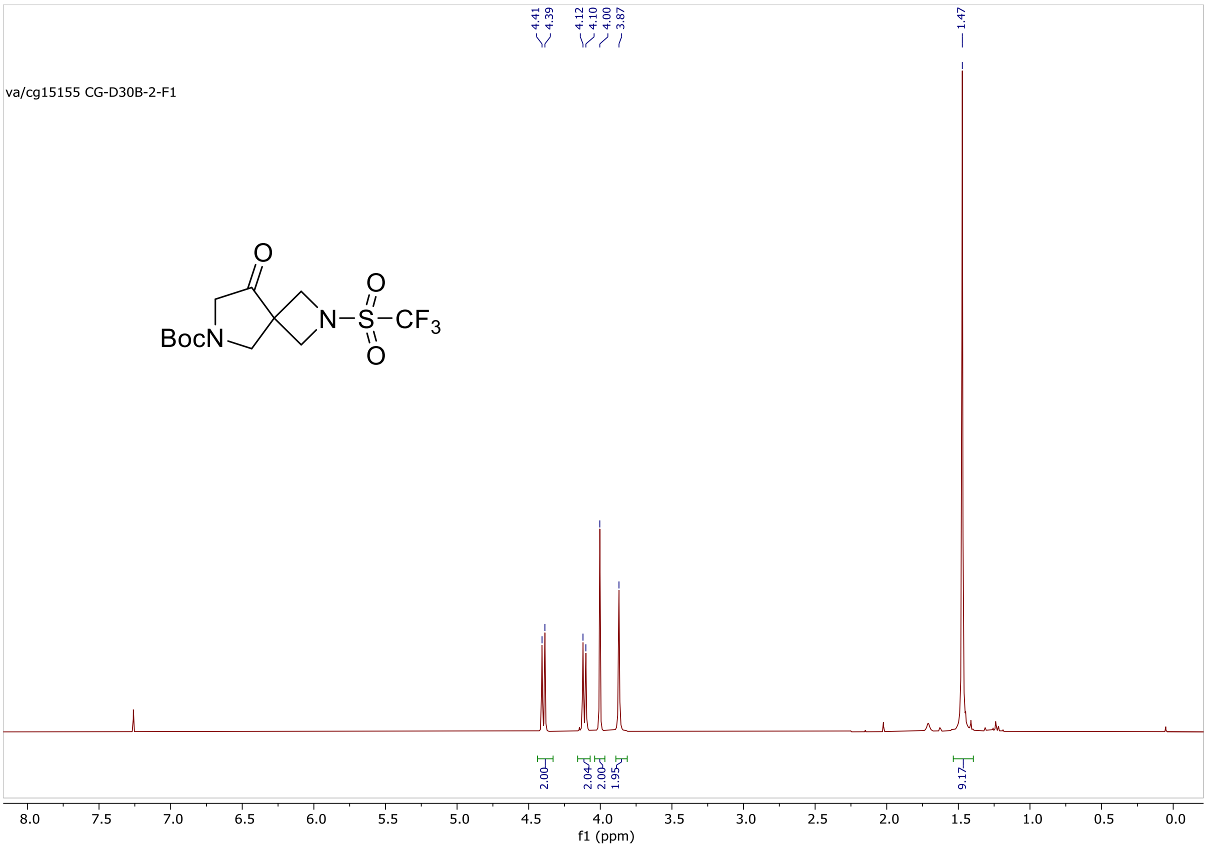

^{13}**C NMR** ($CDCl_3$, 101 MHz) of **7l**

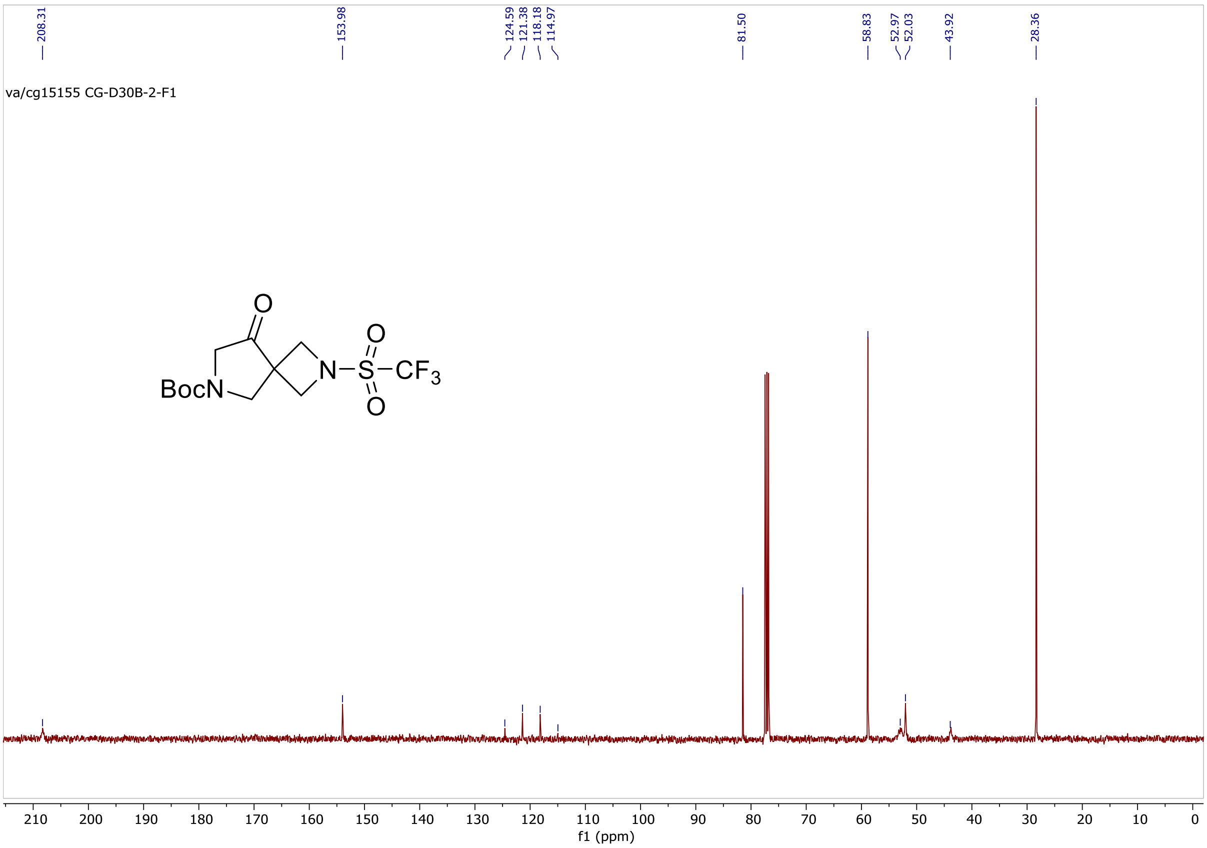

^{1}**H NMR** ($CDCl_3$, 400 MHz) of **6m**, See procedure

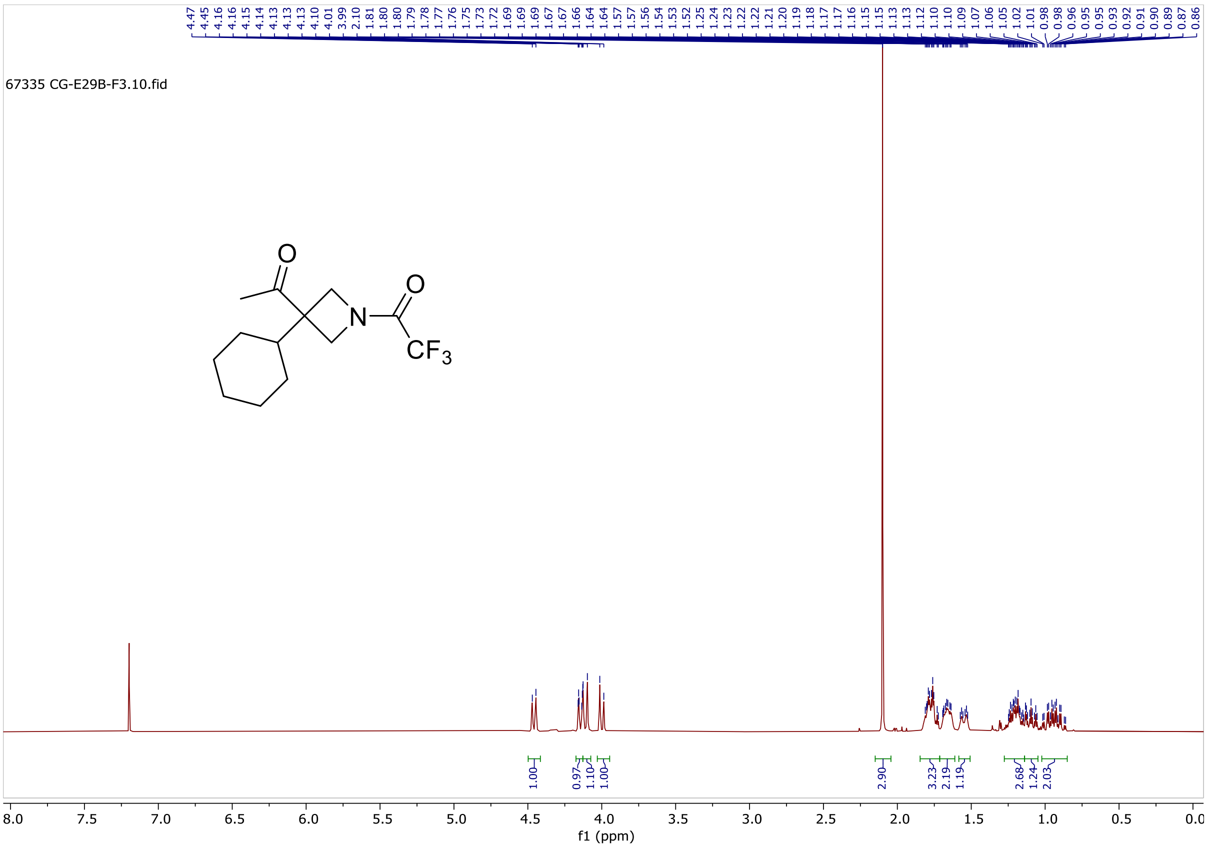

^{13}C NMR ($CDCl_3$, 101 MHz) of **6m**

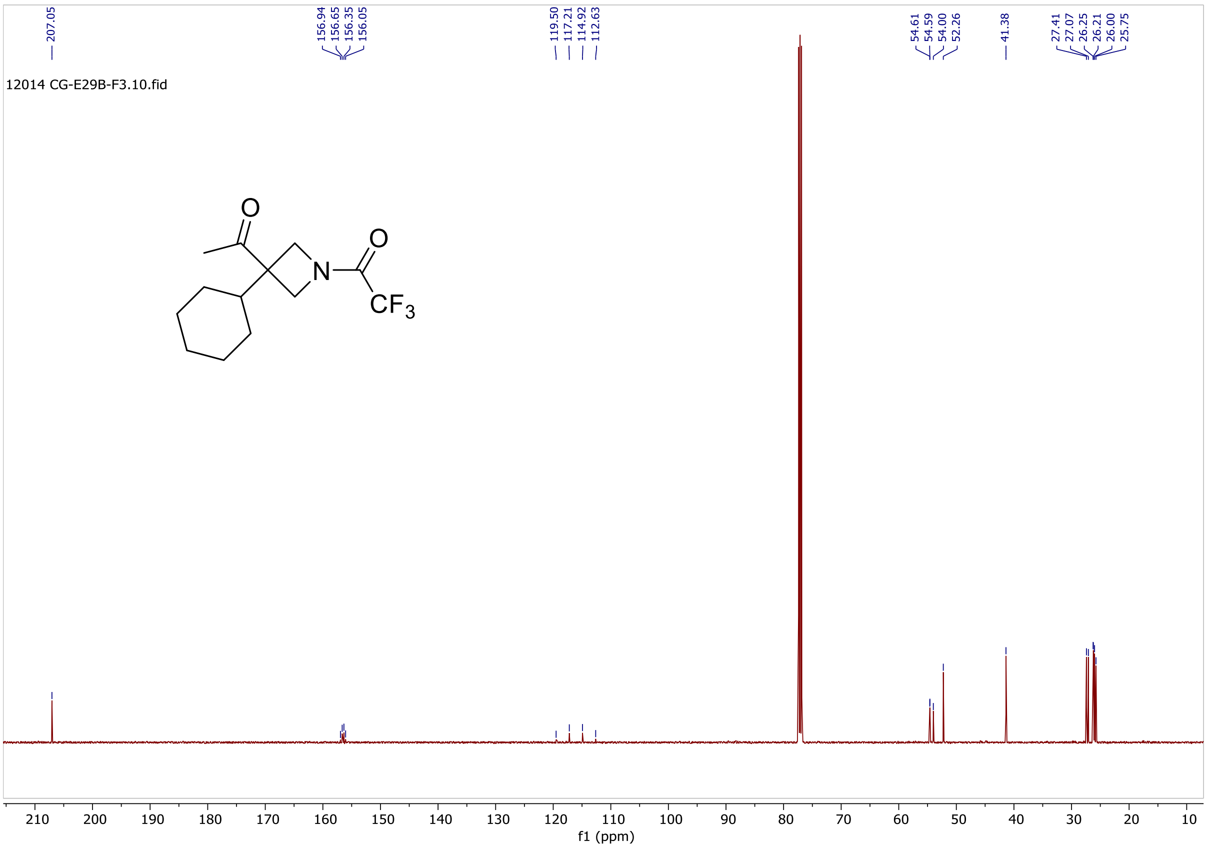

^{1}H NMR ($CDCl_3$, 400 MHz) of **7m**, See procedure

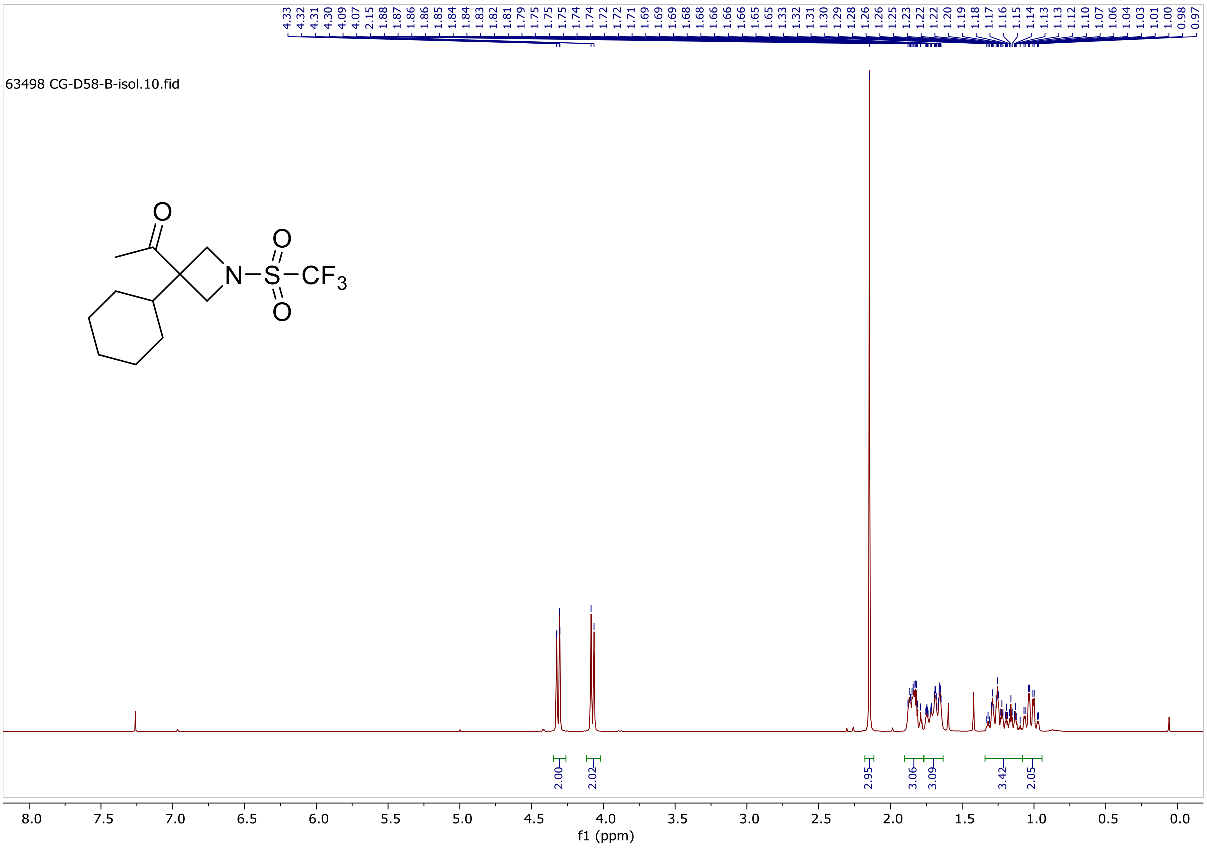

^{13}**C NMR** ($CDCl_3$, 101 MHz) of **7m**

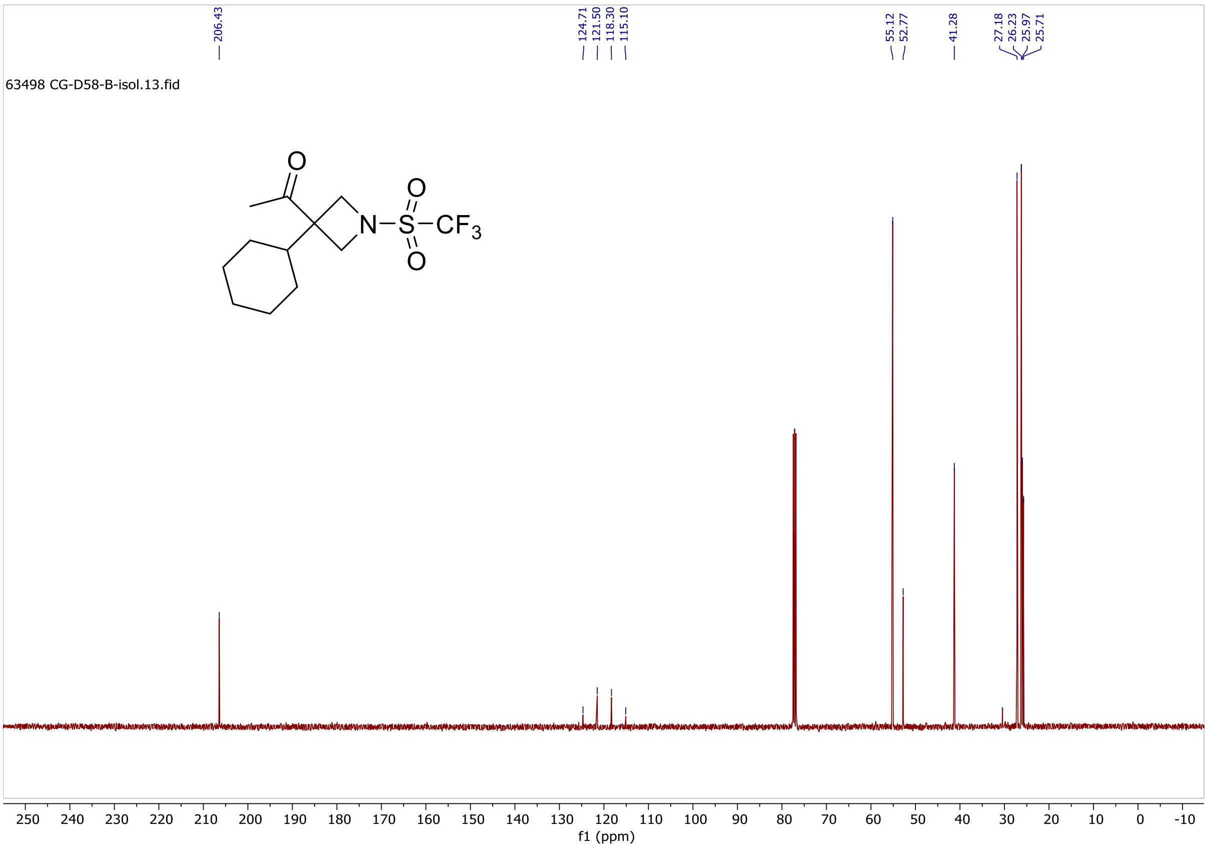

^{1}**H NMR** ($CDCl_3$, 400 MHz) of **6n**, See procedure

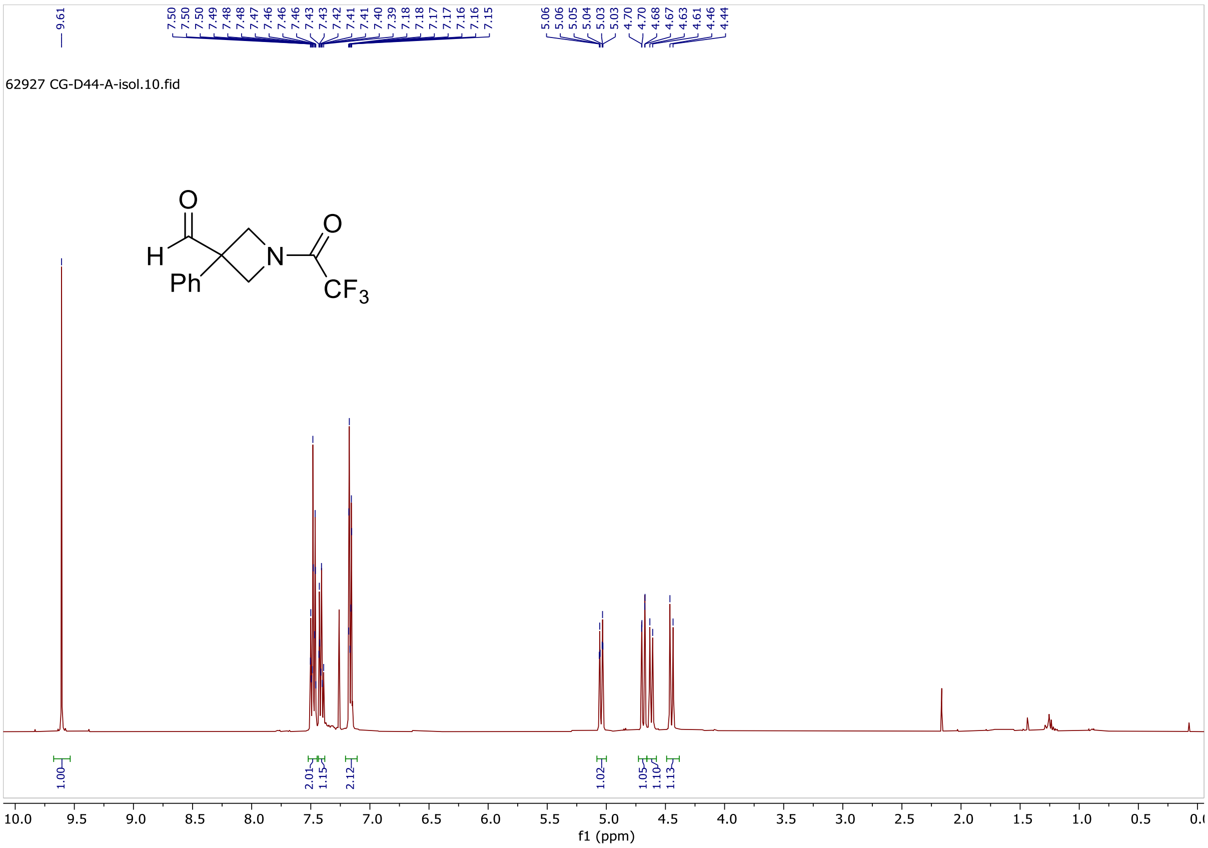

^{13}C NMR (CDCl$_3$, 101 MHz) of **6n**

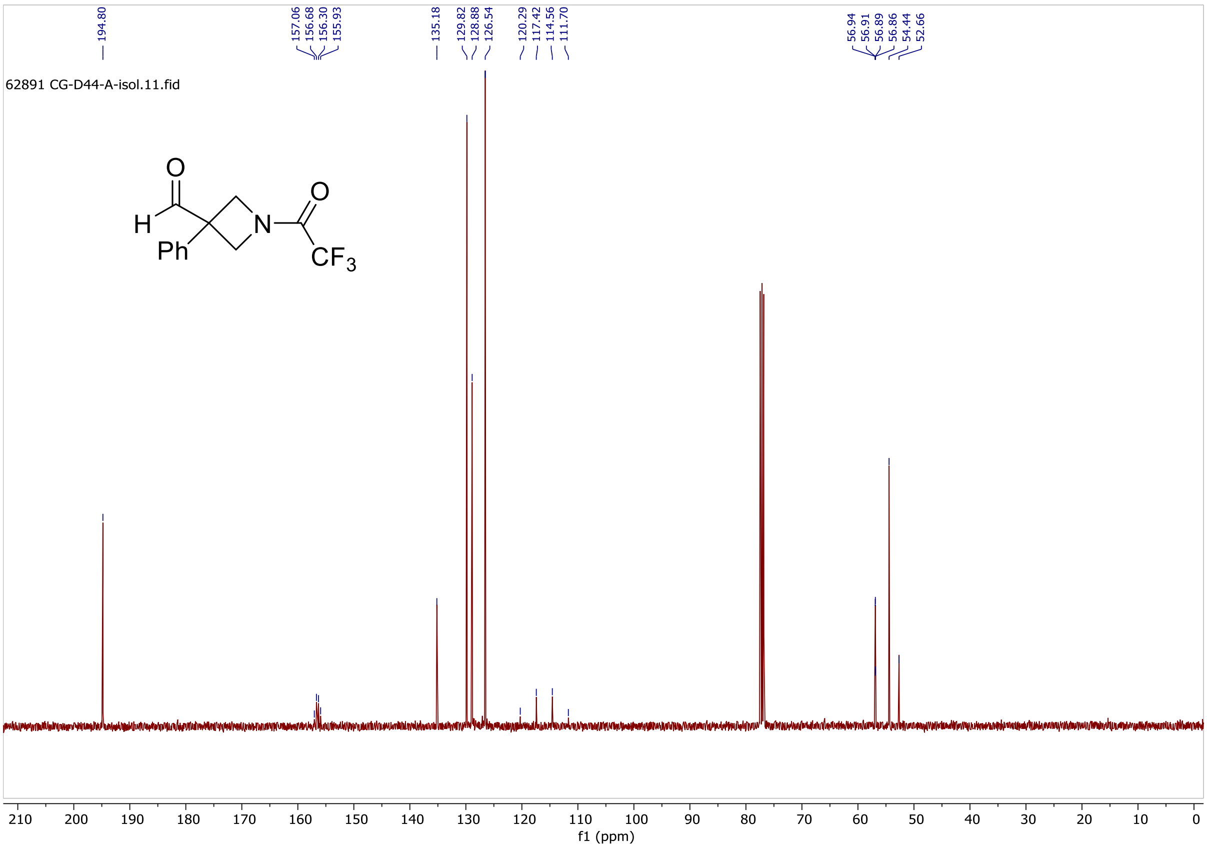

^{1}H NMR (CDCl$_3$, 400 MHz) of **7n**, See procedure

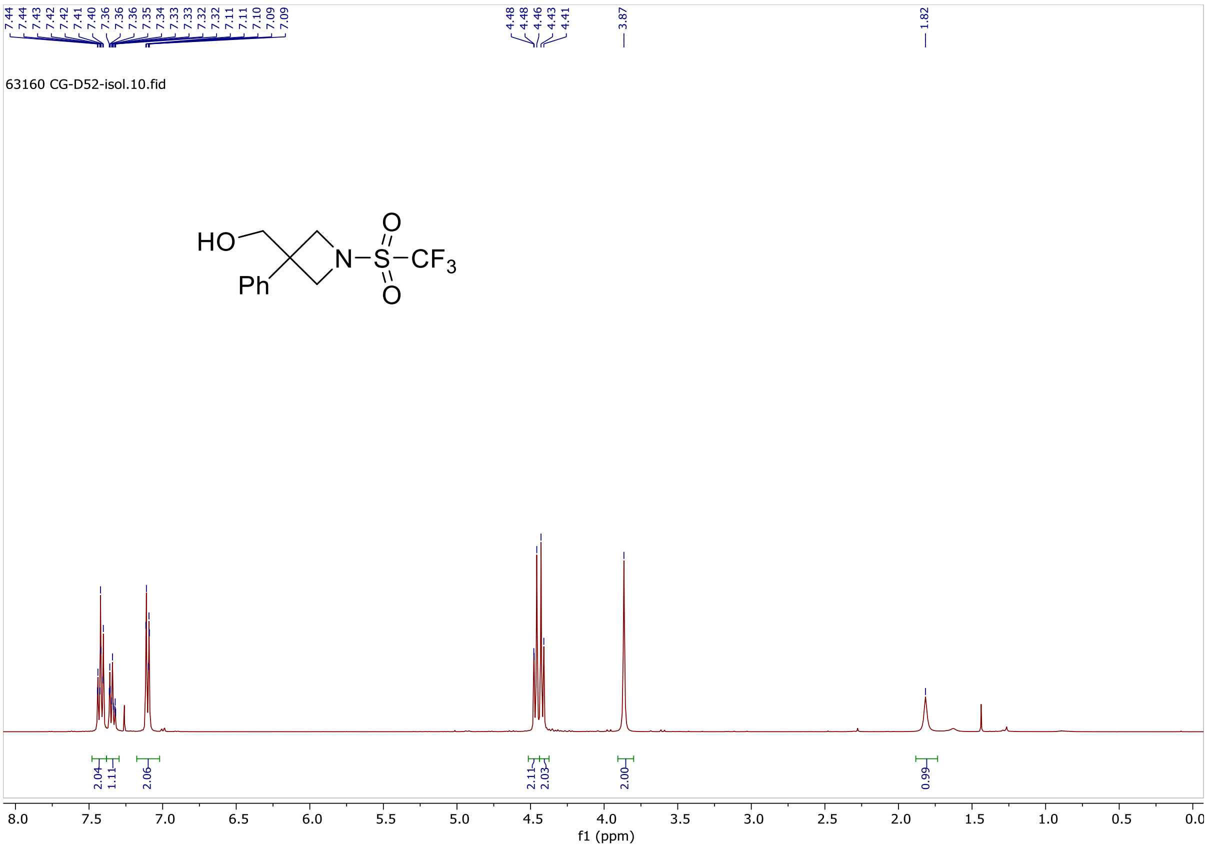

^{13}C NMR ($CDCl_3$, 101 MHz) of 7n

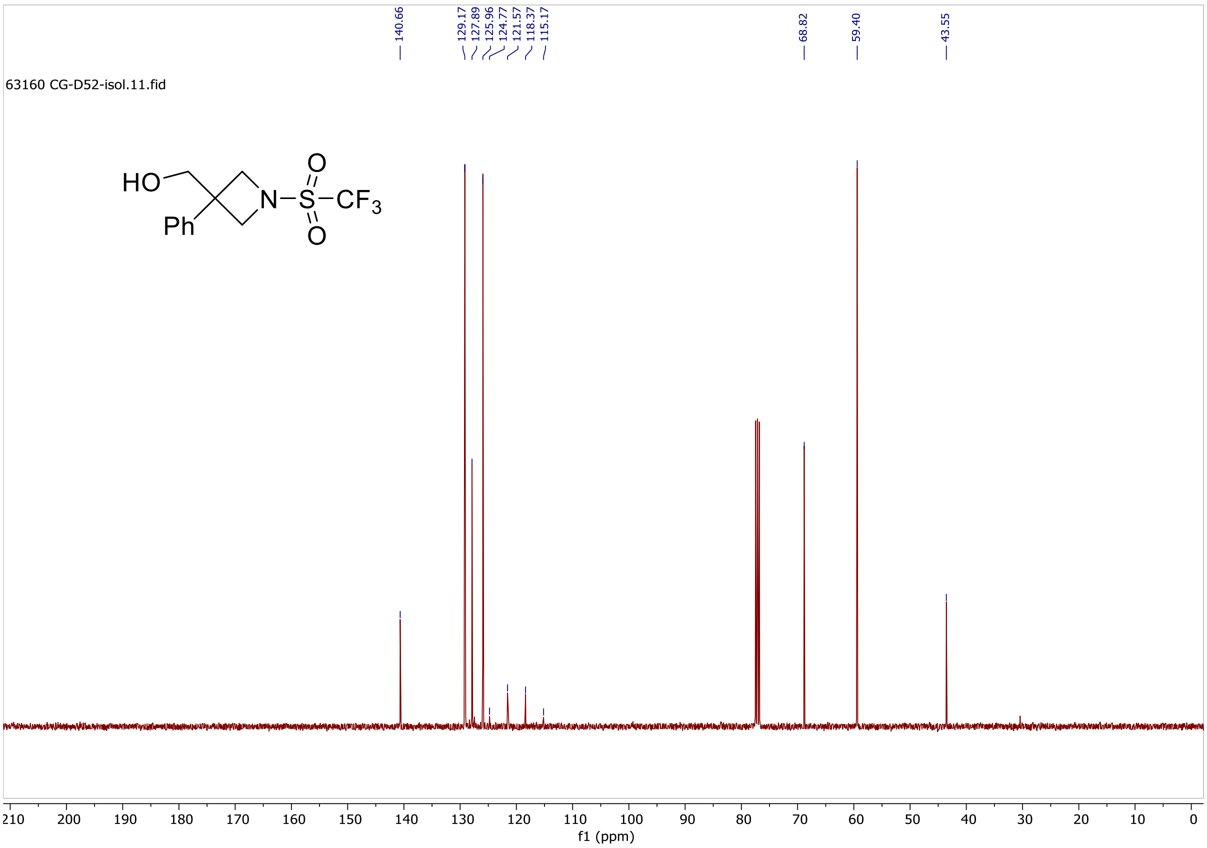

^{1}H NMR ($CDCl_3$, 400 MHz) of 6o, See procedure

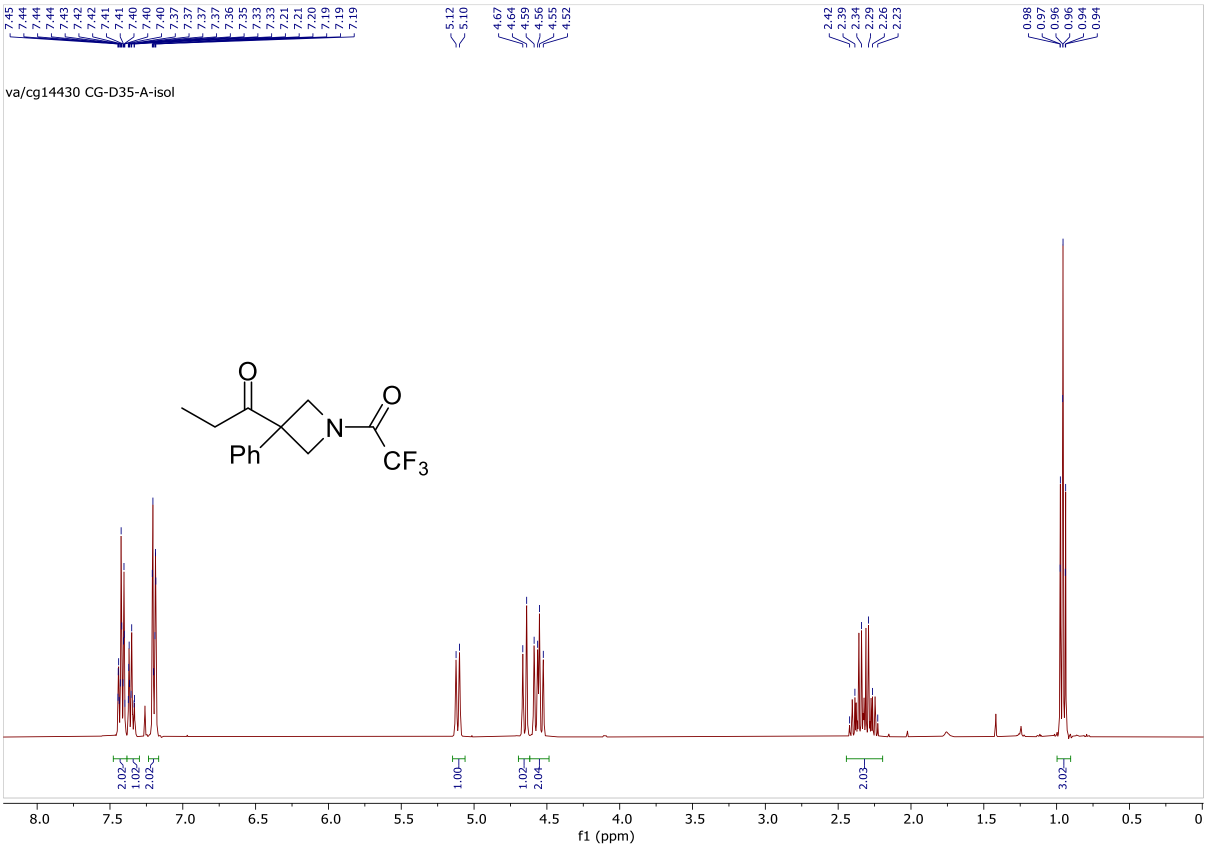

^{13}C NMR ($CDCl_3$, 101 MHz) of **6o**

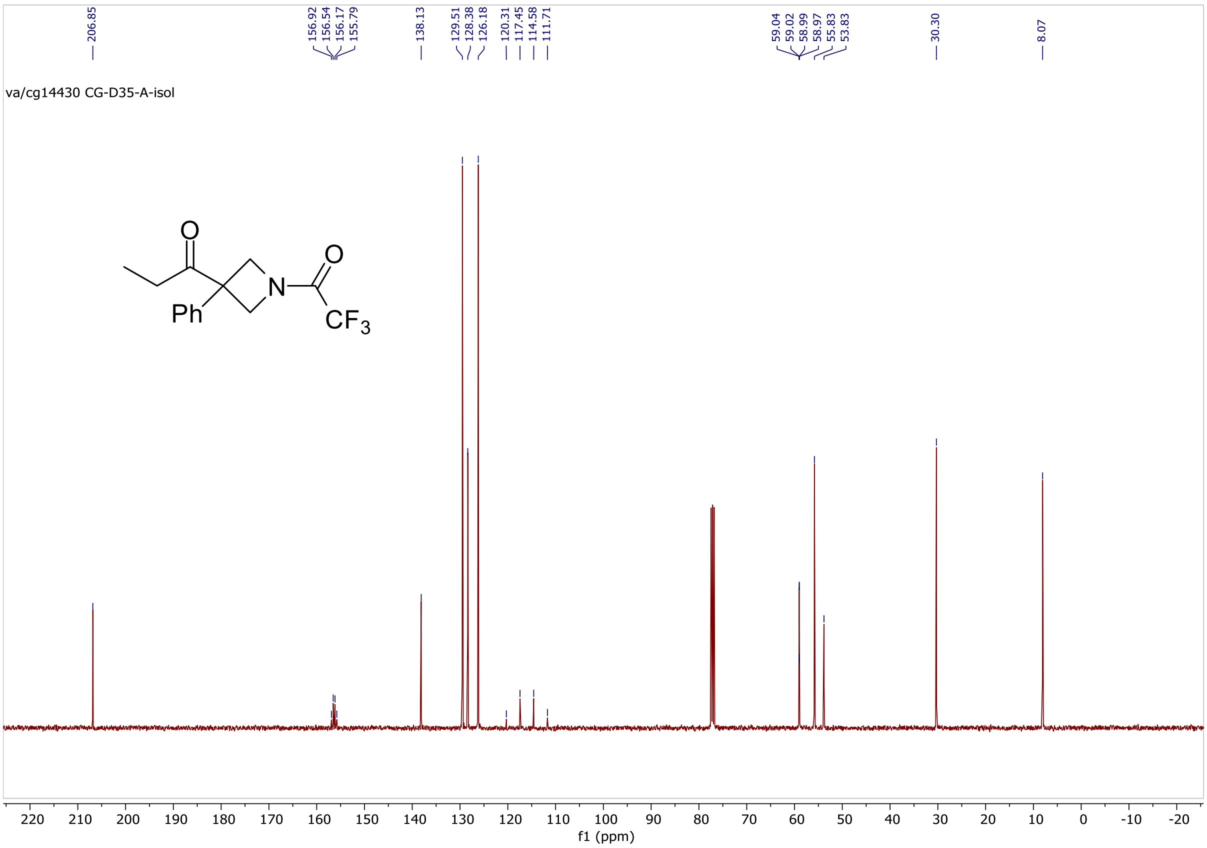

^{1}H NMR ($CDCl_3$, 400 MHz) of **7o**, See procedure

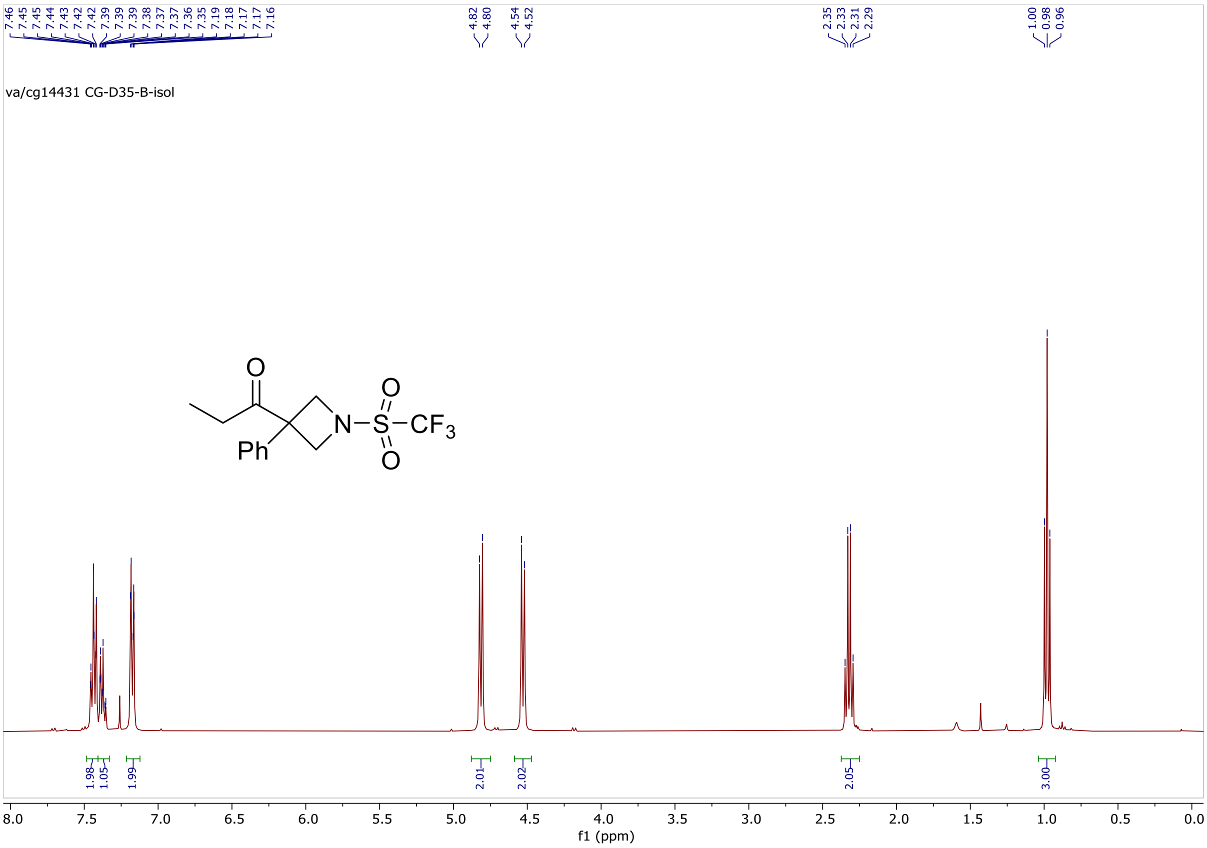

^{13}C NMR ($CDCl_3$, 101 MHz) of **7o**

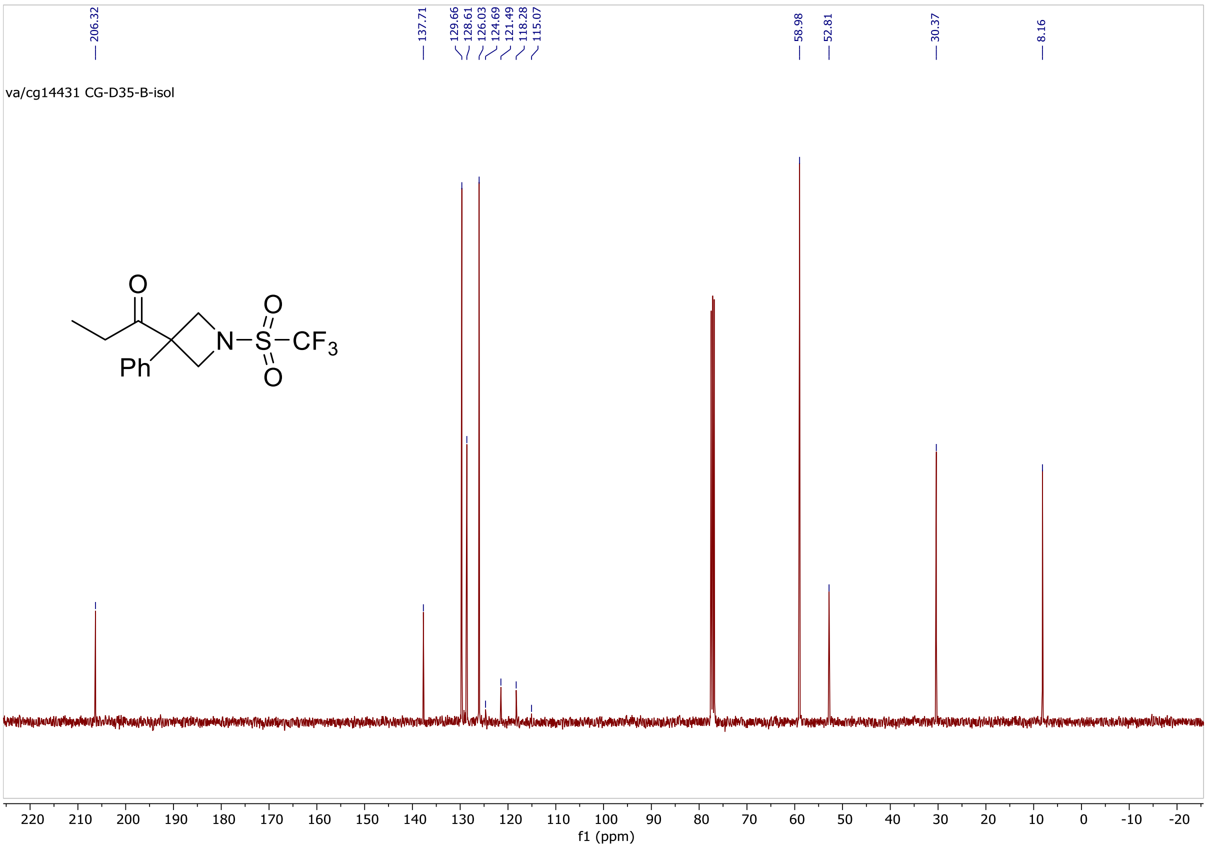

^{1}H NMR ($CDCl_3$, 400 MHz) of **6p**, See procedure

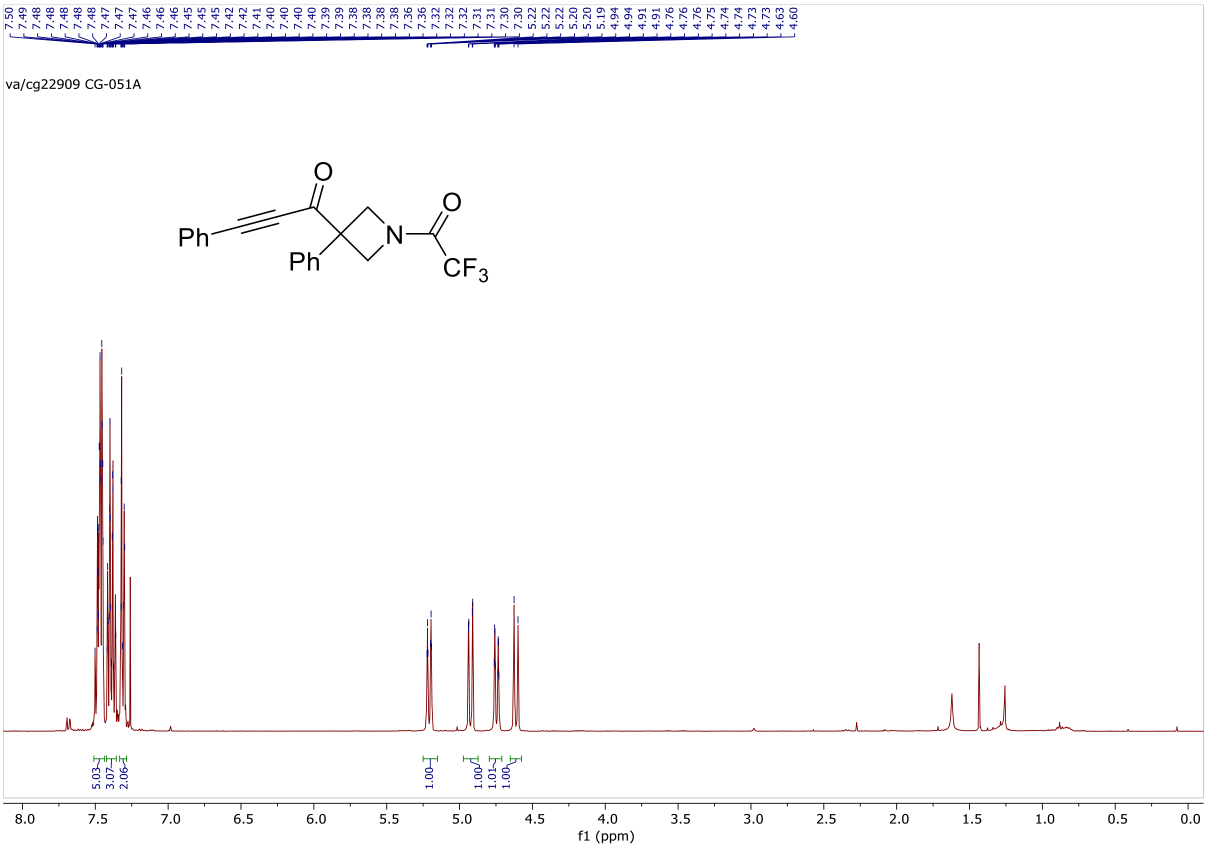

^{13}**C NMR** (CDCl$_3$, 101 MHz) of **6p**

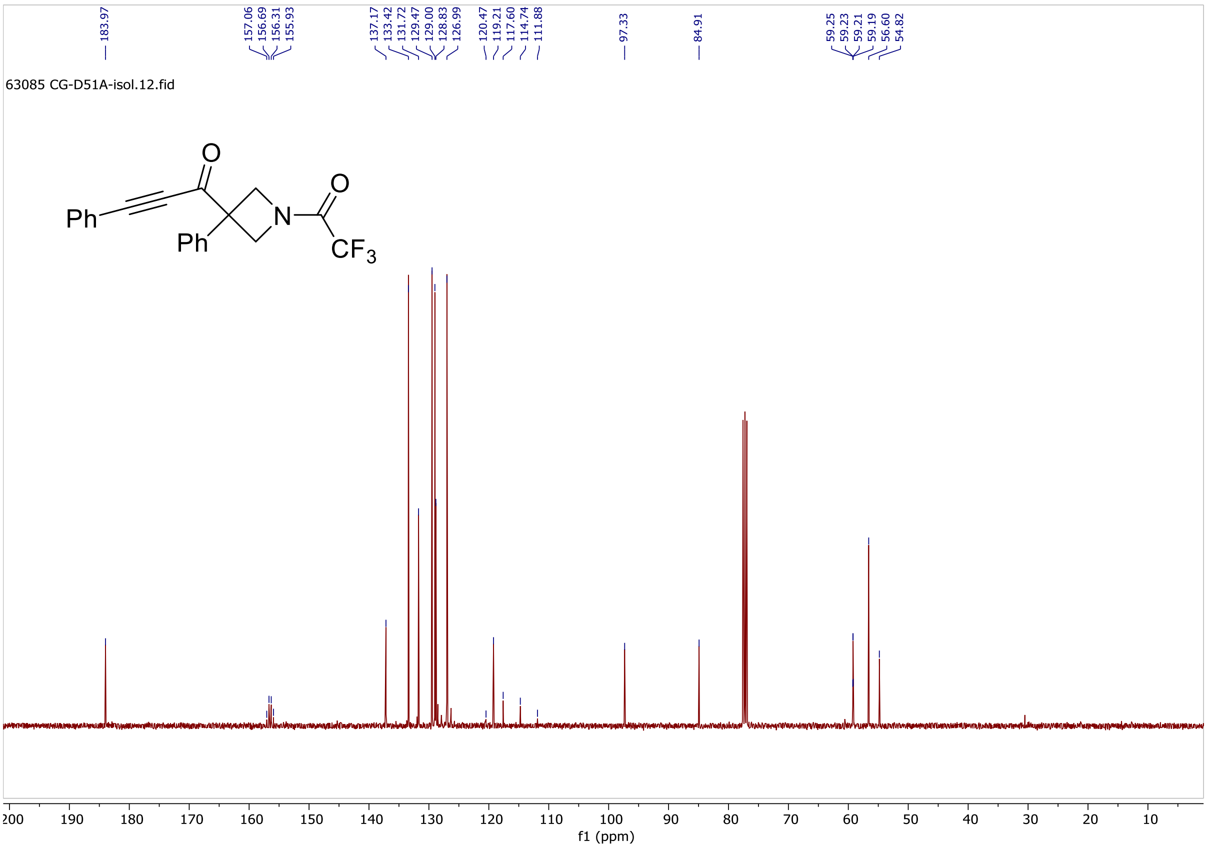

^{1}**H NMR** (CDCl$_3$, 400 MHz) of **7p**, See procedure

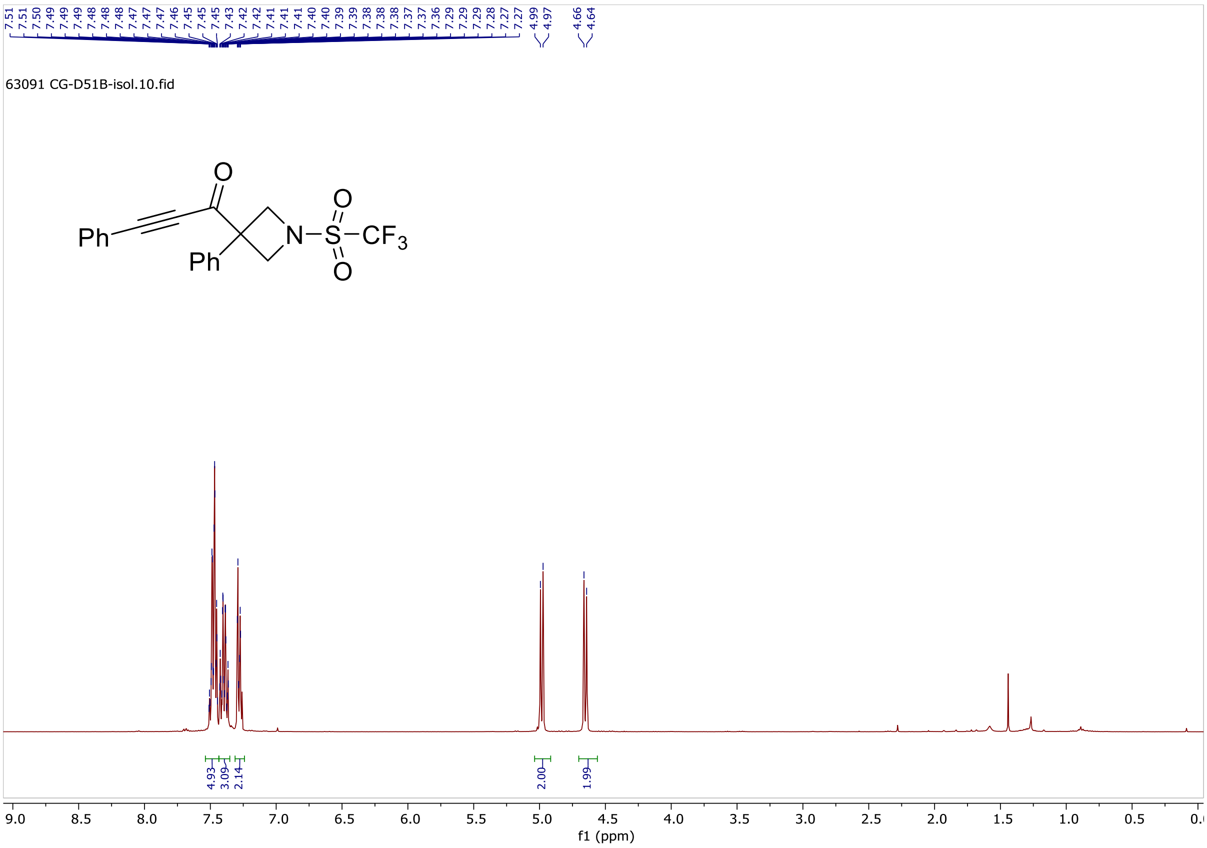

^{13}C NMR ($CDCl_3$, 101 MHz) of **7p**

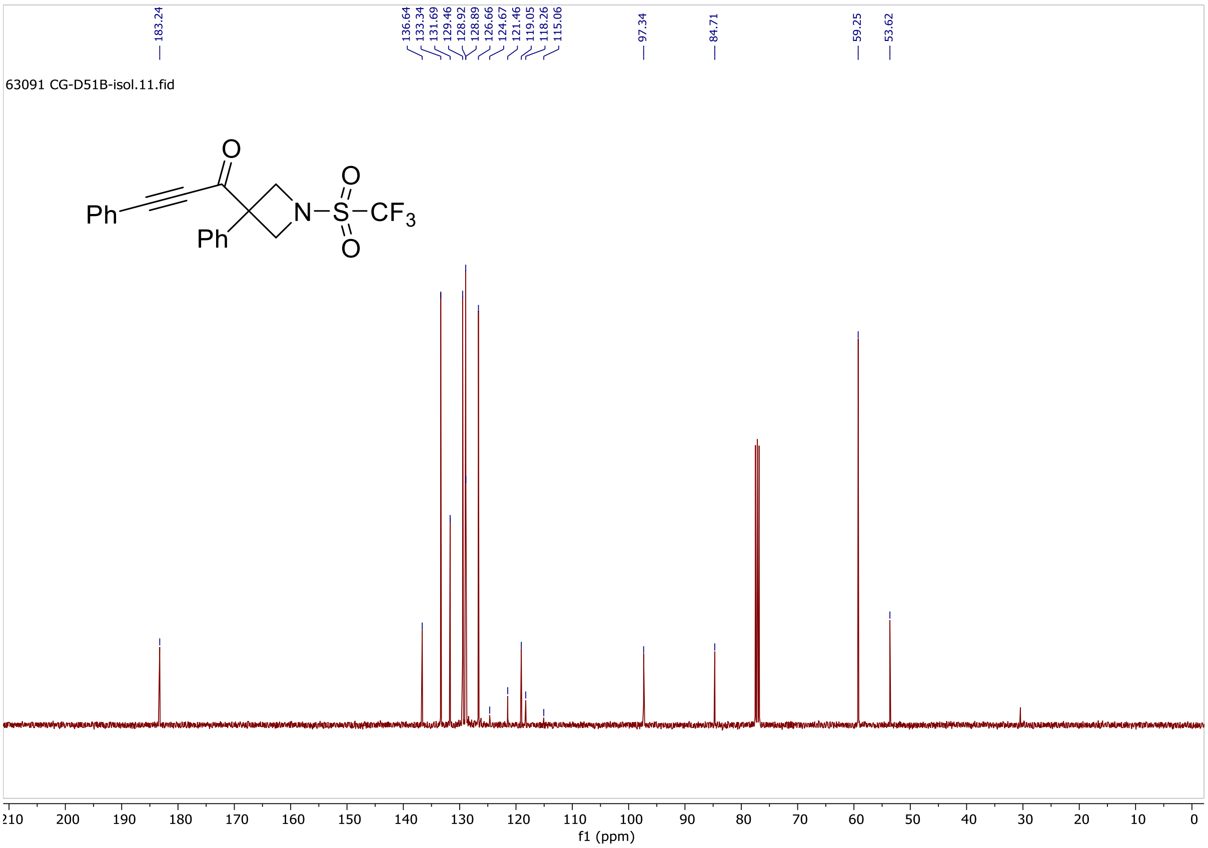

^{1}H NMR ($CDCl_3$, 400 MHz) of **6q**, See procedure

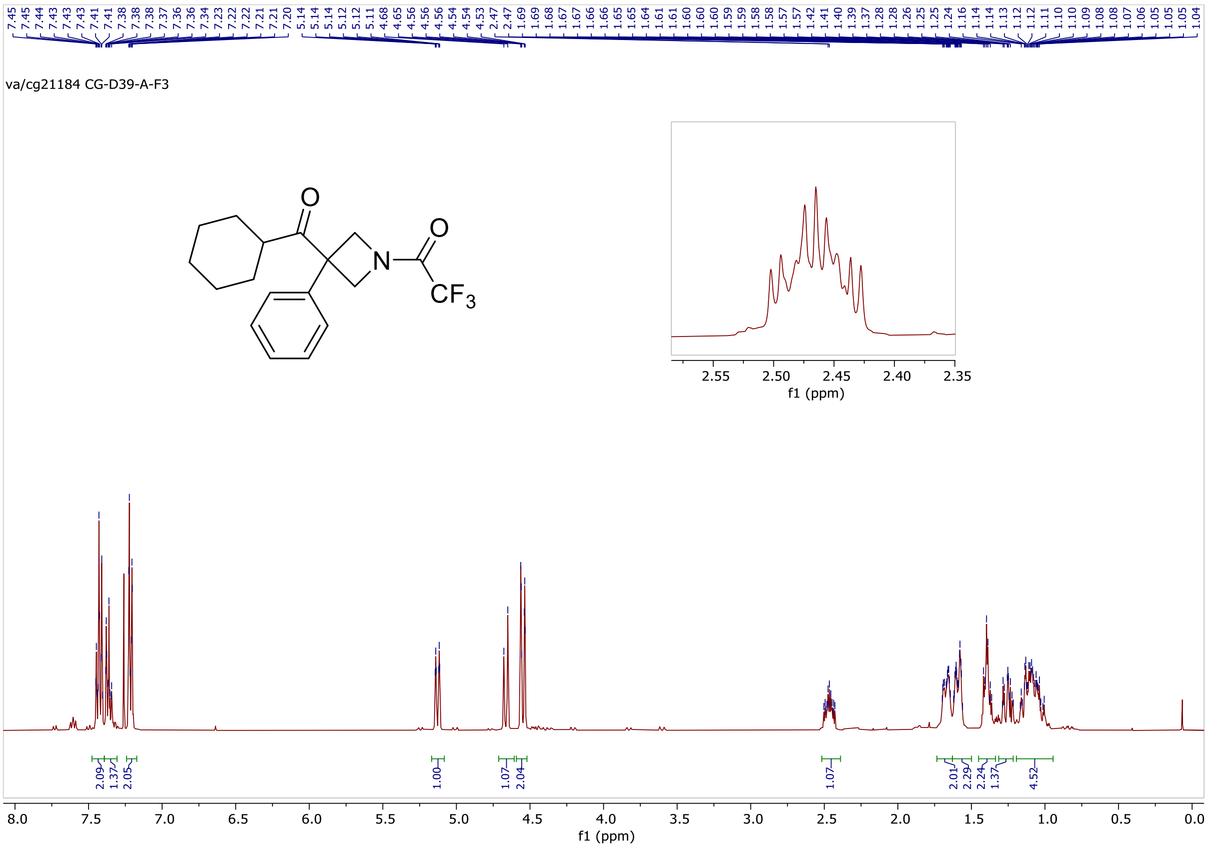

^{13}**C NMR** (CDCl$_3$, 101 MHz) of **6q**

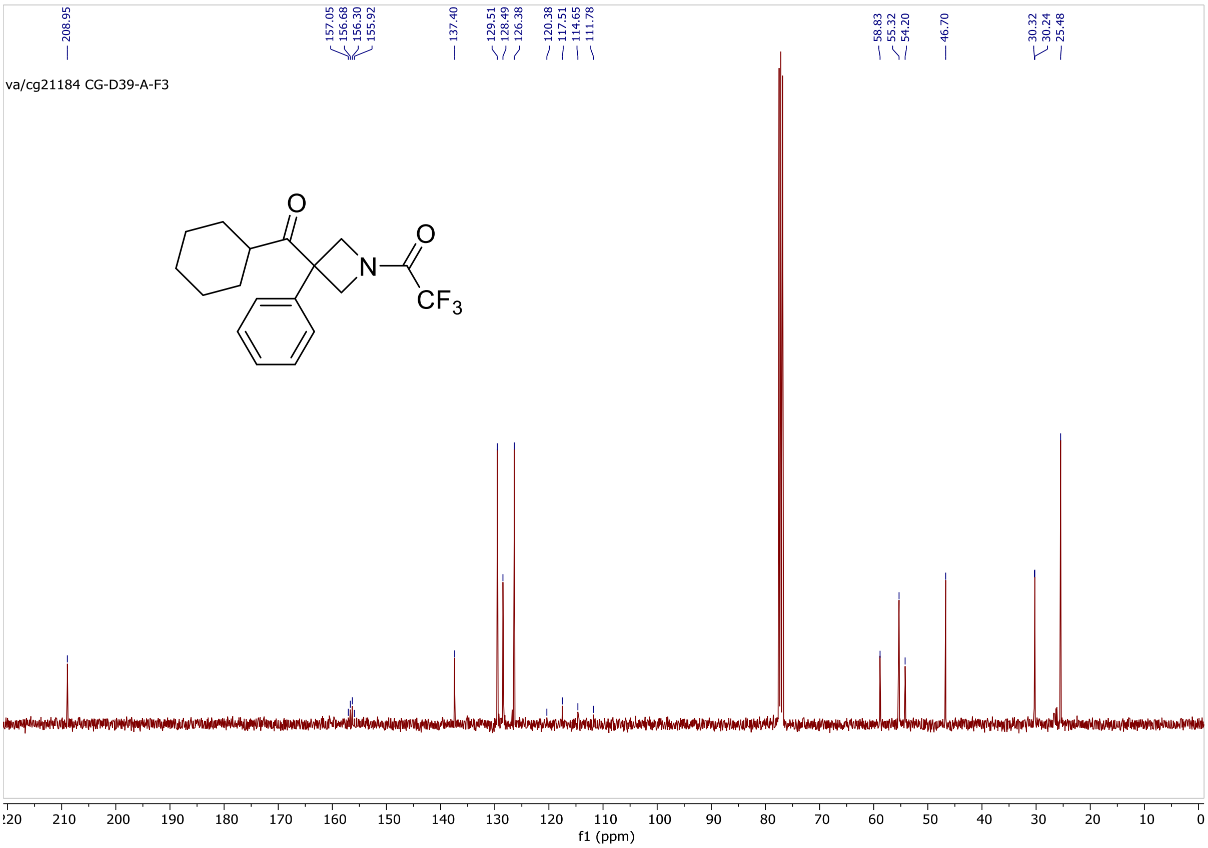

^{1}**H NMR** (CDCl$_3$, 400 MHz) of **13**, See procedure

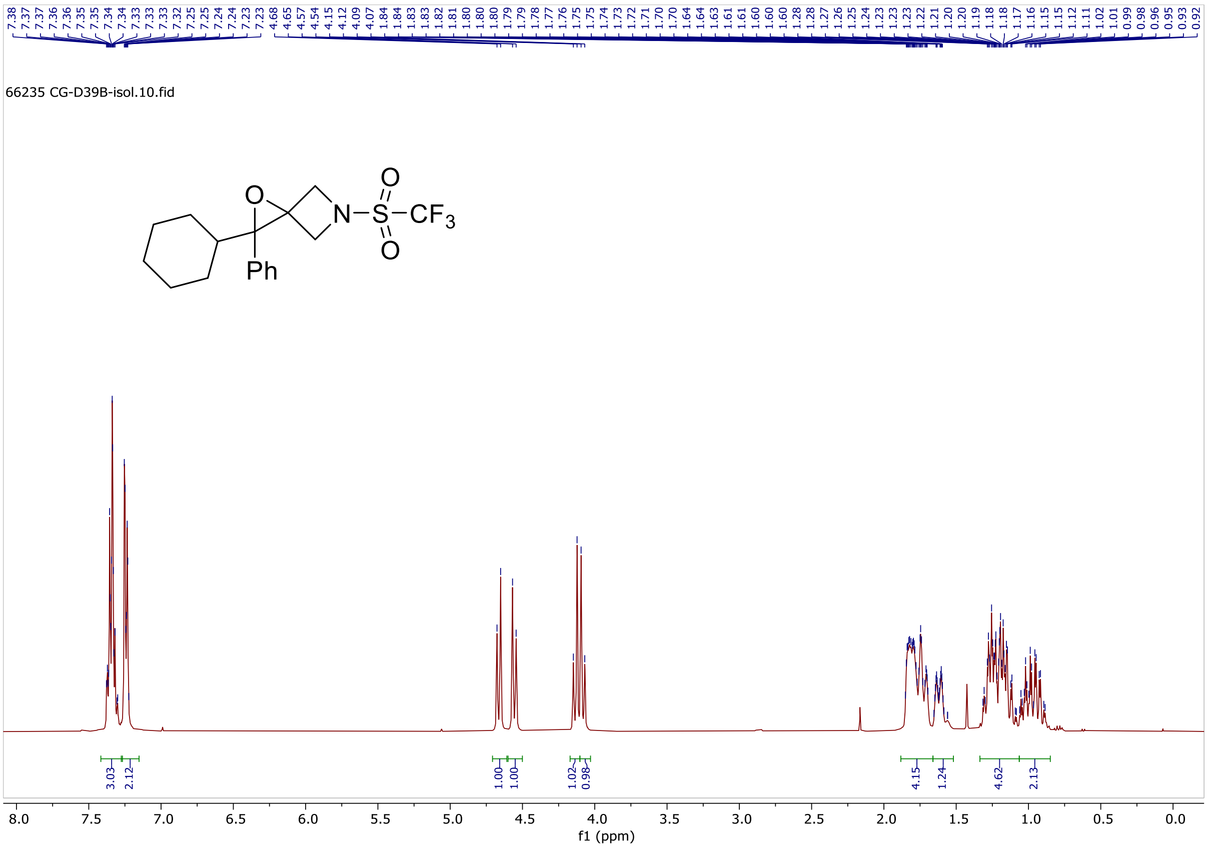

^{13}C NMR ($CDCl_3$, 101 MHz) of 13

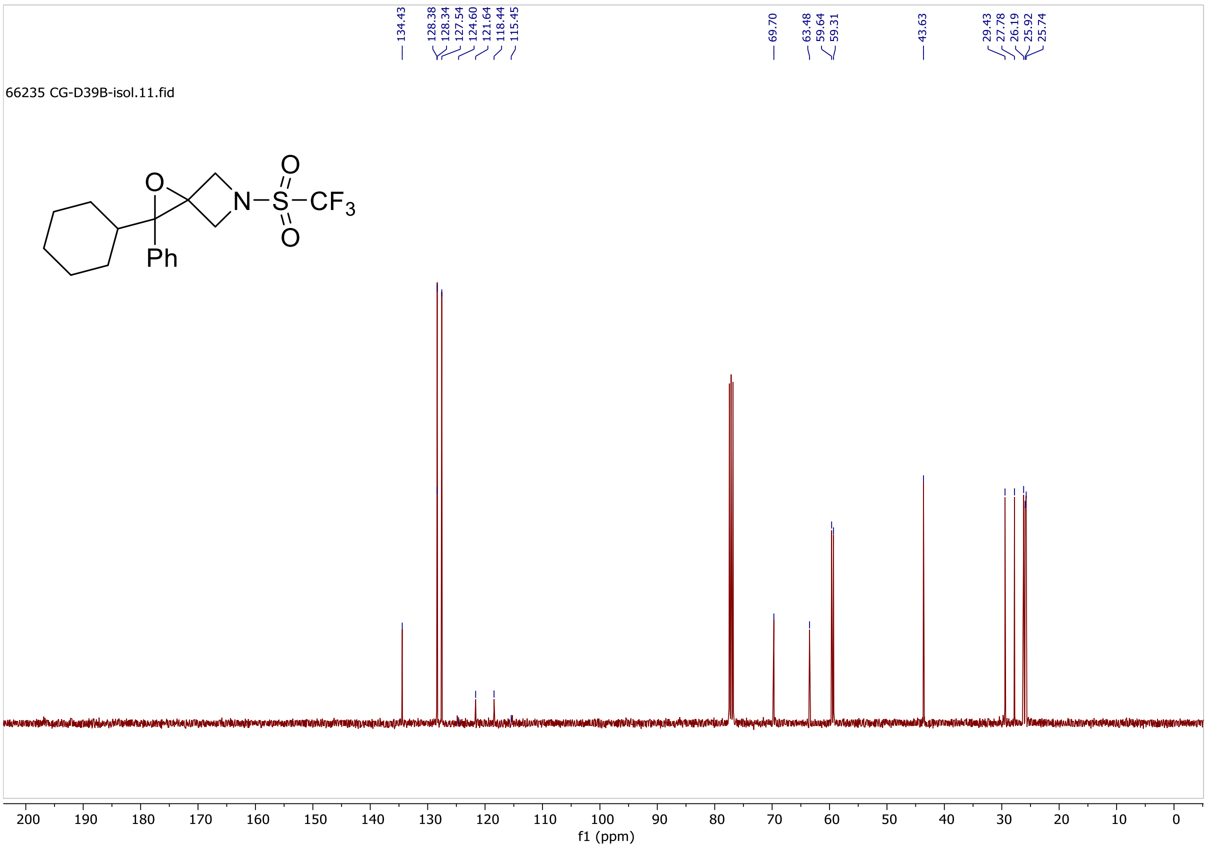

^{1}H NMR ($CDCl_3$, 400 MHz) of 7q and 7q’, See procedure

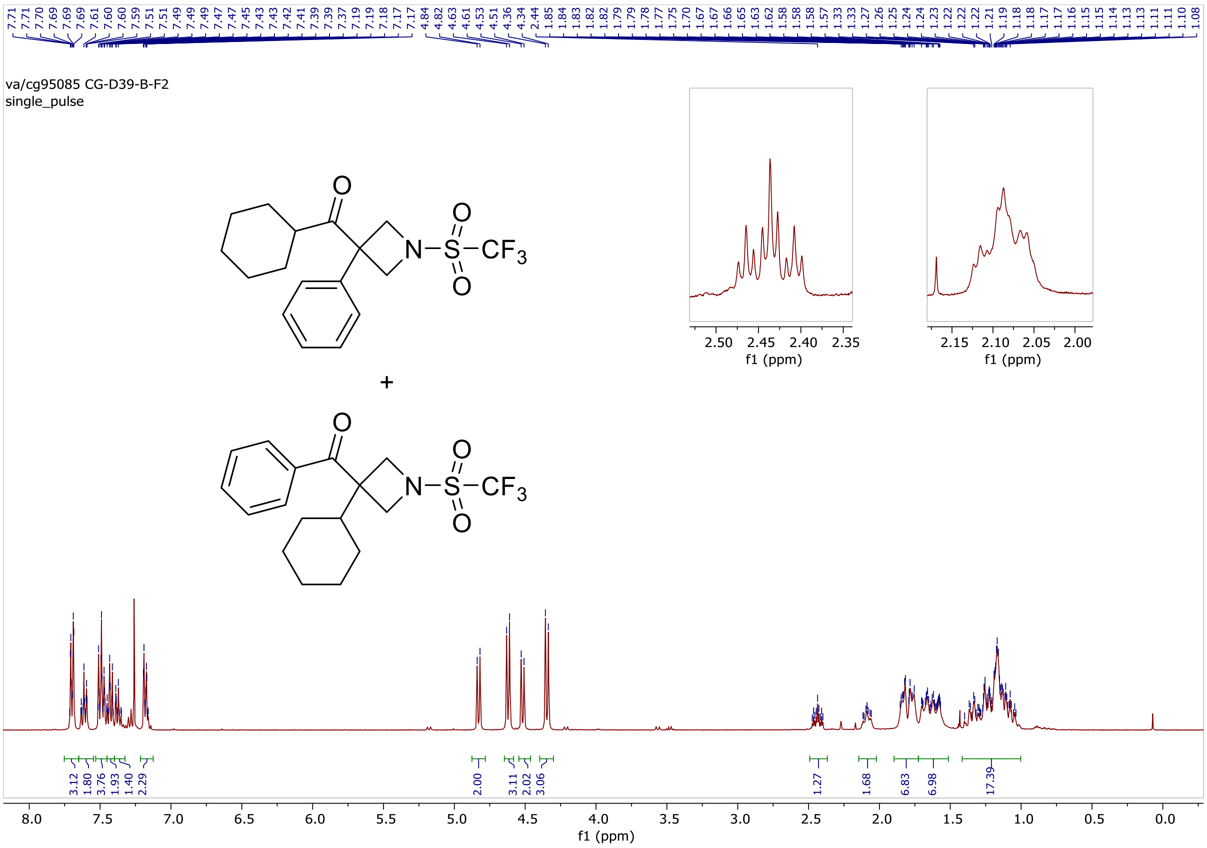

^{13}C NMR ($CDCl_3$, 101 MHz) of **7q** and **7q'**

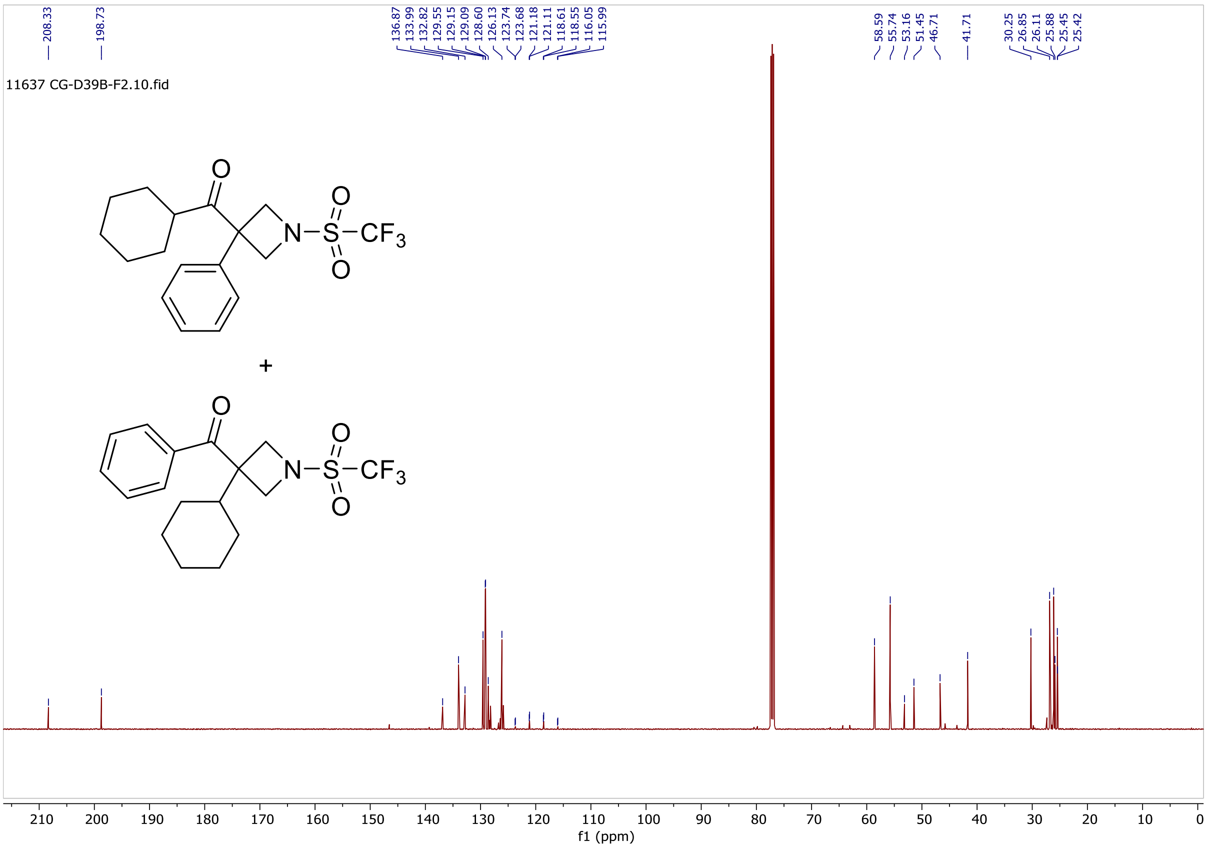

^{1}H NMR (CD_2Cl_2, 400 MHz) of **6r**, See procedure

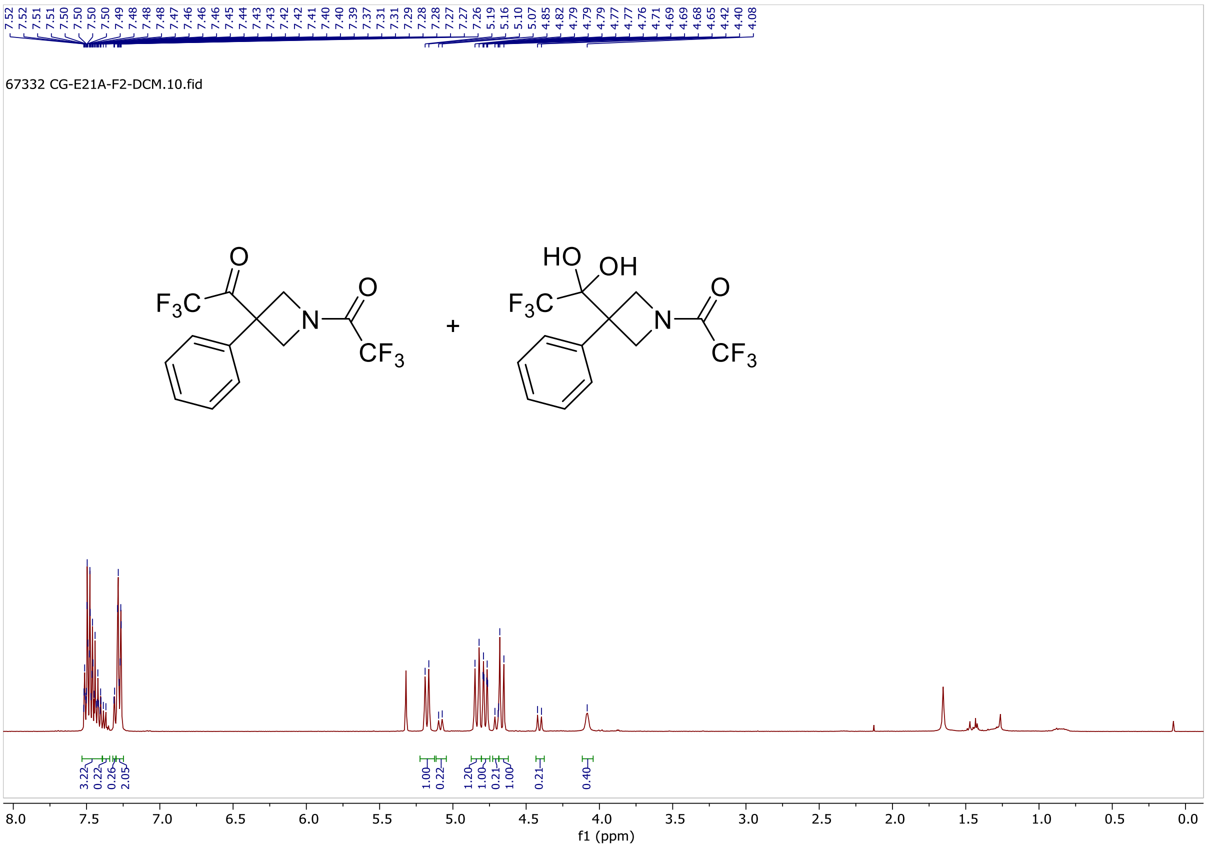

^{13}C NMR (CD_2Cl_2, 101 MHz) of **6r**

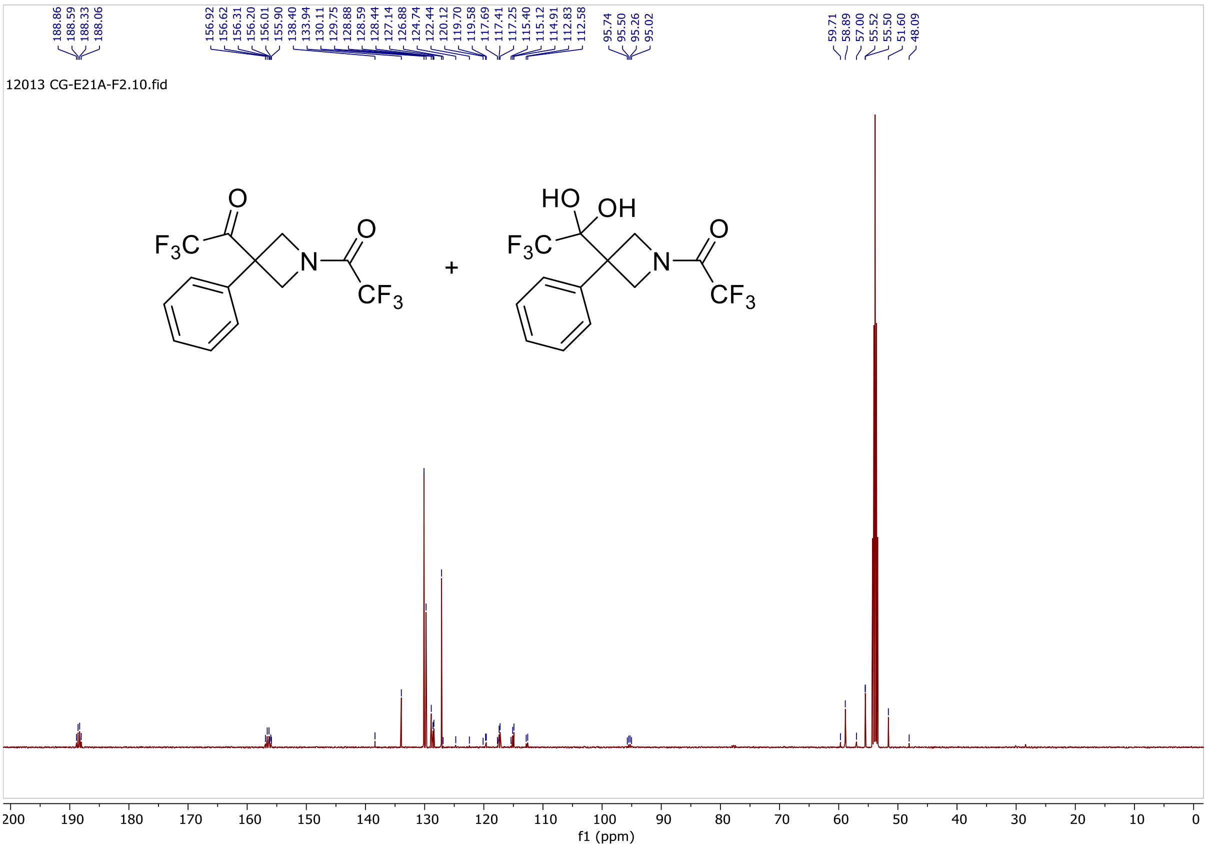

^{1}H NMR (CD_2Cl_2, 400 MHz) of **7r**, See procedure

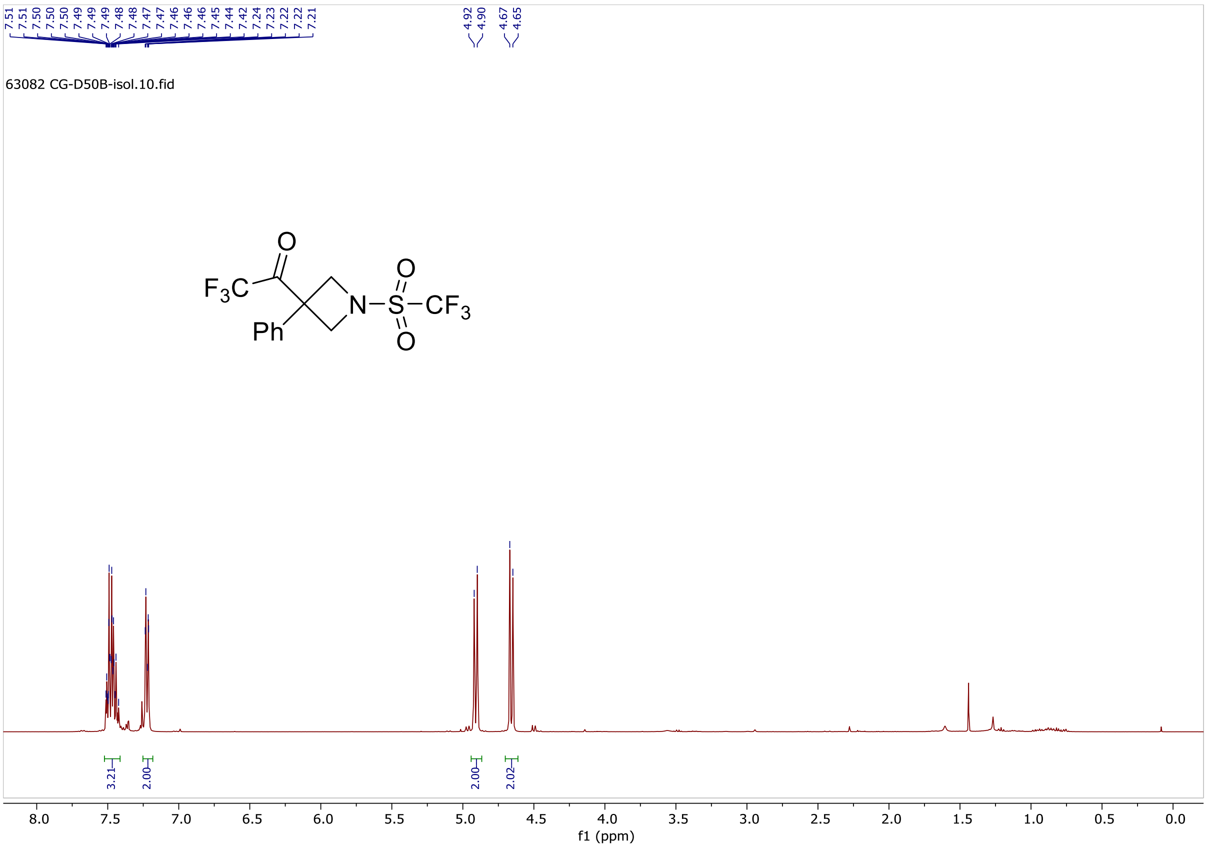

^{13}C NMR (CD_2Cl_2, 101 MHz) of **7r**

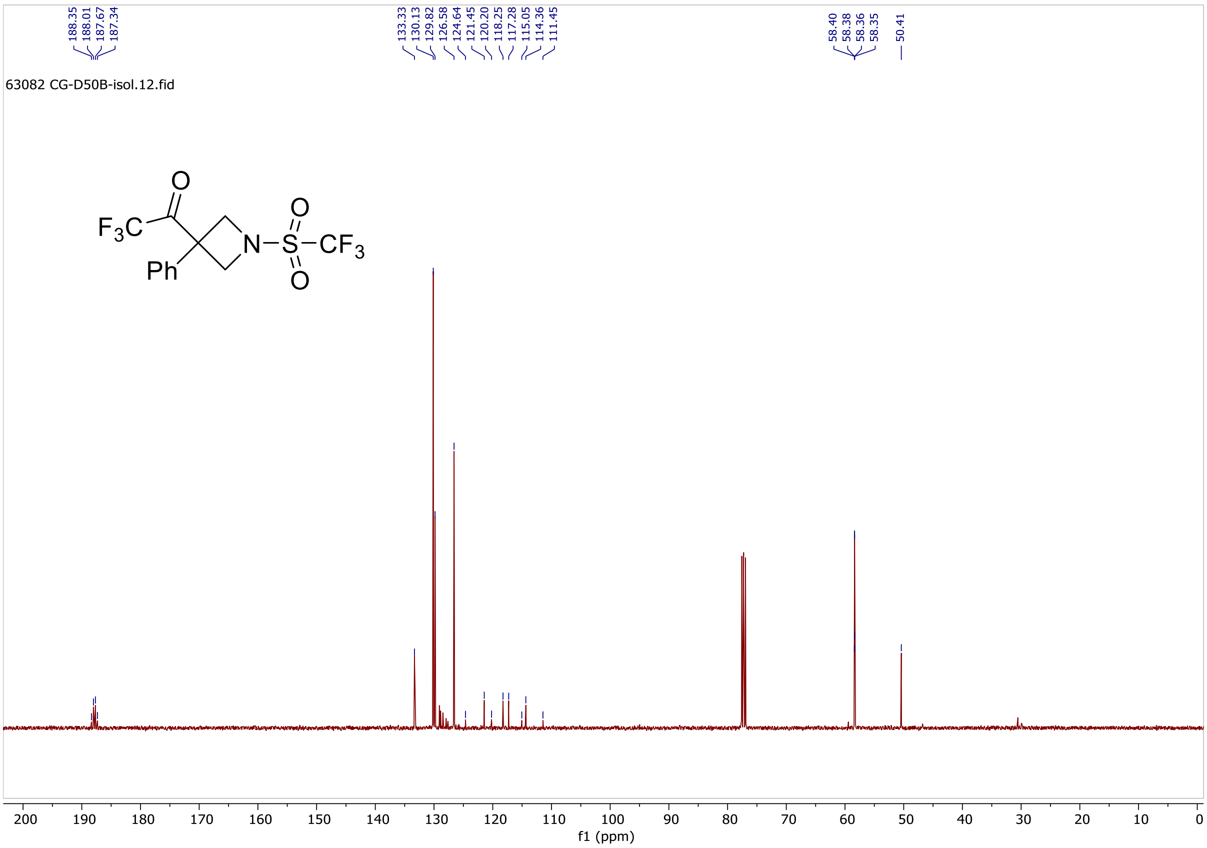

^{1}H NMR ($CDCl_3$, 400 MHz) of **6s**, See procedure

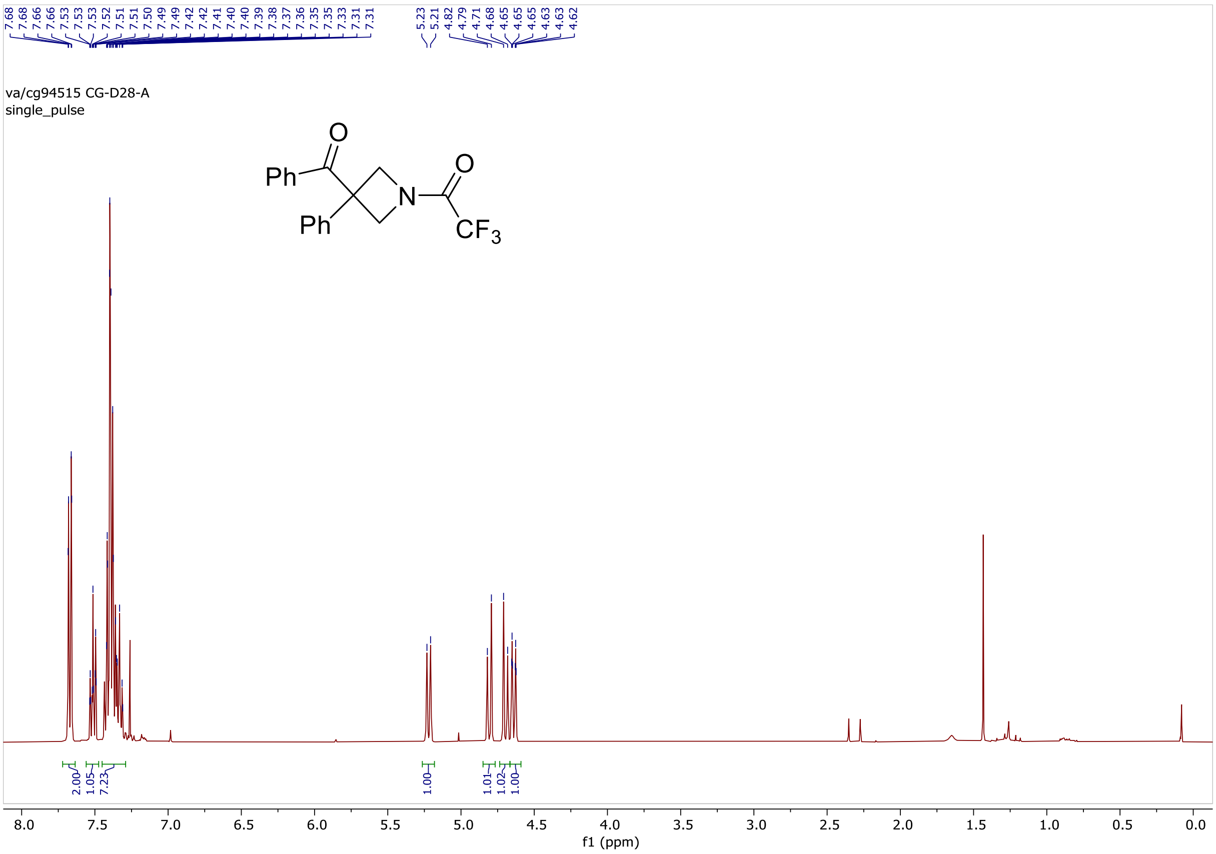

^{13}C NMR (CDCl3, 101 MHz) of **6s**

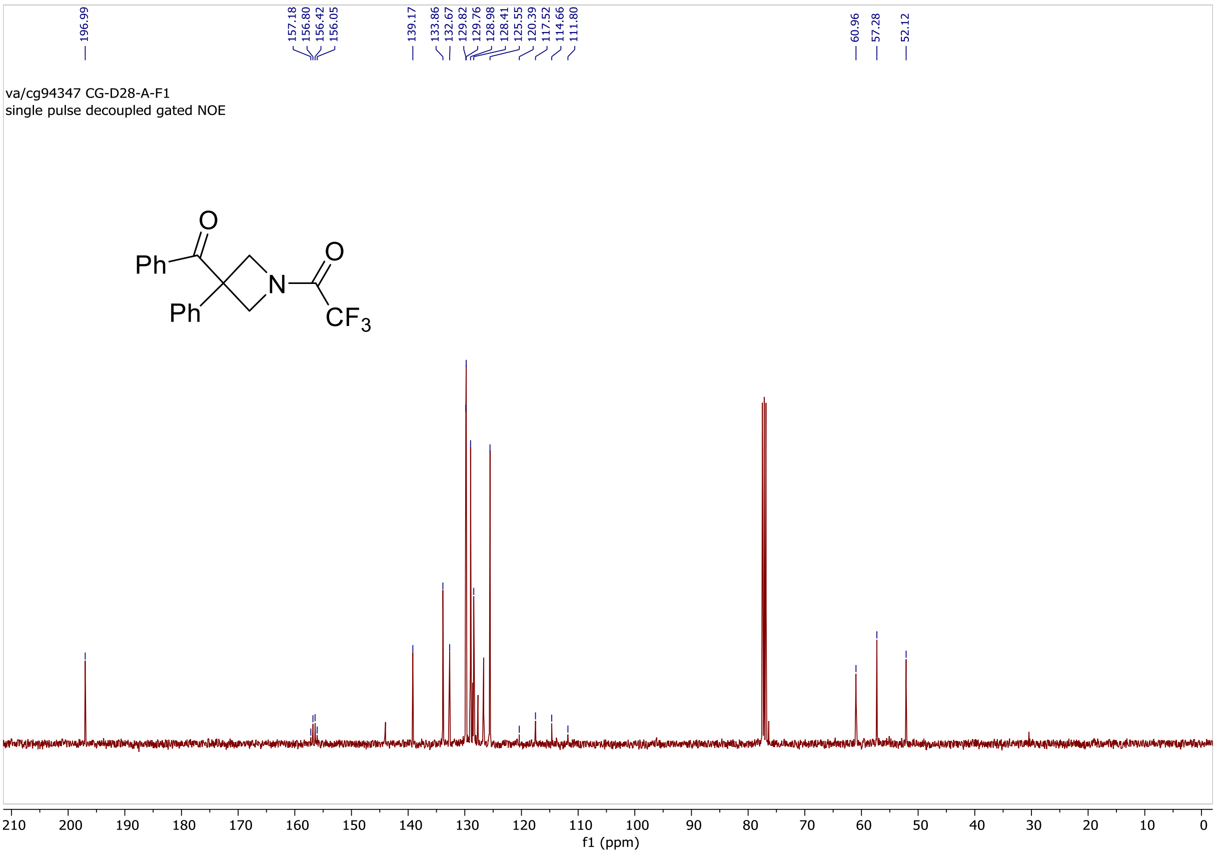

^{1}H NMR (CDCl3, 400 MHz) of **7s**, See procedure

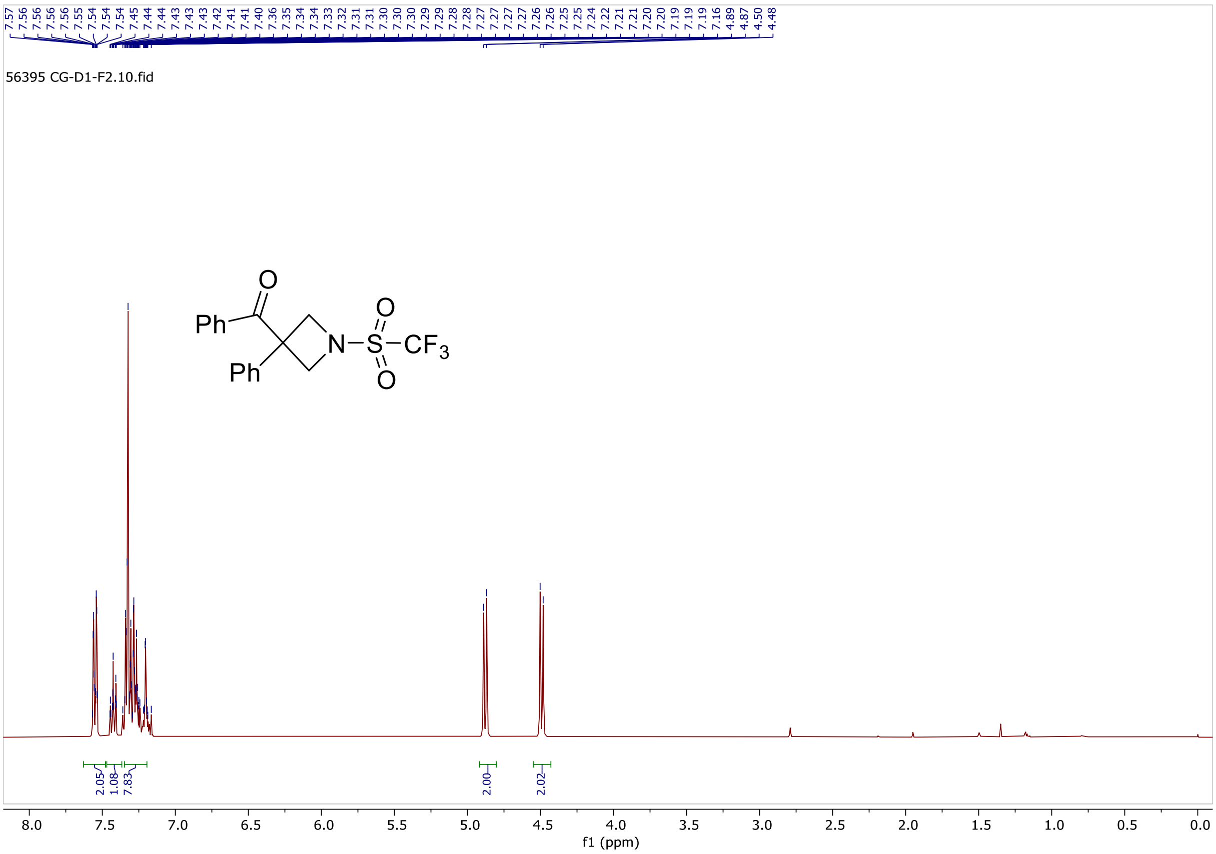

^{13}C NMR ($CDCl_3$, 101 MHz) of **7s**

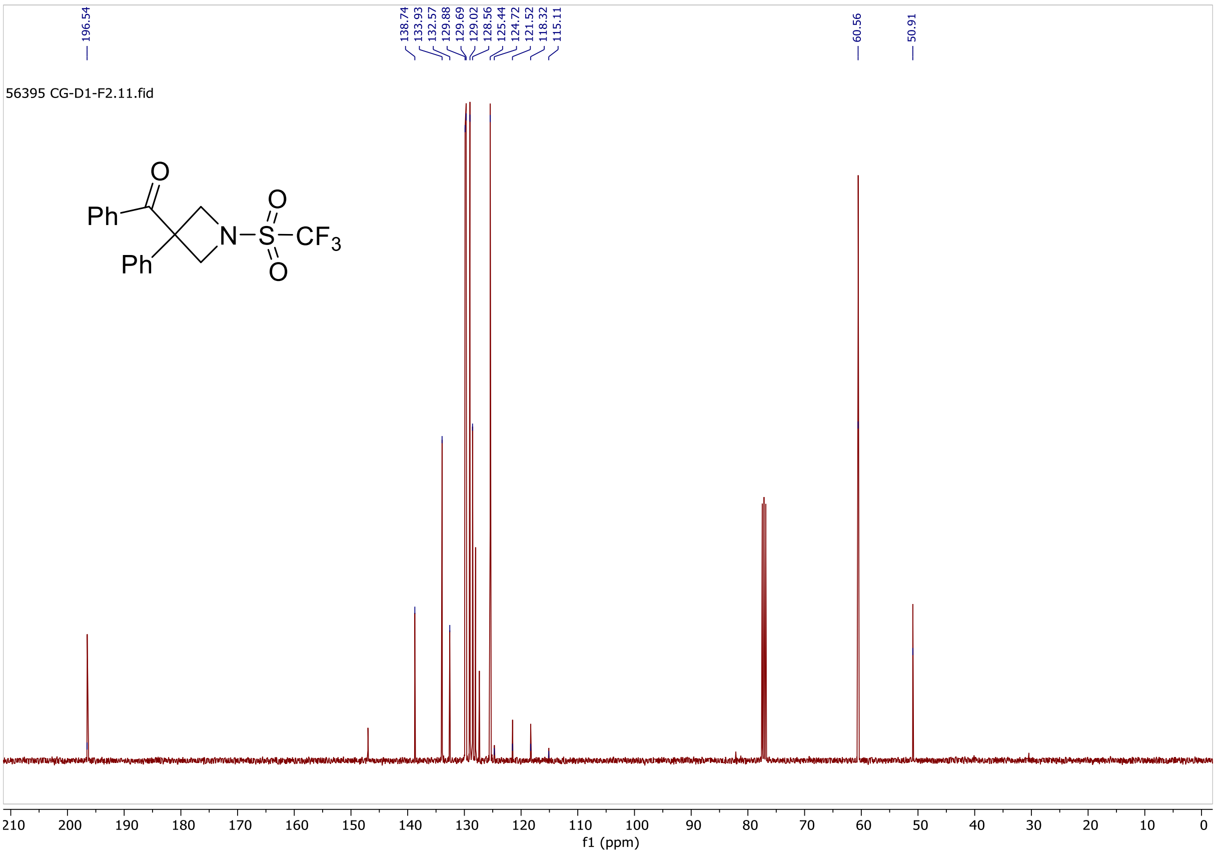

^{1}H NMR ($CDCl_3$, 400 MHz) of **6t**, See procedure

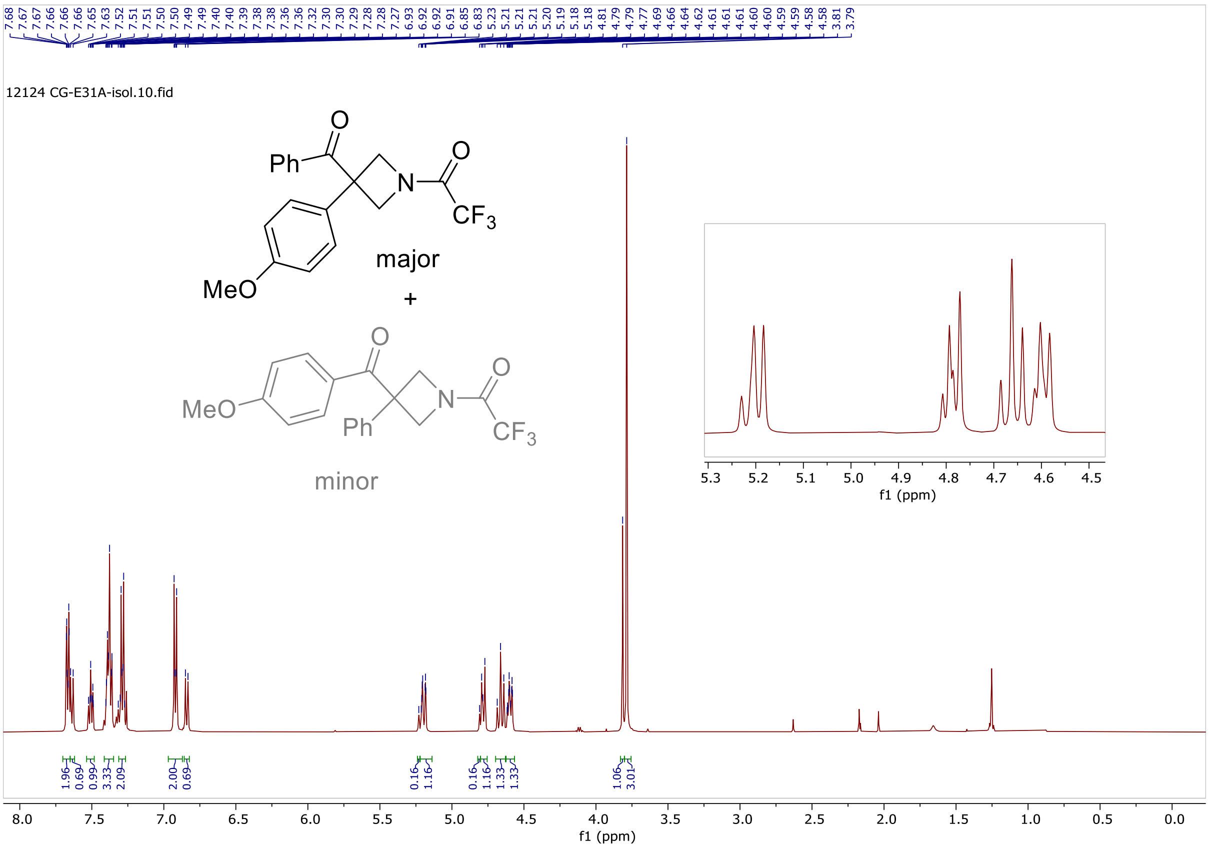

^{13}C NMR (CDCl$_3$, 101 MHz) of **6t**

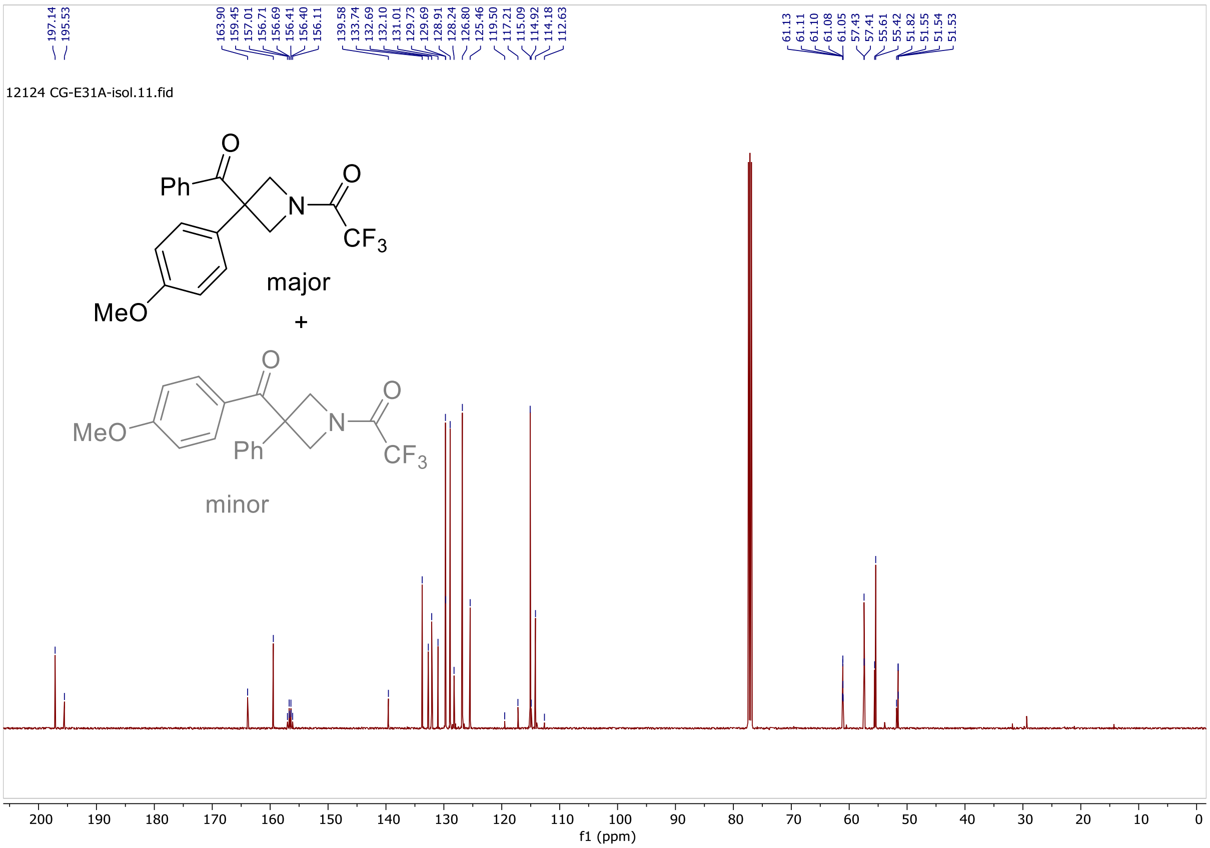

^{1}H NMR (CDCl$_3$, 400 MHz) of **7t** and **7t'**, See procedure

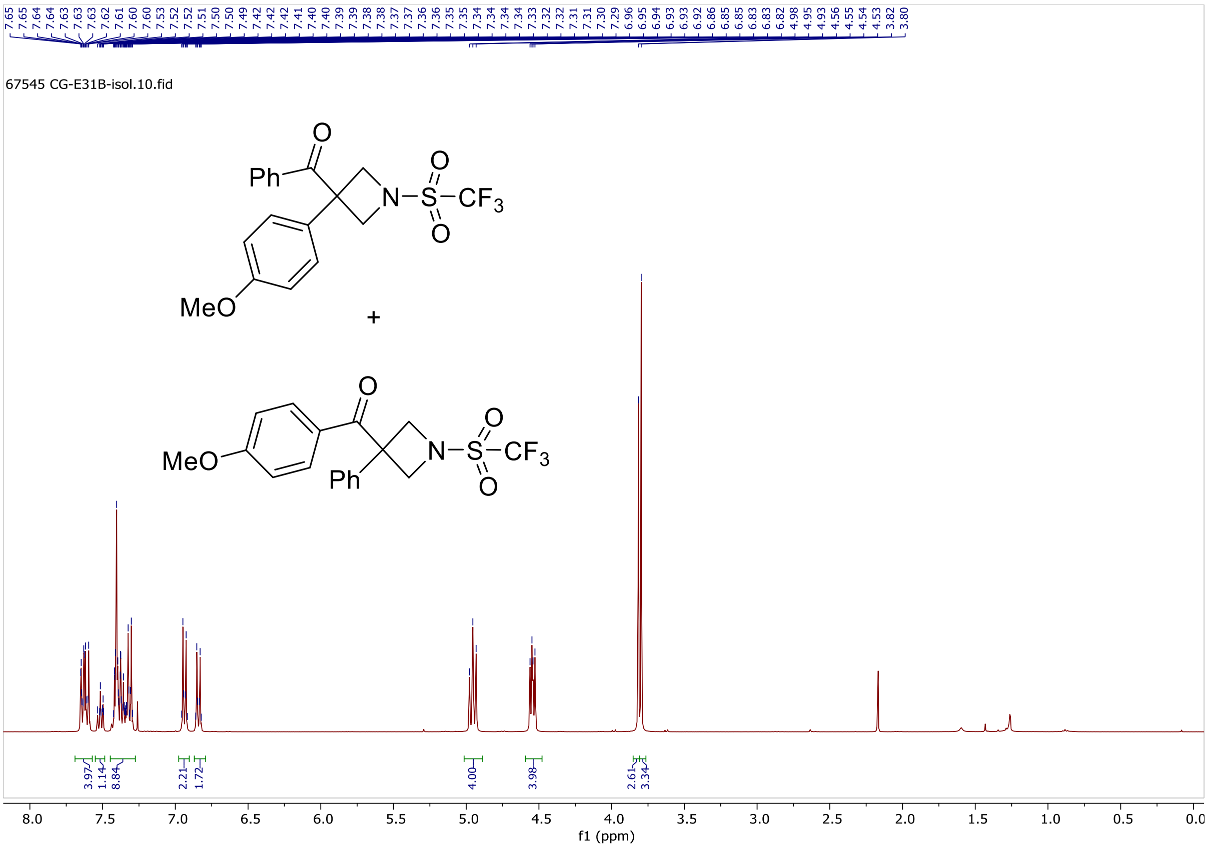

^{13}C NMR ($CDCl_3$, 101 MHz) of **7t** and **7t'**

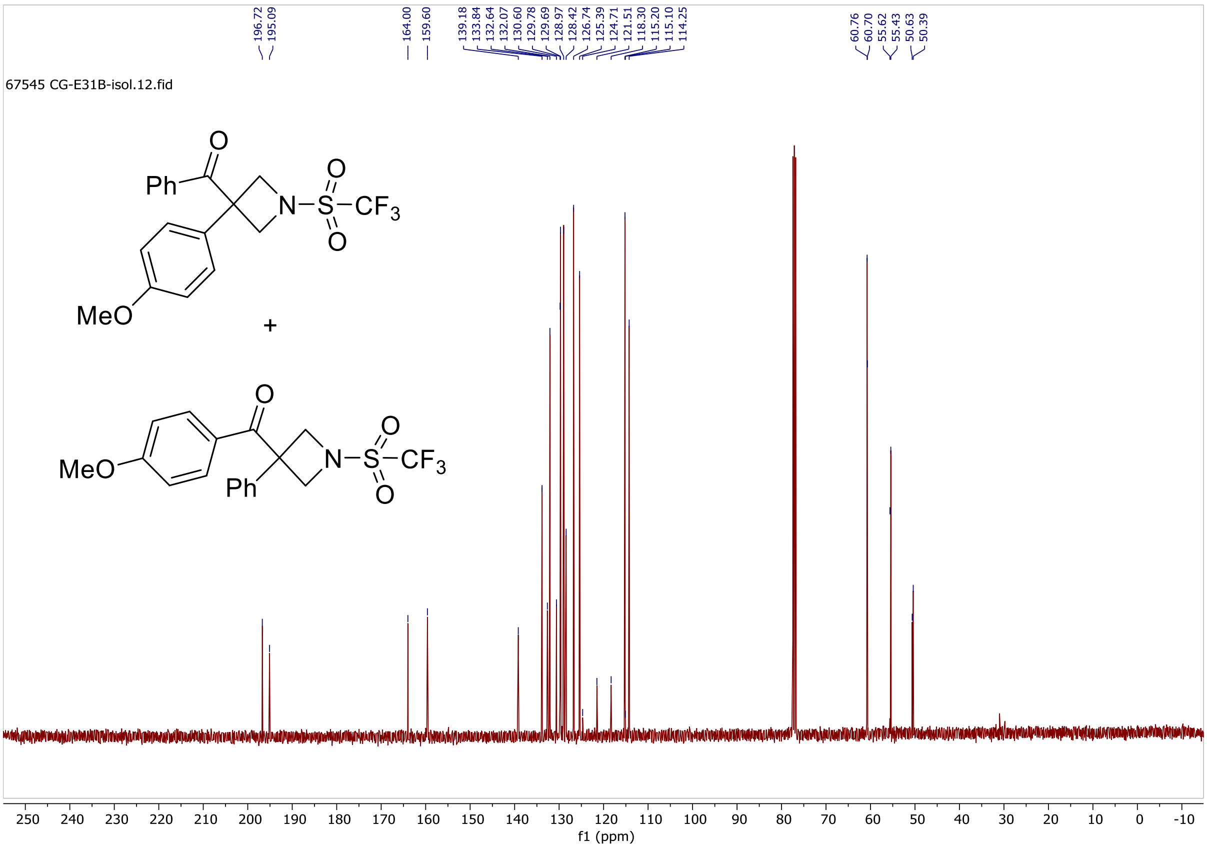

^{1}H NMR ($CDCl_3$, 400 MHz) of **8a**, See procedure

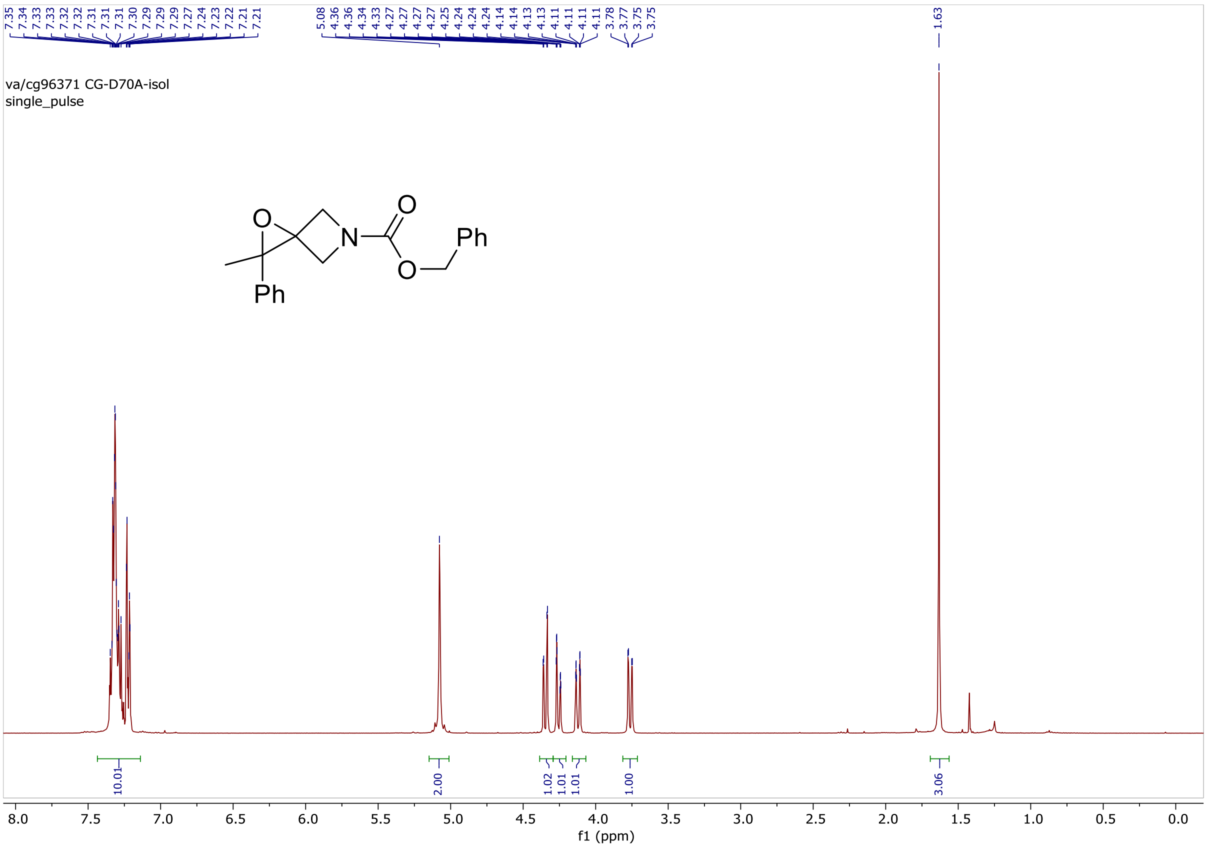

^{13}C NMR ($CDCl_3$, 101 MHz) of **8a**

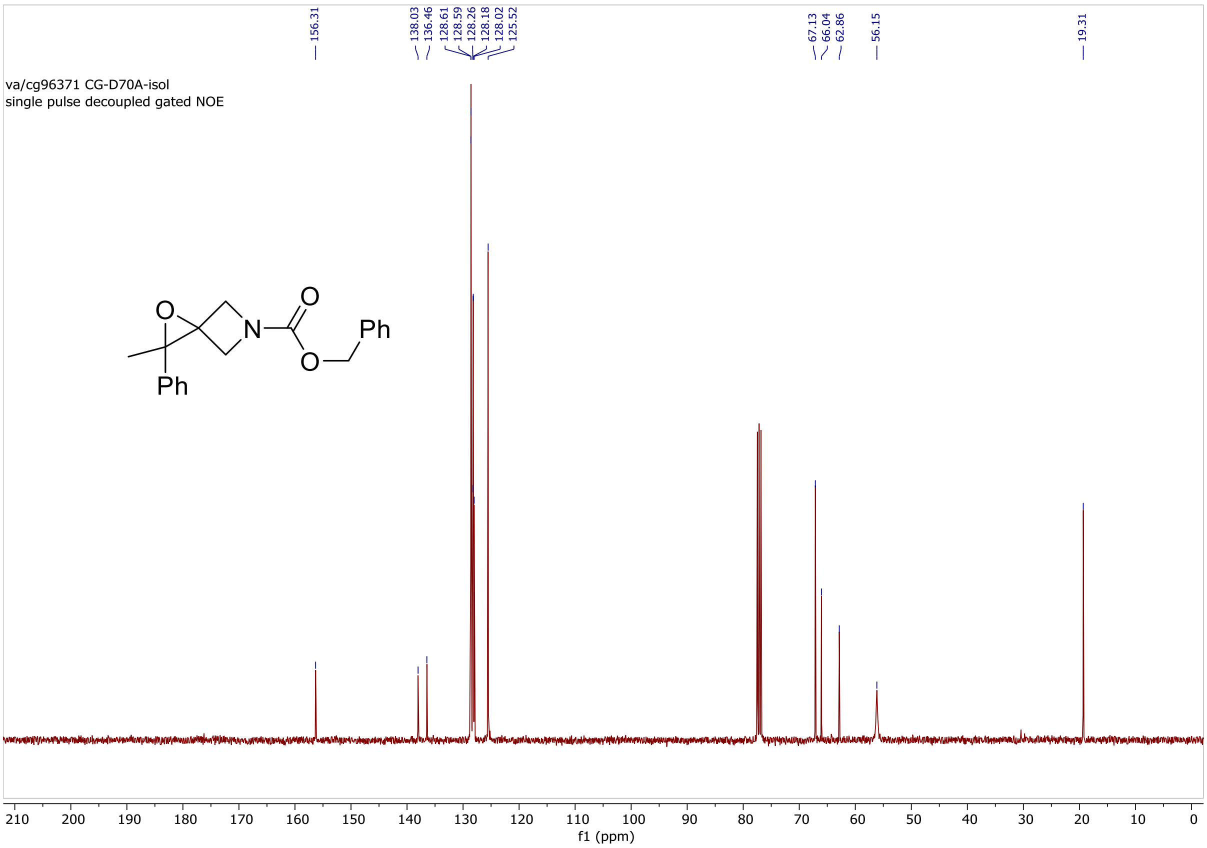

^{1}H NMR ($CDCl_3$, 400 MHz) of **8d'**, See procedure

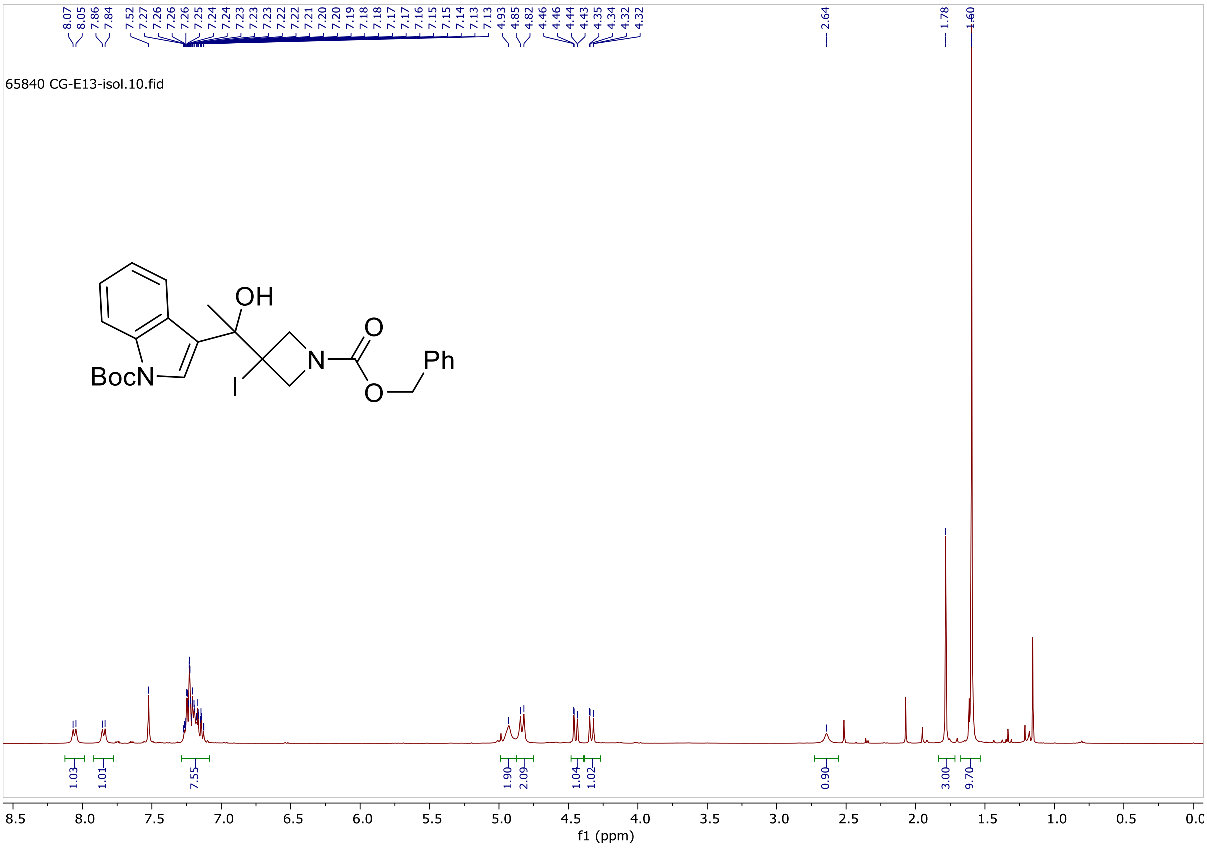

^{13}**C NMR** ($CDCl_3$, 101 MHz) of **8d'**

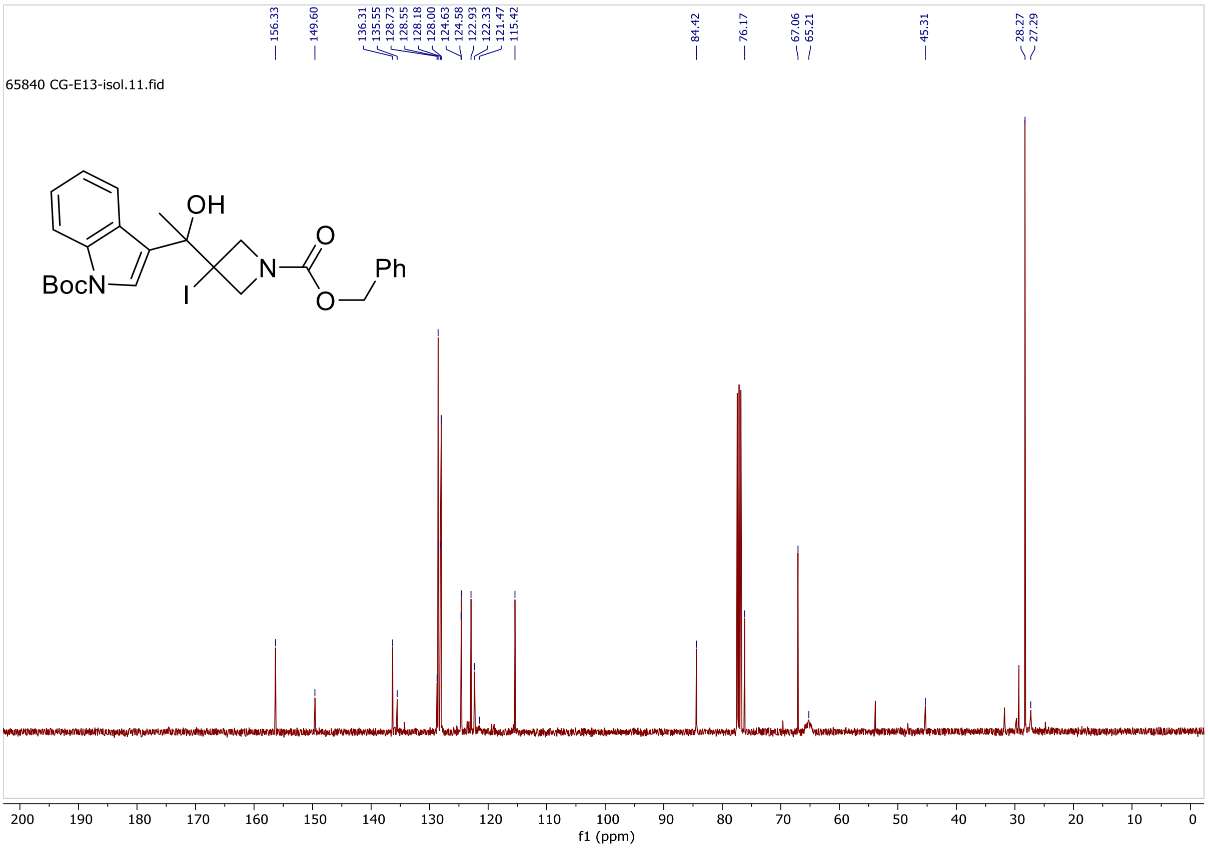

^{1}**H NMR** ($CDCl_3$, 400 MHz) of **8d**, See procedure

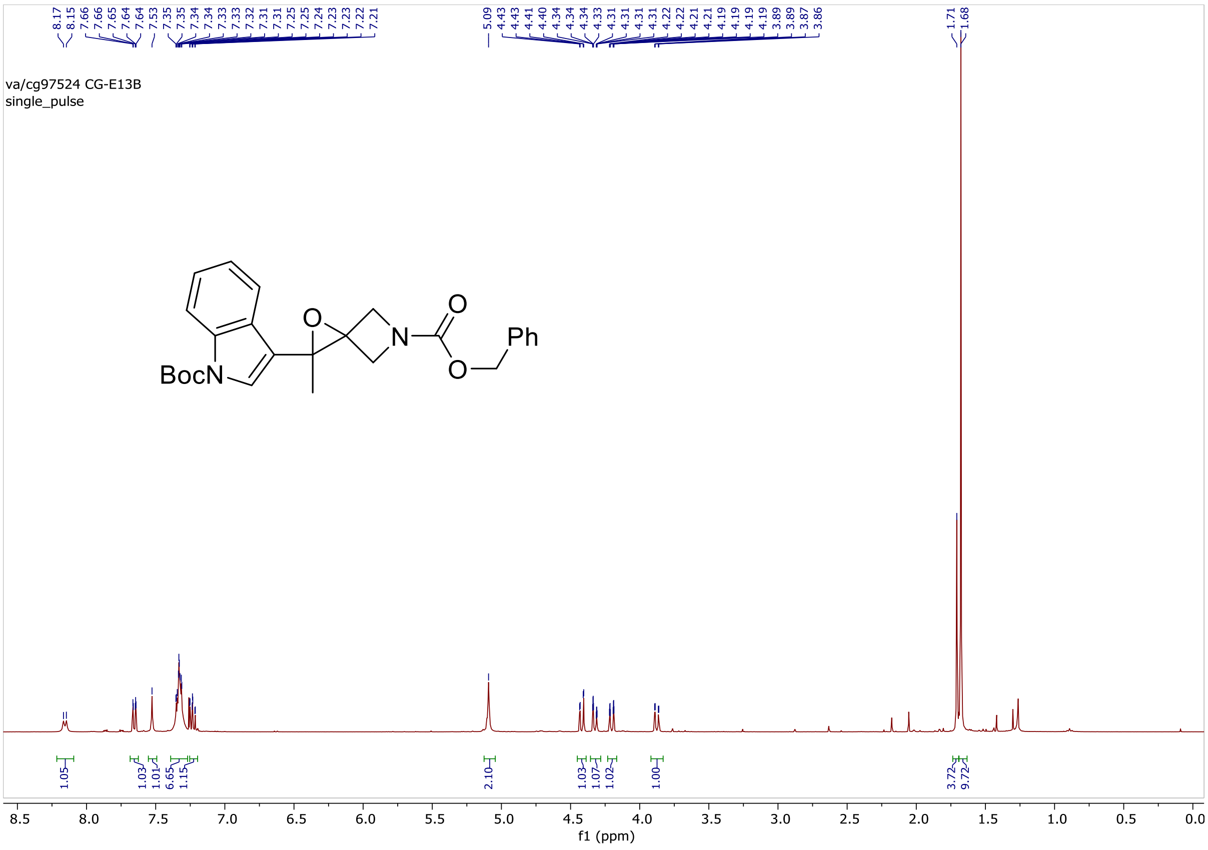

^{13}**C NMR** ($CDCl_3$, 101 MHz) of **8d**

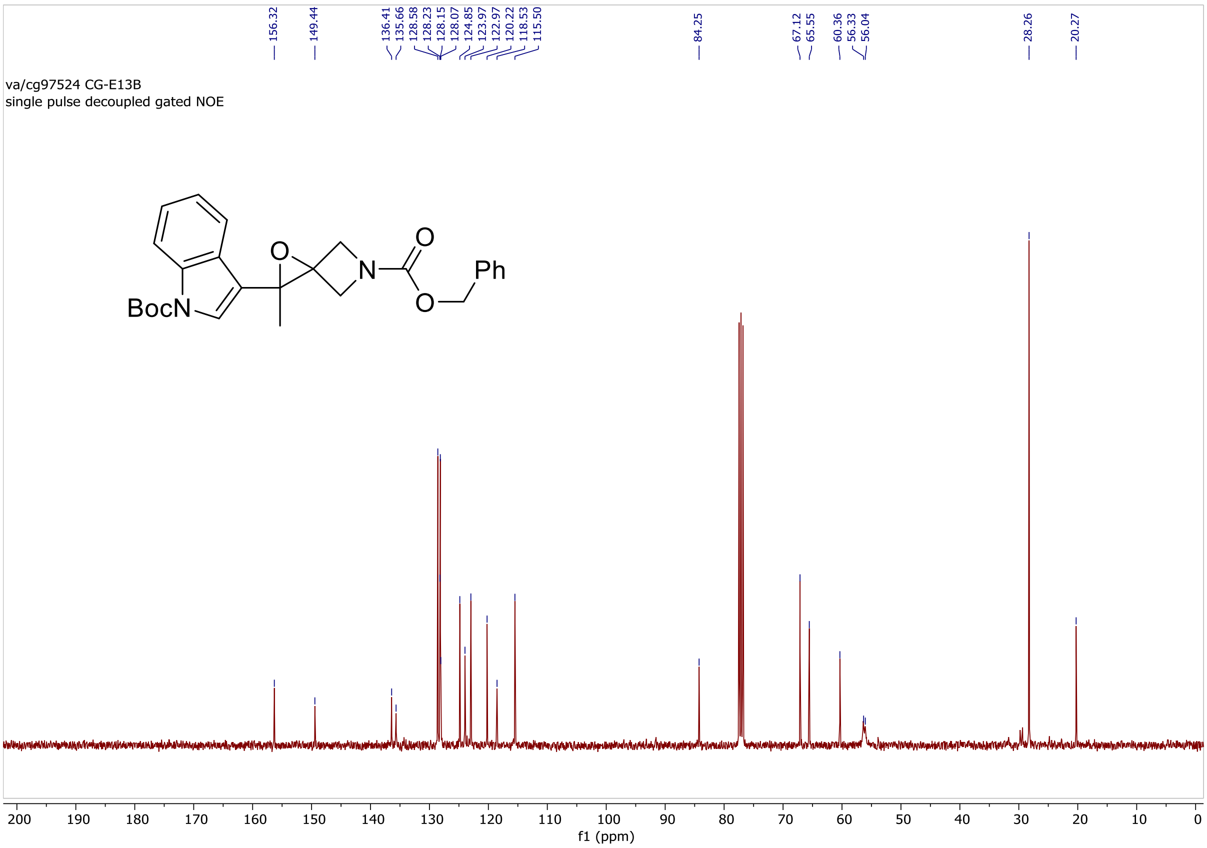

^{1}**H NMR** ($CDCl_3$, 400 MHz) of **8f**, See procedure

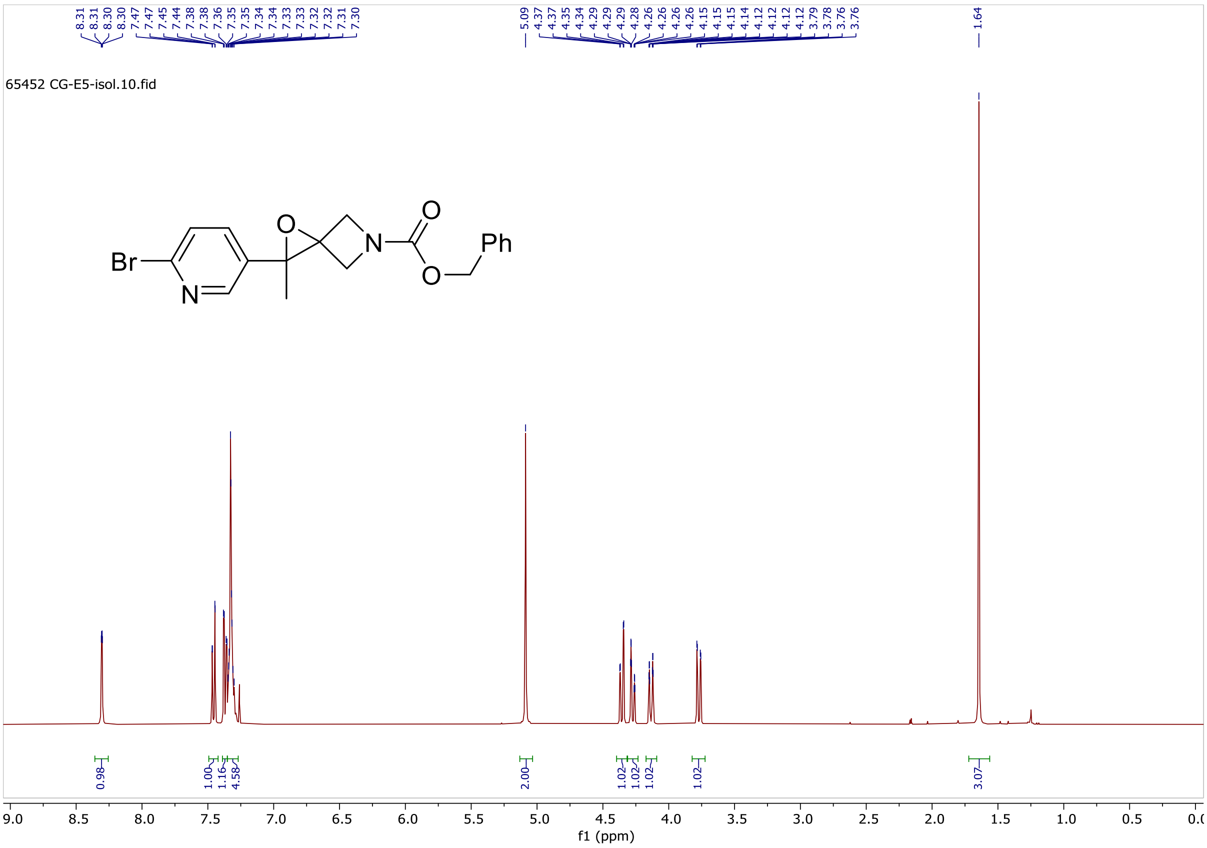

^{13}C NMR ($CDCl_3$, 101 MHz) of **8f**

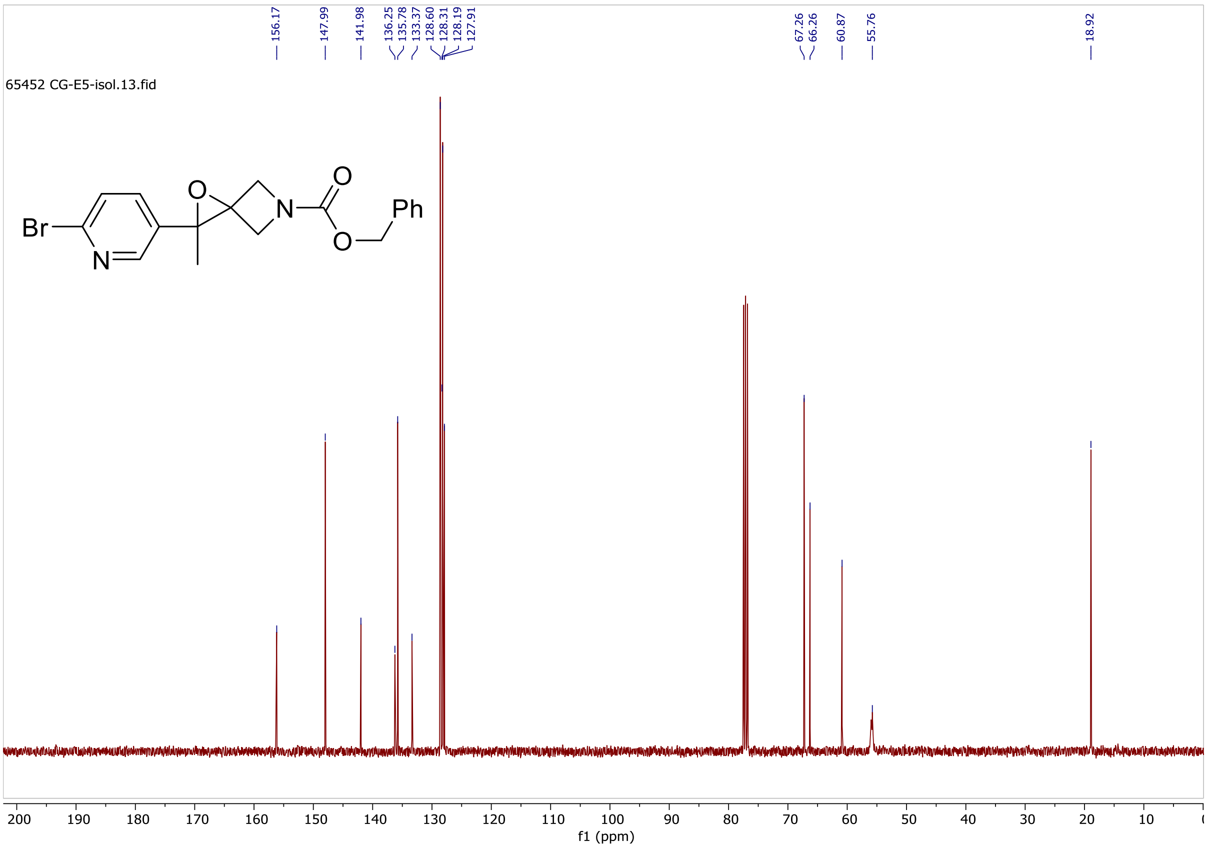

^{1}H NMR ($CDCl_3$, 400 MHz) of **8j**, See procedure

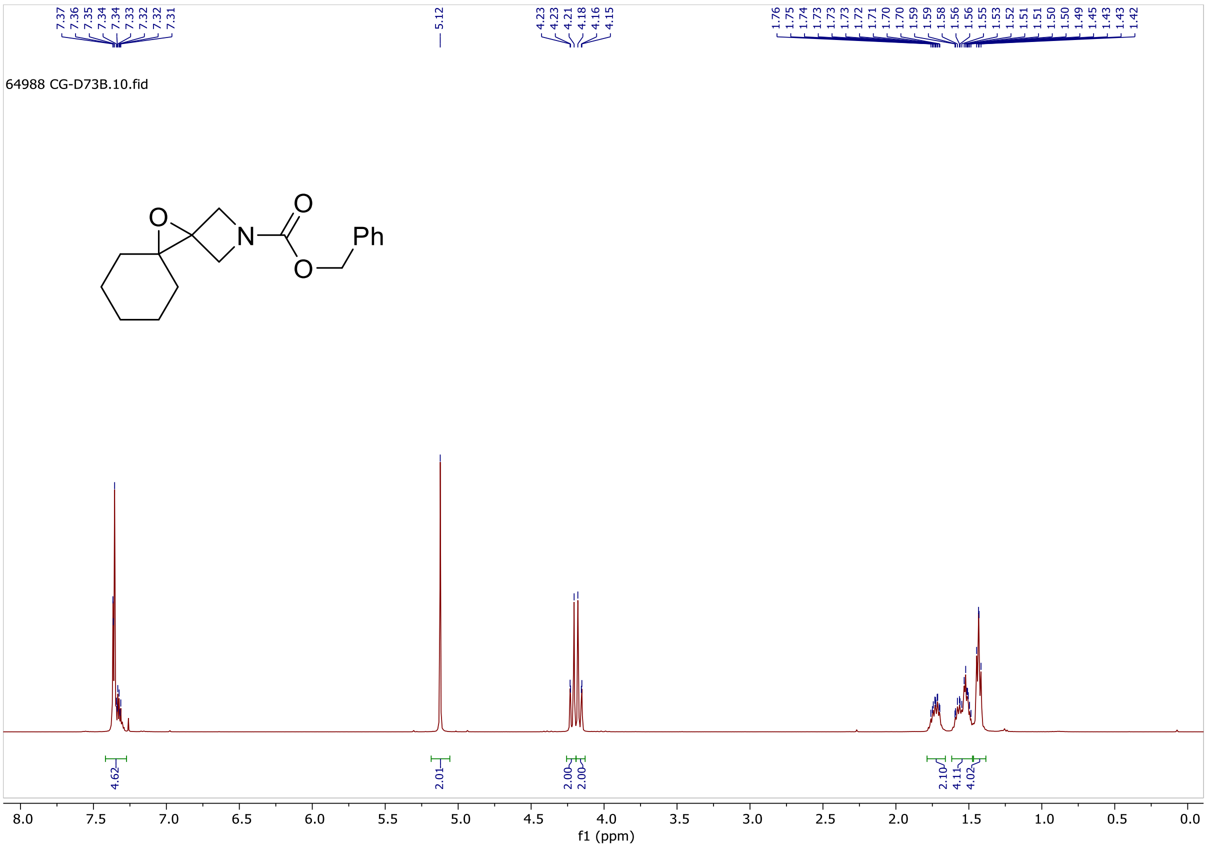

^{13}C NMR ($CDCl_3$, 101 MHz) of **8j**

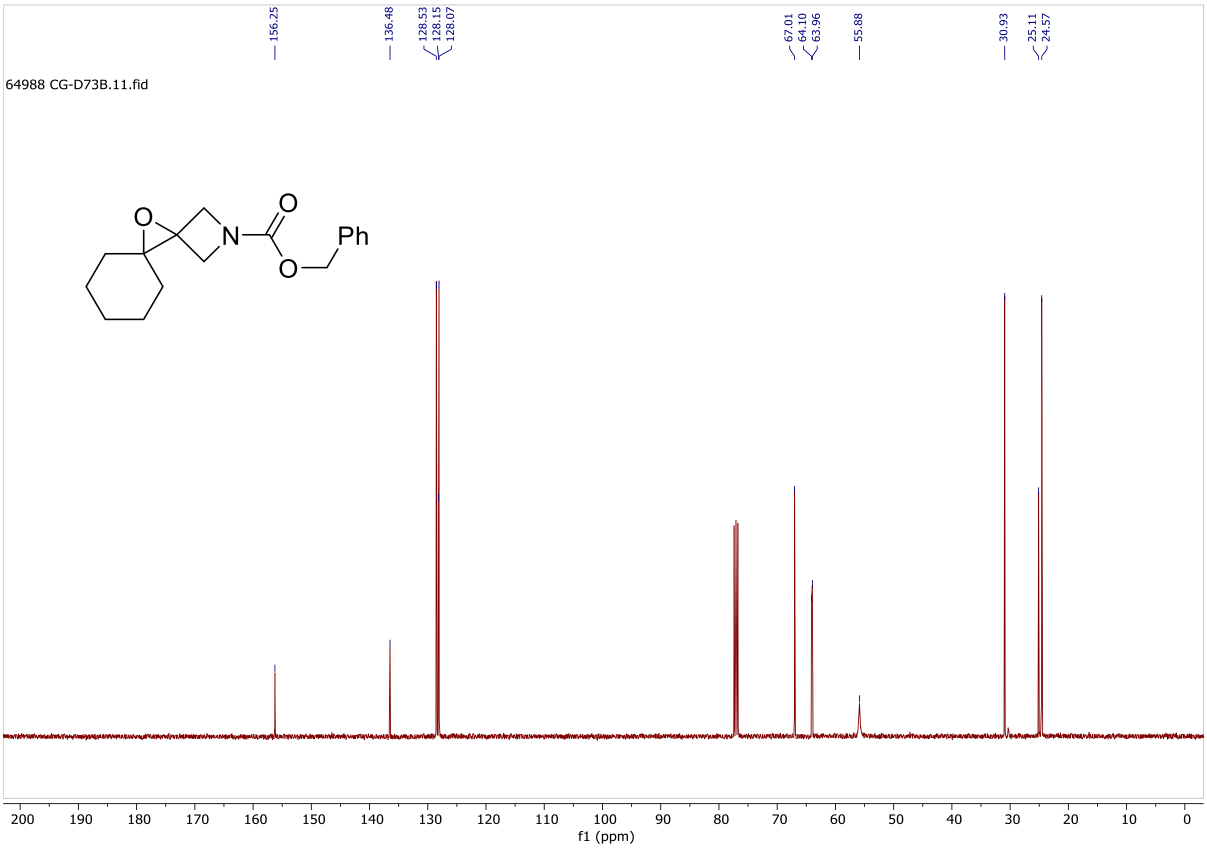

^{1}H NMR ($CDCl_3$, 400 MHz) of **8l**, See procedure

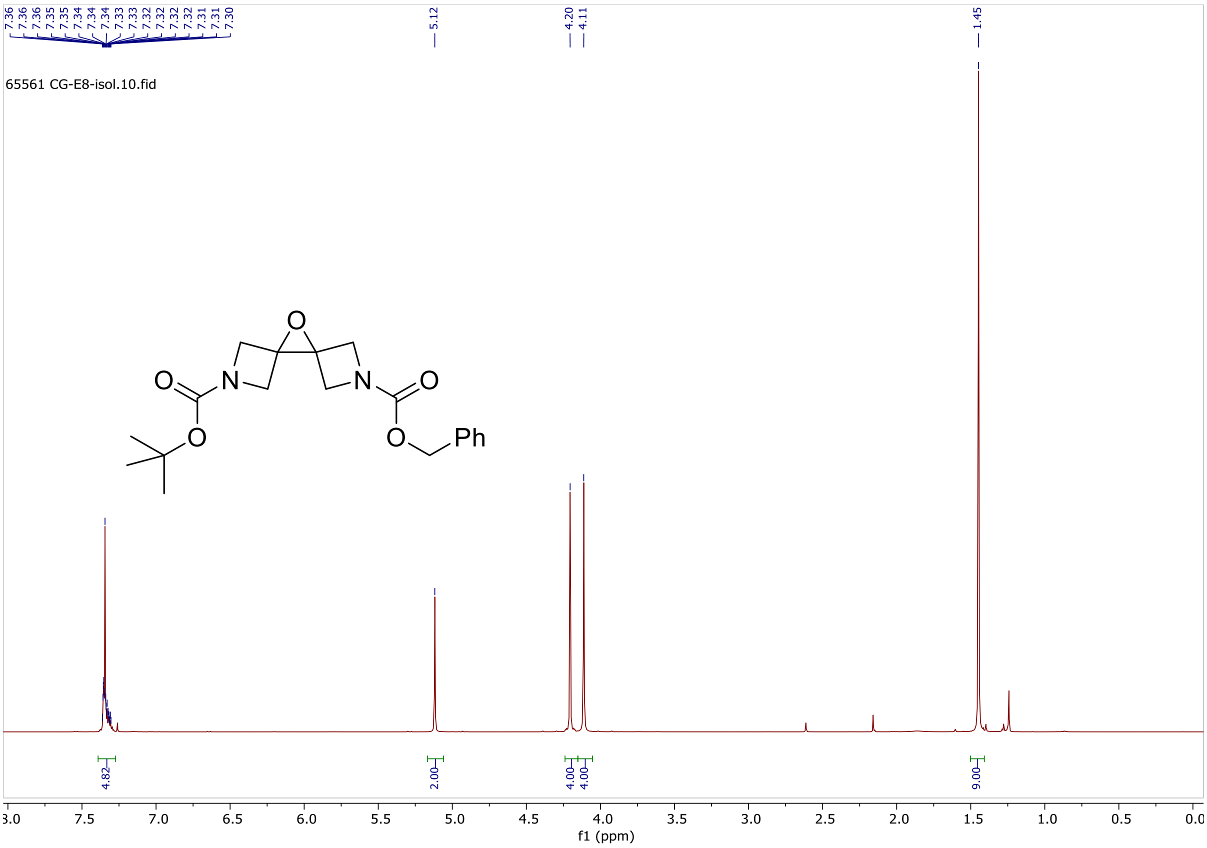

^{13}C NMR (CDCl$_3$, 101 MHz) of **8l**

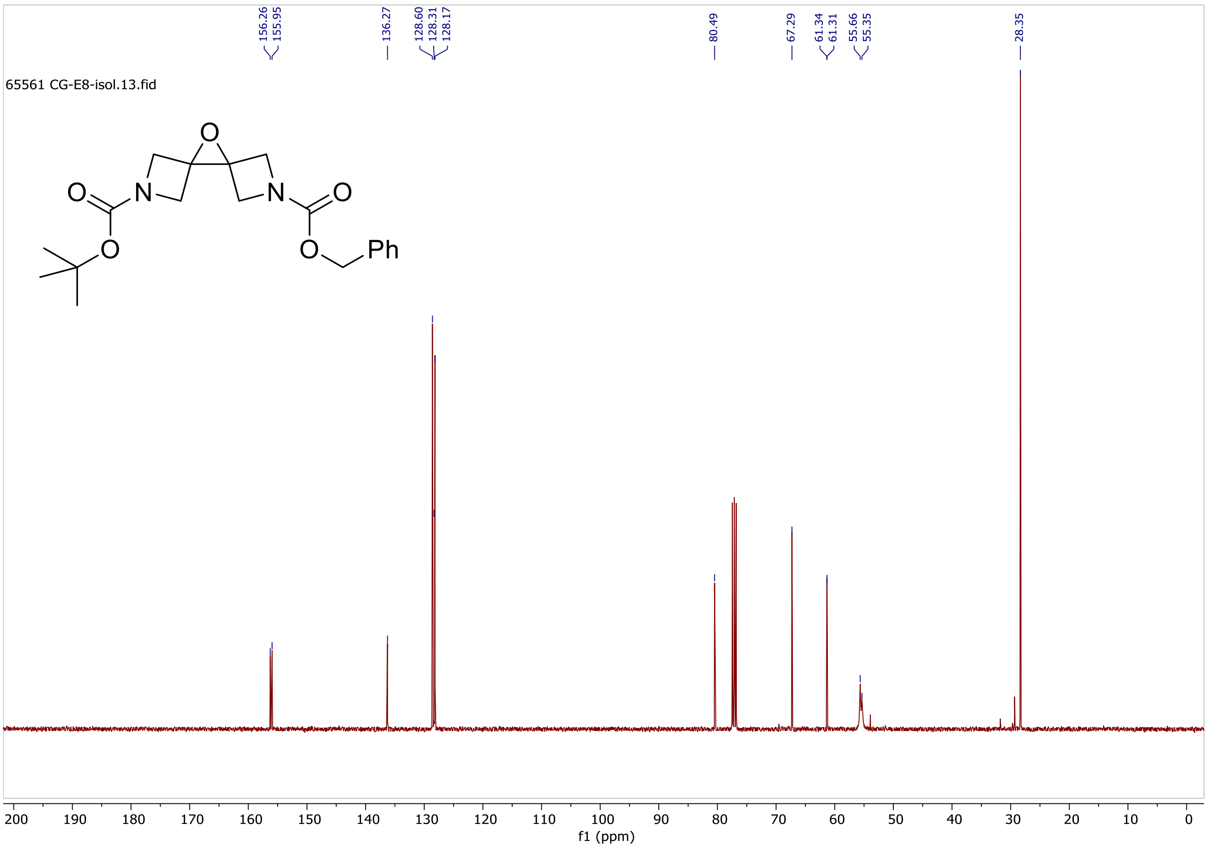

^{1}H NMR (CDCl$_3$, 400 MHz) of **8n**, See procedure

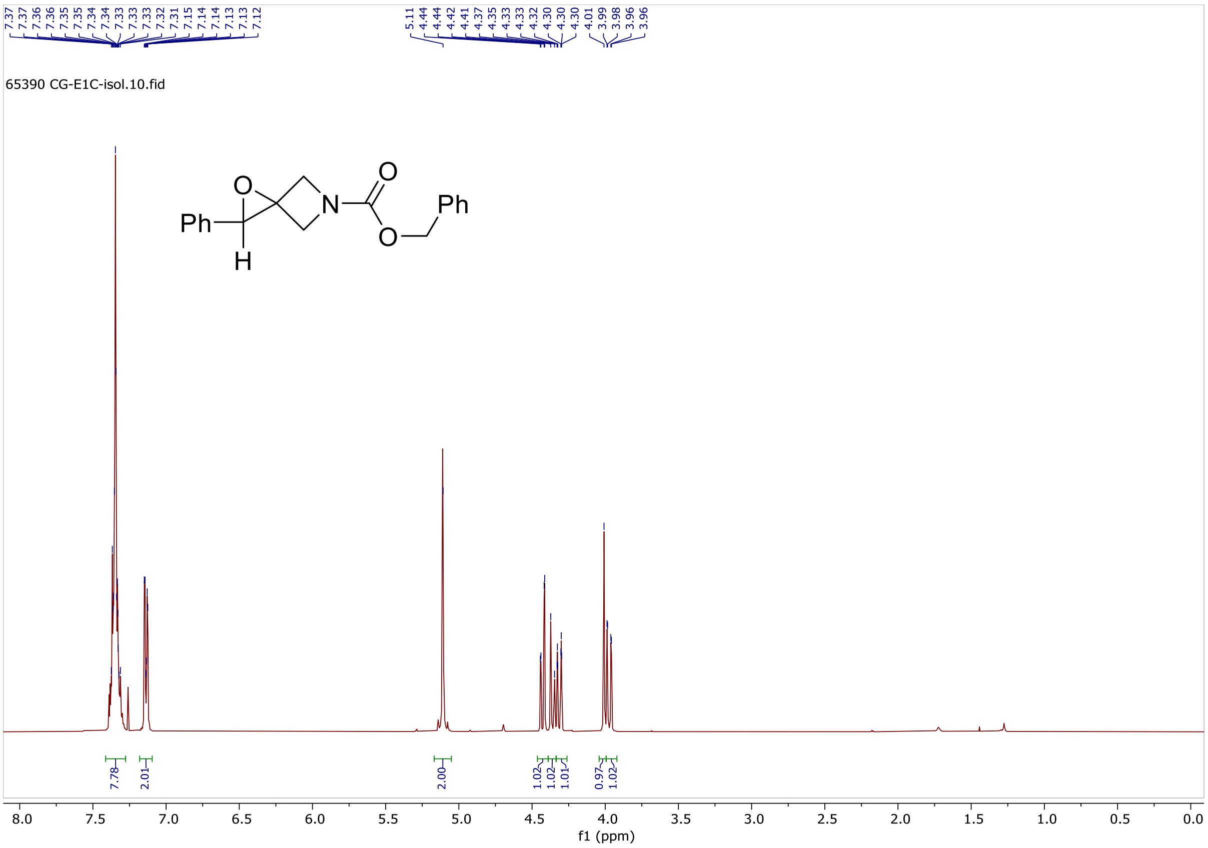

^{13}C NMR (CDCl$_3$, 101 MHz) of **8n**

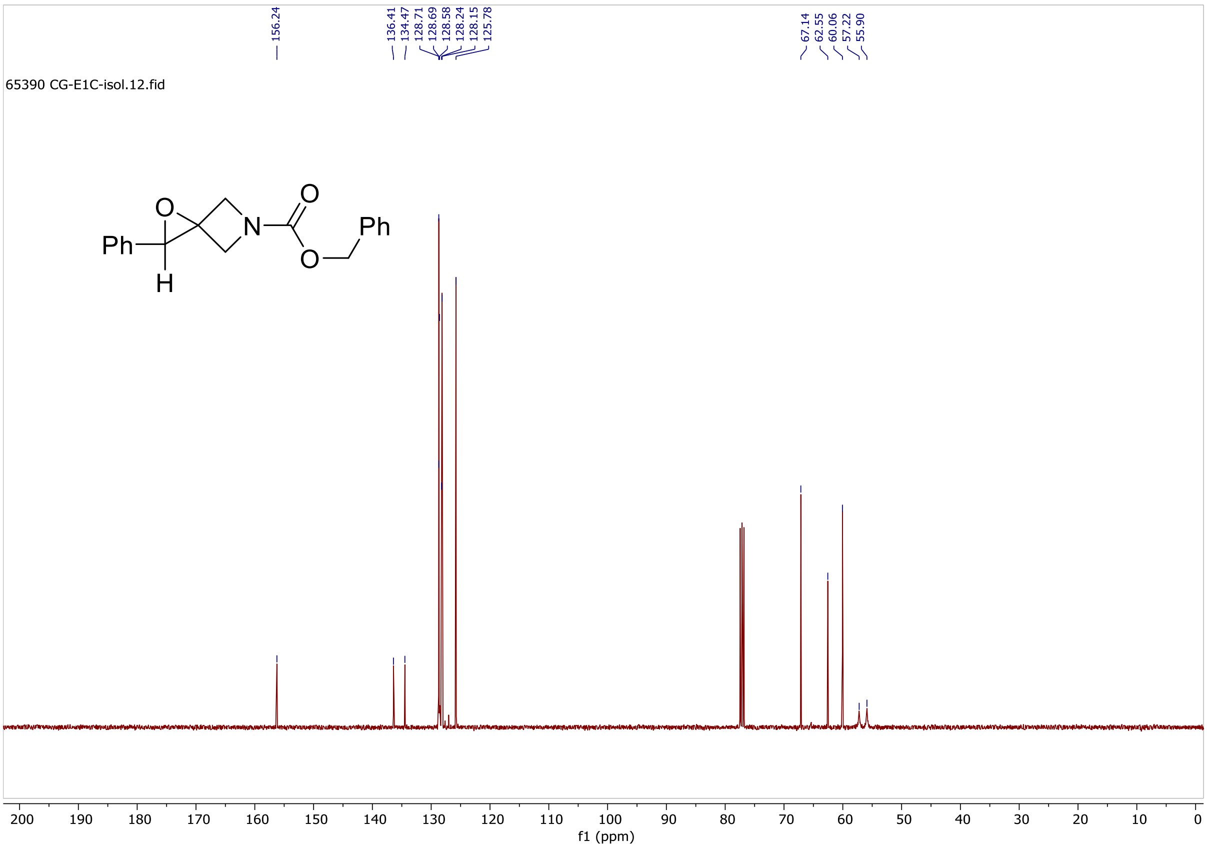

^{1}H NMR (CDCl$_3$, 400 MHz) of **8p'**, See procedure

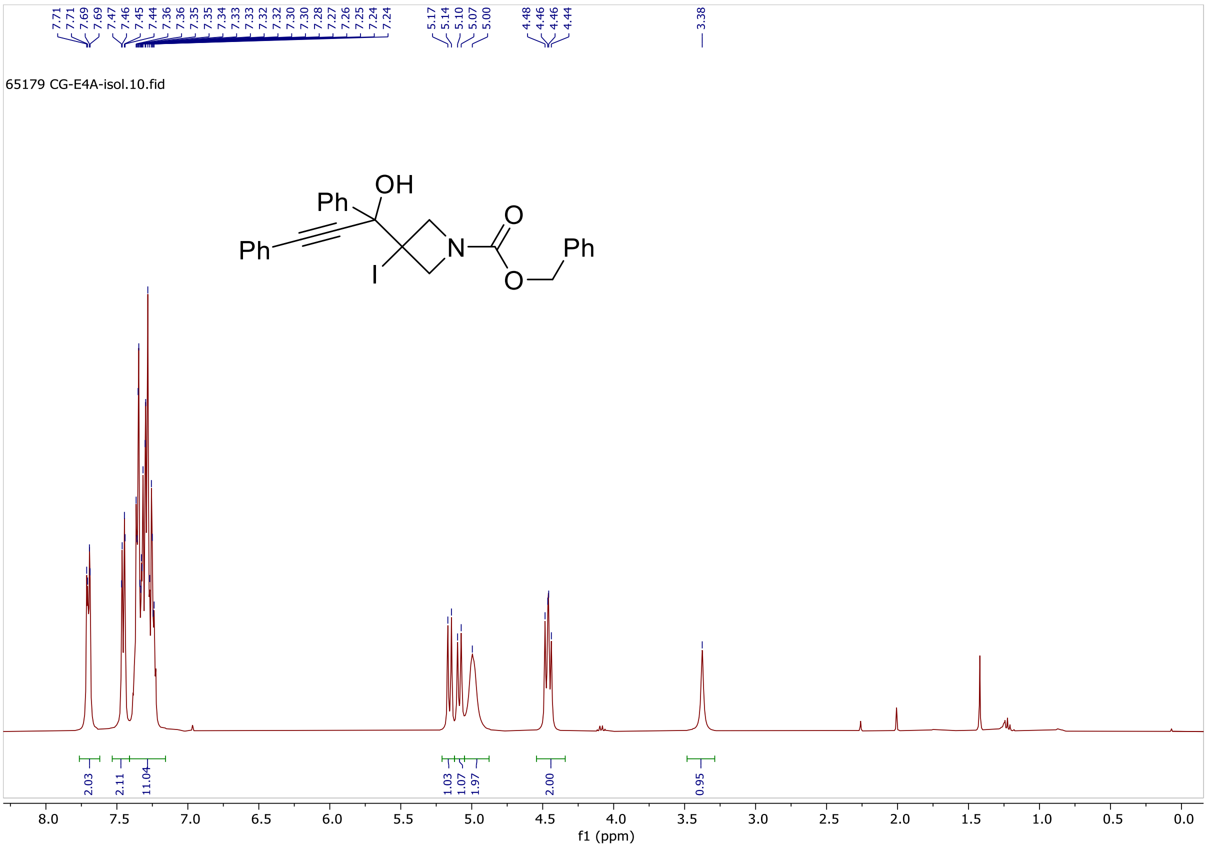

^{13}C NMR ($CDCl_3$, 101 MHz) of **8p’**

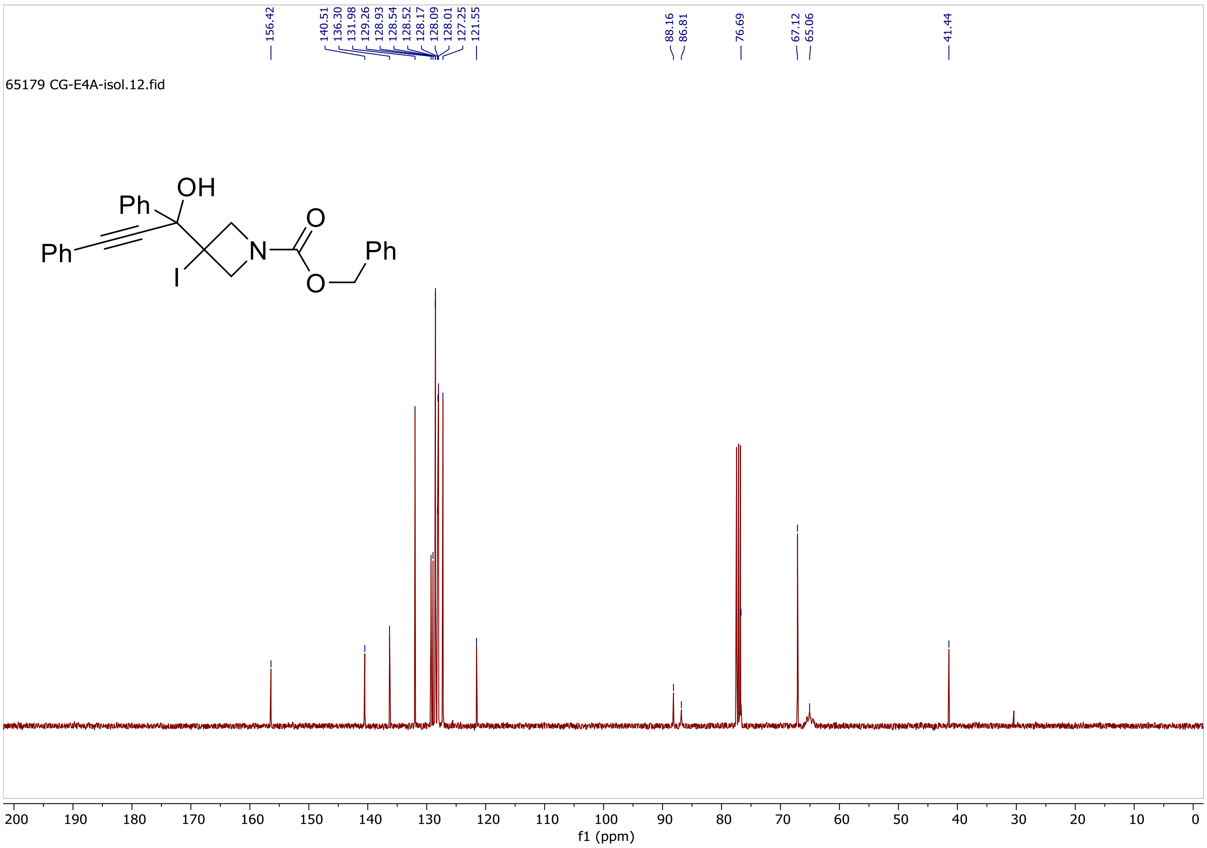

^{1}H NMR ($CDCl_3$, 400 MHz) of **8p**, See procedure

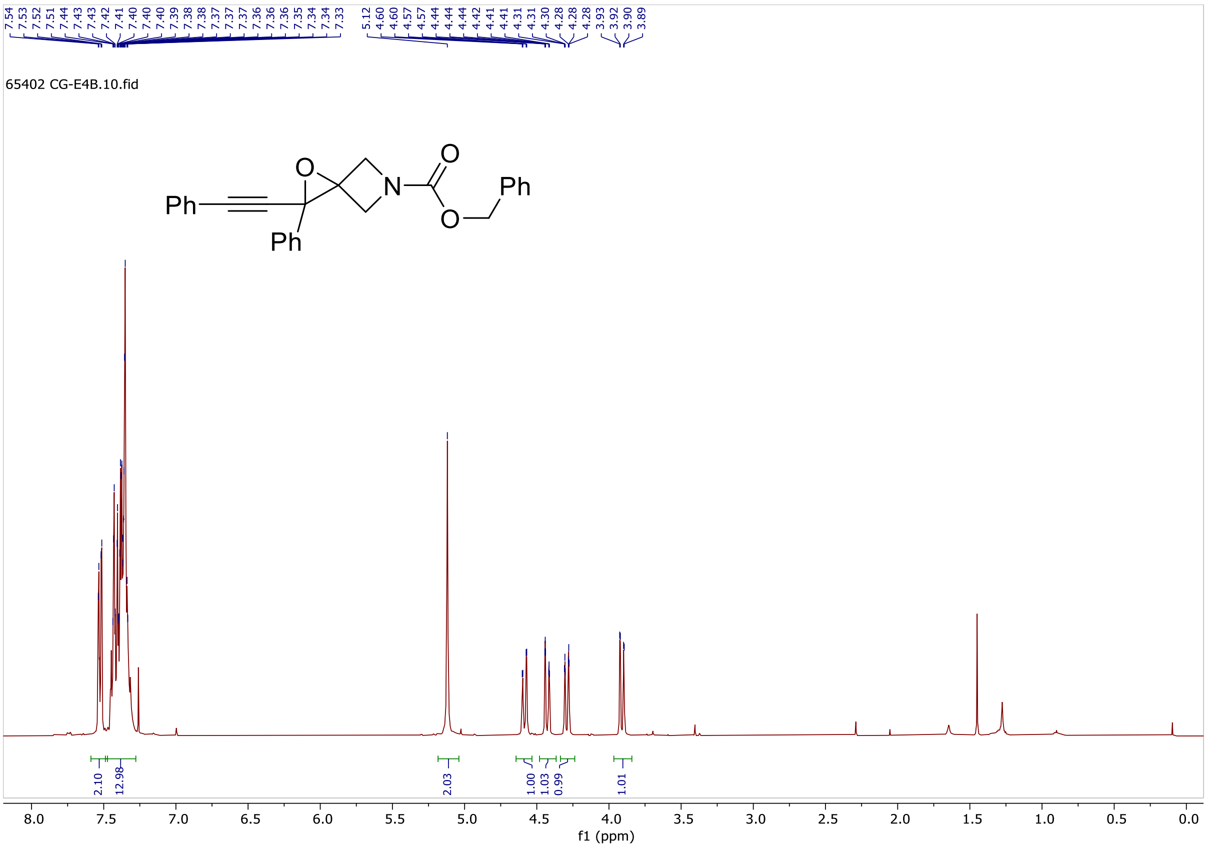

^{13}C NMR (CDCl$_3$, 101 MHz) of **8p**

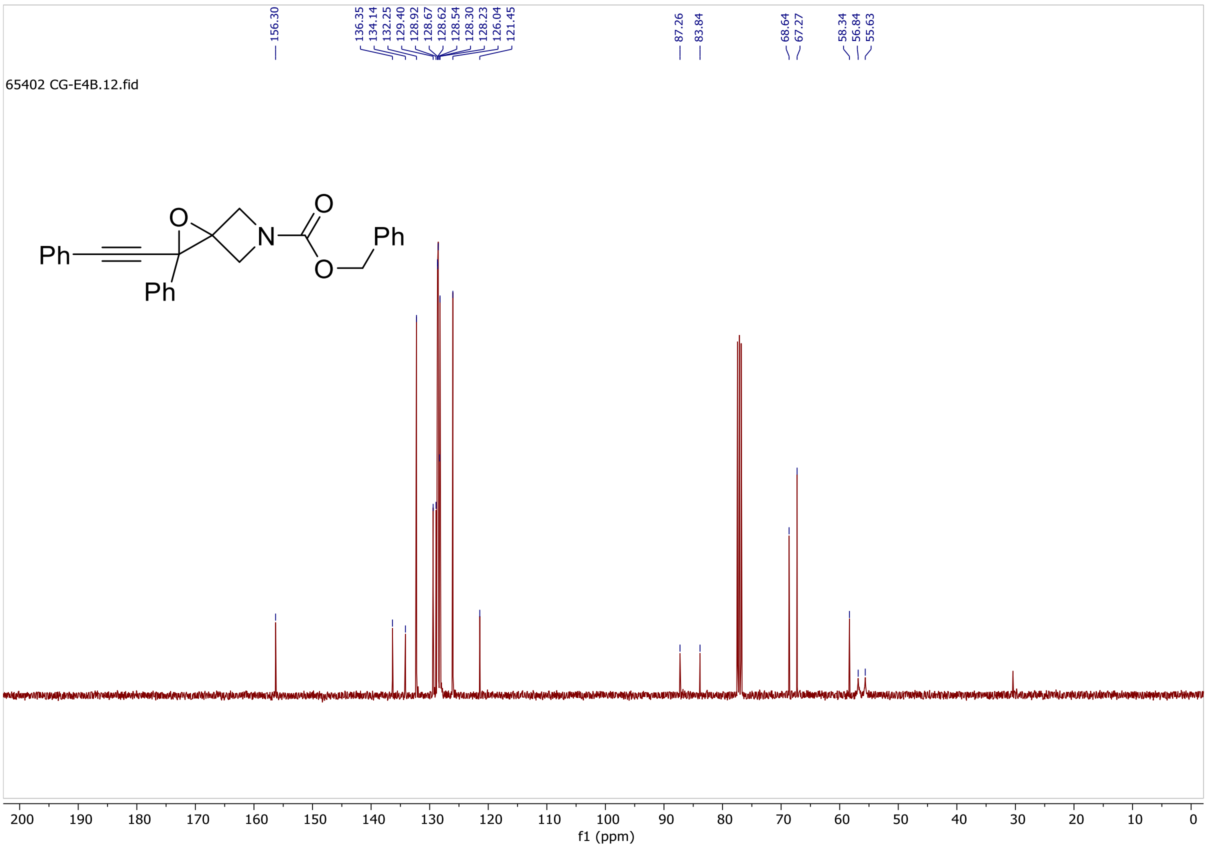

^{1}H NMR (CDCl$_3$, 400 MHz) of **8s**, See procedure

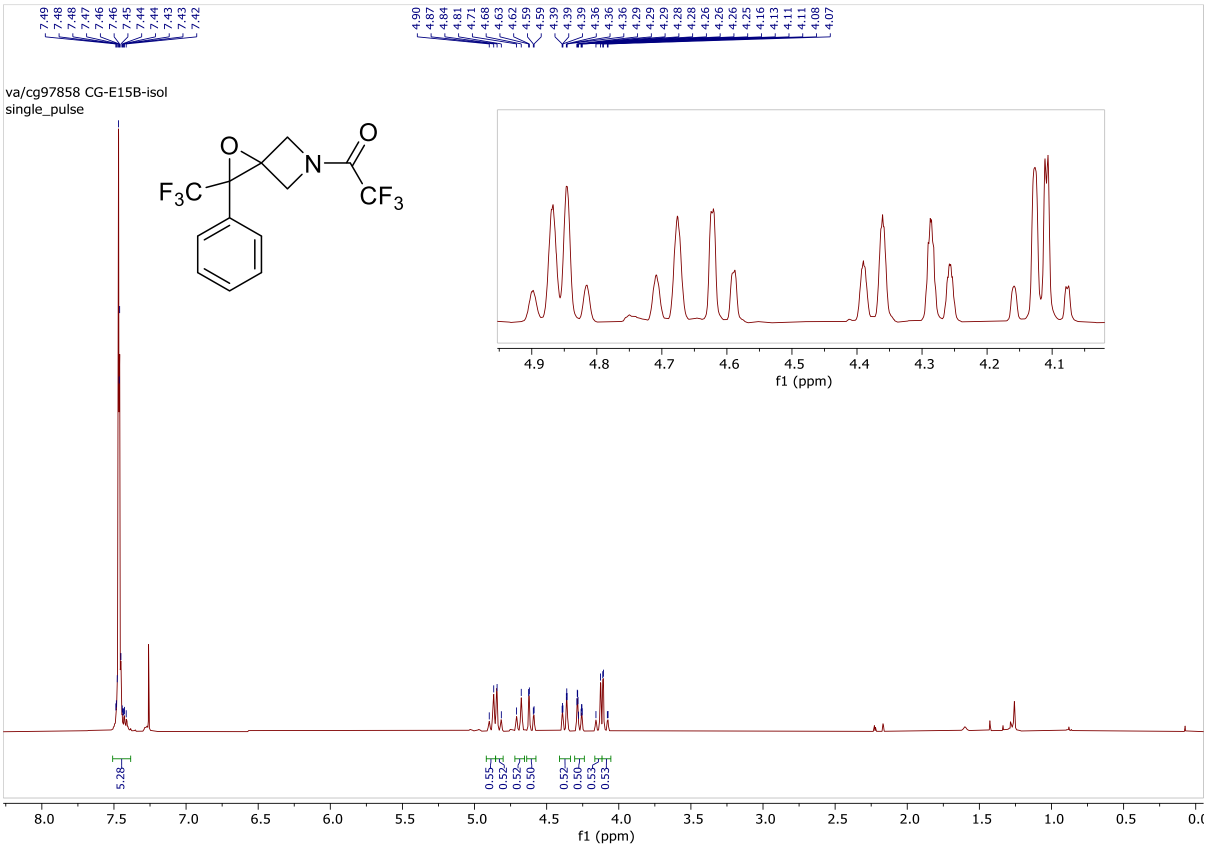

^{13}C NMR (CDCl$_3$, 101 MHz) of **8s**

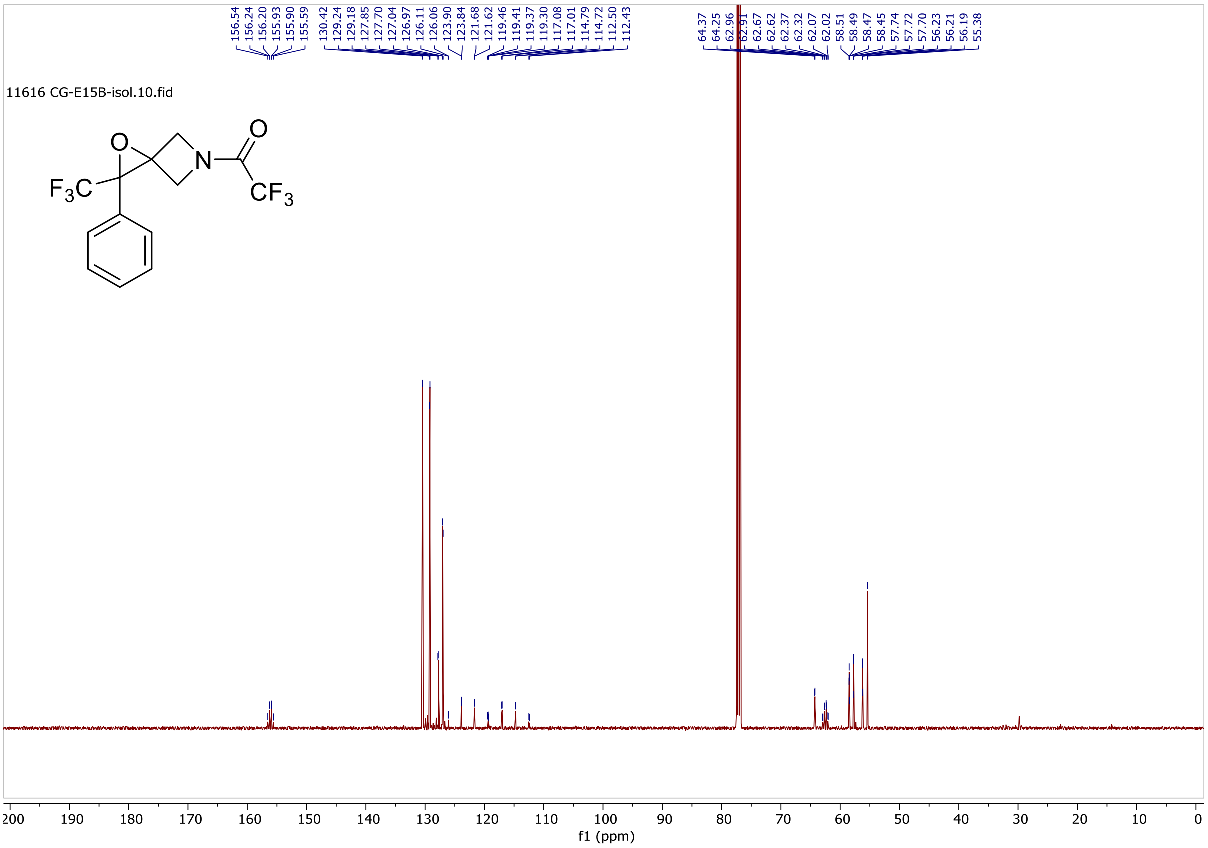

^{1}H NMR (CDCl$_3$, 400 MHz) of **14**, See procedure

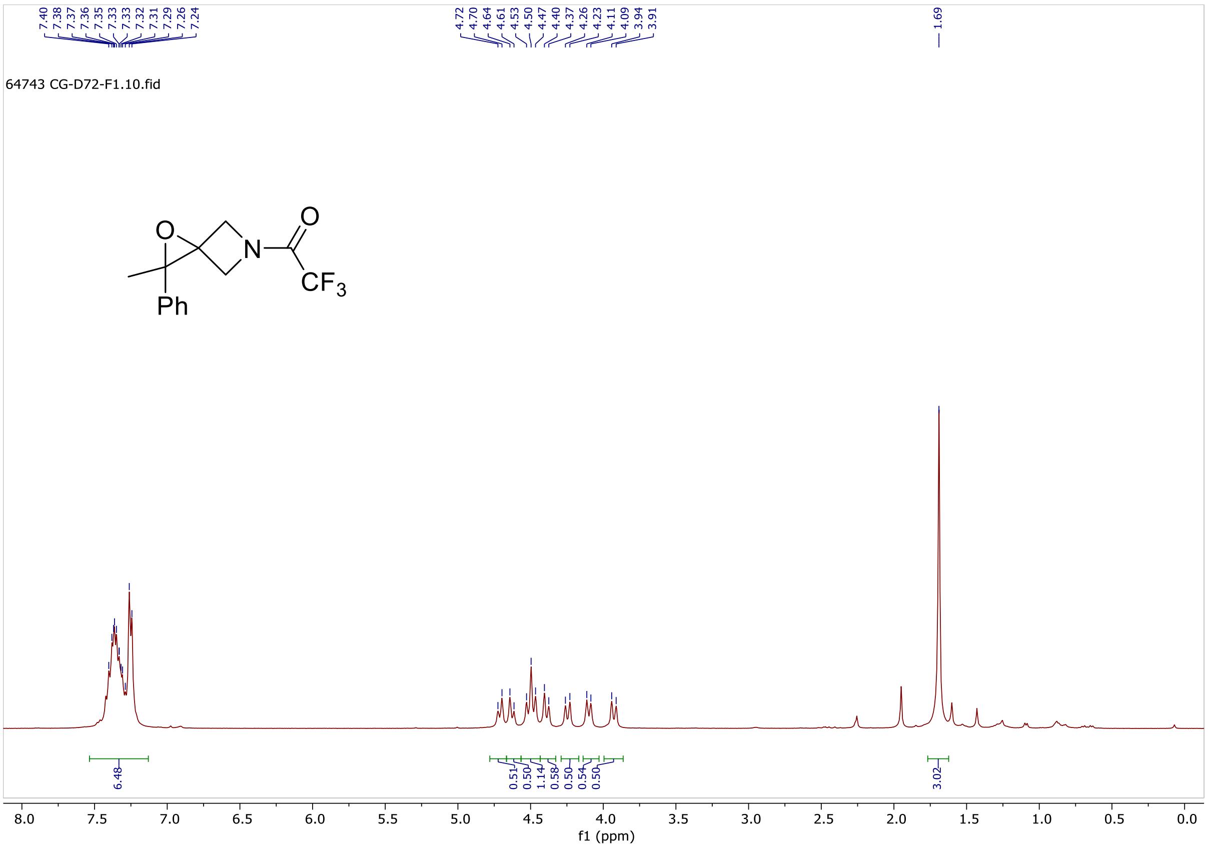

^{13}C NMR ($CDCl_3$, 101 MHz) of **14**

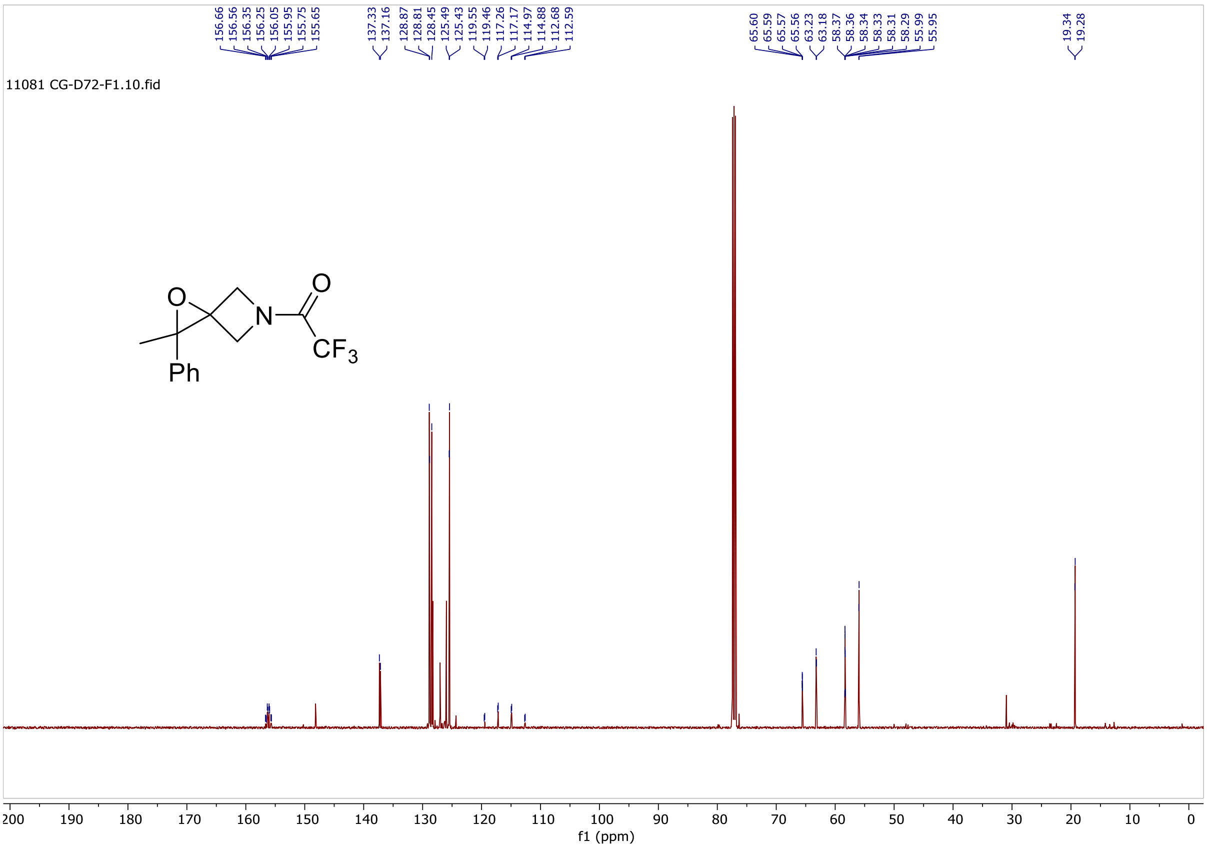

^{1}H NMR ($CDCl_3$, 400 MHz) of **15**, See procedure

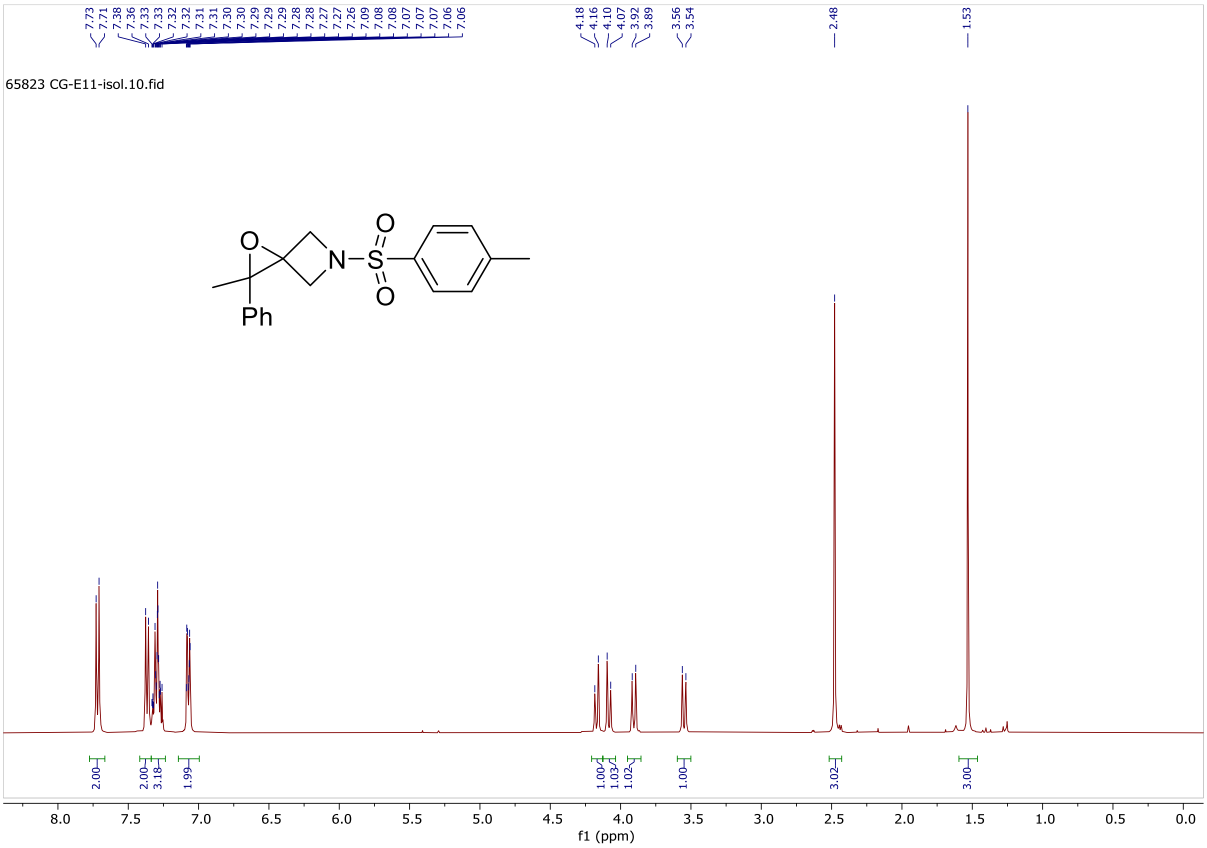

^{13}C NMR (CDCl$_3$, 101 MHz) of **15**

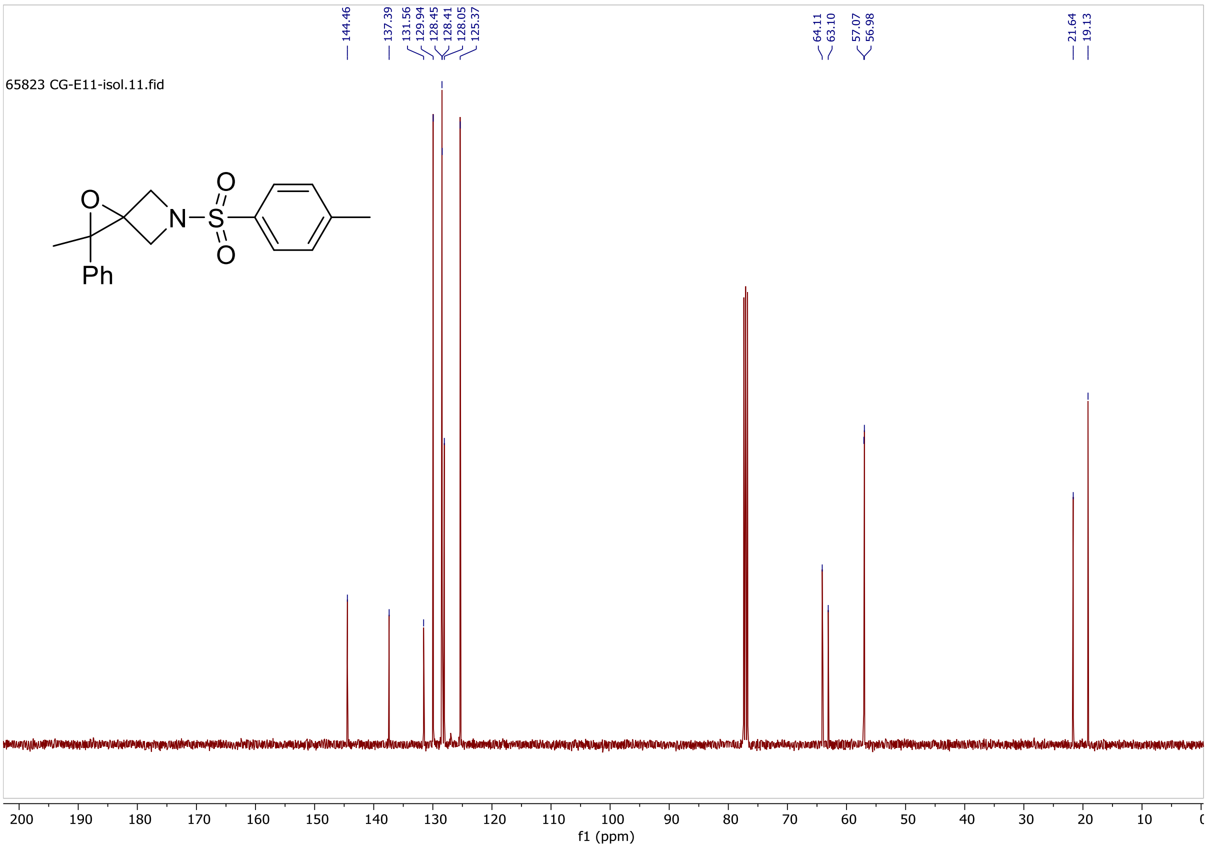

^{1}H NMR (CDCl$_3$, 400 MHz) of **16**, See procedure

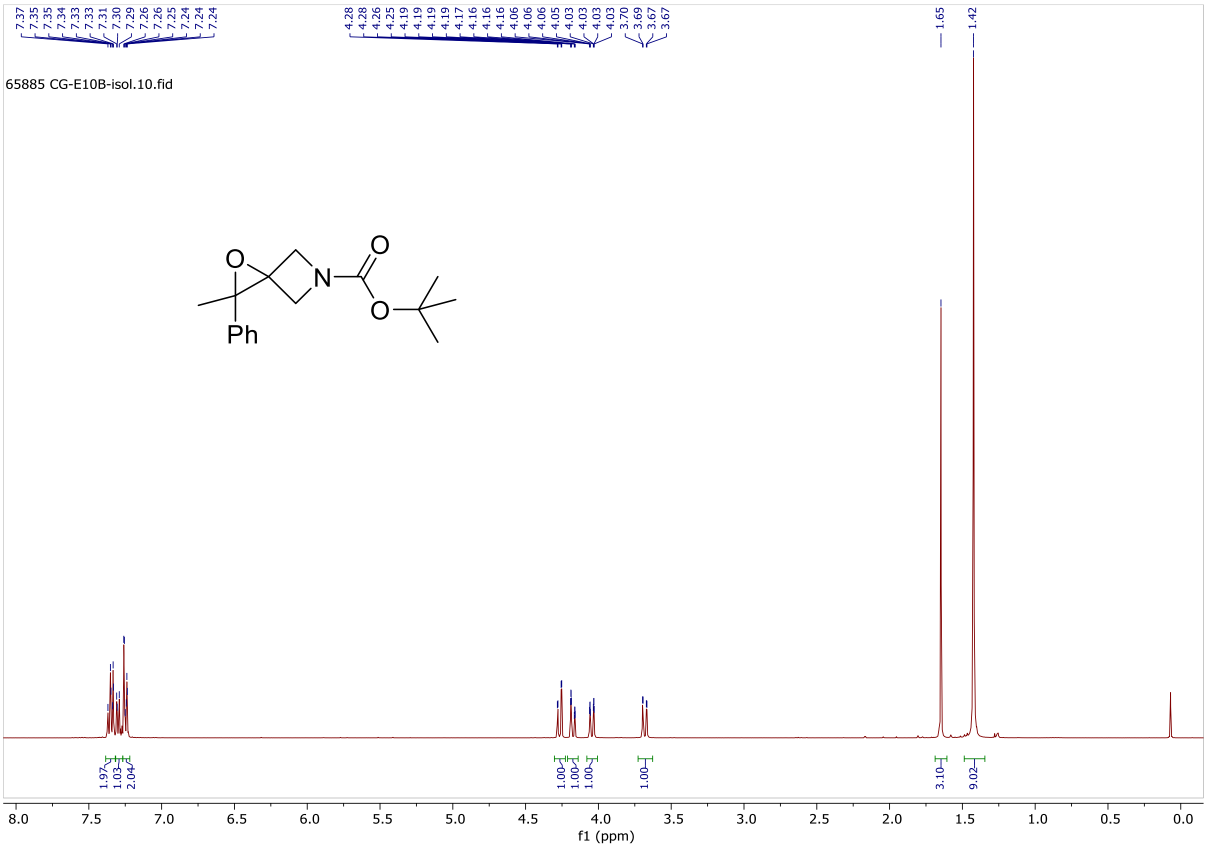

^{13}C NMR (CDCl$_3$, 101 MHz) of **16**

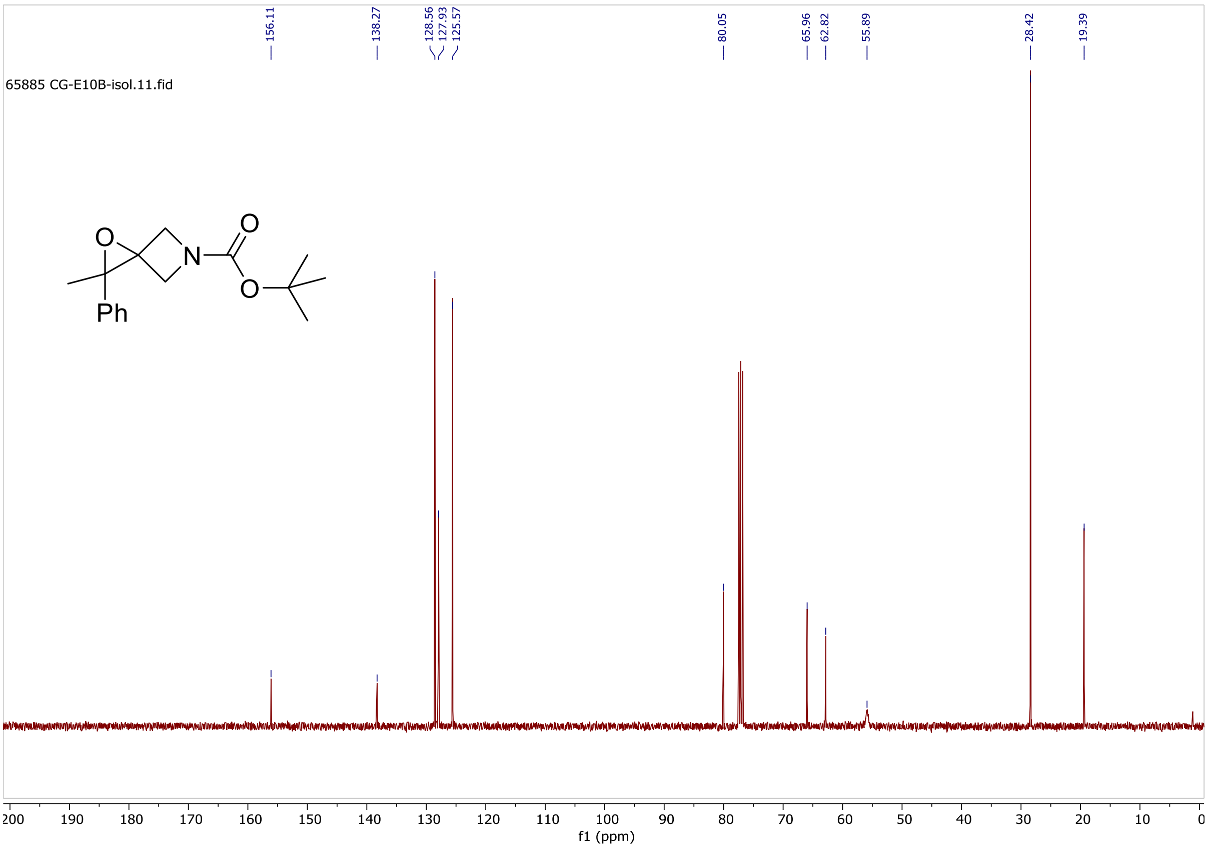

Supplement: Supplementary file 1 — Supplementary [file ANIE-60-7360-s001.pdf]
